# Patient Characteristics and Practice Variation Associated With New Community Prescription of Benzodiazepine and z‐Drug Hypnotics After Critical Illness: A Retrospective Cohort Study Using the UK Clinical Practice Research Datalink

**DOI:** 10.1002/pds.70056

**Published:** 2024-11-27

**Authors:** Elizabeth T. Mansi, Christopher T. Rentsch, Richard S. Bourne, Bruce Guthrie, Nazir I. Lone

**Affiliations:** ^1^ Usher Institute University of Edinburgh Edinburgh UK; ^2^ Faculty of Epidemiology and Population Health London School of Hygiene & Tropical Medicine London UK; ^3^ Department of Internal Medicine Yale School of Medicine New Haven USA; ^4^ Department of Pharmacy and Critical Care Sheffield Teaching Hospitals NHS Foundation Trust Sheffield UK; ^5^ Division of Pharmacy and Optometry School of Health Sciences, Faculty of Biology, Medicine and Health, the University of Manchester Manchester UK; ^6^ Advanced Care Research Centre, University of Edinburgh Edinburgh UK; ^7^ University Department of Anaesthesia, Critical Care, and Pain Medicine School of Clinical Sciences, University of Edinburgh Edinburgh UK

**Keywords:** benzodiazepines, critical care, critical illness, electronic health records, hypnotics and sedatives, postintensive care syndrome, prescriptions

## Abstract

**Purpose:**

Survivors of critical illness are often affected by new or worsened mental health conditions and sleep disorders. We examined the incidence, practice variation and factors associated with new benzodiazepine and z‐drug community prescriptions among critical illness survivors.

**Methods:**

A retrospective cohort study using the UK Clinical Practice Research Datalink data included 52 846 adult critical care survivors hospitalised in 2010 and 2018 who were not prescribed benzodiazepines or z‐drugs before hospitalisation. We performed multilevel multivariable logistic regression to assess patient factors associated with new (any prescription within 90 days) and with new‐and‐persistent (2+ prescriptions within 180 days) benzodiazepine or z‐drug prescribing, and to evaluate variation by primary care practice.

**Results:**

5.2% (2769/52846) of treatment‐naïve survivors (95% CI 5.1–5.4) were prescribed a benzodiazepine or z‐drug, and 2.5% (1311/52846) had new‐and‐persistent prescribing. A history of insomnia (adjusted OR 1.96; 95% CI 1.74–2.21), anxiety or depression (adjusted OR 1.40; 95% CI 1.28–1.53) and recent prescription opioid use (adjusted OR 1.47; 95% CI 1.34–1.61) were associated with new community prescription. Sex was not associated with new prescriptions and older patients were less likely to receive a prescription. 2.6% of the variation in new prescribing and 4.1% of the variation in new‐and‐persistent prescribing were attributable to the prescribing practice.

**Conclusions:**

One in twenty critical illness survivors receive a new community benzodiazepine or z‐drug prescription. Further research is needed to understand where in the patient care pathway initiation occurs and the risk of adverse events in survivors of recent critical illness.

**Summary**

- One in 20 adults hospitalised for critical illness were prescribed a new benzodiazepine or z‐drug within 90 days of hospital discharge by their primary care provider. Almost half of those prescribed received more than one prescription.
- Zopiclone was the most common drug prescribed (50%) followed by diazepam (19%).
- Patient‐level factors associated with benzodiazepine or z‐drug prescription included a history of insomnia, anxiety or depression and recent opioid prescription.
- There was low‐to‐moderate variation in prescription incidence between primary care practices (intraclass correlation of 2.6% for one prescription and 4.1% for more than one prescription).

## 1 Introduction

Millions of patients survive critical illness globally every year [1], but approximately half of survivors of critical illness (defined by admission to a hospital intensive care unit (ICU)) suffer from anxiety, depression, posttraumatic stress disorder or sleep disturbances in the year after their critical illness [2, 3, 4, 5, 6, 7, 8, 9, 10, 11]. Psychological conditions such as these are associated with functional impairment and increased healthcare utilisation in medically unwell populations [10]. Sleep disturbances experienced by many ICU survivors may also exacerbate psychological symptoms and decrease quality of life [12].

Benzodiazepines and z‐drugs (e.g., zopiclone, zolpidem and zaleplon) are anxiolytic and hypnotic medicines prescribed globally [13, 14], despite their known adverse effects of falls, fractures, road traffic accidents and dependency [15, 16, 17, 18]. They are most often prescribed for pharmacological treatment of anxiety or insomnia. There is interest in quantifying the incidence of such medications prescribed to survivors of critical illness as these patients may be physiologically vulnerable to adverse drug effects. Survivors of critical illness may be more likely to receive new prescriptions for such medicines for two main reasons: (1) Treatment for anxiety or sleep disturbances commonly recognised after surviving a critical illness [19, 20] and (2) Potentially inappropriate continuation after initiation in the hospital [21, 22]. Understanding which survivors are more likely to receive new community benzodiazepines and z‐drug hypnotics after critical illness may help target medicines optimisation processes and improve patient outcomes.

Previous research has been restricted to patients over 65 years of age or lacked patient details such as comorbidities or co‐prescribing prior to hospital admission [23, 24, 25, 26]. This study aimed to identify patient factors (including preadmission characteristics) associated with new and new‐and‐persistent community benzodiazepines or z‐drug hypnotic prescriptions among survivors of critical illness. Additionally, we examined variation in prescribing by primary care practices.

## 2 Methods

### 2.1 Study Design, Setting and Participants

**FIGURE 1:**
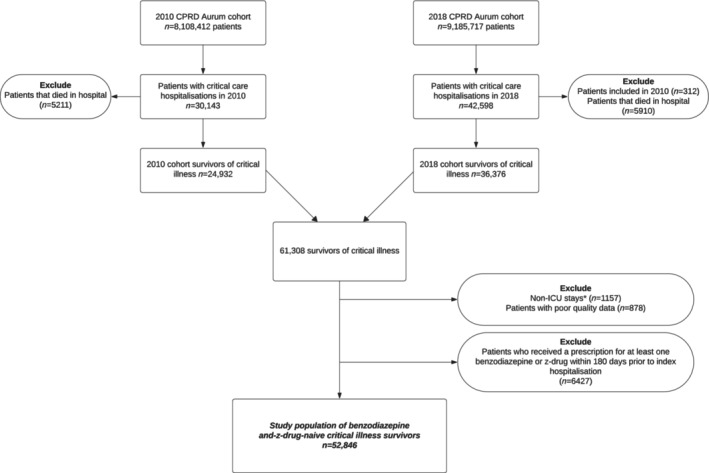
Patient flow diagram. CPRD: Clinical Practice Research Datalink; ICU: Intensive care unit. *Non‐ICU stays were patients who were provided critical care outside a typical ICU (e.g., ward).

**FIGURE 2:**
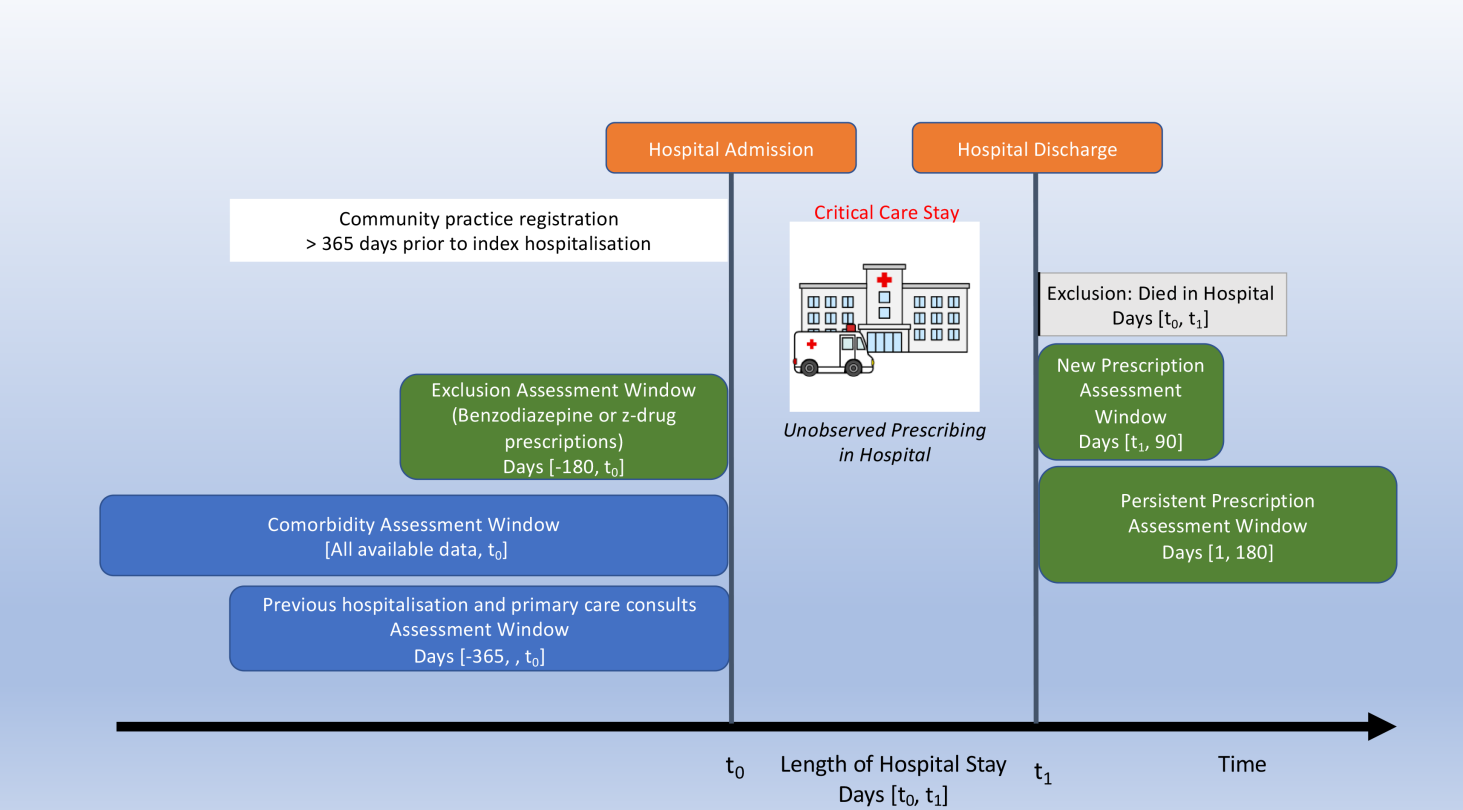
Study diagram.

This retrospective cohort study was a secondary analysis of a dataset assembled for a larger study investigating multimorbidity [27]. The multimorbidity study was comprised of two large cohorts of patients who were registered with a Clinical Practice Research Datalink (CPRD) Aurum‐participating primary care practice in the United Kingdom on 1 January 2010 and 1 January 2018. CPRD includes routinely collected data and prescriptions from primary care practices (over 19 million UK patients in 2018) [28]. The data are representative of the population by sex, age and socioeconomic deprivation [28]. In the UK, primary care practices are responsible for almost all community prescribing (including medicines initiated/recommended by specialists). To be included, patients were aged 18 years and over and were registered with their primary care practice for at least 1 year prior to their respective cohort entry dates. Individual patients within a CPRD‐registered primary care practice were not included if they opted out of participation. For our study, we included only patients who were admitted to a hospital ICU in the same calendar year as their cohort and who survived to hospital discharge (“index hospitalisation”). For patients with more than one hospitalisation with critical care, only the first hospitalisation was selected so that all individuals were represented only once. Patients not meeting minimum data quality standards (e.g., missing sex or age) were excluded. Patients who received a benzodiazepine or z‐drug prescription in the 180 days prior to index hospitalisation were excluded, as they were not at risk for new community prescription (Figure 1). Patients were followed up for 180 days after index hospital discharge for outcome assessments (Figure 2).

### 2.2 Data Sources and Linkage

The following datasets were linked by anonymised patient identifier by the Trusted Third‐Party National Health Service (NHS) England: CPRD Aurum (primary care diagnoses and prescription data), Hospital Episode Statistics Admitted Patient Care (HES APC, hospitalisation data including critical care), office for National Statistics (ONS, death registration data) and Index of Multiple Deprivation (IMD, socioeconomic data). These data sources have been previously described in detail [28, 29, 30]. CPRD data include the quantity and strength of prescribed medication but does not record the indication or duration of prescriptions. Access to the data was approved by CPRD (ref. 23_002860), which oversees all aspects of information governance, ethics and confidentiality for studies accessing anonymised healthcare data held by CPRD.

*Outcomes*: The primary outcome of interest was a new community prescription of a benzodiazepine or z‐drug within 90 days of index hospital discharge (binary). The secondary outcome was new‐and‐persistent prescribing, defined by receiving two or more benzodiazepine or z‐drug prescriptions within 180 days of index hospital discharge when the first prescription was within 90 days of index hospital discharge. The first prescription could be any benzodiazepine or z‐drug, and subsequent prescriptions not limited to the same prescription by generic name or drug class to be classified as new and persistent. For example, if a patient received one prescription for diazepam on day 30, without a subsequent prescription within 180 days from hospital discharge, this was considered only a new prescription. Conversely, if a patient received one prescription for diazepam on day 45, and later a prescription for diazepam (or zopiclone) within 180 days from index discharge, this would be classified as both new and new‐and‐persistent prescribing. Prescriptions did not have to be dispensed to be counted (dispensing data is not present in these data).

*Patient covariates*: Demographic factors included sex, age, and ethnicity, which were obtained from the CPRD Aurum dataset. Ethnicity was reported in accordance with UK government recommendations and census data [31]. Socioeconomic status was measured using the 2019 IMD quintiles of the patient's postcode. We considered variables prior to index hospitalisation such as patient comorbidities, community prescribing data for opioids and gabapentinoids in the 180 days prior to index hospitalisation, and number of hospitalisations and number of primary care consultations in the 365 days prior to index hospitalisation. To have categories containing approximately the same number of patients, we categorised the number of previous hospital admissions into 0, 1, 2, 3 or more, and the number of previous primary care consultations into 0–2, 3–7, 8–14 and 15 or more.

Index hospitalisation variables obtained from HES APC included year of hospitalisation (2010 or 2018), method of hospital admission, primary diagnosis, ICU type, total organ systems supported (aggregate of cardiovascular, respiratory and renal support), out‐of‐hours discharge from ICU (based on discharge between 22:00 and 08:00 of last ICU stay for those with more than one per index hospitalisation) and total hospital length of stay. Primary diagnosis for index hospitalisation was categorised into 10 categories defined by International Classification of Diseases—Tenth Edition (ICD‐10) codes: circulatory (I00‐I99), neoplasms (C00‐D49), digestive (K00‐K95), injury or poisoning (S00‐T88), respiratory (J00‐J99), musculoskeletal (M00‐M99), genitourinary (N00‐N99), infectious (A00‐B99), symptoms, signs and abnormal clinical and laboratory findings, not elsewhere classified (R00‐R99) and all other conditions. The number of comorbidities at index hospitalisation was based on a modified count of the Elixhauser‐defined list of comorbidities [32] using all available hospital diagnosis codes (ICD‐10) and primary care diagnosis codes (UK Read codes) prior to index. We modified the Elixhauser comorbidity count by removing three mental health diagnoses which were instead included as individual binary covariates (psychoses, alcohol misuse, drug misuse). We additionally removed depression from the Elixhauser count and fitted a binary covariate for ‘depression or anxiety’, and a binary covariate for insomnia (see Appendix S1 for codelists).

### 2.3 Post Hoc Analysis

For patients who received a new benzodiazepine or z‐drug prescription, we assessed primary care diagnoses of anxiety, depression and insomnia within 90 days of hospital discharge (not included in multivariable models).

### 2.4 Statistical Methods

Patient characteristics were reported using proportions or medians with interquartile ranges (IQR). For the proportions of patients prescribed a new benzodiazepine or z‐drug, all patients were kept in the denominator (even those that died or were rehospitalised within 90 days of index discharge) and 95% confidence intervals (CIs) were determined by the one‐sample proportions test with Yates continuity correction in R. Associations between patient factors and outcomes were evaluated using multilevel (mixed‐effects) multivariable logistic regression models of patients nested within the prescribing primary care practice. Adjusted odds ratios (ORs) with 95% CI were estimated for patient characteristics using the *finalfit* package in R. Covariates were selected based on the literature and clinical expertise, availability in our data sources, and those that would have been available at time of index hospital discharge. Covariates included in adjusted models were sex, age group, ethnicity, IMD quintile, modified Elixhauser comorbidity category, history of insomnia, anxiety/depression, psychoses, alcohol misuse, drug misuse, opioid prescription, gabapentinoids prescription, number of hospital admission category, number of primary care consultations category, year of hospital admission, hospital admission method, primary condition at index hospitalisation, ICU type, total organs supported in ICU, out‐of‐hours ICU discharge and hospital length of stay category. The intraclass correlation (ICC—a measure of the proportion of variation in outcome attributable to prescribing primary care practices) was calculated before and after adjustment for patient‐level characteristics [33]. Patients with missing data (7.6%) were excluded from multivariable analyses. All analyses were performed using R version 4.1.1.

### 2.5 Sensitivity Analysis

We conducted sensitivity analyses excluding patients who died or were readmitted to hospital in the first 30 and 90 days from index hospital discharge. We did this because there may be local variation in discharge pathways for patients receiving palliative care and because patients in the hospital are not at risk for community prescribing. We further conducted a sensitivity analysis by redefining treatment‐naïve patients as those who did not receive a benzodiazepine or z‐drug prescription within 365 days of index hospital admission.

## 3 Results

There were 52 846 adults naïve to benzodiazepines or z‐drugs prior to hospitalisation who survived critical illness to hospital discharge (42% women, Table 1). Patient median age was 66 years (IQR 53–75), and most patients (88%) were of white ethnicity. Opioids were prescribed within 180 days before index hospitalisation in 27% of patients. Sixty percent of the study population were hospitalised in 2018, with 40% hospitalised in 2010. The most common main condition for index hospitalisation involved circulatory diseases (29%), followed by neoplasms (19%). Most patients (71%) had a critical care stay in a general/medical/surgical ICU while 20% had a stay in a cardio/thoracic ICU. More than half (61%) of critical illness survivors had two or more organ systems supported (cardiovascular, respiratory or renal systems) in ICU. The median hospital length of stay was 9 days (IQR 5–18 days). Among patients prescribed a benzodiazepine or z‐drug within 90 days of hospital discharge, 19.8% (547/2769) received a diagnosis of anxiety, depression or insomnia within the same time period and 54.5% (1508/2769) had a history *or* new diagnosis of anxiety, depression or insomnia (*post hoc* analysis).

**TABLE 1:** Study population sociodemographic, comorbidity, and hospital characteristics.

Patient characteristics		Total (%) *n* = 52 846	No Rx (%) *n* = 50 077	Received Rx (%) *n* = 2769
Sex	Male	30 516 (57.7)	28 898 (57.7)	1618 (58.4)
	Female	22 330 (42.3)	21 179 (42.3)	1151 (41.6)
Age group	18–49	11 055 (20.9)	10 522 (21.0)	533 (19.2)
	50–64	13 992 (26.5)	13 192 (26.3)	800 (28.9)
	65–79	20 316 (38.4)	19 239 (38.4)	1077 (38.9)
	80+	7483 (14.2)	7124 (14.2)	359 (13.0)
Ethnicity	Asian	2905 (5.5)	2784 (5.6)	121 (4.4)
	Black	2053 (3.9)	1999 (4.0)	54 (2.0)
	Mixed	311 (0.6)	300 (0.6)	11 (0.4)
	White	46 515 (88.4)	43 962 (88.2)	2553 (92.5)
	Other	835 (1.6)	813 (1.6)	22 (0.8)
	Missing	227	219	8
IMD quintile	1, Least deprived	9964 (18.9)	9428 (18.9)	536 (19.4)
	2	10 346 (19.6)	9750 (19.5)	596 (21.5)
	3	10 133 (19.2)	9610 (19.2)	523 (18.9)
	4	10 993 (20.8)	10 458 (20.9)	535 (19.3)
	5, Most deprived	11 327 (21.5)	10 751 (21.5)	576 (20.8)
	Missing	83	80	3
Modified Elixhauser comorbidity count^a^	None	7034 (13.3)	6726 (13.4)	308 (11.1)
	1	9882 (18.7)	9399 (18.8)	483 (17.4)
	2	9750 (18.4)	9191 (18.4)	559 (20.2)
	3	8427 (15.9)	7969 (15.9)	458 (16.5)
	4	6550 (12.4)	6185 (12.4)	365 (13.2)
	5 or more	11 203 (21.2)	10 607 (21.2)	596 (21.5)
History of insomnia		4082 (7.7)	3657 (7.3)	425 (15.3)
History of anxiety or depression		16 457 (31.1)	15 321 (30.6)	1136 (41.0)
History of psychoses		1088 (2.1)	1008 (2.0)	80 (2.9)
History of alcohol misuse		6299 (11.9)	5879 (11.7)	420 (15.2)
History of drug misuse		1262 (2.4)	1165 (2.3)	97 (3.5)
History of opioid prescription		14 194 (26.9)	13 165 (26.3)	1029 (37.2)
History of gabapentin/pregabalin prescription		3215 (6.1)	3005 (6.0)	210 (7.6)
Number of hospital admissions (1 year)	None	17 871 (33.8)	17 075 (34.1)	796 (28.7)
	1	14 259 (27.0)	13 526 (27.0)	733 (26.5)
	2	8205 (15.5)	7693 (15.4)	512 (18.5)
	3+	12 511 (23.7)	11 783 (23.5)	728 (26.3)
Number of primary care consultations (1 year)	0–2	13 224 (25.0)	12 628 (25.2)	596 (21.5)
	3–7	12 610 (23.9)	12 060 (24.1)	550 (19.9)
	8–14	14 169 (26.8)	13 420 (26.8)	749 (27.0)
	15+	12 843 (24.3)	11 969 (23.9)	874 (31.6)
Year of hospital admission	2010	21 110 (39.9)	19 752 (39.4)	1358 (49.0)
	2018	31 736 (60.1)	30 325 (60.6)	1411 (51.0)
Hospital admission method	Elective	23 431 (44.3)	22 246 (44.4)	1185 (42.8)
	Emergency	25 539 (48.3)	24 134 (48.2)	1405 (50.7)
	Transfer/Other	3873 (7.3)	3694 (7.4)	179 (6.5)
	Missing	3	3	0
Primary condition at index hospitalisation	Circulatory	15 234 (28.8)	14 417 (28.8)	817 (29.5)
	Neoplasms	10 182 (19.3)	9510 (19.0)	672 (24.3)
	Digestive	5773 (10.9)	5489 (11.0)	284 (10.3)
	Injury	4943 (9.4)	4670 (9.3)	273 (9.9)
	Respiratory	4673 (8.8)	4450 (8.9)	223 (8.1)
	Musculoskeletal	2126 (4.0)	2025 (4.0)	101 (3.6)
	Genitourinary	1797 (3.4)	1726 (3.4)	71 (2.6)
	Infectious	1567 (3.0)	1502 (3.0)	65 (2.3)
	Abnormal findings^b^	1298 (2.5)	1213 (2.4)	85 (3.1)
	Other	5253 (9.9)	5075 (10.1)	178 (6.4)
ICU type	Gen/Med/Surg	36 282 (71.2)	34 443 (71.3)	1839 (69.9)
	Cardio/Thoracic	10 348 (20.3)	9747 (20.2)	601 (22.8)
	Neuro	2282 (4.5)	2141 (4.4)	141 (5.4)
	Other	2043 (4.0)	1992 (4.1)	51 (1.9)
	Missing	1891	1754	137
Total organ systems supported	None	4300 (8.8)	4121 (8.8)	179 (7.0)
	1	15 033 (30.6)	14 323 (30.8)	710 (27.8)
	2	27 662 (56.3)	26 122 (56.1)	1540 (60.3)
	3	2130 (4.3)	2004 (4.3)	126 (4.9)
	Missing	3721	3507	214
Out‐of‐hours ICU discharge		7868 (15.4)	7435 (14.8)	433 (15.6)
	Missing	1891	1754 (3.5)	137 (4.9)
Hospital LOS	less than 7 days	21 110 (39.9)	20 201 (40.3)	909 (32.8)
	7–13 days	14 387 (27.2)	13 621 (27.2)	766 (27.7)
	14 days or more	17 349 (32.8)	16 255 (32.5)	1094 (39.5)

*Note: Rx*: prescription (here meaning benzodiazepine or z‐drug).

Abbreviations: IMD: index of multiple deprivation; ICU: intensive care unit; Gen/Med/Surg: General/Medical/Surgical; LOS: length of stay.

^a^ Modified by removing four mental health conditions (depression, psychoses, alcohol misuse and drug misuse) for individual analysis.

^b^ Symptoms, signs and abnormal clinical and laboratory findings, not elsewhere classified.

### 3.1 New Community Benzodiazepine and z‐Drug Prescription

There were 2769/52846 (5.2%; 95% CI 5.1–5.4) benzodiazepine and z‐drug‐naïve survivors prescribed a new benzodiazepine or z‐drug within 90 days of hospital discharge (median days to prescription 21 days (IQR 7–49)). Of all benzodiazepines and z‐drug prescriptions within 90 days, zopiclone was the most commonly prescribed drug (50%) followed by diazepam (19%), midazolam (9.8%), temazepam (9.6%), lorazepam (6.6%) and zolpidem (2.1%). New prescribing was more common in 2010 (6.4%, 95% CI 6.1–6.8) than in 2018 (4.4%, 95% CI 4.2–4.7). Furthermore, 1311/52846 (2.5%; 95% CI 2.4–2.6) of survivors received new‐and‐persistent prescriptions (median: 3 prescriptions, IQR 2–5 prescriptions). New‐and‐persistent prescribing was also more common in 2010 (3.2%, 95% CI 3.0–3.5) than in 2018 (2.0%, 95% CI 1.8–2.2).

For the complete case analyses of associations between new community benzodiazepine or z‐drug prescription and patient characteristics, 48 831 (92.4%) of the study population were included (Table S1). There was no association between sex and new prescription, and patients over 65 years were less likely to receive a new prescription than those younger than 65 years (Table 2). Compared to patients with white ethnicity, ethnic minorities had lower odds of new prescription. The two least deprived quintiles of socioeconomic status had a small but statistically significant higher odds of new prescription. There was no association between the modified Elixhauser comorbidity count and new prescription. Patients with a preadmission history of insomnia (adjusted OR 1.96; 95% CI 1.74–2.21), anxiety or depression (adjusted OR 1.40; 95% CI 1.28–1.53) and opioid prescription (adjusted OR 1.47; 95% CI 1.34–1.61) had higher odds of new community prescription after discharge. History of psychoses, alcohol misuse, drug misuse or prescription gabapentinoids were not associated with a new community benzodiazepine or z‐drug prescription. Patients with emergency hospitalisation had higher odds of new prescription (adjusted OR 1.39; 95% CI 1.25–1.55). Patients hospitalised for neoplasms had higher odds of new prescription compared to other index conditions (adjusted OR 1.47; 95% CI 1.29–1.67 compared to patients hospitalised for circulatory disease). Among ICU factors, care in a cardio/thoracic or neurologic ICU were associated with new prescription. Total organ systems supported and out‐of‐hours discharge from ICU were not associated with new prescription, but hospital length of stay greater than 7 days was associated with new prescription (adjusted OR 1.28; 95% CI 1.15–1.43 for 14+ days).

**TABLE 2:** Association of new community benzodiazepine or z‐drug prescription after hospital discharge in 52 846 adults surviving critical illness to hospital discharge.

Patient characteristics		Received Rx (%)	Univariable OR (95% CI)	Multivariable OR (95% CI) Complete cases *n* = 48 831
Sex	Male	1618 (5.3)	Reference	Reference
	Female	1151 (5.2)	0.97 (0.90–1.05)	0.92 (0.85–1.01)
Age group	18–49	533 (4.8)	Reference	Reference
	50–64	800 (5.7)	1.19 (1.06–1.33)	0.95 (0.84–1.08)
	65–79	1077 (5.3)	1.09 (0.98–1.22)	0.87 (0.76–0.99)
	80+	359 (4.8)	0.98 (0.86–1.13)	0.80 (0.67–0.94)
Ethnicity	Asian	121 (4.2)	0.76 (0.63–0.92)	0.88 (0.72–1.08)
	Black	54 (2.6)	0.48 (0.36–0.63)	0.54 (0.40–0.73)
	Mixed	11 (3.5)	0.64 (0.35–1.17)	0.68 (0.36–1.29)
	White	2553 (5.5)	Reference	Reference
	Other	22 (2.6)	0.47 (0.31–0.72)	0.59 (0.39–0.91)
IMD quintile	1, Least deprived	536 (5.4)	1.05 (0.92–1.19)	1.16 (1.01–1.32)
	2	596 (5.8)	1.12 (0.99–1.26)	1.21 (1.06–1.38)
	3	523 (5.2)	1.12 (0.99–1.26)	1.05 (0.92–1.20)
	4	523 (5.2)	0.95 (0.84–1.08)	1.02 (0.90–1.16)
	5, Most deprived	576 (5.1)	Reference	Reference
Modified Elixhauser comorbidity count^a^	None	308 (4.4)	Reference	Reference
	1	483 (4.9)	1.12 (0.97–1.30)	1.01 (0.86–1.18)
	2	559 (5.7)	1.33 (1.15–1.53)	1.13 (0.97–1.33)
	3	458 (5.4)	1.26 (1.08–1.46)	0.99 (0.84–1.18)
	4	365 (5.6)	1.29 (1.10–1.51)	1.03 (0.86–1.24)
	5 or more	596 (5.3)	1.23 (1.07–1.41)	0.92 (0.78–1.10)
History of insomnia	No	2344 (4.8)	Reference	Reference
	Yes	425 (10.4)	2.30 (2.06–2.56)	1.96 (1.74–2.21)
History of anxiety or depression	No	1633 (4.5)	Reference	Reference
	Yes	1136 (6.9)	1.58 (1.46–1.71)	1.40 (1.28–1.53)
History of psychoses	No	2689 (5.2)	Reference	Reference
	Yes	80 (7.4)	1.45 (1.14–1.81)	1.25 (0.97–1.60)
History of alcohol misuse	No	2349 (5.0)	Reference	Reference
	Yes	420 (6.7)	1.34 (1.21–1.49)	1.14 (1.01–1.29)
History of drug misuse	No	2672 (5.2)	Reference	Reference
	Yes	97 (7.7)	1.52 (1.23–1.87)	1.19 (0.94–1.50)
History of opioid prescription	No	1740 (4.5)	Reference	Reference
	Yes	1029 (7.2)	1.66 (1.53–1.80)	1.47 (1.34–1.61)
History of gabapentin/ pregabalin prescription	No	2559 (5.2)	Reference	Reference
	Yes	210 (6.5)	1.29 (1.11–1.48)	1.01 (0.86–1.19)
Number of hospital admissions (1 year)	None	796 (4.5)	Reference	Reference
	1	733 (5.1)	1.16 (1.05–1.29)	1.07 (0.96–1.20)
	2	512 (6.2)	1.42 (1.27–1.59)	1.22 (1.07–1.39)
	3+	728 (5.8)	1.33 (1.20–1.47)	1.10 (0.97–1.23)
Number of primary care consultations (1 year)	0–2	596 (4.5)	Reference	Reference
	3–7	550 (4.4)	0.97 (0.86–1.09)	1.06 (0.93–1.21)
	8–14	749 (5.3)	1.19 (1.06–1.33)	1.25 (1.10–1.41)
	15+	874 (6.8)	1.55 (1.39–1.74)	1.47 (1.29–1.67)
Year of admission	2010	1358 (6.4)	Reference	Reference
	2018	1411 (4.4)	0.68 (0.63–0.73)	0.63 (0.58–0.68)
Hospital admission method	Elective	1185 (5.1)	Reference	Reference
	Emergency	1405 (5.5)	1.09 (1.01–1.18)	1.39 (1.25–1.55)
	Transfer/Other	179 (4.6)	0.91 (0.77–1.07)	1.19 (0.99–1.43)
Primary condition at index hospitalisation	Circulatory	817 (5.4)	Reference	Reference
	Neoplasms	672 (6.6)	1.25 (1.12–1.39)	1.47 (1.29–1.67)
	Digestive	284 (4.9)	0.91 (0.79–1.05)	0.84 (0.71–0.98)
	Injury	273 (5.5)	1.04 (0.90–1.20)	0.96 (0.81–1.13)
	Respiratory	223 (4.8)	0.89 (0.76–1.03)	0.75 (0.63–0.89)
	Musculoskeletal	101 (4.8)	0.88 (0.71–1.09)	0.94 (0.73–1.20)
	Genitourinary	71 (4.0)	0.73 (0.57–0.94)	0.74 (0.56–0.97)
	Infectious	65 (4.1)	0.77 (0.59–0.99)	0.75 (0.56–0.99)
	Abnormal findings^b^	85 (6.5)	1.24 (0.98–1.56)	1.11 (0.86–1.43)
	Other	178 (3.4)	0.62 (0.53–0.74)	0.71 (0.59–0.86)
ICU type	Gen/Med/Surg	1839 (5.1)	Reference	Reference
	Cardio/Thoracic	601 (5.8)	1.15 (1.05–1.27)	1.29 (1.14–1.45)
	Neuro	141 (6.2)	1.23 (1.03–1.47)	1.32 (1.09–1.58)
	Other	51 (2.5)	0.48 (0.36–0.63)	0.68 (0.50–0.92)
Total organ systems supported	None	179 (4.2)	Reference	Reference
	1	710 (4.7)	1.14 (0.97–1.35)	1.05 (0.88–1.25)
	2	1540 (5.6)	1.36 (1.16–1.59)	1.18 (1.00–1.39)
	3	126 (5.9)	1.45 (1.14–1.83)	1.23 (0.96–1.57)
Out‐of‐hours ICU discharge	No	2199 (5.1)	Reference	Reference
	Yes	433 (5.5)	1.08 (0.97–1.20)	1.08 (0.96–1.20)
Hospital LOS	< 7 days	909 (4.3)	Reference	Reference
	7–13 days	766 (5.3)	1.25 (1.13–1.38)	1.14 (1.02–1.26)
	14 days or more	1094 (6.3)	1.50 (1.37–1.64)	1.28 (1.15–1.43)

*Note:* Multivariable models were adjusted for all covariates shown. *Rx*: prescription (here meaning benzodiazepine or z‐drug).

Abbreviations: Gen/Med/Surg: General/Medical/Surgical; ICU: intensive care unit; IMD: index of multiple deprivation; LOS: length of stay.

^a^ Modified by removing four mental health conditions (depression, psychoses, alcohol misuse and drug misuse) for individual analysis.

^b^ Symptoms, signs and abnormal clinical and laboratory findings, not elsewhere classified.

### 3.2 Between‐Practice Variation

Patients were registered with 1398 primary care practices, with a median of 32 patients per practice (IQR 20–49; range 1–387). There was variation between practices in the proportion of survivors prescribed new benzodiazepine or z‐drugs (median: 4.8%, IQR 0–7.5) and new‐and‐persistent prescribing (median: 1.5%, IQR 0–4.0). The intraclass correlation (ICC) was 2.9% before adjustment for patient characteristics, compared to 4.0% for new‐and‐persistent prescribing. After adjustment for patient characteristics, the ICC for new prescribing was 2.6% (i.e., 2.6% of the variation in new prescribing to patients was attributable to variation between practices even after accounting for patient characteristics) and 4.1% for new‐and‐persistent prescribing.

### 3.3 Sensitivity Analyses

Excluding 14 104 (26.7%) patients who died or were readmitted to hospital within 30 days of index discharge, demonstrated similar findings to the primary analysis (Table S2). Excluding 22 160 (41.9%) patients who died or were readmitted to hospital within 90 days of index discharge, demonstrated similar findings to the primary analysis (Table S3). Finally, by changing the definition of treatment‐naïve patients from those not receiving a benzodiazepine or z‐drug prescription within 180 days prior to index hospital admission, to 365 days, we excluded 1254 (2.4%) patients and found very similar results (Table S4).

## 4 Discussion

Our study found that 5.2% of critical illness survivors naïve to benzodiazepines or z‐drugs prior to hospitalisation received a community prescription for a benzodiazepine or z‐drug within 90 days of hospital discharge, with half of the prescriptions for zopiclone. Among those prescribed, 47% received two or more prescriptions within 180 days of index discharge (2.5% of all patients). Comparing 2018 to 2010, new prescribing decreased from 6.4% to 4.4% and new‐and‐persistent prescribing decreased from 3.2% to 2.0%. Patients with a history of insomnia, anxiety or depression, recent opioid prescription, and emergency hospital admission had higher odds for benzodiazepine or z‐drug prescription after hospitalisation compared to those without. Sex was not associated with new prescribing, but patients aged over 65 years were less likely to receive a new prescription than younger patients.

Our finding that one in 20 benzodiazepine and z‐drug‐naïve critical illness survivors in England are prescribed a benzodiazepine or z‐drug within 90 days of hospital discharge is similar to our findings in the Lothian region of Scotland [34]. This earlier study found that 6.5% of critical care survivors aged 18 years or older were prescribed a new hypnotic or anxiolytic within 90 days of hospital discharge, the majority of which were benzodiazepines or z‐drugs, but the data were not linkable to primary care data preventing detailed analysis of patient characteristics. A recent population‐based study from Ontario, Canada, limited to critical care survivors over 65 years of age (mean age 76) found that 3.5% were dispensed a new prescription for a benzodiazepine and 1.1% for a nonbenzodiazepine sedative within seven days of hospital discharge, the majority of which were prescribed by the discharging hospital [26]. Similar to our findings, they also found that half of those prescribed received two or more prescriptions within 180 days of index discharge. Of note, in Ontario, z‐drugs are only covered by the drug benefit programme if specific clinical circumstances are met, and were rarely prescribed, with the most commonly prescribed drug in their study being a benzodiazepine much less commonly used in the UK (lorazepam). The same group have also recently described trends in sedative prescription after a critical care episode in their treatment‐naïve older patient population cohort in Ontario [35]. Similar to our findings, the percentage of patients having a prescription for benzodiazepines decreased over time, demonstrating an approximate 10% annual decrease, from 6.8% in 2003 to 1.1% in 2019.

The reduction in new prescribing of benzodiazepine/z‐drugs in our study population between 2010 and 2018 is likely related to changes in patient management due to ICU practice guidelines published between the cohort years. In 2013, clinical practice guidelines for management of adults in the ICU recommended minimising benzodiazepine use in lieu of sedation with dexmedetomidine or propofol (e.g., for mechanical ventilation or other invasive procedures) [36]. In 2015, the UK Guidelines for the Provision of Intensive Care Services [37] incorporated the standard of a designated intensive care pharmacist for every critical care unit and recommendations to perform medicine reconciliation. Implementation of changes to ICU clinical practice, such as bundles of care, have been demonstrated to improve patient outcomes including mortality, durations of coma, delirium, mechanical ventilation and length of ICU stay [38]. In turn, these improvements in ICU patient care are associated with reduced mental health disorders in patients post‐ICU and hence need for further pharmacologic management in recovery [1]. Medication reconciliation and review processes on transition from ICU also now reduce the risk of potentially inappropriate sedatives continuing without an ongoing indication [22]. In primary care, there have also been changes in practice to use alternative anxiolytic treatments including more sedating antidepressants and psychological therapies [14, 35, 39, 40].

It is perhaps unsurprising that patients with a history of insomnia, anxiety or depression have a higher incidence of benzodiazepine or z‐drug prescribing as this is the primary indication of the drug class and not likely to improve after a critical illness. However, patients with a recent history of prescription opioid use were also more likely to be prescribed a benzodiazepine or z‐drug after hospital discharge. Other studies have reported an association between opioid and benzodiazepine use in older primary care and surgical patient groups [41, 42]. Of concern is that the combination of opioid and benzodiazepine has been associated with increased risk of hospitalisation and death [41, 43, 44, 45, 46, 47].

We found variation of prescribing among primary care practices which is similar in size to other between‐practice variations in care, although this variation may potentially be a sequela of recommendations by the discharging hospital. There are over 200 hospitals with ICUs throughout England; however, our data sources lacked a hospital identifier preventing us from exploring whether between‐hospital variation might explain some of the between‐practice variation. Of note, the higher variation between practices observed in patients receiving more than one benzodiazepine or z‐drug prescription after hospital discharge is a small, but tangible area for improvement considering the high potential of physiologic dependency with regular use [48].

Our study has several strengths. The CPRD Aurum database, our primary data source, includes routinely collected data and prescriptions from primary care practices making it fit‐for‐purpose for pharmacoepidemiologic studies. HES APC includes all admissions to NHS England hospitals and includes data on critical care stays, and ONS is based on national mortality registration which is legally required after a death. Our ability to include patients under 65 years of age without restriction allows for greatest generalisability to all survivors of critical illness and is uncommon when compared to published studies. We also used analytical methods to account for prescribing practice and quantify variation practices.

Our study has a number of limitations. A major limitation is the lack of in‐hospital prescribing data [49]. Patients receiving benzodiazepines or z‐drugs from the discharging hospital would be misclassified as not receiving the medication if there was no subsequent prescription from their primary care practice, potentially underestimating the incidence of early prescription. However, past studies on hospitalised patients (not limited to critical care, and mostly limited to patients over 65 years of age) have shown that patients starting benzodiazepines and z‐drugs in the hospital are frequently prescribed these medicines again shortly after hospital discharge [23, 24, 25, 50, 51]. Another limitation is that we did not have prescription label information or the intended duration of the prescription from these data sources. Treatment duration is difficult to infer as benzodiazepines and z‐drugs are often recommended ‘as required’. Almost half of patients with new prescriptions did not have a postdischarge diagnosis of anxiety, depression or insomnia recorded (a common feature of studies using electronic health record data), which makes it hard to explore the appropriateness of prescribing.

Our study findings are the first to examine benzodiazepine and z‐drug community prescribing after critical illness in a national UK cohort. These medications come with inherent risks, still unquantified in critical illness survivors. ICU teams need to effectively implement evidence‐based practices that improve sedative medication use and review [52, 53]. System changes to improve the continuity of care and medicines optimisation in patients recovering from critical illness are also needed [22, 54]. It is essential that discharge summaries include duration and medication tapering/deprescribing plans for patients leaving the hospital with prescriptions as primary care practitioners may be reluctant to stop medicines initiated in the hospital [25]. Additionally, primary care or post‐ICU recovery services should include early medicine review post‐patient discharge, and consider interventions other than pharmacotherapy, including psychological follow‐up and assessments and psychological therapies for anxiety and sleep disturbances [40]. Finally, prescribing practice variation not explained by the measured variables may represent a modifiable target for practice improvement.

Prospective studies or availability of in‐hospital prescribing data are needed to address whether community prescriptions are the result of recommendations from the discharging hospital, inappropriate continuation, or initiation postdischarge. For those patients where benzodiazepines or z‐drugs are initiated in the hospital, identifying the point in the care pathway (e.g., ICU, ward or at hospital discharge) may help determine the appropriateness of community prescribing after discharge. Further research is then needed to understand how continuity of patient care after a critical illness is effectively coordinated and managed across the care interfaces and how these impact on medication use, patient outcomes and care satisfaction. Future research should account for variation in benzodiazepine and z‐drug prescribing. Additionally, safety studies of these medicines after recent critical illness are imperative in these vulnerable patients. Research such as this could improve clinical practice and help guide further clinical policy to improve patient outcomes after critical illness.

### 4.1 Conclusion

Our study found that one in 20 adult survivors of critical illness received a new community benzodiazepine or z‐drug prescription within 90 days of hospital discharge, with almost half of those receiving additional prescriptions. Further research is required to understand the source and indications for benzodiazepine and z‐drugs in survivors of critical illness and the impact of exposure on adverse drug effects. These will inform future priorities and focus of medicines optimisation and treatment interventions in these vulnerable patients.

### 4.2 Plain Language Summary

Over half of critical care survivors have anxiety and sleep disturbances within the first few months after hospital discharge. Our study assessed adult patients in the UK who were not prescribed medications for anxiety or insomnia before they were hospitalised for critical care (such as benzodiazepines, like diazepam (Valium), or z‐drugs like zolpidem (Ambien)). We then evaluated the occurrence of new and continued prescriptions, as well as factors associated with prescribing after critical care hospitalisation. We considered patients' sex, age, ethnicity and socioeconomic status. We also factored their medical history, main condition for hospitalisation, as well as specific information relating to their critical illness. Among over 50 000 survivors that we studied, we found that one in 20 were prescribed a new benzodiazepine or z‐drug by their primary care provider within 90 days of hospital discharge, and almost half of these were prescribed more than once. Patients with a history of insomnia, anxiety or depression, and recent opioid prescription were most likely to receive a new prescription. Sex was not associated with new prescription and older patients were less likely to receive a prescription. Whether prescribed anxiety medications or sleeping pills are safe in critical care survivors requires further research.

## Conflicts of Interest

The authors declare no conflicts of interest.

## Supporting information


Appendix S1.

Tables S1‐S4.
