# Supplementary material for: Patient Characteristics and Practice Variation Associated With New Community Prescription of Benzodiazepine and z‐Drug Hypnotics After Critical Illness: A Retrospective Cohort Study Using the UK Clinical Practice Research Datalink
Source: Pharmacoepidemiol Drug Saf. 2024 Nov 27;33(12):e70056. doi: 10.1002/pds.70056 (PMC11602247; doi:10.1002/pds.70056)
Supplement: Supplementary file 1 — Appendix S1. [file PDS-33-e70056-s001.docx]

**Appendix**

Primary care Read code lists (v2) for categories assessed.

**The codelists below were obtained from the Health Data Research United Kingdom (HDR UK) Phenotype Library**^1^

Table of Contents

[Alcohol abuse (Elixhauser) 3](#_Toc163588851)

[Anxiety 5](#_Toc163588852)

[Blood loss anaemia (Elixhauser) 7](#_Toc163588853)

[Cardiac arrhythmia (Elixhauser) 8](#_Toc163588854)

[Chronic pulmonary disease (Elixhauser) 13](#_Toc163588855)

[Coagulopathy (Elixhauser) 25](#_Toc163588856)

[Congestive heart failure (Elixhauser) 27](#_Toc163588857)

[Deficiency anaemia (Elixhauser) 30](#_Toc163588858)

[Depression (Elixhauser) 31](#_Toc163588859)

[Diabetes, uncomplicated (Elixhauser) 34](#_Toc163588860)

[Diabetes, with end-organ damage (Elixhauser) 43](#_Toc163588861)

[Drug abuse (Elixhauser) 49](#_Toc163588862)

[Fluid and electrolyte disorders (Elixhauser) 55](#_Toc163588863)

[HIV AIDS (Elixhauser) 56](#_Toc163588864)

[Hypertension, uncomplicated (Elixhauser) 58](#_Toc163588865)

[Hypertension with end-organ damage (Elixhauser) 59](#_Toc163588866)

[Hypothyroidism (Elixhauser) 60](#_Toc163588867)

[Insomnia 61](#_Toc163588868)

[Liver disease (Elixhauser) 62](#_Toc163588869)

[Lymphoma (Elixhauser) 67](#_Toc163588870)

[Metastatic cancer (Elixhauser) 74](#_Toc163588871)

[Obesity (Elixhauser) 78](#_Toc163588872)

[Other neurological disorders (Elixhauser) 79](#_Toc163588873)

[Paralysis (Elixhauser) 93](#_Toc163588874)

[Peptic ulcer disease (Elixhauser) 96](#_Toc163588875)

[Peripheral vascular disease (Elixhauser) 98](#_Toc163588876)

[Psychoses (Elixhauser) 107](#_Toc163588877)

[Pulmonary circulation disorders (Elixhauser) 114](#_Toc163588878)

[Renal disease (Elixhauser) 115](#_Toc163588879)

[Rhematoid arthritis and collagen diseases (Elixhauser) 124](#_Toc163588880)

[Solid tumour or leukaemia (Elixhauser) 128](#_Toc163588881)

[Valvular disease (Elixhauser) 164](#_Toc163588882)

[Weight loss (Elixhauser) 171](#_Toc163588883)

1. HDR UK. HDR UK Phenotype Library. Accessed March 8, 2024. http://phenotypes.healthdatagateway.org/

# Alcohol abuse (Elixhauser)

| **Read code** | **Description** |
| --- | --- |
| **E250300** | Nondependent alcohol abuse in remission |
| **E010.12** | Delirium tremens |
| **J671000** | Alcohol-induced chronic pancreatitis |
| **ZV6D600** | [V]Alcohol abuse counselling and surveillance |
| **E230200** | Episodic acute alcoholic intoxication in alcoholism |
| **Eu10611** | [X]Korsakov's psychosis, alcohol induced |
| **E231000** | Unspecified chronic alcoholism |
| **C150500** | Alcohol-induced pseudo-Cushing's syndrome |
| **E230.11** | Alcohol dependence with acute alcoholic intoxication |
| **E250z00** | Nondependent alcohol abuse NOS |
| **Eu10011** | [X]Acute alcoholic drunkenness |
| **SLH3.00** | Alcohol deterrent poisoning |
| **8H7p.00** | Referral to community alcohol team |
| **E23..00** | Alcohol dependence syndrome |
| **136S.00** | Hazardous alcohol use |
| **Eu10100** | [X]Mental and behav dis due to use of alcohol: harmful use |
| **E011.00** | Alcohol amnestic syndrome |
| **U60H311** | [X] Adverse reaction to alcohol deterrents |
| **L255300** | Maternal care for (suspected) damage to fetus from alcohol |
| **8IAt.00** | Extended interven for excessive alcohol consumption declined |
| **E011z00** | Alcohol amnestic syndrome NOS |
| **E015.00** | Alcoholic paranoia |
| **1B1c.00** | Alcohol induced hallucinations |
| **E230300** | Acute alcoholic intoxication in remission, in alcoholism |
| **J613000** | Alcoholic hepatic failure |
| **Eu10513** | [X]Alcoholic paranoia |
| **E230100** | Continuous acute alcoholic intoxication in alcoholism |
| **8IAJ.00** | Declined referral to specialist alcohol treatment service |
| **J617000** | Chronic alcoholic hepatitis |
| **Eu10212** | [X]Chronic alcoholism |
| **8IAF.00** | Brief intervention for excessive alcohol consumptn declined |
| **E231z00** | Chronic alcoholism NOS |
| **Eu10y00** | [X]Men & behav dis due to use alcohol: oth men & behav dis |
| **Eu10500** | [X]Mental & behav dis due to use alcohol: psychotic disorder |
| **Z191211** | Alcohol reduction programme |
| **J611.00** | Acute alcoholic hepatitis |
| **1366.00** | Very heavy drinker - >9u/day |
| **Eu10400** | [X]Men & behav dis due alcohl: withdrawl state with delirium |
| **E231300** | Chronic alcoholism in remission |
| **E231100** | Continuous chronic alcoholism |
| **8H35.00** | Admitted to alcohol detoxification centre |
| **8HHe.00** | Referral to community drug and alcohol team |
| **U60H300** | [X]Alcohol deterrents caus adverse effects in therapeut use |
| **Z191100** | Alcohol withdrawal regime |
| **J610.00** | Alcoholic fatty liver |
| **E230.00** | Acute alcoholic intoxication in alcoholism |
| **E01yz00** | Other alcoholic psychosis NOS |
| **E014.00** | Pathological alcohol intoxication |
| **G555.00** | Alcoholic cardiomyopathy |
| **Eu10600** | [X]Mental and behav dis due to use alcohol: amnesic syndrome |
| **E012000** | Chronic alcoholic brain syndrome |
| **136T.00** | Harmful alcohol use |
| **Eu10411** | [X]Delirium tremens, alcohol induced |
| **Eu10512** | [X]Alcoholic jealousy |
| **E013.00** | Alcohol withdrawal hallucinosis |
| **F11x000** | Cerebral degeneration due to alcoholism |
| **8CAv.00** | Advised to contact primary care alcohol worker |
| **E011200** | Wernicke-Korsakov syndrome |
| **Eu10200** | [X]Mental and behav dis due to use alcohol: dependence syndr |
| **SM00100** | Denatured alcohol causing toxic effect |
| **E230000** | Acute alcoholic intoxication, unspecified, in alcoholism |
| **E012.11** | Alcoholic dementia NOS |
| **E230z00** | Acute alcoholic intoxication in alcoholism NOS |
| **66e..00** | Alcohol disorder monitoring |
| **9NN2.00** | Under care of community alcohol team |
| **ZV57A00** | [V]Alcohol rehabilitation |
| **Z191.00** | Alcohol detoxification |
| **E010.00** | Alcohol withdrawal delirium |
| **13Y8.00** | Alcoholics anonymous |
| **E01..00** | Alcoholic psychoses |
| **E250.00** | Nondependent alcohol abuse |
| **E250000** | Nondependent alcohol abuse, unspecified |
| **Z4B1.00** | Alcoholism counselling |
| **E01z.00** | Alcoholic psychosis NOS |
| **8BA8.00** | Alcohol detoxification |
| **9k1B.00** | Extended intervention for excessive alcohol consumptn complt |
| **Eu10700** | [X]Men & behav dis due alcoh: resid & late-onset psychot dis |
| **E23..11** | Alcoholism |
| **F25B.00** | Alcohol-induced epilepsy |
| **F375.00** | Alcoholic polyneuropathy |
| **E23..12** | Alcohol problem drinking |
| **136W.00** | Alcohol misuse |
| **E250200** | Nondependent alcohol abuse, episodic |
| **J613.00** | Alcoholic liver damage unspecified |
| **Eu10.00** | [X]Mental and behavioural disorders due to use of alcohol |
| **7P22100** | Delivery of rehabilitation for alcohol addiction |
| **E040.11** | Korsakoff's non-alcoholic psychosis |
| **J612000** | Alcoholic fibrosis and sclerosis of liver |
| **8HkJ.00** | Referral to alcohol brief intervention service |
| **J153.00** | Alcoholic gastritis |
| **Eu10213** | [X]Dipsomania |
| **E250100** | Nondependent alcohol abuse, continuous |
| **66e0.00** | Alcohol abuse monitoring |
| **9k14.00** | Alcohol counselling by other agencies |
| **1462.00** | H/O: alcoholism |
| **Eu10511** | [X]Alcoholic hallucinosis |
| **E23z.00** | Alcohol dependence syndrome NOS |
| **F394100** | Alcoholic myopathy |
| **8HkG.00** | Referral to specialist alcohol treatment service |
| **Eu10711** | [X]Alcoholic dementia NOS |
| **E011000** | Korsakov's alcoholic psychosis |
| **Eu10300** | [X]Mental and behav dis due to use alcohol: withdrawal state |
| **F11x011** | Alcoholic encephalopathy |
| **ZV11300** | [V]Personal history of alcoholism |
| **E01y000** | Alcohol withdrawal syndrome |
| **E231.11** | Dipsomania |
| **Eu10800** | [X]Alcohol withdrawal-induced seizure |
| **Eu10211** | [X]Alcohol addiction |
| **Eu10712** | [X]Chronic alcoholic brain syndrome |
| **136Q.00** | Very heavy drinker |
| **Eu10z00** | [X]Ment & behav dis due use alcohol: unsp ment & behav dis |
| **E231200** | Episodic chronic alcoholism |
| **E231.00** | Chronic alcoholism |
| **8G32.00** | Aversion therapy - alcoholism |
| **J612.00** | Alcoholic cirrhosis of liver |

# Anxiety

| **Read code** | **Description** |
| --- | --- |
| **Z481.00** | Phobia counselling |
| **Eu40300** | [X]Needle phobia |
| **E200500** | Recurrent anxiety |
| **Eu40212** | [X]Animal phobias |
| **Eu34113** | [X]Neurotic depression |
| **E200400** | Chronic anxiety |
| **225K.00** | O/E - fearful mood |
| **E202100** | Agoraphobia with panic attacks |
| **E202z00** | Phobic disorder NOS |
| **Eu41y11** | [X]Anxiety hysteria |
| **E202D00** | Fear of death |
| **Eu41111** | [X]Anxiety neurosis |
| **E200.00** | Anxiety states |
| **Eu40214** | [X]Simple phobia |
| **Eu34114** | [X]Persistant anxiety depression |
| **E202700** | Animal phobia |
| **Eu40y00** | [X]Other phobic anxiety disorders |
| **Eu40011** | [X]Agoraphobia without history of panic disorder |
| **E202400** | Social phobia, fear of public speaking |
| **E202000** | Phobia unspecified |
| **Eu41000** | [X]Panic disorder [episodic paroxysmal anxiety] |
| **E20y200** | Other occupational neurosis |
| **Eu40000** | [X]Agoraphobia |
| **1466.00** | H/O: anxiety state |
| **Eu40211** | [X]Acrophobia |
| **Eu41300** | [X]Other mixed anxiety disorders |
| **8G52.00** | Antiphobic therapy |
| **E202900** | Fear of crowds |
| **Eu41211** | [X]Mild anxiety depression |
| **E202500** | Social phobia, fear of public washing |
| **9N54.00** | Encounter for fear |
| **Eu41y00** | [X]Other specified anxiety disorders |
| **Eu41112** | [X]Anxiety reaction |
| **8HHp.00** | Referral for guided self-help for anxiety |
| **Eu41200** | [X]Mixed anxiety and depressive disorder |
| **E202800** | Claustrophobia |
| **Eu41.00** | [X]Other anxiety disorders |
| **E202300** | Social phobia, fear of eating in public |
| **Eu40012** | [X]Panic disorder with agoraphobia |
| **Eu41113** | [X]Anxiety state |
| **E200200** | Generalised anxiety disorder |
| **Eu41012** | [X]Panic state |
| **E200z00** | Anxiety state NOS |
| **E20yz00** | Other neurotic disorder NOS |
| **Eu41011** | [X]Panic attack |
| **E202B00** | Cancer phobia |
| **E200111** | Panic attack |
| **225J.00** | O/E - panic attack |
| **E20z.00** | Neurotic disorder NOS |
| **Eu41z11** | [X]Anxiety NOS |
| **Eu40z12** | [X]Phobic state NOS |
| **E202200** | Agoraphobia without mention of panic attacks |
| **Eu40z11** | [X]Phobia NOS |
| **E202.00** | Phobic disorders |
| **146G.00** | H/O: agoraphobia |
| **ZV11200** | [V]Personal history of neurosis |
| **Eu40213** | [X]Claustrophobia |
| **E200000** | Anxiety state unspecified |
| **E202.11** | Social phobic disorders |
| **Eu34111** | [X]Depressive neurosis |
| **E20..00** | Neurotic disorders |
| **1B1H.11** | Fear |
| **Eu40200** | [X]Specific (isolated) phobias |
| **Eu40100** | [X]Social phobias |
| **E20y.00** | Other neurotic disorders |
| **Eu40.00** | [X]Phobic anxiety disorders |
| **E201B00** | Compensation neurosis |
| **E202600** | Acrophobia |
| **E202E00** | Fear of pregnancy |
| **E200300** | Anxiety with depression |
| **286..00** | Poor insight into neurotic condition |
| **Eu41100** | [X]Generalized anxiety disorder |
| **E20y100** | Writer's cramp neurosis |
| **E202A00** | Fear of flying |
| **Z4L1.00** | Anxiety counselling |
| **E202.12** | Phobic anxiety |
| **E20y300** | Psychasthenic neurosis |
| **285..00** | Neurotic condition, insight present |
| **1Bb..00** | Specific fear |
| **1B1V.00** | C/O - panic attack |
| **Eu40112** | [X]Social neurosis |
| **E202C00** | Dental phobia |
| **E200100** | Panic disorder |
| **Eu41z00** | [X]Anxiety disorder, unspecified |
| **Eu40z00** | [X]Phobic anxiety disorder, unspecified |
| **8G94.00** | Anxiety management training |

# Blood loss anaemia (Elixhauser)

| **Read code** | **Description** |
| --- | --- |
| **D000.00** | Iron deficiency anaemia due to chronic blood loss |
| **D211.11** | Normocytic anaemia following acute bleed |
| **D000.11** | Normocytic anaemia due to chronic blood loss |
| **D000.12** | Iron deficiency anaemia due to blood loss |

# Cardiac arrhythmia (Elixhauser)

| **Read code** | **Description** |
| --- | --- |
| **9Os1.00** | Atrial fibrillation monitoring second letter |
| **7934000** | Percutaneous transluminal ablation of atrioventricular node |
| **G567200** | Pre-excitation atrioventricular conduction |
| **G57yA00** | Re-entry ventricular arrhythmia |
| **7936B00** | Implantation simple one wire intravenous cardiac pacemaker |
| **7936500** | Implantation of emergency intravenous cardiac pacemaker |
| **G571.11** | Ventricular tachycardia |
| **8OAD.00** | Provision of written information about atrial fibrillation |
| **7936z00** | Cardiac pacemaker system introduced via vein NOS |
| **7936F00** | Renewal of intravenous cardiac pacemaker system |
| **G574000** | Ventricular fibrillation |
| **3298.00** | ECG: complete A-V block |
| **G565000** | Bundle branch block unspecified |
| **G573500** | Persistent atrial fibrillation |
| **G572100** | Bouveret-Hoffmann syndrome |
| **32I2.00** | ECG: P-R interval abnormal |
| **G57y500** | Wandering atrial pacemaker |
| **9Os4.00** | Atrial fibrillation monitoring telephone invite |
| **G565z00** | Other bundle branch block NOS |
| **G573400** | Permanent atrial fibrillation |
| **G57..11** | Cardiac arrhythmias |
| **G576200** | Ventricular ectopic beats |
| **G570000** | Paroxysmal atrial tachycardia |
| **3299.00** | ECG: right bundle branch block |
| **G562.00** | Left bundle branch hemiblock |
| **3294.00** | ECG:partial A-V block-long P-R |
| **G574100** | Ventricular flutter |
| **G56zz00** | Conduction disorders NOS |
| **G567.00** | Anomalous atrioventricular excitation |
| **ZV45M00** | [V]Biventricular pacemaker in situ |
| **G561z00** | Atrioventricular block NOS |
| **7937800** | Implantation of dual chamber cardiac pacemaker system |
| **7936A00** | Implant intravenous pacemaker for atrial fibrillation |
| **G57..00** | Cardiac dysrhythmias |
| **329G.00** | ECG: right bundle branch and left posterior fascicular block |
| **7936200** | Maintenance of battery of intravenous cardiac pacemaker syst |
| **7937z00** | Other cardiac pacemaker system NOS |
| **7936511** | Implantation of temporary intravenous cardiac pacemaker |
| **G574z00** | Ventricular fibrillation and flutter NOS |
| **G570300** | Paroxysmal nodal tachycardia |
| **7937.00** | Other cardiac pacemaker system |
| **8HTy.00** | Referral to atrial fibrillation clinic |
| **7P19z00** | Pacemaker testing NOS |
| **G56..11** | Conduction disorders of heart |
| **3273.00** | ECG: atrial flutter |
| **7936K00** | Implantation of intravenous cardiac pacemaker system NEC |
| **G566200** | Right fascicular block |
| **G572z00** | Paroxysmal tachycardia NOS |
| **G560.00** | Complete atrioventricular block |
| **G55A.11** | Tachycardia-induced cardiomyopathy |
| **2JS..00** | Patient with internal cardiac defibrillator pacemaker |
| **7937700** | Implantation of single chamber cardiac pacemaker system |
| **329D.00** | ECG: left anterior fascicular block |
| **328..00** | ECG: ventricular arrhythmia |
| **3297.11** | Electrocardiogram: Mobitz type 1 second degree AV block |
| **Gyu5V00** | [X]Other and unspecified fascicular block |
| **G559.00** | Arrhythmogenic right ventricular cardiomyopathy |
| **14AD.00** | H/O ventricular fibrillation |
| **G573100** | Atrial flutter |
| **G574.00** | Ventricular fibrillation and flutter |
| **7937y00** | Other specified other cardiac pacemaker system |
| **14AN.00** | H/O: atrial fibrillation |
| **G573000** | Atrial fibrillation |
| **3283.00** | ECG: ventricular fibrillation |
| **G566100** | Interventricular block NOS |
| **G565200** | Right BBB with left anterior fascicular block |
| **G567z00** | Anomalous atrioventricular excitation NOS |
| **3272.00** | ECG: atrial fibrillation |
| **G566.00** | Other heart block |
| **G561400** | Second degree atrioventricular block |
| **7P19.00** | Pacemaker testing |
| **7P19000** | Distant pacemaker test |
| **G562100** | Left posterior fascicular block |
| **32J2.00** | ECG: QRS complex abnormal |
| **G56y100** | Atrioventricular dissociation |
| **G561200** | Mobitz type II atrioventricular block |
| **G566000** | Sinoatrial block |
| **G56y.00** | Other conduction disorders |
| **G55A.00** | Tachycardiomyopathy |
| **G573z00** | Atrial fibrillation and flutter NOS |
| **329A.00** | ECG: left bundle branch block |
| **G561000** | Atrioventricular block unspecified |
| **Gyu5a00** | [X]Other specified cardiac arrhythmias |
| **14AQ.00** | History of supraventricular tachycardia |
| **G573200** | Paroxysmal atrial fibrillation |
| **3292.00** | ECG: partial sinu-atrial block |
| **G564.00** | Right bundle branch block |
| **G576300** | Atrial premature depolarization |
| **7936300** | Maintenance of intravenous cardiac pacemaker system NEC |
| **7937300** | Maintenance of cardiac pacemaker system NEC |
| **P6y5.00** | Congenital heart block |
| **G565500** | Bifascicular block |
| **329..00** | ECG: heart block |
| **Gyu5X00** | [X]Other specified heart block |
| **G576400** | Junctional premature depolarization |
| **7936C00** | Implantation of complex 1 wire intravenous cardiac pacemaker |
| **G57y900** | Supraventricular tachycardia NOS |
| **G567400** | Wolff-Parkinson-White syndrome |
| **9hF1.00** | Excepted from atrial fibrillation qual indic: Inform dissent |
| **Gyu5U00** | [X]Other and unspecified atrioventricular block |
| **32K2.00** | ECG: Q-T interval abnormal |
| **7937900** | Implantation of biventricular cardiac pacemaker system |
| **G56..12** | Heart block |
| **14V1.00** | H/O: cardiac pacemaker in situ |
| **329B.00** | ECG: trifascicular block |
| **3293.00** | ECG:complete sinu-atrial block |
| **7936900** | Implantation of intravenous atrial overdrive pacemaker |
| **P6y5200** | Congenital incomplete atrio-ventricular heart block |
| **P6y5000** | Congenital heart block, unspecified |
| **G57y400** | Sinoatrial node dysfunction NOS |
| **G573600** | Paroxysmal atrial flutter |
| **7936y00** | Other specified cardiac pacemaker system introduced via vein |
| **7937200** | Maintenance of battery of cardiac pacemaker system NEC |
| **7930000** | Open ablation of atrioventricular node |
| **7936800** | Implantation of intravenous triggered cardiac pacemaker |
| **ZV53013** | [V]Fitting or adjustment of neuropacemaker device |
| **G576500** | Ventricular premature depolarization |
| **G57y.00** | Other cardiac dysrhythmias |
| **14AP.00** | History of ventricular tachycardia |
| **7936.11** | Introduction of intravenous cardiac pacemaker system |
| **G571.00** | Paroxysmal ventricular tachycardia |
| **6A9..00** | Atrial fibrillation annual review |
| **G57yz00** | Other cardiac dysrhythmia NOS |
| **3297.00** | ECG: Wenckebach phenomenon |
| **32K3.00** | ECG: Q-T interval prolonged |
| **8HRF.00** | Referral for cardiac pacemaker check |
| **G573300** | Non-rheumatic atrial fibrillation |
| **7936J00** | Implantat intravenous biventricular cardiac pacemaker system |
| **G567100** | Accessory atrioventricular conduction |
| **7936100** | Resiting of lead of intravenous pacemaker system |
| **SP00100** | Mechanical complication of cardiac pacemaker |
| **G56y600** | Pacemaker twiddler's syndrome |
| **G567300** | Ventricular pre-excitation |
| **329C.00** | ECG: bifascicular block |
| **329H.00** | Electrocardiogram: Mobitz type 2 second degree AV block |
| **9Os..00** | Atrial fibrillation monitoring administration |
| **G570200** | Paroxysmal junctional tachycardia |
| **G573.00** | Atrial fibrillation and flutter |
| **G561311** | Mobitz type 1 second degree atrioventricular block |
| **G566z00** | Other heart block NOS |
| **3295.00** | ECG: partial A-V block - 2:1 |
| **7936G00** | Implantat intraven single chamber cardiac pacemaker system |
| **32I3.00** | ECG: P-R interval prolonged |
| **G572.00** | Paroxysmal tachycardia unspecified |
| **793M100** | Perc transluminal ablation of atrial wall for atrial flutter |
| **793M300** | Perc translum ablat conduct sys heart for atrial flutter NEC |
| **9hF..00** | Exception reporting: atrial fibrillation quality indicators |
| **G56..00** | Conduction disorders |
| **G561100** | First degree atrioventricular block |
| **G565400** | Trifascicular block |
| **G56yz00** | Other conduction disorders NOS |
| **327Z.00** | ECG: supraventric. arryth. NOS |
| **3296.00** | ECG: partial A-V block - 3:1 |
| **G56y000** | Lown-Ganong-Levine syndrome |
| **G561.00** | Partial atrioventricular block |
| **9Os0.00** | Atrial fibrillation monitoring first letter |
| **7936700** | Implantation of intravenous fixed-rate cardiac pacemaker |
| **G560.11** | Third degree atrioventricular block |
| **G576.11** | Premature beats |
| **G570.00** | Paroxysmal supraventricular tachycardia |
| **329F.00** | ECG: right bundle branch and left anterior fascicular block |
| **G565100** | Right BBB with left posterior fascicular block |
| **7936D00** | Implantation complex two wire intravenous cardiac pacemaker |
| **G565.00** | Other bundle branch block |
| **G567000** | Accelerated atrioventricular conduction |
| **212R.00** | Atrial fibrillation resolved |
| **G562.11** | Left bundle branch block |
| **P6y5100** | Congenital complete atrio-ventricular heart block |
| **327..00** | ECG: supraventricular arrhythmia |
| **3282.00** | ECG: ventricular tachycardia |
| **7936400** | Removal of intravenous cardiac pacemaker system |
| **G57z.00** | Cardiac dysrhythmia NOS |
| **32J3.00** | ECG: QRS complex prolonged |
| **7937100** | Resiting of lead of cardiac pacemaker system NEC |
| **7L1H.13** | Defibrillation |
| **328Z.00** | ECG: ventricular arrhythmia NOS |
| **7936600** | Implantation of permanent intravenous cardiac pacemaker |
| **G563.00** | Left main stem bundle branch block |
| **7936H00** | Implantat intravenous dual chamber cardiac pacemaker system |
| **G565300** | Other bilateral bundle branch block |
| **329Z.00** | ECG: heart block NOS |
| **G570100** | Paroxysmal atrioventricular tachycardia |
| **32K4.00** | ECG: Q-T interval shortened |
| **G576100** | Supraventricular ectopic beats |
| **9N2b.00** | Seen by cardiac pacemaker technician |
| **Gyu5W00** | [X]Other and unspecified right bundle-branch block |
| **662S.00** | Atrial fibrillation monitoring |
| **3264.00** | ECG: atrial ectopics |
| **G562000** | Left anterior fascicular block |
| **32F2.00** | ECG: T wave abnormal |
| **ZV53300** | [V]Fitting or adjustment of cardiac pacemaker |
| **7L1H700** | External ventricular defibrillation |
| **G572000** | Essential paroxysmal tachycardia |
| **G562z00** | Left bundle branch hemiblock NOS |
| **329E.00** | ECG: left posterior fascicular block |
| **G561300** | Mobitz type I (Wenckebach) atrioventricular block |
| **7P19y00** | Other specified pacemaker testing |
| **G570z00** | Paroxysmal supraventricular tachycardia NOS |
| **14AR.00** | History of atrial flutter |
| **7.936E+03** | Implantation of intravenous dual chamber permanent pacemaker |
| **7937000** | Implantation of cardiac pacemaker system NEC |
| **G56y400** | Right fascicular block |
| **G574011** | Cardiac arrest-ventricular fibrillation |
| **32D2.00** | ECG: S wave abnormal |
| **TB01000** | Implant of cardiac pacemaker with complication, no blame |
| **8CMW200** | Atrial fibrillation care pathway |
| **9Os2.00** | Atrial fibrillation monitoring third letter |
| **Q491.00** | Neonatal cardiac dysrhythmia |
| **ZV45000** | [V]Cardiac pacemaker in situ |
| **7937400** | Removal of cardiac pacemaker system NEC |
| **G56z.00** | Conduction disorders unspecified |
| **3274.00** | ECG: paroxysmal atrial tachy. |
| **Gyu5Y00** | [X]Other specified conduction disorders |
| **9Os3.00** | Atrial fibrillation monitoring verbal invite |
| **14V1.11** | H/O: cardiac pacemaker |
| **7936000** | Implantation of intravenous cardiac pacemaker system |
| **G57y600** | Nodal rhythm disorder |

# Chronic pulmonary disease (Elixhauser)

| **Read code** | **Description** |
| --- | --- |
| **H312000** | Chronic asthmatic bronchitis |
| **H312011** | Chronic wheezy bronchitis |
| **663s.00** | Asthma never causes daytime symptoms |
| **9hA1.00** | Excepted from asthma quality indicators: Patient unsuitable |
| **H465.00** | Chemical-induced pulmonary oedema |
| **H32y.00** | Other emphysema |
| **663P.00** | Asthma limiting activities |
| **H35z100** | Hypersensitivity pneumonitis NOS |
| **H50..00** | Empyema |
| **9hA2.00** | Excepted from asthma quality indicators: Informed dissent |
| **493 HR** | ASTHMA HIGH RISK |
| **8IEy.00** | Chronic obstructive pulmon dis wr self managem plan declined |
| **1I70.00** | Chronic obstructive pulmonary disease excluded by spirometry |
| **66YB100** | Chronic obstructive pulmonary disease 6 monthly review |
| **H35..00** | Extrinsic allergic alveolitis |
| **H432.00** | Berylliosis |
| **H310z00** | Simple chronic bronchitis NOS |
| **2126200** | Asthma resolved |
| **H3...11** | Chronic obstructive airways disease |
| **H433.00** | Graphite fibrosis of lung |
| **H530z00** | Abscess of lung NOS |
| **H46..00** | Respiratory disease due to chemical fumes and vapours |
| **493 JC** | CHRONIC ASTHMA |
| **H501200** | Pleural empyema |
| **66YL.00** | Chronic obstructive pulmonary disease follow-up |
| **H3y..11** | Other specified chronic obstructive pulmonary disease |
| **H583000** | Loeffler's syndrome |
| **66k..00** | Cystic fibrosis monitoring |
| **9OJA.00** | Asthma monitoring check done |
| **H53z.00** | Abscess of lung and mediastinum NOS |
| **663O000** | Asthma never disturbs sleep |
| **663n.00** | Asthma treatment compliance satisfactory |
| **H58y200** | Pulmolithiasis |
| **H572.00** | Lung disease with systemic sclerosis |
| **493 EP** | ASTHMA EPISODIC |
| **1761.00** | C/O bronchial catarrh |
| **H35y500** | Pituitary snuff-takers' disease |
| **H331.00** | Intrinsic asthma |
| **C370900** | Exacerbation of cystic fibrosis |
| **8Hkw.00** | Referral to COPD community nursing team |
| **9OJ2.00** | Refuses asthma monitoring |
| **H564.11** | Cryptogenic organising pneumonia |
| **1788.00** | Asthma trigger - cold air |
| **493 KA** | EXACERBATION OF ASTHMA |
| **H531.00** | Abscess of mediastinum |
| **663x.00** | Asthma limits walking on the flat |
| **H33z200** | Late-onset asthma |
| **493 BI** | ASTHMA FREQUENCY ON EXERCISE ONLY |
| **H33z111** | Asthma attack NOS |
| **491 BS** | CHRONIC SPASMODIC BRONCHITIS |
| **H40..00** | Coal workers' pneumoconiosis |
| **491** | CHRONIC BRONCHITIS |
| **H301.00** | Laryngotracheobronchitis |
| **H501500** | Pyopneumothorax |
| **H530000** | Single lung abscess |
| **H53..00** | Abscess of lung and mediastinum |
| **H30z.00** | Bronchitis NOS |
| **66Yp.00** | Asthma review using Roy Colleg of Physicians three questions |
| **H581.00** | Interstitial emphysema |
| **C370200** | Cystic fibrosis with pulmonary manifestations |
| **H4yy.00** | Other external agent causing respiratory condition |
| **173A.00** | Exercise induced asthma |
| **L5161B** | BIRD FANCIER'S LUNG |
| **H52y100** | Chronic pneumothorax |
| **H33zz12** | Allergic asthma NEC |
| **663Q.00** | Asthma not limiting activities |
| **6.63E+102** | Asthma severely restricts exercise |
| **H583100** | Tropical eosinophilia |
| **H41z.00** | Asbestosis NOS |
| **518** | BRONCHIECTASIS |
| **5151.00** | ANTHRACOSILICOSIS |
| **H331100** | Intrinsic asthma with status asthmaticus |
| **491 BT** | BRONCHITIS OBSTRUCTIVE |
| **66YI.00** | COPD self-management plan given |
| **663e.00** | Asthma restricts exercise |
| **66YJ.00** | Asthma annual review |
| **H41..00** | Asbestosis |
| **C370100** | Cystic fibrosis with meconium ileus |
| **H35y.00** | Other allergic alveolitis |
| **H541z00** | Pulmonary oedema NOS |
| **663U.00** | Asthma management plan given |
| **H312z00** | Obstructive chronic bronchitis NOS |
| **9kf0.11** | COPD patient unsuitable for pulmonary rehabilitation |
| **9Nk7000** | Seen in chronic obstructive pulmonary disease clinic |
| **679J200** | Health education - structured patient focused asthma discuss |
| **9OJ4.00** | Asthma monitor 1st letter |
| **H330.00** | Extrinsic (atopic) asthma |
| **66Y5.00** | Change in asthma management plan |
| **493 EB** | ASTHMA EXERCISE INDUCED |
| **66Yr.00** | Asthma causes symptoms most nights |
| **H441.00** | Cannabinosis |
| **114 PF** | PIGEON FANCIER'S LUNG |
| **H30..12** | Recurrent wheezy bronchitis |
| **Q312300** | Perinatal interstitial emphysema |
| **1783.00** | Asthma trigger - warm air |
| **66Yh.00** | Chronic obstructive pulmonary disease does not disturb sleep |
| **493 HT** | INTRINSIC ASTHMA |
| **H312100** | Emphysematous bronchitis |
| **H43..00** | Pneumoconiosis due to other inorganic dust |
| **H530300** | Abscess of lung with pneumonia |
| **N04y012** | Fibrosing alveolitis associated with rheumatoid arthritis |
| **1786.00** | Asthma trigger - animals |
| **9OJ6.00** | Asthma monitor 3rd letter |
| **5199CL** | OBSTRUCTIVE LUNG DISEASE |
| **9OJZ.00** | Asthma monitoring admin.NOS |
| **9Oi..00** | Chronic obstructive pulmonary disease monitoring admin |
| **H56z.00** | Alveolar and parietoalveolar disease NOS |
| **H322.00** | Centrilobular emphysema |
| **U60F61A** | [X] Adverse reaction to antiasthmatic NOS |
| **H331111** | Intrinsic asthma with asthma attack |
| **H312300** | Bronchiolitis obliterans |
| **8CE6.00** | Chronic obstructive pulmonary disease leaflet given |
| **H320000** | Segmental bullous emphysema |
| **H501100** | Thorax abscess NOS |
| **9h52.00** | Excepted from COPD quality indicators: Informed dissent |
| **H57y200** | Pulmonary sarcoidosis |
| **H31..00** | Chronic bronchitis |
| **661M100** | Asthma self-management plan agreed |
| **663v.00** | Asthma causes daytime symptoms most days |
| **H460z00** | Bronchitis and pneumonitis due to chemical fumes NOS |
| **9No7.00** | Seen in cystic fibrosis clinic |
| **H352z00** | Bird-fancier's lung NOS |
| **H312200** | Acute exacerbation of chronic obstructive airways disease |
| **490 T** | TRACHEOBRONCHITIS |
| **H58z.00** | Lung disease NOS |
| **H37..00** | Moderate chronic obstructive pulmonary disease |
| **661M300** | COPD self-management plan agreed |
| **663p.00** | Asthma treatment compliance unsatisfactory |
| **H320100** | Zonal bullous emphysema |
| **H32..00** | Emphysema |
| **L4930LO** | LATE ONSET ASTHMA |
| **663** | Asthma sometimes restricts exercise |
| **H564.00** | Bronchiolitis obliterans organising pneumonia |
| **TJF7z00** | Adverse reaction to antiasthmatic NOS |
| **13Y4.00** | Asthma society member |
| **493 AJ** | ASTHMA SEVERITY MODERATE |
| **66Yd.00** | COPD accident and emergency attendance since last visit |
| **H442.00** | Flax-dressers' disease |
| **H35y200** | Fish-meal workers' lung |
| **H44..00** | Pneumopathy due to inhalation of other dust |
| **H35z.00** | Allergic alveolitis and pneumonitis NOS |
| **H583z00** | Pulmonary eosinophilia NOS |
| **7832AB** | WHEEZING BRONCHIAL |
| **8IEZ.00** | Chronic obstructive pulmonary disease rescue pack declined |
| **H563300** | Usual interstitial pneumonitis |
| **H46zz00** | Respiratory conditions due to chemical fumes NOS |
| **H335.00** | Chronic asthma with fixed airflow obstruction |
| **H464z00** | Chronic respiratory conditions due to chemical fumes NOS |
| **663V000** | Occasional asthma |
| **H46z.00** | Respiratory conditions due to chemical fumes NOS |
| **1780.00** | Aspirin induced asthma |
| **H300.00** | Tracheobronchitis NOS |
| **493 EA** | ASTHMA EXERCISE INCLUDED |
| **H500.00** | Empyema with fistula |
| **H4...00** | Lung disease due to external agents |
| **H310.00** | Simple chronic bronchitis |
| **H33z100** | Asthma attack |
| **5192CM** | OBSTRUCTIVE LUNG DISEASE COMPENSATORY |
| **H560.00** | Pulmonary alveolar proteinosis |
| **H56y000** | Endogenous lipoid pneumonia |
| **9Oi3.00** | Chronic obstructive pulmonary disease monitoring verb invite |
| **H35y700** | Wood asthma |
| **C10N100** | Cystic fibrosis related diabetes mellitus |
| **H313.00** | Mixed simple and mucopurulent chronic bronchitis |
| **H58y.00** | Other lung disease NEC |
| **H332.00** | Mixed asthma |
| **9N1d.00** | Seen in asthma clinic |
| **66YK.00** | Asthma follow-up |
| **H33z.11** | Hyperreactive airways disease |
| **66Yg.00** | Chronic obstructive pulmonary disease disturbs sleep |
| **6.63E+102** | Asthma severely restricts exercise |
| **5192BY** | BYSSINOSIS |
| **9OJ..00** | Asthma monitoring admin. |
| **AB63600** | Aspergillus bronchitis |
| **H464.00** | Chronic respiratory conditions due to chemical fumes |
| **H33zz11** | Exercise induced asthma |
| **H430.00** | Aluminosis of lung |
| **38DT.00** | Asthma control questionnaire |
| **H57..00** | Lung involvement in diseases EC |
| **H060500** | Acute tracheobronchitis |
| **H33zz13** | Allergic bronchitis NEC |
| **H48..00** | Progressive massive fibrosis |
| **H4yz.00** | External agent causing respiratory conditions NOS |
| **H321.00** | Panlobular emphysema |
| **66YB.00** | Chronic obstructive pulmonary disease monitoring |
| **9OJ9.00** | Asthma monitoring deleted |
| **663q.00** | Asthma daytime symptoms |
| **493** | ASTHMA |
| **H320311** | Tension pneumatocoele |
| **1781.00** | Asthma trigger - pollen |
| **H302.00** | Wheezy bronchitis |
| **H501600** | Pyothorax |
| **663r.00** | Asthma causes night symptoms 1 to 2 times per month |
| **6.63E+02** | Asthma sometimes restricts exercise |
| **5161F** | FARMERS' LUNG |
| **9kf1.11** | Referred for COPD structured smoking assessment |
| **493 GS** | ASTHMA POLLEN INITIATED |
| **H47y000** | Detergent asthma |
| **493 A** | BRONCHITIS ALLERGIC |
| **H434.00** | Siderosis |
| **66YZ.00** | Does not have asthma management plan |
| **H562.00** | Pulmonary alveolar microlithiasis |
| **9Oi4.00** | Chronic obstructive pulmonary disease monitor phone invite |
| **66Yf.00** | Number of COPD exacerbations in past year |
| **H56..00** | Other alveolar and parietoalveolar disease |
| **8795.00** | Asthma control step 2 |
| **66YE.00** | Asthma monitoring due |
| **H32y100** | Atrophic (senile) emphysema |
| **679J.00** | Health education - asthma |
| **SK07.00** | Subcutaneous emphysema |
| **491 AC** | BRONCHITIS ACUTE ON CHRONIC |
| **663f.00** | Asthma never restricts exercise |
| **H357.00** | 'Ventilation' pneumonitis |
| **C370000** | Cystic fibrosis with no meconium ileus |
| **H57y000** | Pulmonary amyloidosis |
| **H33z000** | Status asthmaticus NOS |
| **66YQ.00** | Asthma monitoring by nurse |
| **8798.00** | Asthma control step 5 |
| **H34z.00** | Bronchiectasis NOS |
| **466 D** | BRONCHITIS PURULENT |
| **H571.00** | Rheumatic pneumonia |
| **U60F615** | [X] Adverse reaction to theophylline - asthma |
| **493 D** | STATUS ASTHMATICUS |
| **H32y200** | MacLeod's unilateral emphysema |
| **H410.11** | Asbestos-induced pleural plaque |
| **9kf..00** | COPD - enhanced services administration |
| **H31yz00** | Other chronic bronchitis NOS |
| **H57y500** | Lung disease with syphilis |
| **H57y.00** | Lung disease with diseases EC |
| **66YS.00** | Chronic obstructive pulmonary disease monitoring by nurse |
| **H4y1.00** | Chronic pulmonary radiation disease |
| **H52yz11** | Spontaneous pneumothorax NOS |
| **H563.12** | Cryptogenic fibrosing alveolitis |
| **9NgP.11** | On COPD (chr obstruc pulmonary disease) supportv cre pathway |
| **H44z.00** | Pneumopathy due to inhalation of other dust NOS |
| **8CR0.00** | Asthma clinical management plan |
| **H331.11** | Late onset asthma |
| **H56y100** | Interstitial pneumonia |
| **8CMA000** | Patient has a written asthma personal action plan |
| **663V100** | Mild asthma |
| **H330100** | Extrinsic asthma with status asthmaticus |
| **H311000** | Purulent chronic bronchitis |
| **H500100** | Empyema with bronchopleural fistula |
| **Hyu4300** | [X]Hypersensitivity pneumonitis due to other organic dusts |
| **H352000** | Budgerigar-fanciers' lung |
| **A11..11** | Lung tuberculosis |
| **H32y000** | Acute vesicular emphysema |
| **H4...11** | Pneumoconioses |
| **66YL.11** | COPD follow-up |
| **178B.00** | Asthma trigger - exercise |
| **H431.00** | Bauxite fibrosis of lung |
| **U60F600** | [X]Antiasthmats caus adverse effects in therapeut use, NEC |
| **H530.00** | Abscess of lung |
| **H4...12** | Occupational lung disease |
| **H501.00** | Empyema with no fistula |
| **493 BD** | ASTHMA AND BRONCHITIS |
| **9OJ8.00** | Asthma monitor phone invite |
| **8791.00** | Further asthma - drug prevent. |
| **H33z011** | Severe asthma attack |
| **663V.00** | Asthma severity |
| **C370500** | Cystic fibrosis with distal intestinal obstruction syndrome |
| **679J100** | Health education - structured asthma discussion |
| **Hyu3100** | [X]Other specified chronic obstructive pulmonary disease |
| **H58yz00** | Other lung disease NEC NOS |
| **H3z..11** | Chronic obstructive pulmonary disease NOS |
| **493 AA** | ASTHMA ACUTE |
| **491 E** | CHRONIC BRONCHITIS WITH EMPHYSEMA |
| **H54z.00** | Pulmonary congestion and hypostasis NOS |
| **1789.00** | Asthma trigger - respiratory infection |
| **H58y500** | Respiratory bronchiolitis associated interstitial lung dis |
| **H57y100** | Lung disease with polymyositis |
| **H58y100** | Calcification of lung |
| **H47..00** | Pneumonitis due to inhalation of solids or liquids |
| **66Y9.00** | Step up change in asthma management plan |
| **663P000** | Asthma limits activities 1 to 2 times per month |
| **AD04.00** | Toxoplasma pneumonitis |
| **H36..00** | Mild chronic obstructive pulmonary disease |
| **C370111** | Meconium ileus in cystic fibrosis |
| **C350300** | Idiopathic pulmonary haemosiderosis |
| **66YA.00** | Step down change in asthma management plan |
| **A124300** | Tuberculosis of lung, confirmed by unspecified means |
| **H35yz00** | Other allergic alveolitis NOS |
| **8CR1.00** | Chronic obstructive pulmonary disease clini management plan |
| **H32y111** | Acute interstitial emphysema |
| **H4z..00** | Lung disease due to external agents NOS |
| **H4y2000** | Acute drug-induced interstitial lung disorders |
| **H421.00** | Simple silicosis |
| **66YD.00** | Chronic obstructive pulmonary disease monitoring due |
| **9OJA.11** | Asthma monitored |
| **Hyu3000** | [X]Other emphysema |
| **663..11** | Asthma monitoring |
| **388t000** | Royal College Physician asthma assessment 3 question score |
| **P861.00** | Congenital bronchiectasis |
| **H540100** | Hypostatic bronchopneumonia |
| **H563.13** | Idiopathic pulmonary fibrosis |
| **H422.00** | Complicated silicosis |
| **9Q21.00** | Patient in asthma study |
| **66Yu.00** | Number days absent from school due to asthma in past 6 month |
| **9OqCC00** | Cystic fibrosis repeat screening with further sample require |
| **466 BC** | BRONCHITIS SUBACUTE |
| **663y.00** | Number of asthma exacerbations in past year |
| **38Dg.00** | Chronic obstructive pulmonary disease assessment test |
| **66YC.00** | Absent from work or school due to asthma |
| **H45..00** | Pneumoconiosis NOS |
| **38DV.00** | Mini asthma quality of life questionnaire |
| **H57yz00** | Lung disease with diseases EC NOS |
| **H31z.00** | Chronic bronchitis NOS |
| **C370z00** | Cystic fibrosis NOS |
| **9e03.00** | GP OOH service notified of COPD care plan |
| **H31y.00** | Other chronic bronchitis |
| **H330011** | Hay fever with asthma |
| **493 AI** | ASTHMA SEVERITY MILD |
| **H500400** | Empyema with pleural fistula NOS |
| **H540z00** | Pulmonary hypostasis NOS |
| **691 TM** | ECZEMA WITH ASTHMA |
| **1O2..00** | Asthma confirmed |
| **H55..11** | Cirrhosis of lung |
| **H30..00** | Bronchitis unspecified |
| **8796.00** | Asthma control step 3 |
| **H541000** | Chronic pulmonary oedema |
| **492** | EMPHYSEMA PULMONARY |
| **9h51.00** | Excepted from COPD quality indicators: Patient unsuitable |
| **Hyu5000** | [X]Other interstitial pulmonary diseases with fibrosis |
| **H530200** | Gangrenous pneumonia |
| **66YM.00** | Chronic obstructive pulmonary disease annual review |
| **AB24.00** | Candidiasis of lung |
| **173d.00** | Work aggravated asthma |
| **H356.00** | Maple bark strippers' lung |
| **663N000** | Asthma causing night waking |
| **H3y..00** | Other specified chronic obstructive airways disease |
| **9kf2.00** | COPD structured smoking assessment declined - enh serv admin |
| **679V.00** | Health education - chronic obstructive pulmonary disease |
| **H333.00** | Acute exacerbation of asthma |
| **66k0.00** | Cystic fibrosis annual review |
| **1782.00** | Asthma trigger - tobacco smoke |
| **8CMW500** | Chronic obstructive pulmonary disease care pathway |
| **H56y.00** | Other alveolar and parietoalveolar disease |
| **H35zz00** | Allergic alveolitis and pneumonitis NOS |
| **H331z00** | Intrinsic asthma NOS |
| **H330111** | Extrinsic asthma with asthma attack |
| **9h5..00** | Exception reporting: COPD quality indicators |
| **H4y2100** | Chronic drug-induced interstitial lung disorders |
| **8B3j.00** | Asthma medication review |
| **H330.12** | Childhood asthma |
| **493 AC** | BRONCHITIS ALLERGIC CHRONIC |
| **1785.00** | Asthma trigger - damp |
| **h33z100** | Asthma attack |
| **H440.00** | Byssinosis |
| **H500000** | Empyema with bronchocutaneous fistula |
| **P840.00** | Congenital cystic lung disease, unspecified |
| **H563100** | Diffuse pulmonary fibrosis |
| **8BMW.00** | Issue of chronic obstructive pulmonary disease rescue pack |
| **178..00** | Asthma trigger |
| **663j.00** | Asthma - currently active |
| **9Oi2.00** | Chronic obstructive pulmonary disease monitoring 3rd letter |
| **9Oi0.00** | Chronic obstructive pulmonary disease monitoring 1st letter |
| **663N200** | Asthma disturbs sleep frequently |
| **H410.00** | Pleural plaque disease due to asbestosis |
| **H33..00** | Asthma |
| **H330.13** | Hay fever with asthma |
| **H58y700** | Interstitial lung disease due to connective tissue disease |
| **Hyu4000** | [X]Pneumoconiosis due to other dust containing silica |
| **H34..00** | Bronchiectasis |
| **H340.00** | Recurrent bronchiectasis |
| **66Yq.00** | Asthma causes night time symptoms 1 to 2 times per week |
| **H38..00** | Severe chronic obstructive pulmonary disease |
| **663N.00** | Asthma disturbing sleep |
| **H59..00** | Respiratory failure |
| **H320z00** | Chronic bullous emphysema NOS |
| **H311z00** | Mucopurulent chronic bronchitis NOS |
| **U60F611** | [X] Adverse reaction to antiasthmatics |
| **H352.00** | Bird-fancier's lung |
| **H540.00** | Pulmonary hypostasis |
| **663h.00** | Asthma - currently dormant |
| **H582.00** | Compensatory emphysema |
| **178A.00** | Asthma trigger - airborne dust |
| **1784.00** | Asthma trigger - emotion |
| **663V200** | Moderate asthma |
| **493 BG** | ASTHMA FREQUENCY REGULARLY |
| **H563.11** | Hamman - Rich syndrome |
| **H35y000** | Cheese-washers' lung |
| **H5...00** | Other respiratory system diseases |
| **8CMV.00** | Has chronic obstructive pulmonary disease care plan |
| **H3y1.00** | Chron obstruct pulmonary dis wth acute exacerbation, unspec |
| **H35y100** | Coffee-workers' lung |
| **66Yi.00** | Multiple COPD emergency hospital admissions |
| **H42z.00** | Silica pneumoconiosis NOS |
| **H561.00** | Idiopathic pulmonary haemosiderosis |
| **38Dd.00** | Clinical chronic obstructive pulmonary disease questionnaire |
| **9kf2.11** | COPD structured smoking assessment declined |
| **H593.00** | Chronic type 2 respiratory failure |
| **661N300** | COPD self-management plan review |
| **K3441B** | EXCISION BRONCHIECTASIS |
| **H350.00** | Farmers' lung |
| **H33..11** | Bronchial asthma |
| **H423.00** | Massive silicotic fibrosis |
| **14B3.12** | History of chronic obstructive pulmonary disease |
| **H311100** | Fetid chronic bronchitis |
| **9OJ3.00** | Asthma monitor offer default |
| **H341.00** | Post-infective bronchiectasis |
| **8CeD.00** | Preferred place of care for next exacerbation of COPD |
| **SLF7z00** | Antiasthmatic poisoning NOS |
| **H060z00** | Acute bronchitis NOS |
| **H563.00** | Idiopathic fibrosing alveolitis |
| **H3A..00** | End stage chronic obstructive airways disease |
| **679J000** | Health education - asthma self management |
| **H464100** | Obliterative bronchiolitis due to chemical fumes |
| **H35z000** | Allergic extrinsic alveolitis NOS |
| **H32yz00** | Other emphysema NOS |
| **663O.00** | Asthma not disturbing sleep |
| **663W.00** | Asthma prophylactic medication used |
| **H330.14** | Pollen asthma |
| **8CE2.00** | Asthma leaflet given |
| **H43z.00** | Pneumoconiosis due to inorganic dust NOS |
| **493 BR** | BRONCHIAL ASTHMA |
| **661N100** | Asthma self-management plan review |
| **493 AD** | ASTHMA OCCASIONAL |
| **H331000** | Intrinsic asthma without status asthmaticus |
| **H33zz00** | Asthma NOS |
| **H4y1z00** | Chronic pulmonary radiation disease NOS |
| **8797.00** | Asthma control step 4 |
| **H592.00** | Chronic type 1 respiratory failure |
| **H320.00** | Chronic bullous emphysema |
| **66YT.00** | Chronic obstructive pulmonary disease monitoring by doctor |
| **9OJ7.00** | Asthma monitor verbal invite |
| **H310000** | Chronic catarrhal bronchitis |
| **H31y100** | Chronic tracheobronchitis |
| **H58..00** | Other diseases of lung |
| **H330z00** | Extrinsic asthma NOS |
| **H501300** | Lung empyema NOS |
| **H334.00** | Brittle asthma |
| **66YP.00** | Asthma night-time symptoms |
| **14B4.00** | H/O: asthma |
| **TJF7.00** | Adverse reaction to antiasthmatics |
| **H58y000** | Broncholithiasis |
| **H353.00** | Suberosis ( cork-handlers' lung ) |
| **A115.00** | Tuberculous bronchiectasis |
| **9NI8.00** | Asthma outreach clinic |
| **H39..00** | Very severe chronic obstructive pulmonary disease |
| **H55..00** | Postinflammatory pulmonary fibrosis |
| **H563200** | Pulmonary fibrosis |
| **H354.00** | Malt workers' lung |
| **H530100** | Multiple lung abscess |
| **8H2P.00** | Emergency admission, asthma |
| **9NNX.00** | Under care of asthma specialist nurse |
| **66Ys.00** | Asthma never causes night symptoms |
| **H330000** | Extrinsic asthma without status asthmaticus |
| **H501400** | Purulent pleurisy |
| **H435.00** | Stannosis |
| **H56yz00** | Other alveolar and parietoalveolar disease NOS |
| **493 NA** | NOCTURNAL ASTHMA |
| **H591.00** | Chronic respiratory failure |
| **H35..11** | Hypersensitivity pneumonitis |
| **663P200** | Asthma limits activities most days |
| **TJF7300** | Adverse reaction to theophylline (asthma) |
| **13N8.00** | Bird fancier |
| **490** | BRONCHITIS |
| **H563z00** | Idiopathic fibrosing alveolitis NOS |
| **663P100** | Asthma limits activities 1 to 2 times per week |
| **9OJ1.00** | Attends asthma monitoring |
| **H310100** | Smokers' cough |
| **H330.11** | Allergic asthma |
| **C370y00** | Cystic fibrosis with other manifestations |
| **8793.00** | Asthma control step 0 |
| **H355.00** | Mushroom workers' lung |
| **H35y800** | Air-conditioner and humidifier lung |
| **H420.00** | Talc pneumoconiosis |
| **H33z.00** | Asthma unspecified |
| **H311.00** | Mucopurulent chronic bronchitis |
| **8794.00** | Asthma control step 1 |
| **H58y400** | Squamous metaplasia of lung |
| **1787.00** | Asthma trigger - seasonal |
| **9OJ5.00** | Asthma monitor 2nd letter |
| **H3y0.00** | Chronic obstruct pulmonary dis with acute lower resp infectn |
| **C370.00** | Cystic fibrosis |
| **H464200** | Chronic pulmonary fibrosis due to chemical fumes |
| **H35y300** | Furriers' lung |
| **493 KB** | ASTHMA EXACERBATION |
| **Hyu4100** | [X]Pneumoconiosis due to other specified inorganic dusts |
| **H52z.00** | Pneumothorax NOS |
| **H58y300** | Interstitial lung disease NEC |
| **H35y600** | Sequoiosis (red-cedar asthma) |
| **H464000** | Chronic emphysema due to chemical fumes |
| **H3z..00** | Chronic obstructive airways disease NOS |
| **H320300** | Bullous emphysema with collapse |
| **66YR.00** | Asthma monitoring by doctor |
| **9hA..00** | Exception reporting: asthma quality indicators |
| **9kf1.00** | Refer COPD structured smoking assessment - enhanc serv admin |
| **H3...00** | Chronic obstructive pulmonary disease |
| **9kf0.00** | COPD patient unsuitable for pulmonary rehab - enh serv admin |
| **Hyu5100** | [X]Other specified interstitial pulmonary diseases |
| **H501000** | Pleural abscess |
| **8H2R.00** | Admit COPD emergency |
| **H42..00** | Silica and silicate pneumoconiosis |
| **66Ye.00** | Emergency COPD admission since last appointment |
| **Q312.00** | Perinatal interstitial emphysema and related conditions |
| **9Oi1.00** | Chronic obstructive pulmonary disease monitoring 2nd letter |
| **9NgP.00** | On chronic obstructive pulmonary disease supprtv cre pathway |
| **173c.00** | Occupational asthma |
| **H4y1000** | Chronic pulmonary fibrosis following radiation |
| **H312.00** | Obstructive chronic bronchitis |
| **8BMa000** | Chronic obstructiv pulmonary disease medication optimisation |
| **C370300** | Cystic fibrosis with intestinal manifestations |
| **H450.00** | Pneumoconiosis associated with tuberculosis |
| **H583.00** | Pulmonary eosinophilia |
| **663N100** | Asthma disturbs sleep weekly |
| **663t.00** | Asthma causes daytime symptoms 1 to 2 times per month |
| **H540000** | Hypostatic pneumonia |
| **H32z.00** | Emphysema NOS |
| **H320200** | Giant bullous emphysema |
| **H30..11** | Chest infection - unspecified bronchitis |
| **H4y..00** | Other specified lung diseases due to external agent |
| **491 R** | BRONCHITIS RECURRENT |
| **A789900** | HIV disease resulting in lymphoid interstitial pneumonitis |
| **H351.00** | Bagassosis |
| **663m.00** | Asthma accident and emergency attendance since last visit |
| **H352100** | Pigeon-fanciers' lung |
| **C370400** | Arthropathy in cystic fibrosis |
| **493 AB** | ASTHMA ATTACK |
| **5152.00** | ASBESTOSIS |
| **H06..00** | Acute bronchitis and bronchiolitis |
| **663u.00** | Asthma causes daytime symptoms 1 to 2 times per week |
| **H50z.00** | Empyema NOS |
| **493 GR** | ASTHMA ALLERGIC GRASS |
| **663V300** | Severe asthma |
| **H583200** | Eosinophilic bronchitis |
| **H4y2.00** | Drug-induced interstitial lung disorders |
| **663d.00** | Emergency asthma admission since last appointment |
| **663w.00** | Asthma limits walking up hills or stairs |
| **H460.00** | Bronchitis and pneumonitis due to chemical fumes |
| **66YB000** | Chronic obstructive pulmonary disease 3 monthly review |

# Coagulopathy (Elixhauser)

| **Read code** | **Description** |
| --- | --- |
| **D303000** | Congenital afibrinogenaemia |
| **D307211** | Acquired prothrombin deficiency |
| **42P2.00** | Thrombocytopenia |
| **D313.12** | Idiopathic thrombocytopenic purpura |
| **D306.00** | Defibrination syndrome |
| **L363.00** | Postpartum coagulation defects |
| **D314z00** | Secondary thrombocytopenia NOS |
| **D313.11** | Evan's syndrome |
| **D311.00** | Qualitative platelet defects |
| **9b93100** | Haemophilia - specialty |
| **B937400** | Essential (haemorrhagic) thrombocythaemia |
| **D303100** | Deficiency of factor II or prothrombin |
| **42P8.00** | Heparin induced thrombocytopenia screening test |
| **42QB.11** | Plasma factor VIII level |
| **D301.12** | Haemophilia B |
| **D314y00** | Other specified secondary thrombocytopenia |
| **D302.00** | Haemophilia-C (factor XI deficiency) |
| **D314300** | Heparin-induced thrombocytopenia |
| **D305100** | Haemorrhagic disorder due to hyperheparinaemia |
| **42Qj.00** | Factor VIII inhibitor activity |
| **D313200** | Thrombocytopenic purpura with absent radius |
| **Q451z00** | Transient neonatal thrombocytopenia NOS |
| **D300.11** | Antihaemophilic globulin deficiency |
| **D300.12** | Haemophilia A |
| **D313100** | Congenital thrombocytopenic purpura |
| **D311z00** | Qualitative platelet deficiency NOS |
| **D306.12** | Disseminated intravascular coagulation |
| **42P3.00** | Thrombocythaemia |
| **B937411** | Primary thrombocythaemia |
| **D307200** | Acquired factor II deficiency |
| **42P2.11** | Auto-immune thrombocytopenia |
| **D31..00** | Purpura and other haemorrhagic conditions |
| **D311000** | Hereditary haemorrhagic thrombasthenia |
| **B937500** | Idiopathic thrombocythaemia |
| **D307100** | Deficiency of coagulation factor due to vitamin K deficiency |
| **D31yz00** | Other specified haemorrhagic condition NOS |
| **D304.00** | Von Willebrand's disease |
| **D313z11** | Essential thrombocytopenia NOS |
| **D303.00** | Congenital deficiency of other clotting factors |
| **D313z00** | Primary thrombocytopenia NOS |
| **D313011** | Idiopathic purpura |
| **D313300** | [X]Essential thrombocytopenia NOS |
| **D313012** | ITP - idiopathic thrombocytopenic purpura |
| **Q451y11** | Neonatal thrombocytopenia due to platelet alloimmunisation |
| **D31z.00** | Haemorrhagic condition NOS |
| **D305000** | Haemorrhagic disorder due to antithrombinaemia |
| **B937.12** | Idiopathic thrombocythaemia |
| **D311.11** | Bernard - Soulier thrombopathy |
| **D303200** | Deficiency of factor V or labile factor |
| **C391211** | Thrombocytopenic eczema with immunodeficiency |
| **D307z00** | Acquired coagulation factor deficiency NOS |
| **D303111** | Hypoprothrombinaemia |
| **D301.11** | Christmas disease |
| **Dyu3300** | [X]Other specified haemorrhagic conditions |
| **D313111** | Hereditary thrombocytopenia NEC |
| **D307000** | Deficiency of coagulation factor due to liver disease |
| **D303600** | Deficiency of factor XIII or fibrin stabilizing factor |
| **Dyu3400** | [X]Haemorrhagic condition, unspecified |
| **Dyu3000** | [X]Other specified coagulation defects |
| **D303611** | Fibrin stabilising factor deficiency |
| **D307.00** | Acquired coagulation factor deficiency |
| **42Qr.00** | Prothrombin consumption |
| **D303400** | Deficiency of factor X or Stuart-Prower factor |
| **BBs4.00** | [M]Idiopathic thrombocythaemia |
| **D303y00** | Congenital deficiency of other clotting factor OS |
| **D307y00** | Other specified acquired coagulation factor deficiency |
| **D303z00** | Congenital deficiency of other clotting factor NOS |
| **D313y00** | Other specified primary thrombocytopenia |
| **D303300** | Deficiency of factor VII or stable factor |
| **D306.11** | Afibrinogenaemia - acquired |
| **D313.15** | Thrombocytopenic purpura |
| **D305.00** | Haemorrhagic disorder due to circulating anticoagulants |
| **C290.00** | Vitamin K deficiency |
| **L363z00** | Postpartum coagulation defects NOS |
| **N032000** | Arthropathy due to haemophilia |
| **D314100** | Thrombocytopenia due to drugs |
| **D307212** | Acquired hypoprothrombinaemia |
| **G756100** | Thrombotic thrombocytopenic purpura |
| **D302.12** | Rosenthal's disease |
| **42Ql.00** | Factor VIII von Willebrands Factor ratio |
| **D302.11** | Haemophilia C |
| **Dyu3200** | [X]Other primary thrombocytopenia |
| **42QB.00** | Factor VIII assay |
| **88A8.11** | Fibrinolysis |
| **D314200** | Thrombocytopenia due to extracorporeal circulation of blood |
| **D301.00** | Haemophilia-B (factor IX deficiency) |
| **D313.00** | Primary thrombocytopenia |
| **D300.00** | Haemophilia-A (factor VIII deficiency) |
| **D315.00** | Thrombocytopenia NOS |
| **D303500** | Deficiency of factor XII or Hageman factor |
| **D313000** | Idiopathic thrombocytopenic purpura |
| **D314.00** | Secondary thrombocytopenia |
| **D30..00** | Coagulation defects |

# Congestive heart failure (Elixhauser)

| **Read code** | **Description** |
| --- | --- |
| **8CMW800** | Heart failure clinical pathway |
| **8CMK.00** | Has heart failure management plan |
| **G580.14** | Biventricular failure |
| **8HHz.00** | Referral to heart failure exercise programme |
| **662W.00** | Heart failure annual review |
| **585g.00** | Echocardiogram shows left ventricular diastolic dysfunction |
| **14AM.00** | H/O: Heart failure in last year |
| **G580300** | Compensated cardiac failure |
| **G554000** | Congestive cardiomyopathy |
| **4270.00** | CONGESTIVE HEART FAILURE |
| **9m5..00** | High risk of heart failure screening invitation |
| **9N0k.00** | Seen in heart failure clinic |
| **4270R** | HEART FAILURE RIGHT-SIDED |
| **8HTL000** | Referral to rapid access heart failure clinic |
| **1O1..00** | Heart failure confirmed |
| **SP11111** | Heart failure as a complication of care |
| **9N2p.00** | Seen by community heart failure nurse |
| **402 C** | HYPERTENSION CONGESTIVE HEART FAILURE |
| **G5yyC00** | Diastolic dysfunction |
| **9Or2.00** | Heart failure monitoring verbal invite |
| **9Or5.00** | Heart failure monitoring third letter |
| **585k.00** | Echocardiogram shows normal left ventricular function |
| **G581.00** | Left ventricular failure |
| **679W100** | Education about deteriorating heart failure |
| **9N6T.00** | Referred by heart failure nurse specialist |
| **7824AC** | HEART FAILURE ACUTE |
| **9hH0.00** | Excepted heart failure quality indicators: Patient unsuitabl |
| **ZRad.00** | New York Heart Assoc classification heart failure symptoms |
| **G581.13** | Impaired left ventricular function |
| **G5y4z00** | Post cardiac operation heart failure NOS |
| **G580200** | Decompensated cardiac failure |
| **67D4.00** | Heart failure information given to patient |
| **8HTL.00** | Referral to heart failure clinic |
| **Q48y100** | Congenital cardiac failure |
| **8H2S.00** | Admit heart failure emergency |
| **G583.12** | Heart failure with preserved ejection fraction |
| **8B29.00** | Cardiac failure therapy |
| **G580400** | Congestive heart failure due to valvular disease |
| **662p.00** | Heart failure 6 month review |
| **8IE0.00** | Referral to heart failure education group declined |
| **G58z.00** | Heart failure NOS |
| **4271B** | CARDIAC ASTHMA |
| **G581.12** | Pulmonary oedema - acute |
| **G580000** | Acute congestive heart failure |
| **662f.00** | New York Heart Association classification - class I |
| **R2y1000** | [D]Cardiorespiratory failure |
| **Q490.00** | Neonatal cardiac failure |
| **8HBE.00** | Heart failure follow-up |
| **14A6.00** | H/O: heart failure |
| **679X.00** | Heart failure education |
| **4271A** | LEFT VENTRICULAR FAILURE ACUTE |
| **8CeC.00** | Preferred place of care for next exacerbation heart failure |
| **388D.00** | New York Heart Assoc classification heart failure symptoms |
| **8HHb.00** | Referral to heart failure nurse |
| **4270CC** | CONGESTIVE HEART FAILURE COMPENSATED |
| **425 CC** | CONGESTIVE CARDIOMYOPATHY |
| **9hH1.00** | Excepted heart failure quality indicators: Informed dissent |
| **G5yy900** | Left ventricular systolic dysfunction |
| **G5yyB00** | Right ventricular diastolic dysfunction |
| **585f.00** | Echocardiogram shows left ventricular systolic dysfunction |
| **SP11200** | Cardiorespiratory failure as a complication of care |
| **G581.11** | Asthma - cardiac |
| **G1yz100** | Rheumatic left ventricular failure |
| **G5yyA00** | Left ventricular diastolic dysfunction |
| **662g.00** | New York Heart Association classification - class II |
| **8HgD.00** | Discharge from heart failure nurse service |
| **G583.11** | HFNEF - heart failure with normal ejection fraction |
| **G582.00** | Acute heart failure |
| **662h.00** | New York Heart Association classification - class III |
| **4271H** | HEART FAILURE LEFT-SIDED |
| **7824FM** | MYOCARDIAL FAILURE |
| **662i.00** | New York Heart Association classification - class IV |
| **9hH..00** | Exception reporting: heart failure quality indicators |
| **8CL3.00** | Heart failure care plan discussed with patient |
| **G580.11** | Congestive cardiac failure |
| **G580100** | Chronic congestive heart failure |
| **SP11100** | Cardiac insufficiency as a complication of care |
| **9N4s.00** | Did not attend practice nurse heart failure clinic |
| **8Hk0.00** | Referred to heart failure education group |
| **G580.12** | Right heart failure |
| **8IE1.00** | Referral to heart failure exercise programme declined |
| **8Hg8.00** | Discharge from practice nurse heart failure clinic |
| **G58z.11** | Weak heart |
| **G583.00** | Heart failure with normal ejection fraction |
| **9Or..00** | Heart failure monitoring administration |
| **661M500** | Heart failure self-management plan agreed |
| **G581000** | Acute left ventricular failure |
| **G580.00** | Congestive heart failure |
| **4270D** | CONGESTIVE HEART FAILURE DECOMPENSATED |
| **4270C** | CONGESTIVE CARDIAC FAILURE |
| **9Or0.00** | Heart failure review completed |
| **G580.13** | Right ventricular failure |
| **9Or1.00** | Heart failure monitoring telephone invite |
| **9Or4.00** | Heart failure monitoring second letter |
| **G58..00** | Heart failure |
| **7824FH** | HEART FAILURE |
| **662T.00** | Congestive heart failure monitoring |
| **G584.00** | Right ventricular failure |
| **7824BW** | WEAK HEART |
| **4271.00** | LVF (LEFT VENTRICULAR FAILURE) |
| **G58z.12** | Cardiac failure NOS |
| **G58..11** | Cardiac failure |
| **9Or3.00** | Heart failure monitoring first letter |

# Deficiency anaemia (Elixhauser)

| **Read code** | **Description** |
| --- | --- |
| **D010.12** | Biermer's congenital pernicious anaemia |
| **D013.00** | Other specified megaloblastic anaemia NEC |
| **C294300** | Iron deficiency |
| **D00z.00** | Unspecified iron deficiency anaemia |
| **D012200** | Folate-deficiency anaemia, drug induced |
| **D00z100** | Chlorotic anaemia |
| **D01z.00** | Other deficiency anaemias NOS |
| **D012.11** | Folic acid deficiency anaemia |
| **F381500** | Myasthenic syndrome due to pernicious anaemia |
| **D00yz00** | Other specified iron deficiency anaemia NOS |
| **D011z00** | Other vitamin B12 deficiency anaemia NOS |
| **D01..00** | Other deficiency anaemias |
| **D012z00** | Folate-deficiency anaemia NOS |
| **D011.00** | Other vitamin B12 deficiency anaemias |
| **D011.11** | Vitamin B12 deficiency anaemia |
| **D010.00** | Pernicious anaemia |
| **1452.00** | H/O: Anaemia vit.B12 deficient |
| **U604100** | [X]Vit B12/folic/oth ant-megalobl-anaem caus adv ef ther use |
| **D00y000** | Sideropenic dysphagia |
| **D011100** | Vit B12 defic anaemia due to malabsorption with proteinuria |
| **D014000** | Amino-acid deficiency anaemia |
| **Dyu0600** | [X]Vitamin B12 deficiency anaemia, unspecified |
| **D014.00** | Protein-deficiency anaemia |
| **Dyu0200** | [X]Other vitamin B12 deficiency anaemias |
| **D0z..00** | Deficiency anaemias NOS |
| **D00y.00** | Other specified iron deficiency anaemia |
| **D012300** | Folate-deficiency anaemia due to malabsorption |
| **D011013** | Vegan's anaemia |
| **66E6.00** | Reticulcytosis after B12 |
| **D01y000** | Vitamin C deficiency anaemia |
| **D012.00** | Folate-deficiency anaemia |
| **D00y012** | Plummer-Vinson syndrome |
| **D0...12** | Sideropenic anaemia |
| **D010.11** | Addison's anaemia |
| **D012100** | Folate-deficiency anaemia due to dietary causes |
| **L182500** | Iron deficiency anaemia of pregnancy |
| **Dyu0100** | [X]Other dietary vitamin B12 deficiency anaemia |
| **D001.00** | Iron deficiency anaemia due to dietary causes |
| **D00..12** | Microcytic - hypochromic anaemia |
| **D012400** | Folate-deficiency anaemia due to liver disorders |
| **D013z00** | Other specified megaloblastic anaemia NEC NOS |
| **D012112** | Megaloblastic anaemia due to dietary causes |
| **D011011** | Imerslund - Grasbeck syndrome |
| **D013000** | Combined B12 and folate deficiency anaemia |
| **D0...00** | Deficiency anaemias |
| **D00zz00** | Iron deficiency anaemia NOS |
| **D00..00** | Iron deficiency anaemias |
| **D01y100** | Vitamin E deficiency anaemia |
| **Dyu0000** | [X]Other iron deficiency anaemias |
| **D0y..00** | Other specified deficiency anaemias |
| **D00z200** | Idiopathic hypochromic anaemia |
| **D00y.12** | Plummer - Vinson syndrome |
| **D014z00** | Protein-deficiency anaemia NOS |
| **D01..11** | Megaloblastic anaemia |
| **D012111** | Goat's milk anaemia |
| **D00z000** | Achlorhydric anaemia |
| **D011000** | Vitamin B12 deficiency anaemia due to dietary causes |
| **D011X00** | Vitamin B12 deficiency anaemia, unspecified |
| **D01yz00** | Other specified nutritional deficiency anaemia NOS |
| **D01z000** | [X]Megaloblastic anaemia NOS |
| **D01z.11** | Megaloblastic anaemia NOS |
| **Dyu0300** | [X]Other folate deficiency anaemias |

# Depression (Elixhauser)

| **Read code** | **Description** |
| --- | --- |
| **E2B1.00** | Chronic depression |
| **Eu33214** | [X]Vital depression, recurrent without psychotic symptoms |
| **Eu32211** | [X]Single episode agitated depressn w'out psychotic symptoms |
| **E112300** | Single major depressive episode, severe, without psychosis |
| **1BT..00** | Depressed mood |
| **9Ov..00** | Depression monitoring administration |
| **E211200** | Depressive personality disorder |
| **Eu32.11** | [X]Single episode of depressive reaction |
| **62T1.00** | Puerperal depression |
| **Eu34100** | [X]Dysthymia |
| **E112z00** | Single major depressive episode NOS |
| **9Ov2.00** | Depression monitoring third letter |
| **Eu34113** | [X]Neurotic depression |
| **Eu32600** | [X]Major depression, moderately severe |
| **8BK0.00** | Depression management programme |
| **Eu33000** | [X]Recurrent depressive disorder, current episode mild |
| **Eu32313** | [X]Single episode of psychotic depression |
| **Eu32z13** | [X]Prolonged single episode of reactive depression |
| **E113300** | Recurrent major depressive episodes, severe, no psychosis |
| **Eu41200** | [X]Mixed anxiety and depressive disorder |
| **E11..12** | Depressive psychoses |
| **Eu33z11** | [X]Monopolar depression NOS |
| **E112.00** | Single major depressive episode |
| **Eu53011** | [X]Postnatal depression NOS |
| **Eu32212** | [X]Single episode major depression w'out psychotic symptoms |
| **E204.00** | Neurotic depression reactive type |
| **Eu33.12** | [X]Recurrent episodes of psychogenic depression |
| **E118.00** | Seasonal affective disorder |
| **Eu32y12** | [X]Single episode of masked depression NOS |
| **Eu25111** | [X]Schizoaffective psychosis, depressive type |
| **Eu32000** | [X]Mild depressive episode |
| **Eu31.13** | [X]Manic-depressive reaction |
| **Eu3y111** | [X]Recurrent brief depressive episodes |
| **E112100** | Single major depressive episode, mild |
| **Eu32100** | [X]Moderate depressive episode |
| **Eu43212** | [X]Grief reaction |
| **9Ov1.00** | Depression monitoring second letter |
| **E2B0.00** | Postviral depression |
| **6658000** | Antidepressant drug treatment changed |
| **E290z00** | Brief depressive reaction NOS |
| **E290000** | Grief reaction |
| **ZV11111** | [V]Personal history of manic-depressive psychosis |
| **Eu31.12** | [X]Manic-depressive psychosis |
| **8HHq.00** | Referral for guided self-help for depression |
| **9k4..00** | Depression - enhanced services administration |
| **Eu32z12** | [X]Depressive disorder NOS |
| **E113200** | Recurrent major depressive episodes, moderate |
| **E112.12** | Endogenous depression first episode |
| **2257.00** | O/E - depressed |
| **E130.11** | Psychotic reactive depression |
| **E11y200** | Atypical depressive disorder |
| **Eu33.13** | [X]Recurrent episodes of reactive depression |
| **1B1U.00** | Symptoms of depression |
| **Eu33z00** | [X]Recurrent depressive disorder, unspecified |
| **E113600** | Recurrent major depressive episodes, in full remission |
| **Eu20400** | [X]Post-schizophrenic depression |
| **TJ90z00** | Adverse reaction to antidepressants NOS |
| **E113.11** | Endogenous depression - recurrent |
| **13Y3.00** | Manic-depression association member |
| **Eu33314** | [X]Recurr severe episodes/psychogenic depressive psychosis |
| **8CAa.00** | Patient given advice about management of depression |
| **Eu33.11** | [X]Recurrent episodes of depressive reaction |
| **E112.13** | Endogenous depression first episode |
| **E115600** | Bipolar affective disorder, now depressed, in full remission |
| **Eu33.14** | [X]Seasonal depressive disorder |
| **E004300** | Arteriosclerotic dementia with depression |
| **E112.14** | Endogenous depression |
| **Eu32312** | [X]Single episode of psychogenic depressive psychosis |
| **9HA0.00** | On depression register |
| **E291.00** | Prolonged depressive reaction |
| **Eu32z14** | [X] Reactive depression NOS |
| **Eu41211** | [X]Mild anxiety depression |
| **E135.00** | Agitated depression |
| **Eu32314** | [X]Single episode of reactive depressive psychosis |
| **Eu33313** | [X]Recurr severe episodes/major depression+psychotic symptom |
| **E115200** | Bipolar affective disorder, currently depressed, moderate |
| **Eu32y00** | [X]Other depressive episodes |
| **Eu32.12** | [X]Single episode of psychogenic depression |
| **1B17.00** | Depressed |
| **E113000** | Recurrent major depressive episodes, unspecified |
| **E11z200** | Masked depression |
| **E113100** | Recurrent major depressive episodes, mild |
| **Eu33400** | [X]Recurrent depressive disorder, currently in remission |
| **Eu34112** | [X]Depressive personality disorder |
| **Eu32y11** | [X]Atypical depression |
| **E112500** | Single major depressive episode, partial or unspec remission |
| **6659000** | Antidepressant drug treatment started |
| **9H92.00** | Depression interim review |
| **212S.00** | Depression resolved |
| **Eu32z11** | [X]Depression NOS |
| **Eu33.15** | [X]SAD - Seasonal affective disorder |
| **Eu33200** | [X]Recurr depress disorder cur epi severe without psyc sympt |
| **6G00.00** | Postnatal depression counselling |
| **E115500** | Bipolar affect disord, now depressed, part/unspec remission |
| **Eu33211** | [X]Endogenous depression without psychotic symptoms |
| **Eu32.13** | [X]Single episode of reactive depression |
| **E204.11** | Postnatal depression |
| **Eu32300** | [X]Severe depressive episode with psychotic symptoms |
| **Eu33.00** | [X]Recurrent depressive disorder |
| **1465.00** | H/O: depression |
| **E113500** | Recurrent major depressive episodes,partial/unspec remission |
| **E200300** | Anxiety with depression |
| **9kQ..00** | On full dose long term treatment depression - enh serv admin |
| **Eu33312** | [X]Manic-depress psychosis,depressed type+psychotic symptoms |
| **E112600** | Single major depressive episode, in full remission |
| **E290.00** | Brief depressive reaction |
| **Eu32500** | [X]Major depression, mild |
| **9k40.00** | Depression - enhanced service completed |
| **U609211** | [X] Adverse reaction to antidepressant |
| **Eu32200** | [X]Severe depressive episode without psychotic symptoms |
| **Eu32z00** | [X]Depressive episode, unspecified |
| **E112.11** | Agitated depression |
| **E113z00** | Recurrent major depressive episode NOS |
| **U609213** | [X] Adverse reaction to antidepressants NOS |
| **E115.11** | Manic-depressive - now depressed |
| **9H90.00** | Depression annual review |
| **Eu32800** | [X]Major depression, severe with psychotic symptoms |
| **Eu34114** | [X]Persistant anxiety depression |
| **Eu33y00** | [X]Other recurrent depressive disorders |
| **9Ov0.00** | Depression monitoring first letter |
| **9H91.00** | Depression medication review |
| **E115100** | Bipolar affective disorder, currently depressed, mild |
| **665A000** | Antidepressant drug treatment stopped |
| **Eu31300** | [X]Bipolar affect disorder cur epi mild or moderate depressn |
| **Eu25100** | [X]Schizoaffective disorder, depressive type |
| **Eu33315** | [X]Recurrent severe episodes of psychotic depression |
| **Eu33213** | [X]Manic-depress psychosis,depressd,no psychotic symptoms |
| **Eu32700** | [X]Major depression, severe without psychotic symptoms |
| **Eu33212** | [X]Major depression, recurrent without psychotic symptoms |
| **Eu32213** | [X]Single episode vital depression w'out psychotic symptoms |
| **E113700** | Recurrent depression |
| **Eu32.00** | [X]Depressive episode |
| **E115z00** | Bipolar affective disorder, currently depressed, NOS |
| **E112000** | Single major depressive episode, unspecified |
| **E113.00** | Recurrent major depressive episode |
| **Eu33100** | [X]Recurrent depressive disorder, current episode moderate |
| **E112200** | Single major depressive episode, moderate |
| **Eu53012** | [X]Postpartum depression NOS |
| **Eu25112** | [X]Schizophreniform psychosis, depressive type |
| **Eu92000** | [X]Depressive conduct disorder |
| **Eu34111** | [X]Depressive neurosis |
| **E2B..00** | Depressive disorder NEC |
| **E115300** | Bipolar affect disord, now depressed, severe, no psychosis |
| **Eu32400** | [X]Mild depression |
| **TJ90.00** | Adverse reaction to antidepressants |

# Diabetes, uncomplicated (Elixhauser)

| **Read code** | **Description** |
| --- | --- |
| **C109J12** | Insulin treated Type II diabetes mellitus |
| **66AJ100** | Brittle diabetes |
| **2G5K.00** | O/E - Left diabetic foot at high risk |
| **C10F700** | Type 2 diabetes mellitus - poor control |
| **1434.00** | H/O: diabetes mellitus |
| **42WZ.00** | Hb. A1C - diabetic control NOS |
| **8HLE.00** | Diabetology D.V. done |
| **9N1Q.00** | Seen in diabetic clinic |
| **66AO.00** | Date diabetic treatment stopp. |
| **C10G.00** | Secondary pancreatic diabetes mellitus |
| **ZRB6.11** | DWBQ - Diabetes wellbeing questionnaire |
| **C10FJ11** | Insulin treated Type II diabetes mellitus |
| **C10FD00** | Type 2 diabetes mellitus with hypoglycaemic coma |
| **ZRB6.00** | Diabetes wellbeing questionnaire |
| **C10F911** | Type II diabetes mellitus without complication |
| **8IAs.00** | Diabetic dietary review declined |
| **L180700** | Pre-existing malnutrition-related diabetes mellitus |
| **8HTi.00** | Referral to multidisciplinary diabetic clinic |
| **C10F900** | Type 2 diabetes mellitus without complication |
| **Cyu2.00** | [X]Diabetes mellitus |
| **9m02.00** | Eligibility temporarily inactive for diabetic retinop screen |
| **918T.00** | Diabetes key contact |
| **ZC2C800** | Dietary advice for diabetes mellitus |
| **250 G** | ULCER DIABETIC |
| **2G5A.00** | O/E - Right diabetic foot at risk |
| **ZLA2500** | Seen by diabetic liaison nurse |
| **C10C.11** | Maturity onset diabetes in youth |
| **C10E812** | Insulin dependent diabetes mellitus - poor control |
| **9m00.00** | Eligible for diabetic retinopathy screening |
| **C10FP11** | Type II diabetes mellitus with ketoacidotic coma |
| **66AK.00** | Diabetic - cooperative patient |
| **66A5.00** | Diabetic on insulin |
| **9360.00** | Patient held diabetic record issued |
| **9OLA.00** | Diabetes monitor. check done |
| **66Ao.00** | Diabetes type 2 review |
| **C10E.12** | Insulin dependent diabetes mellitus |
| **66At011** | Type 1 diabetic dietary review |
| **8HHy.00** | Referral to diabetic register |
| **ZC2C900** | Dietary advice for type I diabetes |
| **C10D.00** | Diabetes mellitus autosomal dominant type 2 |
| **68AB.00** | Diabetic digital retinopathy screening offered |
| **C109712** | Type 2 diabetes mellitus - poor control |
| **661M400** | Diabetes self-management plan agreed |
| **66Aa.00** | Diabetic diet - poor compliance |
| **L180500** | Pre-existing diabetes mellitus, insulin-dependent |
| **C109711** | Type II diabetes mellitus - poor control |
| **250 AB** | ABSCESS DIABETIC |
| **2G5I.00** | O/E - Left diabetic foot at low risk |
| **9OL2.00** | Refuses diabetes monitoring |
| **9OLB.00** | Attended diabetes structured education programme |
| **ZLD7500** | Discharge by diabetic liaison nurse |
| **M037200** | Cellulitis in diabetic foot |
| **250 HP** | PRECOMA DIABETIC |
| **250 NT** | UNSTABLE DIABETIC |
| **9NN8.00** | Under care of diabetologist |
| **250 PR** | PRURITUS DIABETIC |
| **9NiE.00** | Did not attend XPERT diabetes structured education programme |
| **C109.12** | Type 2 diabetes mellitus |
| **250 JA** | DIABETIC ACIDOSIS |
| **8A12.00** | Diabetic crisis monitoring |
| **66AM.00** | Diabetic - follow-up default |
| **C10B000** | Steroid induced diabetes mellitus without complication |
| **C102000** | Diabetes mellitus, juvenile type, with hyperosmolar coma |
| **9Oy0200** | Diabetic foot screening invitation first letter |
| **C10A100** | Malnutrition-related diabetes mellitus with ketoacidosis |
| **9NiC.00** | Did not attend DAFNE diabetes structured education programme |
| **66AV.00** | Diabetic on insulin and oral treatment |
| **66As.00** | Diabetic on subcutaneous treatment |
| **3883.00** | Diabetes treatment satisfaction questionnaire |
| **9OL8.00** | Diabetes monitor.phone invite |
| **679R.00** | Patient offered diabetes structured education programme |
| **C108.00** | Insulin dependent diabetes mellitus |
| **250 A** | SUGAR DIABETES |
| **8Hj3.00** | Referral to DAFNE diabetes structured education programme |
| **TJ23z00** | Adverse reaction to insulins and antidiabetic agents NOS |
| **C10F.11** | Type II diabetes mellitus |
| **9m0E.00** | Excluded from diabetic retinopathy screen physical disorder |
| **8H4e.00** | Referral to diabetes special interest general practitioner |
| **C10EM11** | Type I diabetes mellitus with ketoacidosis |
| **9OLG.00** | Attended XPERT diabetes structured education programme |
| **67IJ100** | Pre-conception advice for diabetes mellitus |
| **9N1o.00** | Seen in multidisciplinary diabetic clinic |
| **ZRB5.11** | DTSQ - Diabetes treatment satisfaction questionnaire |
| **C10EN00** | Type 1 diabetes mellitus with ketoacidotic coma |
| **9NND.00** | Under care of diabetic foot screener |
| **C10P011** | Type 1 diabetes mellitus in remission |
| **9OL1.00** | Attends diabetes monitoring |
| **66AN.00** | Date diabetic treatment start |
| **C10FK11** | Hyperosmolar non-ketotic state in type II diabetes mellitus |
| **9m0D.00** | Excluded from diabetic retinopthy screen as learn disability |
| **8HTk.00** | Referral to diabetic eye clinic |
| **C100100** | Diabetes mellitus, adult onset, no mention of complication |
| **C10EE00** | Type 1 diabetes mellitus with hypoglycaemic coma |
| **8CS0.00** | Diabetes care plan agreed |
| **8BL2.00** | Patient on maximal tolerated therapy for diabetes |
| **2G5F.00** | O/E - Right diabetic foot at moderate risk |
| **66o..00** | Further diabetic monitoring |
| **66AQ000** | Unsuitable for diabetes year of care programme |
| **8I94.00** | Diabetes structured education programme not available |
| **C10P000** | Type I diabetes mellitus in remission |
| **9h42.00** | Excepted from diabetes quality indicators: Informed dissent |
| **44V3.00** | Glucose tol. test diabetic |
| **C109.00** | Non-insulin dependent diabetes mellitus |
| **9m04.00** | Excluded from diabetic retinopathy screening |
| **66A9.00** | Understands diet - diabetes |
| **C109900** | Non-insulin-dependent diabetes mellitus without complication |
| **U602311** | [X] Adverse reaction to insulins and antidiabetic agents |
| **9N4p.00** | Did not attend diabetic retinopathy clinic |
| **66A1.00** | Initial diabetic assessment |
| **679L000** | Education in self management of diabetes |
| **9OLD.00** | Diabetic patient unsuitable for digital retinal photography |
| **250 DC** | DIETARY CONTROL DIABETES |
| **C108E00** | Insulin dependent diabetes mellitus with hypoglycaemic coma |
| **66AZ.00** | Diabetic monitoring NOS |
| **ZRB4.00** | Diabetes clinic satisfaction questionnaire |
| **250 AN** | DIABETES |
| **8I83.00** | Did not complete DESMOND diabetes structured educat program |
| **66AH200** | Conversion to insulin by diabetes specialist nurse |
| **C108912** | Type 1 diabetes mellitus maturity onset |
| **C10E411** | Unstable type I diabetes mellitus |
| **66A2.00** | Follow-up diabetic assessment |
| **13AB.00** | Diabetic lipid lowering diet |
| **3882.00** | Diabetes well being questionnaire |
| **66AT.00** | Annual diabetic blood test |
| **8Hj1.00** | Family/carer referral to diabetes structured education prog |
| **9N0n.00** | Seen in community diabetes specialist clinic |
| **C108911** | Type I diabetes mellitus maturity onset |
| **C10C.12** | Maturity onset diabetes in youth type 1 |
| **2G5J.00** | O/E - Left diabetic foot at moderate risk |
| **66AY.00** | Diabetic diet - good compliance |
| **9Oy0000** | Diabetic foot screening invitation |
| **66At.00** | Diabetic dietary review |
| **9OL4.00** | Diabetes monitoring 1st letter |
| **ZL62500** | Referral to diabetes nurse |
| **C10FK00** | Hyperosmolar non-ketotic state in type 2 diabetes mellitus |
| **66AU.00** | Diabetes care by hospital only |
| **ZV65312** | [V]Dietary counselling in diabetes mellitus |
| **8Hg4.00** | Discharged from care of diabetes specialist nurse |
| **C10FS00** | Maternally inherited diabetes mellitus |
| **9N2i.00** | Seen by diabetic liaison nurse |
| **8Hlc.00** | Referral to community diabetes service |
| **8I3W.00** | Diabetic foot examination declined |
| **C108412** | Unstable type 1 diabetes mellitus |
| **C10FD11** | Type II diabetes mellitus with hypoglycaemic coma |
| **C10B.00** | Diabetes mellitus induced by steroids |
| **9h43.00** | Excepted from diabetes qual indicators: service unavailable |
| **9OLH.00** | Attended DAFNE diabetes structured education programme |
| **C10FN00** | Type 2 diabetes mellitus with ketoacidosis |
| **66Af.00** | Patient diabetes education review |
| **C108900** | Insulin dependent diabetes maturity onset |
| **8I84.00** | Did not complete XPERT diabetes structured education program |
| **9m0..00** | Diabetic retinopathy screening administrative status |
| **C10E800** | Type 1 diabetes mellitus - poor control |
| **2BBK.00** | O/E - no left diabetic retinopathy |
| **8H7f.00** | Referral to diabetes nurse |
| **250 DR** | DIABETIC DIARRHOEA |
| **8H7C.00** | Refer, diabetic liaison nurse |
| **66Ac.00** | Diabetic peripheral neuropathy screening |
| **9NJy.00** | In-house diabetic foot screening |
| **2G5d.00** | O/E - Left diabetic foot at increased risk |
| **C10EM00** | Type 1 diabetes mellitus with ketoacidosis |
| **66A..00** | Diabetic monitoring |
| **66Ai.00** | Diabetic 6 month review |
| **L180600** | Pre-existing diabetes mellitus, non-insulin-dependent |
| **66AI.00** | Diabetic - good control |
| **8H2J.00** | Admit diabetic emergency |
| **8CE0.00** | Diabetic leaflet given |
| **250 CT** | DIABETIC CATARACT |
| **66AJz00** | Diabetic - poor control NOS |
| **9N1i.00** | Seen in diabetic foot clinic |
| **9Oy0400** | Diabetic foot screening invitation third letter |
| **8OA3.00** | Provision of written information about diabetes and driving |
| **9OLK.00** | DESMOND diabetes structured education programme completed |
| **C103y00** | Other specified diabetes mellitus with coma |
| **C10EN11** | Type I diabetes mellitus with ketoacidotic coma |
| **C10D.11** | Maturity onset diabetes in youth type 2 |
| **ZRB5.00** | Diabetes treatment satisfaction questionnaire |
| **C10F711** | Type II diabetes mellitus - poor control |
| **8I6F.00** | Diabetic retinopathy screening not indicated |
| **9NM0.00** | Attending diabetes clinic |
| **C109J00** | Insulin treated Type 2 diabetes mellitus |
| **C109K00** | Hyperosmolar non-ketotic state in type 2 diabetes mellitus |
| **8HVU.00** | Private referral to diabetologist |
| **C10N000** | Secondary diabetes mellitus without complication |
| **C10E400** | Unstable type 1 diabetes mellitus |
| **66Aq.00** | Diabetic foot screen |
| **C10ER00** | Latent autoimmune diabetes mellitus in adult |
| **C108811** | Type I diabetes mellitus - poor control |
| **9N1v.00** | Seen in diabetic eye clinic |
| **C109911** | Type II diabetes mellitus without complication |
| **9Oy0300** | Diabetic foot screening invitation second letter |
| **66AW.00** | Diabetic foot risk assessment |
| **66AG.00** | Diabetic drug side effects |
| **C104.11** | Diabetic nephropathy |
| **C108E11** | Type I diabetes mellitus with hypoglycaemic coma |
| **66AQ.00** | Diabetes: shared care programme |
| **9OL..00** | Diabetes monitoring admin. |
| **9OLJ.00** | DAFNE diabetes structured education programme completed |
| **C108.11** | IDDM-Insulin dependent diabetes mellitus |
| **66AX.00** | Diabetes: shared care in pregnancy - diabetol and obstet |
| **8I6G.00** | Diabetic foot examination not indicated |
| **250 H** | COMA DIABETIC |
| **8IEa.00** | Referral to DAFNE diabetes structured educn prog declined |
| **C109.11** | NIDDM - Non-insulin dependent diabetes mellitus |
| **8Hj0.00** | Referral to diabetes structured education programme |
| **C100011** | Insulin dependent diabetes mellitus |
| **9OL3.00** | Diabetes monitoring default |
| **2BBM.00** | O/E - diabetic maculopathy absent both eyes |
| **8HKE.00** | Diabetology D.V. requested |
| **8HME.00** | Listed for Diabetology admissn |
| **8HTE100** | Referral to community diabetes clinic |
| **C10N.00** | Secondary diabetes mellitus |
| **C109J11** | Insulin treated non-insulin dependent diabetes mellitus |
| **66AJ.11** | Unstable diabetes |
| **C109700** | Non-insulin dependent diabetes mellitus - poor control |
| **C108411** | Unstable type I diabetes mellitus |
| **13B1.00** | Diabetic diet |
| **C109D00** | Non-insulin dependent diabetes mellitus with hypoglyca coma |
| **C10A.00** | Malnutrition-related diabetes mellitus |
| **9N0o.00** | Seen in community diabetic specialist nurse clinic |
| **8A13.00** | Diabetic stabilisation |
| **C102z00** | Diabetes mellitus NOS with hyperosmolar coma |
| **8H7r.00** | Refer to diabetic foot screener |
| **C10P100** | Type II diabetes mellitus in remission |
| **C10P.00** | Diabetes mellitus in remission |
| **C101000** | Diabetes mellitus, juvenile type, with ketoacidosis |
| **9m06.00** | Excluded from diabetic retinopathy screening as deceased |
| **C10E912** | Insulin dependent diabetes maturity onset |
| **679L200** | Education about diabetes and driving |
| **8H3O.00** | Non-urgent diabetic admission |
| **U60231E** | [X] Adverse reaction to insulins and antidiabetic agents NOS |
| **C10E811** | Type I diabetes mellitus - poor control |
| **66Al.00** | Diabetic monitoring - higher risk albumin excretion |
| **66Ab.00** | Diabetic foot examination |
| **3881.00** | Education score - diabetes |
| **66At100** | Type II diabetic dietary review |
| **250 AK** | MATURITY ONSET DIABETES MELLITUS INSULIN |
| **679L.00** | Health education - diabetes |
| **C10E.00** | Type 1 diabetes mellitus |
| **250 JL** | KETOSIS DIABETIC |
| **2500AH** | LATENT DIABETES |
| **C108812** | Type 1 diabetes mellitus - poor control |
| **66AS000** | Diabetes Year of Care annual review |
| **C108E12** | Type 1 diabetes mellitus with hypoglycaemic coma |
| **250 AT** | DIABETIC AMYOTROPHY |
| **9OLF.00** | Diabetes structured education programme completed |
| **9M10.00** | Informed dissent for diabetes national audit |
| **8IE2.00** | Diabetes care plan declined |
| **C101.00** | Diabetes mellitus with ketoacidosis |
| **L180000** | Diabetes mellitus - unspec whether in pregnancy/puerperium |
| **9M00.00** | Informed consent for diabetes national audit |
| **9N4I.00** | DNA - Did not attend diabetic clinic |
| **C108.12** | Type 1 diabetes mellitus |
| **13L4.11** | Diabetic child |
| **68A9.00** | Diabetic retinopathy screening offered |
| **9OLA.11** | Diabetes monitored |
| **13Y1.00** | Diabetic association member |
| **250 E** | HYPOGLYCAEMIA IN DIABETES MELLITUS |
| **250 JK** | KETOACIDOSIS DIABETIC |
| **250 NH** | HYPEROSMOLAR DIABETIC STATE |
| **2G5G.00** | O/E - Right diabetic foot at high risk |
| **C100111** | Maturity onset diabetes |
| **66AS.00** | Diabetic annual review |
| **TJ23.00** | Adverse reaction to insulins and antidiabetic agents |
| **C10A000** | Malnutrition-related diabetes mellitus with coma |
| **250 HC** | HYPOGLYCAEMIC COMA DIABETIC |
| **C103000** | Diabetes mellitus, juvenile type, with ketoacidotic coma |
| **66AP.00** | Diabetes: practice programme |
| **9NiZ.00** | Did not attend diabetes foot screening |
| **C10EE12** | Insulin dependent diabetes mellitus with hypoglycaemic coma |
| **66At000** | Type I diabetic dietary review |
| **8CR2.00** | Diabetes clinical management plan |
| **9OLL.00** | XPERT diabetes structured education programme completed |
| **8Hj4.00** | Referral to DESMOND diabetes structured education programme |
| **66AH.00** | Diabetic treatment changed |
| **C109912** | Type 2 diabetes mellitus without complication |
| **C100112** | Non-insulin dependent diabetes mellitus |
| **66AQ100** | Declined consent for diabetes year of care programme |
| **C10EA12** | Insulin-dependent diabetes without complication |
| **9OL6.00** | Diabetes monitoring 3rd letter |
| **C10EA11** | Type I diabetes mellitus without complication |
| **C10FJ00** | Insulin treated Type 2 diabetes mellitus |
| **C108A11** | Type I diabetes mellitus without complication |
| **C350011** | Bronzed diabetes |
| **8I81.00** | Did not complete diabetes structured education programme |
| **9m0C.00** | Excluded frm diabetic retinopathy screen as terminal illness |
| **C10E412** | Unstable insulin dependent diabetes mellitus |
| **C101z00** | Diabetes mellitus NOS with ketoacidosis |
| **8I82.00** | Did not complete DAFNE diabetes structured education program |
| **C109D11** | Type II diabetes mellitus with hypoglycaemic coma |
| **66AJ.00** | Diabetic - poor control |
| **C108400** | Unstable insulin dependent diabetes mellitus |
| **9m05.00** | Excluded from diabetic retinopathy screening as moved away |
| **Q441.00** | Neonatal diabetes mellitus |
| **C10E911** | Type I diabetes mellitus maturity onset |
| **66At111** | Type 2 diabetic dietary review |
| **C10H.00** | Diabetes mellitus induced by non-steroid drugs |
| **ZRB4.11** | CSQ - Diabetes clinic satisfaction questionnaire |
| **C10F.00** | Type 2 diabetes mellitus |
| **C10FN11** | Type II diabetes mellitus with ketoacidosis |
| **9h4..00** | Exception reporting: diabetes quality indicators |
| **1IA..00** | No evidence of diabetic nephropathy |
| **C100000** | Diabetes mellitus, juvenile type, no mention of complication |
| **9N0m.00** | Seen in diabetic nurse consultant clinic |
| **9OL5.00** | Diabetes monitoring 2nd letter |
| **ZRbH.00** | Perceived control of insulin-dependent diabetes |
| **C10EA00** | Type 1 diabetes mellitus without complication |
| **8Hl1.00** | Referral for diabetic retinopathy screening |
| **66A4.00** | Diabetic on oral treatment |
| **C103.00** | Diabetes mellitus with ketoacidotic coma |
| **9N2d.00** | Seen by diabetologist |
| **66AA.11** | Injection sites - diabetic |
| **C10E.11** | Type I diabetes mellitus |
| **8HTe.00** | Referral to diabetes preconception counselling clinic |
| **C108A00** | Insulin-dependent diabetes without complication |
| **ZL62600** | Referral to diabetic liaison nurse |
| **66Au.00** | Diabetic erectile dysfunction review |
| **9b92000** | Diabetic medicine |
| **8CP2.00** | Transition of diabetes care options discussed |
| **C101100** | Diabetes mellitus, adult onset, with ketoacidosis |
| **C100z00** | Diabetes mellitus NOS with no mention of complication |
| **9OLN.00** | Diabetes monitor invitation by SMS (short message service) |
| **9OLM.00** | Diabetes structured education programme declined |
| **ZL22500** | Under care of diabetic liaison nurse |
| **2G5E.00** | O/E - Right diabetic foot at low risk |
| **C109D12** | Type 2 diabetes mellitus with hypoglycaemic coma |
| **8HgC.00** | Discharged from diabetes shared care programme |
| **9m08.00** | Excluded from diabetic retinopathy screening as blind |
| **C10E900** | Type 1 diabetes mellitus maturity onset |
| **68A7.00** | Diabetic retinopathy screening |
| **250 GA** | GANGRENE DIABETIC |
| **C11y000** | Steroid induced diabetes |
| **C108800** | Insulin dependent diabetes mellitus - poor control |
| **42c..00** | HbA1 - diabetic control |
| **66AL.00** | Diabetic-uncooperative patient |
| **C108.13** | Type I diabetes mellitus |
| **66AD.00** | Fundoscopy - diabetic check |
| **66An.00** | Diabetes type 1 review |
| **L180X00** | Pre-existing diabetes mellitus, unspecified |
| **9NiD.00** | Did not attend DESMOND diabetes structured education program |
| **8Hj5.00** | Referral to XPERT diabetes structured education programme |
| **C109.13** | Type II diabetes mellitus |
| **2BBJ.00** | O/E - no right diabetic retinopathy |
| **ZC2CA00** | Dietary advice for type II diabetes |
| **42W..00** | Hb. A1C - diabetic control |
| **C10yz00** | Diabetes mellitus NOS with other specified manifestation |
| **13AC.00** | Diabetic weight reducing diet |
| **C10y.00** | Diabetes mellitus with other specified manifestation |
| **8CA4100** | Pt advised re diabetic diet |
| **9m07.00** | Excluded diabetc retinop screen as under care ophthalmolgist |
| **8CMW700** | Diabetes clinical pathway |
| **9NiA.00** | Did not attend diabetes structured education programme |
| **66A8.00** | Has seen dietician - diabetes |
| **9OL7.00** | Diabetes monitor.verbal invite |
| **6761.00** | Diabetic pre-pregnancy counselling |
| **93C4.00** | Patient consent given for addition to diabetic register |
| **Cyu2000** | [X]Other specified diabetes mellitus |
| **C10..00** | Diabetes mellitus |
| **250 AD** | DIABETES MELLITUS INSULIN DEPENDANT |
| **8IEQ.00** | Referral to community diabetes specialist nurse declined |
| **66Ak.00** | Diabetic monitoring - lower risk albumin excretion |
| **9NN9.00** | Under care of diabetes specialist nurse |
| **679L211** | Advice about diabetes and driving |
| **8B3l.00** | Diabetes medication review |
| **2G5B.00** | O/E - Left diabetic foot at risk |
| **8I3X.00** | Diabetic retinopathy screening refused |
| **2G5e.00** | O/E - Right diabetic foot at increased risk |
| **C103z00** | Diabetes mellitus NOS with ketoacidotic coma |
| **C10y100** | Diabetes mellitus, adult, + other specified manifestation |
| **9m03.00** | Eligibility permanently inactive for diabetic retinop screen |
| **8I57.00** | Patient held diabetic record declined |
| **C100.00** | Diabetes mellitus with no mention of complication |
| **C102.00** | Diabetes mellitus with hyperosmolar coma |
| **66A3.00** | Diabetic on diet only |
| **661N400** | Diabetes self-management plan review |
| **C103100** | Diabetes mellitus, adult onset, with ketoacidotic coma |
| **66AR.00** | Diabetes management plan given |
| **C10G000** | Secondary pancreatic diabetes mellitus without complication |
| **9h41.00** | Excepted from diabetes qual indicators: Patient unsuitable |
| **8Hl4.00** | Referral to community diabetes specialist nurse |
| **C102100** | Diabetes mellitus, adult onset, with hyperosmolar coma |
| **C10FP00** | Type 2 diabetes mellitus with ketoacidotic coma |
| **9OLZ.00** | Diabetes monitoring admin.NOS |
| **67D8.00** | Provision of diabetes clinical summary |
| **C101y00** | Other specified diabetes mellitus with ketoacidosis |
| **C10C.00** | Diabetes mellitus autosomal dominant |
| **9OL..11** | Diabetes clinic administration |

# Diabetes, with end-organ damage (Elixhauser)

| **Read code** | **Description** |
| --- | --- |
| **C107.00** | Diabetes mellitus with peripheral circulatory disorder |
| **2G5C.00** | Foot abnormality - diabetes related |
| **C10F411** | Type II diabetes mellitus with ulcer |
| **C10E712** | Insulin dependent diabetes mellitus with retinopathy |
| **C10EC11** | Type I diabetes mellitus with polyneuropathy |
| **2BBW.00** | O/E - right eye diabetic maculopathy |
| **C10EQ11** | Type I diabetes mellitus with gastroparesis |
| **C109512** | Type 2 diabetes mellitus with gangrene |
| **C107200** | Diabetes mellitus, adult with gangrene |
| **C104100** | Diabetes mellitus, adult onset, with renal manifestation |
| **2BBo.00** | O/E - sight threatening diabetic retinopathy |
| **C108C11** | Type I diabetes mellitus with polyneuropathy |
| **C10FB11** | Type II diabetes mellitus with polyneuropathy |
| **C10EQ00** | Type 1 diabetes mellitus with gastroparesis |
| **C10zy00** | Other specified diabetes mellitus with unspecified comps |
| **C108F11** | Type I diabetes mellitus with diabetic cataract |
| **C108000** | Insulin-dependent diabetes mellitus with renal complications |
| **C108511** | Type I diabetes mellitus with ulcer |
| **2BBP.00** | O/E - right eye background diabetic retinopathy |
| **C10E111** | Type I diabetes mellitus with ophthalmic complications |
| **C109A11** | Type II diabetes mellitus with mononeuropathy |
| **F381300** | Myasthenic syndrome due to diabetic amyotrophy |
| **R054300** | [D]Widespread diabetic foot gangrene |
| **C10FE00** | Type 2 diabetes mellitus with diabetic cataract |
| **C109111** | Type II diabetes mellitus with ophthalmic complications |
| **C10E512** | Insulin dependent diabetes mellitus with ulcer |
| **F372.00** | Polyneuropathy in diabetes |
| **C109F11** | Type II diabetes mellitus with peripheral angiopathy |
| **C108311** | Type I diabetes mellitus with multiple complications |
| **C109012** | Type 2 diabetes mellitus with renal complications |
| **C10E612** | Insulin dependent diabetes mellitus with gangrene |
| **K27y700** | Erectile dysfunction due to diabetes mellitus |
| **C10FF11** | Type II diabetes mellitus with peripheral angiopathy |
| **C106.12** | Diabetes mellitus with neuropathy |
| **2BBF.00** | Retinal abnormality - diabetes related |
| **C10F400** | Type 2 diabetes mellitus with ulcer |
| **2BBQ.00** | O/E - left eye background diabetic retinopathy |
| **C10zz00** | Diabetes mellitus NOS with unspecified complication |
| **F420800** | High risk non proliferative diabetic retinopathy |
| **C314.11** | Renal diabetes |
| **8HBH.00** | Diabetic retinopathy 6 month review |
| **C107000** | Diabetes mellitus, juvenile +peripheral circulatory disorder |
| **C10F511** | Type II diabetes mellitus with gangrene |
| **C10FG11** | Type II diabetes mellitus with arthropathy |
| **C109C11** | Type II diabetes mellitus with nephropathy |
| **C104z00** | Diabetes mellitus with nephropathy NOS |
| **C10F011** | Type II diabetes mellitus with renal complications |
| **C109400** | Non-insulin dependent diabetes mellitus with ulcer |
| **C10EH00** | Type 1 diabetes mellitus with arthropathy |
| **2BBL.00** | O/E - diabetic maculopathy present both eyes |
| **C10F111** | Type II diabetes mellitus with ophthalmic complications |
| **C108z00** | Unspecified diabetes mellitus with multiple complications |
| **C10FL11** | Type II diabetes mellitus with persistent proteinuria |
| **C106.11** | Diabetic amyotrophy |
| **C109H12** | Type 2 diabetes mellitus with neuropathic arthropathy |
| **C108y00** | Other specified diabetes mellitus with multiple comps |
| **C10E711** | Type I diabetes mellitus with retinopathy |
| **C10E000** | Type 1 diabetes mellitus with renal complications |
| **C109B00** | Non-insulin dependent diabetes mellitus with polyneuropathy |
| **250 M** | CHARCOT'S DIABETIC ARTHROPATHY |
| **C109100** | Non-insulin-dependent diabetes mellitus with ophthalm comps |
| **C10E611** | Type I diabetes mellitus with gangrene |
| **C108J12** | Type 1 diabetes mellitus with neuropathic arthropathy |
| **C109A00** | Non-insulin dependent diabetes mellitus with mononeuropathy |
| **F3y0.00** | Diabetic mononeuropathy |
| **F372200** | Asymptomatic diabetic neuropathy |
| **C109312** | Type 2 diabetes mellitus with multiple complications |
| **8HBG.00** | Diabetic retinopathy 12 month review |
| **C10E511** | Type I diabetes mellitus with ulcer |
| **2G5W.00** | O/E - left chronic diabetic foot ulcer |
| **C106.13** | Diabetes mellitus with polyneuropathy |
| **F374z00** | Polyneuropathy in disease NOS |
| **250 LG** | DIABETIC GLOMERULOSCLEROSIS |
| **C107.12** | Diabetes with gangrene |
| **C10EK00** | Type 1 diabetes mellitus with persistent proteinuria |
| **C108J11** | Type I diabetes mellitus with neuropathic arthropathy |
| **C105z00** | Diabetes mellitus NOS with ophthalmic manifestation |
| **F420400** | Diabetic maculopathy |
| **C10EF00** | Type 1 diabetes mellitus with diabetic cataract |
| **C10z.00** | Diabetes mellitus with unspecified complication |
| **C105.00** | Diabetes mellitus with ophthalmic manifestation |
| **C109E00** | Non-insulin depend diabetes mellitus with diabetic cataract |
| **C109411** | Type II diabetes mellitus with ulcer |
| **C109G11** | Type II diabetes mellitus with arthropathy |
| **C109000** | Non-insulin-dependent diabetes mellitus with renal comps |
| **C108700** | Insulin dependent diabetes mellitus with retinopathy |
| **C10FR00** | Type 2 diabetes mellitus with gastroparesis |
| **C10EP11** | Type I diabetes mellitus with exudative maculopathy |
| **C10F500** | Type 2 diabetes mellitus with gangrene |
| **C10EC12** | Insulin dependent diabetes mellitus with polyneuropathy |
| **C10E012** | Insulin-dependent diabetes mellitus with renal complications |
| **F35z000** | Diabetic mononeuritis NOS |
| **C10F300** | Type 2 diabetes mellitus with multiple complications |
| **K01x100** | Nephrotic syndrome in diabetes mellitus |
| **F345000** | Diabetic mononeuritis multiplex |
| **C105100** | Diabetes mellitus, adult onset, + ophthalmic manifestation |
| **C109500** | Non-insulin dependent diabetes mellitus with gangrene |
| **F420300** | Advanced diabetic maculopathy |
| **K08yA11** | Clinical diabetic nephropathy |
| **F420000** | Background diabetic retinopathy |
| **C10E500** | Type 1 diabetes mellitus with ulcer |
| **C108100** | Insulin-dependent diabetes mellitus with ophthalmic comps |
| **C10E112** | Insulin-dependent diabetes mellitus with ophthalmic comps |
| **C108D11** | Type I diabetes mellitus with nephropathy |
| **C10EF12** | Insulin dependent diabetes mellitus with diabetic cataract |
| **C108200** | Insulin-dependent diabetes mellitus with neurological comps |
| **C10H000** | DM induced by non-steroid drugs without complication |
| **C109412** | Type 2 diabetes mellitus with ulcer |
| **C10FL00** | Type 2 diabetes mellitus with persistent proteinuria |
| **250 F** | NEUROPATHY DIABETIC |
| **C10E600** | Type 1 diabetes mellitus with gangrene |
| **C108500** | Insulin dependent diabetes mellitus with ulcer |
| **C107.11** | Diabetes mellitus with gangrene |
| **C10z100** | Diabetes mellitus, adult onset, + unspecified complication |
| **F420700** | High risk proliferative diabetic retinopathy |
| **C109C12** | Type 2 diabetes mellitus with nephropathy |
| **C10F211** | Type II diabetes mellitus with neurological complications |
| **C109612** | Type 2 diabetes mellitus with retinopathy |
| **C108112** | Type 1 diabetes mellitus with ophthalmic complications |
| **C10ED00** | Type 1 diabetes mellitus with nephropathy |
| **C10yy00** | Other specified diabetes mellitus with other spec comps |
| **C10FC11** | Type II diabetes mellitus with nephropathy |
| **2G5H.00** | O/E - Right diabetic foot - ulcerated |
| **C10z000** | Diabetes mellitus, juvenile type, + unspecified complication |
| **2BBR.00** | O/E - right eye preproliferative diabetic retinopathy |
| **C109F00** | Non-insulin-dependent d m with peripheral angiopath |
| **C10E700** | Type 1 diabetes mellitus with retinopathy |
| **M271200** | Mixed diabetic ulcer - foot |
| **C109C00** | Non-insulin dependent diabetes mellitus with nephropathy |
| **C108300** | Insulin dependent diabetes mellitus with multiple complicatn |
| **C106100** | Diabetes mellitus, adult onset, + neurological manifestation |
| **C108600** | Insulin dependent diabetes mellitus with gangrene |
| **C10F611** | Type II diabetes mellitus with retinopathy |
| **2BBT.00** | O/E - right eye proliferative diabetic retinopathy |
| **2G5V.00** | O/E - right chronic diabetic foot ulcer |
| **C107300** | IDDM with peripheral circulatory disorder |
| **C107100** | Diabetes mellitus, adult, + peripheral circulatory disorder |
| **C107400** | NIDDM with peripheral circulatory disorder |
| **F381311** | Diabetic amyotrophy |
| **C108D00** | Insulin dependent diabetes mellitus with nephropathy |
| **F372100** | Chronic painful diabetic neuropathy |
| **C104.00** | Diabetes mellitus with renal manifestation |
| **C109H00** | Non-insulin dependent d m with neuropathic arthropathy |
| **C10EL00** | Type 1 diabetes mellitus with persistent microalbuminuria |
| **2BBX.00** | O/E - left eye diabetic maculopathy |
| **2BBS.00** | O/E - left eye preproliferative diabetic retinopathy |
| **F440700** | Diabetic iritis |
| **C10E312** | Insulin dependent diabetes mellitus with multiple complicat |
| **C109H11** | Type II diabetes mellitus with neuropathic arthropathy |
| **C108011** | Type I diabetes mellitus with renal complications |
| **C10F200** | Type 2 diabetes mellitus with neurological complications |
| **G73y000** | Diabetic peripheral angiopathy |
| **C10E200** | Type 1 diabetes mellitus with neurological complications |
| **250 N** | DIABETIC NEPHROPATHY |
| **C108H00** | Insulin dependent diabetes mellitus with arthropathy |
| **C108712** | Type 1 diabetes mellitus with retinopathy |
| **2BBr.00** | Impaired vision due to diabetic retinopathy |
| **C10FC00** | Type 2 diabetes mellitus with nephropathy |
| **F372.11** | Diabetic polyneuropathy |
| **C109300** | Non-insulin-dependent diabetes mellitus with multiple comps |
| **C109611** | Type II diabetes mellitus with retinopathy |
| **C108211** | Type I diabetes mellitus with neurological complications |
| **66Av.00** | Diabetic assessment of erectile dysfunction |
| **F372.12** | Diabetic neuropathy |
| **C109E11** | Type II diabetes mellitus with diabetic cataract |
| **C109212** | Type 2 diabetes mellitus with neurological complications |
| **2G5L.00** | O/E - Left diabetic foot - ulcerated |
| **C106y00** | Other specified diabetes mellitus with neurological comps |
| **C109E12** | Type 2 diabetes mellitus with diabetic cataract |
| **C10M.00** | Lipoatrophic diabetes mellitus |
| **2BBl.00** | O/E - left eye stable treated prolif diabetic retinopathy |
| **F420200** | Preproliferative diabetic retinopathy |
| **C10EC00** | Type 1 diabetes mellitus with polyneuropathy |
| **C10EL11** | Type I diabetes mellitus with persistent microalbuminuria |
| **C10ED12** | Insulin dependent diabetes mellitus with nephropathy |
| **C106000** | Diabetes mellitus, juvenile, + neurological manifestation |
| **F420600** | Non proliferative diabetic retinopathy |
| **2BBk.00** | O/E - right eye stable treated prolif diabetic retinopathy |
| **C10FQ00** | Type 2 diabetes mellitus with exudative maculopathy |
| **Kyu0300** | [X]Glomerular disorders in diabetes mellitus |
| **14F4.00** | H/O: Admission in last year for diabetes foot problem |
| **F171100** | Autonomic neuropathy due to diabetes |
| **C105000** | Diabetes mellitus, juvenile type, + ophthalmic manifestation |
| **C10F311** | Type II diabetes mellitus with multiple complications |
| **N030000** | Diabetic cheiroarthropathy |
| **C109511** | Type II diabetes mellitus with gangrene |
| **R054200** | [D]Gangrene of toe in diabetic |
| **C10FG00** | Type 2 diabetes mellitus with arthropathy |
| **C108512** | Type 1 diabetes mellitus with ulcer |
| **F420.00** | Diabetic retinopathy |
| **C108F00** | Insulin dependent diabetes mellitus with diabetic cataract |
| **C109112** | Type 2 diabetes mellitus with ophthalmic complications |
| **C10EP00** | Type 1 diabetes mellitus with exudative maculopathy |
| **C10FA11** | Type II diabetes mellitus with mononeuropathy |
| **2BBV.00** | O/E - left eye proliferative diabetic retinopathy |
| **F464000** | Diabetic cataract |
| **C109G00** | Non-insulin dependent diabetes mellitus with arthropathy |
| **C10FH11** | Type II diabetes mellitus with neuropathic arthropathy |
| **C108B11** | Type I diabetes mellitus with mononeuropathy |
| **C108711** | Type I diabetes mellitus with retinopathy |
| **7276.00** | Pan retinal photocoagulation for diabetes |
| **C10E300** | Type 1 diabetes mellitus with multiple complications |
| **F372000** | Acute painful diabetic neuropathy |
| **C10F100** | Type 2 diabetes mellitus with ophthalmic complications |
| **C109200** | Non-insulin-dependent diabetes mellitus with neuro comps |
| **C108012** | Type 1 diabetes mellitus with renal complications |
| **C108B00** | Insulin dependent diabetes mellitus with mononeuropathy |
| **C10FF00** | Type 2 diabetes mellitus with peripheral angiopathy |
| **C10EG00** | Type 1 diabetes mellitus with peripheral angiopathy |
| **K08yA00** | Proteinuric diabetic nephropathy |
| **C10FM11** | Type II diabetes mellitus with persistent microalbuminuria |
| **C10FM00** | Type 2 diabetes mellitus with persistent microalbuminuria |
| **C109011** | Type II diabetes mellitus with renal complications |
| **C108G00** | Insulin dependent diab mell with peripheral angiopathy |
| **C10EB00** | Type 1 diabetes mellitus with mononeuropathy |
| **C10FB00** | Type 2 diabetes mellitus with polyneuropathy |
| **C109F12** | Type 2 diabetes mellitus with peripheral angiopathy |
| **C10FA00** | Type 2 diabetes mellitus with mononeuropathy |
| **F420500** | Advanced diabetic retinal disease |
| **C10FE11** | Type II diabetes mellitus with diabetic cataract |
| **F420z00** | Diabetic retinopathy NOS |
| **C108H11** | Type I diabetes mellitus with arthropathy |
| **C10F600** | Type 2 diabetes mellitus with retinopathy |
| **C109600** | Non-insulin-dependent diabetes mellitus with retinopathy |
| **250 LK** | KIMMELSTIEL- WILSON DISEASE/SYNDROME |
| **C10E212** | Insulin-dependent diabetes mellitus with neurological comps |
| **C107z00** | Diabetes mellitus NOS with peripheral circulatory disorder |
| **C104y00** | Other specified diabetes mellitus with renal complications |
| **C109211** | Type II diabetes mellitus with neurological complications |
| **M271000** | Ischaemic ulcer diabetic foot |
| **C10FH00** | Type 2 diabetes mellitus with neuropathic arthropathy |
| **C109G12** | Type 2 diabetes mellitus with arthropathy |
| **C109B11** | Type II diabetes mellitus with polyneuropathy |
| **C108C00** | Insulin dependent diabetes mellitus with polyneuropathy |
| **C106.00** | Diabetes mellitus with neurological manifestation |
| **C10E311** | Type I diabetes mellitus with multiple complications |
| **C10F000** | Type 2 diabetes mellitus with renal complications |
| **C10E100** | Type 1 diabetes mellitus with ophthalmic complications |
| **C10EJ00** | Type 1 diabetes mellitus with neuropathic arthropathy |
| **C105y00** | Other specified diabetes mellitus with ophthalmic complicatn |
| **F420100** | Proliferative diabetic retinopathy |
| **C10A500** | Malnutritn-relat diabetes melitus wth periph circul complctn |
| **N030100** | Diabetic Charcot arthropathy |
| **C108J00** | Insulin dependent diab mell with neuropathic arthropathy |
| **N030011** | Diabetic cheiropathy |
| **C104000** | Diabetes mellitus, juvenile type, with renal manifestation |
| **C108212** | Type 1 diabetes mellitus with neurological complications |
| **C106z00** | Diabetes mellitus NOS with neurological manifestation |

# Drug abuse (Elixhauser)

| **Read code** | **Description** |
| --- | --- |
| **U60A100** | [X]Opioid recept antag causing adverse effect in therap use |
| **1T0..00** | H/O heroin misuse |
| **8AA..00** | Drug abuse monitoring |
| **E246z00** | Glue sniffing dependence NOS |
| **1V65.00** | Heroin misuse |
| **Eu11211** | [X]Drug addiction - opioids |
| **E248300** | Combined opioid with other drug dependence in remission |
| **1TD2.00** | H/O infrequent opiate misuse |
| **13r3.00** | Abstinent from drug misuse on maintenance replacement |
| **E247.11** | Absinthe addiction |
| **E243200** | Cannabis dependence, episodic |
| **E240.00** | Opioid type drug dependence |
| **E246200** | Glue sniffing dependence, episodic |
| **E241.16** | Sedative dependence |
| **1V...00** | Drug misuse behaviour |
| **1T8..00** | H/O cannabis misuse |
| **E247300** | Other specified drug dependence in remission |
| **ZV11500** | [V]Personal history of drug abuse by injection |
| **E243.00** | Cannabis type drug dependence |
| **8BAX.00** | Drug dependence home detoxification |
| **Eu16300** | [X]Mental and behav dis due hallucinogens: withdrawal state |
| **E241.12** | Barbiturate dependence |
| **Eu55.15** | [X]Laxative habit |
| **13c8.00** | Reduced drugs misuse |
| **1V01.00** | Long-term drug misuser |
| **E255200** | Nondependent opioid abuse, episodic |
| **E021z00** | Drug-induced paranoia or hallucinatory state NOS |
| **E245200** | Hallucinogen dependence, episodic |
| **E248000** | Combined opioid with other drug dependence, unspecified |
| **E259400** | Misuse of prescription only drugs |
| **E253100** | Nondependent hallucinogen abuse, continuous |
| **1T32.00** | H/O infrequent benzodiazepine misuse |
| **Eu11300** | [X]Mental and behav dis due to use opioids: withdrawal state |
| **E245000** | Hallucinogen dependence, unspecified |
| **Eu11z00** | [X]Ment & behav dis due use opioids: unsp ment & behav dis |
| **L183z00** | Drug dependence during pregnancy/childbirth/puerperium NOS |
| **E258.00** | Nondependent antidepressant type drug abuse |
| **U200z00** | [X]Intent self poison nonopioid analgesic unspecif place |
| **E259.00** | Nondependent mixed drug abuse |
| **1V02.00** | Poly-drug misuser |
| **E02y000** | Drug-induced delirium |
| **E252100** | Nondependent cannabis abuse, continuous |
| **1T93.00** | Previous history of solvent misuse |
| **1T63.00** | Previous history of crack cocaine misuse |
| **9k5..00** | Drug misuse - enhanced services administration |
| **1TB..00** | H/O major tranquilliser misuse |
| **1T4..00** | H/O amphetamine misuse |
| **E253z00** | Nondependent hallucinogen abuse NOS |
| **E021100** | Drug-induced hallucinosis |
| **E252.00** | Nondependent cannabis abuse |
| **E245100** | Hallucinogen dependence, continuous |
| **E021.00** | Drug-induced paranoia or hallucinatory states |
| **E249z00** | Combined drug dependence, excluding opioid, NOS |
| **9k51.11** | Shared care drug misuse treatment |
| **8BAc.00** | Substance misuse management stopped - self withdrawal |
| **Eu12211** | [X]Drug addiction - cannabis |
| **E241.13** | Benzodiazepine dependence |
| **1TD1.00** | H/O weekly opiate misuse |
| **E245z00** | Hallucinogen dependence NOS |
| **1T22.00** | H/O infrequent ecstasy misuse |
| **ZV6D700** | [V]Drug abuse counselling and surveillance |
| **1TC..00** | H/O anti-depressant misuse |
| **8I2N.00** | Drug dependence home detoxification contraindicated |
| **E24..00** | Drug dependence |
| **E248200** | Combined opioid with other drug dependence, episodic |
| **8H7x.00** | Referral to drug abuse counsellor |
| **E249200** | Combined drug dependence, excluding opioid, episodic |
| **1T21.00** | H/O weekly ecstasy misuse |
| **E242z00** | Cocaine drug dependence NOS |
| **E25y000** | Nondependent other drug abuse, unspecified |
| **1TC3.00** | Previous history of anti-depressant misuse |
| **1T81.00** | H/O weekly cannabis misuse |
| **1T01.00** | H/O weekly heroin misuse |
| **L183.00** | Drug dependence in pregnancy, childbirth and the puerperium |
| **9HC5.00** | Substance misuse treatment programme completed |
| **8IE7.00** | Substance misuse assessment declined |
| **Eu11100** | [X]Mental and behav dis due to use of opioids: harmful use |
| **1T1..00** | H/O methadone misuse |
| **E248.00** | Combined opioid with other drug dependence |
| **E247100** | Other specified drug dependence, continuous |
| **1T6..00** | H/O crack cocaine misuse |
| **E247200** | Other specified drug dependence, episodic |
| **1V23.00** | Time since stopped drug misuse |
| **E252000** | Nondependent cannabis abuse, unspecified |
| **1T91.00** | H/O weekly solvent misuse |
| **1T50.00** | H/O daily cocaine misuse |
| **E253000** | Nondependent hallucinogen abuse, unspecified |
| **1T83.00** | Previous history of cannabis misuse |
| **E249300** | Combined drug dependence, excluding opioid, in remission |
| **E25y100** | Nondependent other drug abuse, continuous |
| **1T92.00** | H/O infrequent solvent misuse |
| **13cB.00** | Misuses drugs orally |
| **E24A.00** | Ecstasy type drug dependence |
| **1V2..00** | Frequency of drug misuse |
| **8BAo.00** | Benzodiazepine dependence detoxification |
| **E249100** | Combined drug dependence, excluding opioid, continuous |
| **E252300** | Nondependent cannabis abuse in remission |
| **9No5.00** | Seen in substance misuse clinic |
| **E240000** | Unspecified opioid dependence |
| **1T73.00** | Previous history of hallucinogen misuse |
| **1T10.00** | H/O daily methadone misuse |
| **E25yz00** | Nondependent other drug abuse NOS |
| **Eu11.00** | [X]Mental and behavioural disorders due to use of opioids |
| **E247000** | Other specified drug dependence, unspecified |
| **E246100** | Glue sniffing dependence, continuous |
| **Eu11700** | [X]Men & beh dis due opioids: resid & late-onset psychot dis |
| **E249.00** | Combined drug dependence, excluding opioids |
| **E247.00** | Other specified drug dependence |
| **E240200** | Episodic opioid dependence |
| **1T13.00** | Previous history of methadone misuse |
| **U406y00** | [X]Pois/exp ?intent to hallucinogen other spec place |
| **Eu13211** | [X]Drug addiction- sedative / hypnotics |
| **E247z00** | Other specified drug dependence NOS |
| **1T61.00** | H/O weekly crack cocaine misuse |
| **1T40.00** | H/O daily amphetamine misuse |
| **E243100** | Cannabis dependence, continuous |
| **8B2S.00** | Opioid agonist substitution therapy |
| **E25y.00** | Nondependent other drug abuse |
| **8B2T.00** | Opioid antagonist therapy |
| **E253.00** | Nondependent hallucinogen abuse |
| **E245.00** | Hallucinogen dependence |
| **E259200** | Nondependent mixed drug abuse, episodic |
| **1TD3.00** | Previous history of opiate misuse |
| **9s...00** | Drug misuse clinic administration |
| **13cH.00** | Persistent substance misuse |
| **1T7..00** | H/O hallucinogen misuse |
| **1TB0.00** | H/O daily major tranquilliser misuse |
| **E25y200** | Nondependent other drug abuse, episodic |
| **E240z00** | Opioid drug dependence NOS |
| **E255z00** | Nondependent opioid abuse NOS |
| **Eu11500** | [X]Mental & behav dis due to use opioids: psychotic disorder |
| **8BAW.00** | Drug dependence self detoxification |
| **1V26.00** | Misused drugs in past |
| **13c5.00** | Substance misuse increased |
| **9HC6.00** | Substance misuse treatment declined |
| **1V22.00** | Age at starting drug misuse |
| **E259z00** | Nondependent mixed drug abuse NOS |
| **8Hh1.00** | Self referral to substance misuse service |
| **146F.00** | H/O: drug abuse |
| **E258z00** | Nondependent antidepressant type drug abuse NOS |
| **1T41.00** | H/O weekly amphetamine misuse |
| **E253300** | Nondependent hallucinogen abuse in remission |
| **9k53.00** | Pharmacy attended for drug misuse - enhanced services admin |
| **Eu16z00** | [X]Ment/behav dis due use hallucinogens: unsp ment/behav dis |
| **Eu16100** | [X]Mental and behav dis due to use hallucinogens: harmfl use |
| **E253200** | Nondependent hallucinogen abuse, episodic |
| **1T62.00** | H/O infrequent crack cocaine misuse |
| **E02y300** | Drug-induced depressive state |
| **E25y.12** | Laxative abuse |
| **E021000** | Drug-induced paranoid state |
| **1T43.00** | Previous history of amphetamine misuse |
| **8B23.13** | Drug dependence therapy |
| **13r4.00** | Abstinent from drug misuse when receiving blocking therapy |
| **E246.00** | Glue sniffing dependence |
| **9HC4.00** | Substance misuse treatment withdrawn |
| **1T00.00** | H/O daily heroin misuse |
| **13cF.00** | Preoccupied with substance misuse |
| **1V66.00** | Ecstasy misuse |
| **9HC3.00** | Substance misuse clinical management plan reviewed |
| **Eu11y00** | [X]Men & behav dis due to use opioids: oth men & behav dis |
| **1V0..00** | Misuses drugs |
| **9N1yJ00** | Seen in drug misuse clinic |
| **1T3..00** | H/O benzodiazepine misuse |
| **E240100** | Continuous opioid dependence |
| **Eu16.00** | [X]Mental and behavioural disorders due to use hallucinogens |
| **Z416.00** | Substance abuse counselling |
| **1T...00** | History of substance misuse |
| **1T9..00** | H/O solvent misuse |
| **1T11.00** | H/O weekly methadone misuse |
| **E255100** | Nondependent opioid abuse, continuous |
| **9HC..00** | Substance misuse monitoring |
| **1T31.00** | H/O weekly benzodiazepine misuse |
| **E240300** | Opioid dependence in remission |
| **E246300** | Glue sniffing dependence in remission |
| **E259100** | Nondependent mixed drug abuse, continuous |
| **1T52.00** | H/O infrequent cocaine misuse |
| **1T51.00** | H/O weekly cocaine misuse |
| **E243z00** | Cannabis drug dependence NOS |
| **E255300** | Nondependent opioid abuse in remission |
| **67H3.00** | Lifestyle advice regarding drug misuse |
| **1T23.00** | Previous history of ecstasy misuse |
| **1T53.00** | Previous history of cocaine misuse |
| **E249000** | Combined drug dependence, excluding opioid, unspecified |
| **Eu16200** | [X]Mental and behav dis due to hallucinogens: dependence syn |
| **Eu11000** | [X]Mental & behav dis due to use opioids: acute intoxication |
| **9HC2.00** | Substance misuse clinical management plan agreed |
| **1V0E.00** | Health problem secondary to drug misuse |
| **1TC0.00** | H/O daily anti-depressant misuse |
| **9k52.00** | Drug misuse treatment primary care - enhanced services admin |
| **1T2..00** | H/O ecstasy misuse |
| **E243000** | Cannabis dependence, unspecified |
| **L183100** | Drug dependence during pregnancy - baby delivered |
| **9k51.00** | Shared care drug misuse treatment - enhanced services admin |
| **E255000** | Nondependent opioid abuse, unspecified |
| **E243300** | Cannabis dependence in remission |
| **L183300** | Drug dependence during pregnancy - baby not yet delivered |
| **E242.00** | Cocaine type drug dependence |
| **1TD0.00** | H/O daily opiate misuse |
| **1T12.00** | H/O infrequent methadone misuse |
| **1T80.00** | H/O daily cannabis misuse |
| **E252200** | Nondependent cannabis abuse, episodic |
| **E02y400** | Drug-induced personality disorder |
| **E25y300** | Nondependent other drug abuse in remission |
| **9N4i.00** | DNA - Did not attend substance misuse clinic |
| **1TA..00** | H/O barbiturate misuse |
| **13cM.00** | Substance misuse |
| **E248z00** | Combined opioid with other drug dependence NOS |
| **1T02.00** | Previous history of heroin misuse |
| **E245300** | Hallucinogen dependence in remission |
| **E248100** | Combined opioid with other drug dependence, continuous |
| **9NdN.00** | Declined consent for notification of drug misuse |
| **9kS..00** | Drug misuse assessment declined - enhanced services administ |
| **1T82.00** | H/O infrequent cannabis misuse |
| **Eu55.00** | [X]Abuse of non-dependence-producing substances |
| **9K4..00** | SMR25a drug misuse initial assessment form |
| **Eu11200** | [X]Mental and behav dis due to use opioids: dependence syndr |
| **13c6.00** | Substance misuse decreased |
| **8Hq..00** | Admission to substance misuse detoxification centre |
| **E24z.00** | Drug dependence NOS |
| **E255.00** | Nondependent opioid abuse |
| **1T03.00** | H/O infrequent heroin misuse |
| **1T33.00** | Previous history of benzodiazepine misuse |
| **E259000** | Nondependent mixed drug abuse, unspecified |
| **1T60.00** | H/O daily crack cocaine misuse |
| **13cE.00** | Prolonged high dose use of cannabis |
| **9k50.00** | Drug misuse - enhanced service completed |
| **E246000** | Glue sniffing dependence, unspecified |
| **9k1..00** | Alcohol misuse - enhanced services administration |
| **1T42.00** | H/O infrequent amphetamine misuse |
| **E259300** | Nondependent mixed drug abuse in remission |
| **L183.11** | Pregnancy and drug dependence |
| **E25z.00** | Misuse of drugs NOS |
| **1T20.00** | H/O daily ecstasy misuse |
| **Eu16211** | [X]Drug addiction - hallucinogen |
| **E252z00** | Nondependent cannabis abuse NOS |
| **ZV11400** | [V]Personal history of psychoactive substance abuse |
| **8HkF.00** | Referral to substance misuse service |
| **1T30.00** | H/O daily benzodiazepine misuse |
| **677T.00** | Substance misuse structured counselling |
| **Eu16000** | [X]Mental & behav dis due hallucinogens: acute intoxicatn |
| **Eu11400** | [X]Men & behav dis due opioid: withdrawl state with delirium |
| **1T90.00** | H/O daily solvent misuse |
| **9k12.00** | Alcohol misuse - enhanced service completed |
| **1TD..00** | H/O opiate misuse |
| **1T5..00** | H/O cocaine misuse |
| **ZRl..00** | Substance use disorder diagnostic schedule |

# Fluid and electrolyte disorders (Elixhauser)

| **Read code** | **Description** |
| --- | --- |
| **C360.00** | Hyperosmolality and or hypernatraemia |
| **C362y00** | Other specified acidosis |
| **C354000** | Hypocalcaemia NEC |
| **C352z00** | Disorder of magnesium metabolism NOS |
| **C368.00** | Hypokalaemia |
| **44I4200** | Low serum potassium level |
| **C36A.00** | Respiratory acidosis and metabolic alkalosis |
| **C36z000** | Hyperchloraemia |
| **C354z00** | Disorder of calcium metabolism NOS |
| **C354900** | Calcium deficiency |
| **C36z100** | Hypochloraemia |
| **C366111** | Idiopathic fluid retention |
| **C354500** | Familial benign hypercalcaemia |
| **C294400** | Magnesium deficiency |
| **C362100** | Respiratory acidosis |
| **44I8100** | Raised serum calcium level |
| **C363200** | Metabolic alkalaemia |
| **C36..11** | Electrolyte disorders |
| **C365.00** | Volume depletion |
| **C363y00** | Other specified alkalosis |
| **C363z00** | Alkalosis NOS |
| **C363.00** | Alkalosis |
| **C365z00** | Volume depletion NOS |
| **C363500** | Alkalaemia |
| **C36z.00** | Disorders of fluid, electrolyte and acid-base balance NEC |
| **C362900** | Compensated metabolic acidosis |
| **C362200** | Lactic acidosis |
| **C365100** | Hypovolaemia |
| **46M2.00** | Urine electrolytes abnormal |
| **C36..00** | Disorders of fluid, electrolyte and acid-base balance |
| **C354200** | Idiopathic hypercalcaemia |
| **C363000** | Metabolic alkalosis |
| **C352.00** | Disorders of magnesium metabolism |
| **C361.11** | Hyponatraemia |
| **C363100** | Respiratory alkalosis |
| **C361.00** | Hyposmolality and or hyponatraemia |
| **C367.00** | Hyperkalaemia |
| **C360.11** | Hypernatraemia |
| **C354111** | Secondary hypercalcaemia |
| **C36zz00** | Electrolyte imbalance NOS |
| **C354100** | Hypercalcaemia NEC |
| **C362000** | Metabolic acidosis NEC |
| **8B77.00** | Potassium supplementation |
| **44I4100** | Raised serum potassium level |
| **44L2100** | Blood magnesium abnormal |

# HIV AIDS (Elixhauser)

| **Read code** | **Description** |
| --- | --- |
| **L179.00** | HIV disease complicating pregnancy childbirth puerperium |
| **43h9.00** | HIV proviral deoxyribonucleic acid polymerase chain reaction |
| **66j0.00** | Human immunodeficiency virus annual review |
| **A788500** | Human immunodeficiency virus with secondary infection |
| **AyuCD00** | [X]Unspecified human immunodeficiency virus [HIV] disease |
| **A789400** | HIV disease resulting in multiple infections |
| **43j7.00** | HIV 1 nucleic acid detection |
| **AyuC100** | [X]HIV disease resulting in other viral infections |
| **A789X00** | HIV dis reslt/oth mal neopl/lymph,h'matopoetc+reltd tissu |
| **A788.00** | Acquired immune deficiency syndrome |
| **A788y00** | Human immunodeficiency virus with other clinical findings |
| **65VE.00** | Notification of AIDS |
| **A788000** | Acute human immunodeficiency virus infection |
| **ZV01A00** | [V]Asymptomatic human immunodeficency virus infection status |
| **AyuC500** | [X]HIV disease resulting/unspcf infectious??? disease |
| **A789700** | HIV dis resulting oth types of non-Hodgkin's lymphoma |
| **AyuCC00** | [X]HIV disease resulting in other specified conditions |
| **A789.00** | Human immunodef virus resulting in other disease |
| **L7990A** | ACQUIRED IMMUNE DEFICIENCY SYNDROME |
| **A788.11** | Human immunodeficiency virus infection |
| **9kl..00** | HIV pos gen health check serv declind - enhanc service admin |
| **AyuC900** | [X]HIV disease resulting in unspecified malignant neoplasm |
| **65QA.00** | AIDS carrier |
| **A789000** | HIV disease resulting in mycobacterial infection |
| **A788z00** | Acquired human immunodeficiency virus infection syndrome NOS |
| **A788U00** | HIV disease result/haematological+immunologic abnorms,NEC |
| **A788V00** | HIV disease resulting in multiple diseases CE |
| **9Nt1000** | Seen by community HIV (human immunodeficiency virus) nurse |
| **AyuC600** | [X]HIV disease resulting in other non-Hodgkin's lymphoma |
| **AyuC.00** | [X]Human immunodeficiency virus disease |
| **A789200** | HIV disease resulting in candidiasis |
| **66j..00** | Human immunodeficiency virus monitoring |
| **8Hle.00** | Referral to community HIV nurse specialist |
| **A789300** | HIV disease resulting in Pneumocystis carinii pneumonia |
| **A789511** | HIV disease resulting in Kaposi sarcoma |
| **4J3F.00** | Human immunodeficiency virus viral load by log rank |
| **AyuC200** | [X]HIV disease resulting in other mycoses |
| **AyuCA00** | [X]HIV disease resulting in multiple diseases CE |
| **A789100** | HIV disease resulting in cytomegaloviral disease |
| **A788400** | Human immunodeficiency virus with neurological disease |
| **AyuC700** | [X]HIV dis reslt/oth mal neopl/lymph,h'matopoetc? tissu |
| **A789311** | HIV disease resulting in Pneumocystis jirovecii pneumonia |
| **799MD** | AIDS |
| **AyuC800** | [X]HIV disease resulting in other malignant neoplasms |
| **A788200** | HIV infection with persistent generalised lymphadenopathy |
| **A789A00** | HIV disease resulting in wasting syndrome |
| **AyuCB00** | [X]HIV disease result/haematological+immunologic abnorms,NEC |
| **A788300** | Human immunodeficiency virus with constitutional disease |
| **43WK.00** | Human immunodeficiency virus antibody level |
| **AyuC400** | [X]HIV disease resulting/other infectious+parasitic diseases |
| **43C3.11** | HIV positive |
| **AyuC000** | [X]HIV disease resulting in other bacterial infections |
| **43w3.00** | Human immunodeficiency virus RNA/DNA ratio |
| **A788100** | Asymptomatic human immunodeficiency virus infection |
| **A788X00** | HIV disease resulting/unspcf infectious+parasitic disease |
| **A789500** | HIV disease resulting in Kaposi's sarcoma |
| **4J34.00** | HIV viral load |
| **AyuC300** | [X]HIV disease resulting in multiple infections |
| **A788600** | Human immunodeficiency virus with secondary cancers |
| **R109.00** | [D]Laboratory evidence of human immunodeficiency virus [HIV] |

# Hypertension, uncomplicated (Elixhauser)

| **Read code** | **Description** |
| --- | --- |
| **67H8.00** | Lifestyle advice regarding hypertension |
| **662P000** | Hypertension 9 month review |
| **662c.00** | Hypertension six month review |
| **G2y..00** | Other specified hypertensive disease |
| **G20z.11** | Hypertension NOS |
| **662q.00** | Trial reduction of antihypertensive therapy |
| **G24z000** | Secondary renovascular hypertension NOS |
| **9OI..00** | Hypertension monitoring admin. |
| **G20..00** | Essential hypertension |
| **8I3N.00** | Hypertension treatment refused |
| **G2...00** | Hypertensive disease |
| **9N1y200** | Seen in hypertension clinic |
| **G24z.00** | Secondary hypertension NOS |
| **662..12** | Hypertension monitoring |
| **G240.00** | Secondary malignant hypertension |
| **8CR4.00** | Hypertension clinical management plan |
| **14A2.00** | H/O: hypertension |
| **Gyu2.00** | [X]Hypertensive diseases |
| **G20..12** | Primary hypertension |
| **G24z100** | Hypertension secondary to drug |
| **Gyu2100** | [X]Hypertension secondary to other renal disorders |
| **661M600** | Hypertension self-management plan agreed |
| **662b.00** | Moderate hypertension control |
| **G240000** | Secondary malignant renovascular hypertension |
| **G24..00** | Secondary hypertension |
| **Gyu2000** | [X]Other secondary hypertension |
| **G24zz00** | Secondary hypertension NOS |
| **6629.00** | Hypertension:follow-up default |
| **G28..00** | Stage 2 hypertension (NICE - Nat Ins for Hth Clin Excl 2011) |
| **8B26.00** | Antihypertensive therapy |
| **662d.00** | Hypertension annual review |
| **662O.00** | On treatment for hypertension |
| **U60C51A** | [X] Adverse reaction to antihypertensives NOS |
| **G201.00** | Benign essential hypertension |
| **U60C511** | [X] Adverse reaction to other antihypertensives |
| **8IA5.00** | Trial withdrawal of antihypertensive therapy declined |
| **7Q01y00** | Other specified high cost hypertension drugs |
| **662F.00** | Hypertension treatm. started |
| **G241000** | Secondary benign renovascular hypertension |
| **G203.00** | Diastolic hypertension |
| **G2z..00** | Hypertensive disease NOS |
| **G240z00** | Secondary malignant hypertension NOS |
| **G27..00** | Hypertension resistant to drug therapy |
| **6627.00** | Good hypertension control |
| **G200.00** | Malignant essential hypertension |
| **G25..00** | Stage 1 hypertension (NICE - Nat Ins for Hth Clin Excl 2011) |
| **9OI..11** | Hypertension clinic admin. |
| **8HT5.00** | Referral to hypertension clinic |
| **G244.00** | Hypertension secondary to endocrine disorders |
| **662G.00** | Hypertensive treatm.changed |
| **662P.00** | Hypertension monitoring |
| **G241z00** | Secondary benign hypertension NOS |
| **8BL0.00** | Patient on maximal tolerated antihypertensive therapy |
| **G2...11** | BP - hypertensive disease |
| **6146200** | Hypertension induced by oral contraceptive pill |
| **G202.00** | Systolic hypertension |
| **G26..11** | Severe hypertension |
| **6628.00** | Poor hypertension control |
| **G26..00** | Severe hypertension (Nat Inst for Health Clinical Ex 2011) |
| **G20z.00** | Essential hypertension NOS |
| **G241.00** | Secondary benign hypertension |
| **G25..11** | Stage 1 hypertension |
| **9OIA.11** | Hypertension monitored |

# Hypertension with end-organ damage (Elixhauser)

| **Read code** | **Description** |
| --- | --- |
| **G230.00** | Malignant hypertensive heart and renal disease |
| **G211.00** | Benign hypertensive heart disease |
| **G21z.00** | Hypertensive heart disease NOS |
| **G21zz00** | Hypertensive heart disease NOS |
| **G21..00** | Hypertensive heart disease |
| **G211z00** | Benign hypertensive heart disease NOS |
| **F421300** | Hypertensive retinopathy |
| **G210100** | Malignant hypertensive heart disease with CCF |
| **F404200** | Blind hypertensive eye |
| **G21z000** | Hypertensive heart disease NOS without CCF |
| **G210000** | Malignant hypertensive heart disease without CCF |
| **G23z.00** | Hypertensive heart and renal disease NOS |
| **G23..00** | Hypertensive heart and renal disease |
| **G22z.00** | Hypertensive renal disease NOS |
| **G211100** | Benign hypertensive heart disease with CCF |
| **G672.11** | Hypertensive crisis |
| **G234.00** | Hyperten heart&renal dis+both(congestv)heart and renal fail |
| **G21z011** | Cardiomegaly - hypertensive |
| **G21z100** | Hypertensive heart disease NOS with CCF |
| **G210.00** | Malignant hypertensive heart disease |
| **G232.00** | Hypertensive heart&renal dis wth (congestive) heart failure |
| **G221.00** | Benign hypertensive renal disease |
| **G220.00** | Malignant hypertensive renal disease |
| **G231.00** | Benign hypertensive heart and renal disease |
| **G210z00** | Malignant hypertensive heart disease NOS |
| **G222.00** | Hypertensive renal disease with renal failure |

# Hypothyroidism (Elixhauser)

| **Read code** | **Description** |
| --- | --- |
| **C041.00** | Other postablative hypothyroidism |
| **C040.00** | Postsurgical hypothyroidism |
| **C042.00** | Iodine hypothyroidism |
| **C04..12** | Thyroid deficiency |
| **C03..00** | Congenital hypothyroidism |
| **C04..11** | Myxoedema |
| **C03z.11** | Congenital thyroid insufficiency |
| **9Oj4.00** | Hypothyroidism monitoring telephone invitation |
| **Q433700** | Neonatal jaundice with congenital hypothyroidism |
| **C047.00** | Subclinical hypothyroidism |
| **9Oj3.00** | Hypothyroidism monitoring verbal invite |
| **C044.00** | Postinfectious hypothyroidism |
| **9Oj0.00** | Hypothyroidism monitoring first letter |
| **C04..00** | Acquired hypothyroidism |
| **1432.00** | H/O: hypothyroidism |
| **66BB.00** | Hypothyroidism annual review |
| **9Oj1.00** | Hypothyroidism monitoring second letter |
| **C046.00** | Autoimmune myxoedema |
| **Cyu1100** | [X]Other sp cified hypothyroidism |
| **F395300** | Myopathy due to myxoedema |
| **9Oj2.00** | Hypothyroidism monitoring third letter |
| **C040.11** | Post ablative hypothyroidism |
| **C04z100** | Myxoedema coma |
| **C04z.11** | Pretibial myxoedema - hypothyroid |
| **C04..13** | Hypothyroidism |
| **C043100** | Hypothyroidism resulting from phenylbutazone |
| **C03..11** | Cretinism |
| **C04z000** | Premature puberty due to hypothyroidism |
| **C043.00** | Other iatrogenic hypothyroidism |
| **C045.00** | Acquired atrophy of thyroid |
| **C041z00** | Postablative hypothyroidism NOS |
| **C04z.12** | Thyroid insufficiency |
| **C043200** | Hypothyroidism resulting from resorcinol |
| **C03y000** | Congenital hypothyroidism with diffuse goitre |
| **C030.00** | Pendred's syndrome |
| **C03z.00** | Congenital hypothyroidism NOS |
| **C04y.00** | Other acquired hypothyroidism |
| **C04z.00** | Hypothyroidism NOS |
| **C031.00** | Goitrous cretin |
| **9Oj..00** | Hypothyroidism monitoring administration |
| **C043z00** | Iatrogenic hypothyroidism NOS |
| **C04z.13** | Hypothyroid goitre, acquired |
| **C03y100** | Congenital hypothyroidism without goitre |
| **C03y.00** | Other specified congenital hypothyroidism |
| **F381400** | Myasthenic syndrome due to hypothyroidism |
| **C0A5.00** | Subclinical iodine-deficiency hypothyroidism |
| **8CR5.00** | Hypothyroidism clinical management plan |
| **C043000** | Hypothyroidism resulting from para-aminosalicylic acid |
| **C041000** | Irradiation hypothyroidism |
| **C03z.12** | Cretinism |

# Insomnia

| **Read code** | **Description** |
| --- | --- |
| **1BX9.00** | Light sleep |
| **1BX3.00** | early morning waking |
| **1BX0.00** | Delayed onset of sleep |
| **E274111** | Insomnia NOS |
| **R005.11** | [D]Insomnia - symptom |
| **1B1B.00** | Cannot sleep - insomnia |
| **E274D11** | Restless sleep |
| **1B1B.11** | C/O - insomnia |
| **E274.12** | Insomnia due to nonorganic sleep disorder |
| **1B1B200** | Late insomnia |
| **1B1B000** | Initial insomnia |
| **E274E00** | 'Short-sleeper' |
| **E274100** | Transient insomnia |
| **Fy00.00** | Disorders of maintaining and initiating sleep |
| **R005200** | [D]Insomnia NOS |
| **1B1B100** | Middle insomnia |
| **Eu51000** | [X]Nonorganic insomnia |
| **E274200** | Persistent insomnia |

# Liver disease (Elixhauser)

| **Read code** | **Description** |
| --- | --- |
| **ZV02612** | [V]Hepatitis Australia antigen carrier |
| **4JQ3.00** | Hepatitis C virus genotype |
| **4JQD.11** | Hepatitis C PCR positive |
| **ZV02C00** | [V]Hepatitis C carrier |
| **A702.00** | Viral hepatitis B with coma |
| **J631.00** | Hepatitis in viral diseases EC |
| **A705100** | Acute delta-(super)infection of hepatitis B carrier |
| **7609.00** | Open operations on oesophageal varices |
| **C310400** | Glycogenosis with hepatic cirrhosis |
| **J620300** | Liver abscess via umbilicus |
| **J612.11** | Florid cirrhosis |
| **J633000** | Toxic hepatitis |
| **760F300** | Rigid oesophagoscopic injection sclerotherapy oesoph varices |
| **A707.00** | Chronic viral hepatitis |
| **ZV7C000** | [V]Assessment for liver transplant |
| **J615812** | Indian childhood cirrhosis |
| **A705200** | Acute hepatitis E |
| **J61y300** | Portal fibrosis without cirrhosis |
| **G852100** | Oesophageal varices without bleeding in diseases EC |
| **J600011** | Acute liver failure |
| **9Op1.00** | Hepatitis C screening offered |
| **J62y.00** | Other sequelae of chronic liver disease |
| **J631z00** | Hepatitis in viral diseases EC NOS |
| **J632.00** | Hepatitis in other infectious diseases EC |
| **Gyu9400** | [X]Oesophageal varices in diseases classified elsewhere |
| **A704000** | Viral hepatitis C with coma |
| **J63X.00** | Granulomatous hepatitis, not elsewhere classified |
| **J631500** | Hepatitis in other viral disease |
| **9kZ..00** | Hepatitis B screening positive - enhanced services admin |
| **ZV42700** | [V]Liver transplanted |
| **SP08600** | Liver transplant failure and rejection |
| **AE23.00** | Sequelae of viral hepatitis |
| **Q409000** | Congenital hepatitis A infection |
| **J631600** | Hepatitis + adenovirus |
| **7L1f.00** | Compensation for liver failure |
| **7609y00** | Other specified open operation on oesophageal varices |
| **141F.00** | History of viral hepatitis |
| **J615D00** | Bacterial portal cirrhosis |
| **141E.00** | History of hepatitis B |
| **A72x000** | Mumps hepatitis |
| **J635400** | Toxic liver disease with chronic lobular hepatitis |
| **J614y00** | Chronic hepatitis unspecified |
| **J633z00** | Hepatitis unspecified NOS |
| **J661700** | Primary sclerosing cholangitis |
| **67P4200** | Discussion about liver transplantation |
| **760C300** | Fibreoptic endoscopic injection sclerotherapy oesoph varices |
| **7800111** | Auxillary liver transplant |
| **43jW.00** | Hepatitis E nucleic acid detection |
| **7609400** | Open injection sclerotherapy to oesophageal varices |
| **J616000** | Primary biliary cirrhosis |
| **A70B.00** | Hepatitis C genotype 2 |
| **A053.00** | Amoebic liver abscess |
| **A70D.00** | Hepatitis C genotype 4 |
| **A707300** | Chronic viral hepatitis B |
| **G852z00** | Oesophageal varices in diseases EC NOS |
| **J620000** | Liver abscess due to portal pyaemia |
| **J635300** | Toxic liver disease with chronic persistent hepatitis |
| **ZC2CH11** | Dietary advice for liver disease |
| **TB00200** | Liver transplant with complication, without blame |
| **J61yz00** | Other non-alcoholic chronic liver disease NOS |
| **J615300** | Diffuse nodular cirrhosis |
| **J615500** | Hypertrophic portal cirrhosis |
| **G858.00** | Oesophageal varices NOS |
| **J615z11** | Macronodular cirrhosis of liver |
| **J61y800** | Nonalcoholic steatohepatitis |
| **J614300** | Recurrent hepatitis |
| **A70C.00** | Hepatitis C genotype 3 |
| **J614.00** | Chronic hepatitis |
| **J62..00** | Liver abscess and sequelae of chronic liver disease |
| **AyuB000** | [X]Other specified acute viral hepatitis |
| **J631400** | Hepatitis in yellow fever |
| **A705400** | Hepatitis non A non B |
| **J661800** | Secondary sclerosing cholangitis |
| **A707100** | Chronic viral hepatitis B without delta-agent |
| **Q409100** | Congenital hepatitis B infection |
| **J615z12** | Cryptogenic cirrhosis of liver |
| **J632200** | Hepatitis in secondary syphilis |
| **9kZ..11** | Hepatitis B screening positive |
| **A705.00** | Other specified viral hepatitis without coma |
| **J661900** | Sclerosing cholangitis unspecified |
| **A70z.00** | Unspecified viral hepatitis |
| **J620.00** | Liver abscess - excluding amoebic liver abscess |
| **A709.00** | Viral hepatitis without hepatic coma |
| **AyuJ900** | [X]Sequelae of viral hepatitis |
| **ZV02600** | [V]Viral hepatitis carrier |
| **J616z00** | Biliary cirrhosis NOS |
| **AyuB100** | [X]Other chronic viral hepatitis |
| **7Q05300** | RSV treatment and Hepatitis C treatment drugs Band 1 |
| **9NgR.00** | On hepatitis C treatment plan |
| **J625.11** | [X] Liver failure |
| **43k0.00** | Hepatitis B e antigen level |
| **J615700** | Cardiac portal cirrhosis |
| **J622.00** | Hepatic coma |
| **C370800** | Cystic fibrosis related cirrhosis |
| **J620200** | Liver abscess via hepatic artery |
| **J614z00** | Chronic hepatitis NOS |
| **J614200** | Chronic aggressive hepatitis |
| **4JQF.00** | Hepatitis C antigen positive |
| **J632z00** | Hepatitis in infectious diseases EC NOS |
| **J612.12** | Laennec's cirrhosis |
| **BB5D411** | [M]Hepatocellular adenoma |
| **J620100** | Liver abscess due to cholangitis |
| **J614100** | Chronic active hepatitis |
| **43j5.00** | Hepatitis C nucleic acid detection |
| **G852300** | Oesophageal varices in alcoholic cirrhosis of the liver |
| **Jyu7100** | [X]Other and unspecified cirrhosis of liver |
| **Jyu7200** | [X]Other specified inflammatory liver diseases |
| **A98yy13** | Gonococcal perihepatitis |
| **9kV..11** | Hepatitis C screening positive |
| **760F400** | Rigid oesophagoscopic banding of oesophageal varices |
| **J615G00** | Zooparasitic portal cirrhosis |
| **14C5.00** | H/O: liver disease |
| **7805211** | Exploration of liver transplant |
| **A70z100** | Acute viral hepatitis NOS |
| **43jG.00** | Hepatitis B nucleic acid detection |
| **760C500** | Fibreoptic endoscopic banding of oesophageal varices |
| **J617.00** | Alcoholic hepatitis |
| **A916100** | Secondary syphilitic hepatitis |
| **PB62.00** | Congenital cystic liver disease |
| **43jJ.00** | Hepatitis D nucleic acid detection |
| **SP14211** | Liver failure as a complication of care |
| **J616100** | Secondary biliary cirrhosis |
| **A701.11** | Infective hepatitis |
| **AyuB.00** | [X]Viral hepatitis |
| **J601100** | Subacute hepatitis - noninfective |
| **ZV02B00** | [V]Hepatitis B carrier |
| **43M2.00** | Hepatitis A test positive |
| **65Q7.00** | Viral hepatitis carrier |
| **C376100** | Alpha-1-antitrypsin hepatitis |
| **A70z000** | Hepatitis C |
| **J615C00** | Xanthomatous portal cirrhosis |
| **PB62000** | Congenital polycystic liver disease |
| **A785200** | Cytomegaloviral hepatitis |
| **J631000** | Hepatitis in coxsackie virus |
| **Q409z00** | Congenital viral hepatitis NOS |
| **A70G.00** | Acute hepatitis C |
| **J624.00** | Hepatorenal syndrome |
| **A704z00** | Other specified viral hepatitis with hepatic coma NOS |
| **Q409.00** | Congenital viral hepatitis |
| **A707X00** | Chronic viral hepatitis, unspecified |
| **J63y100** | Nonspecific reactive hepatitis |
| **G85..11** | Oesophageal varices |
| **J600100** | Acute hepatitis - noninfective |
| **J61..00** | Cirrhosis and chronic liver disease |
| **J635100** | Toxic liver disease with hepatic necrosis |
| **65V3.11** | Hepatitis notification |
| **J61z.00** | Chronic liver disease NOS |
| **J615z00** | Non-alcoholic cirrhosis NOS |
| **J615z13** | Cirrhosis of liver NOS |
| **Jyu7600** | [X]Toxic liver disease, unspecified |
| **G852.00** | Oesophageal varices in diseases EC |
| **2J23.00** | Hepatitis A - current infection |
| **A707200** | Chronic viral hepatitis C |
| **A705z00** | Other specified viral hepatitis without mention of coma NOS |
| **J635X00** | Toxic liver disease, unspecified |
| **AyuB200** | [X]Chronic viral hepatitis, unspecified |
| **AD05.00** | Toxoplasma hepatitis |
| **A700.00** | Viral hepatitis A with coma |
| **7609z00** | Open operation on oesophageal varices NOS |
| **7800200** | Replacement of previous liver transplant |
| **J635600** | Toxic liver disease with fibrosis and cirrhosis of liver |
| **J615711** | Congestive cirrhosis |
| **J633.00** | Hepatitis unspecified |
| **PB62100** | Fibrocystic liver disease |
| **SP14300** | Hepatorenal syndrome as a complication of care |
| **14i..00** | H/O hepatitis C antiviral drug therapy |
| **4JQD.00** | Hepatitis C viral ribonucleic acid PCR positive |
| **J615H00** | Infectious cirrhosis NOS |
| **A703.00** | Viral (serum) hepatitis B |
| **8LH..00** | Liver transplant planned |
| **G852000** | Oesophageal varices with bleeding in diseases EC |
| **C350012** | Pigmentary cirrhosis of liver |
| **J616.00** | Biliary cirrhosis |
| **J614000** | Chronic persistent hepatitis |
| **J631200** | Hepatitis in infectious mononucleosis |
| **PB62z00** | Congenital cystic liver disease NOS |
| **J62y.12** | Liver failure NOS |
| **G851.00** | Oesophageal varices without bleeding |
| **A98yy11** | Gonococcal hepatitis |
| **7Q05200** | Hepatitis B treatment drugs Band 1 |
| **J635.00** | Toxic liver disease |
| **J635200** | Toxic liver disease with acute hepatitis |
| **43B5.00** | Hepatitis e antigen present |
| **J616200** | Biliary cirrhosis of children |
| **A705000** | Viral hepatitis C without mention of hepatic coma |
| **A70A.00** | Hepatitis C genotype 1 |
| **A701.00** | Viral (infectious) hepatitis A |
| **2J2..00** | Hepatitis A status |
| **9kV..00** | Hepatitis C screening positive - enhanced services admin |
| **J615y00** | Portal cirrhosis unspecified |
| **J63B.00** | Autoimmune hepatitis |
| **J615100** | Multilobular portal cirrhosis |
| **9kX..00** | Hepatitis status 6 months post treatment - enhanced serv adm |
| **A707000** | Chronic viral hepatitis B with delta-agent |
| **J615.00** | Cirrhosis - non alcoholic |
| **J615800** | Juvenile portal cirrhosis |
| **J614400** | Chronic lobular hepatitis |
| **J635500** | Toxic liver disease with chronic active hepatitis |
| **J615400** | Fatty portal cirrhosis |
| **J620z00** | Liver abscess NOS |
| **J62z.00** | Liver abscess and chronic liver disease causing sequelae NOS |
| **9kR..00** | Chronic hepatitis annual review - enhanced services admin |
| **Q434100** | Giant cell hepatitis causing neonatal jaundice |
| **J632000** | Hepatitis in malaria |
| **7800112** | Piggy back liver transplant |
| **Jyu7700** | [X]Granulomatous hepatitis, not elsewhere classified |
| **43B4.00** | Hepatitis B surface antig +ve |
| **J61y.00** | Other non-alcoholic chronic liver disease |
| **J614111** | Autoimmune chronic active hepatitis |
| **A704.00** | Other specified viral hepatitis with coma |
| **Q434000** | Perinatal hepatitis causing jaundice, unspecified |
| **J615.11** | Portal cirrhosis |
| **L176500** | Viral hepatitis comp pregnancy, childbirth & the puerperium |
| **7609300** | Local ligation of oesophageal varices |
| **J615600** | Capsular portal cirrhosis |
| **J632300** | Hepatitis in toxoplasmosis |
| **7L1fz00** | Compensation for liver failure NOS |
| **J631100** | Hepatitis in cytomegalic inclusion virus |
| **A70..00** | Viral hepatitis |
| **G850.00** | Oesophageal varices with bleeding |
| **43w7.00** | Hepatitis A nucleic acid detection |
| **J623.00** | Portal hypertension |
| **G852200** | Oesophageal varices in cirrhosis of the liver |
| **J635000** | Toxic liver disease with cholestasis |

# Lymphoma (Elixhauser)

| **Read code** | **Description** |
| --- | --- |
| **B620800** | Nodular lymphoma of lymph nodes of multiple sites |
| **BBm1.11** | [M]Malignant reticulosis |
| **BBg4.00** | [M]Malignant lymphoma, stem cell type |
| **BBr2700** | [M]Adult T-cell leukaemia/lymphoma |
| **B620200** | Nodular lymphoma of intrathoracic lymph nodes |
| **B62x000** | T-zone lymphoma |
| **B62x400** | Malignant reticulosis |
| **BBgV.00** | [M]Malignant lymphoma, small cell, noncleaved, diffuse |
| **BBj6000** | [M]Hodgkin,s disease, nodular sclerosis, lymphocytic predom |
| **BBgA.00** | [M]Malignant lymphoma, centroblastic-centrocytic, diffuse |
| **ByuD300** | [X]Other specified types of non-Hodgkin's lymphoma |
| **B620500** | Nodular lymphoma of lymph nodes of inguinal region and leg |
| **B613300** | Hodgkin's, lymphocytic-histiocytic pred intra-abdominal node |
| **BBgP.00** | [M]Malignant lymphoma, mixed small and large cell, diffuse |
| **B600300** | Reticulosarcoma of intra-abdominal lymph nodes |
| **B627100** | Follicular non-Hodg mixed sml cleavd & lge cell lymphoma |
| **4M20.00** | Lymphoma stage I |
| **B627X00** | Diffuse non-Hodgkin's lymphoma, unspecified |
| **BBgH.00** | [M]Prolymphocytic lymphosarcoma |
| **B601500** | Lymphosarcoma of lymph nodes of inguinal region and leg |
| **B620z00** | Nodular lymphoma NOS |
| **B616z00** | Hodgkin's disease, lymphocytic depletion NOS |
| **BBp2.00** | [M]Malignant mastocytosis |
| **B617.00** | Nodular lymphocyte predominant Hodgkin lymphoma |
| **B67y000** | Lymphosarcoma cell leukaemia |
| **B621500** | Mycosis fungoides of lymph nodes of inguinal region and leg |
| **BBn0.11** | [M]Multiple myeloma |
| **B62y100** | Malignant lymphoma NOS of lymph nodes of head, face and neck |
| **B621400** | Mycosis fungoides of lymph nodes of axilla and upper limb |
| **B6...11** | Malignant neoplasm of histiocytic tissue |
| **B620.00** | Nodular lymphoma (Brill - Symmers disease) |
| **B616800** | Hodgkin's lymphocytic depletion lymph nodes multiple sites |
| **B628200** | Follicular lymphoma grade 3 |
| **B630.12** | Myelomatosis |
| **BBj1100** | [M]Hodgkin,s disease, lymphocytic predominance, nodular |
| **B613000** | Hodgkin's, lymphocytic-histiocytic predominance unspec site |
| **B612400** | Hodgkin's sarcoma of lymph nodes of axilla and upper limb |
| **B614000** | Hodgkin's disease, nodular sclerosis of unspecified site |
| **ByuDF00** | [X]Non-Hodgkin's lymphoma, unspecified type |
| **B61z100** | Hodgkin's disease NOS of lymph nodes of head, face and neck |
| **BBj1.00** | [M]Hodgkin's disease, lymphocytic predominance |
| **B620100** | Nodular lymphoma of lymph nodes of head, face and neck |
| **B627E00** | Diffuse large B-cell lymphoma |
| **B602.00** | Burkitt's lymphoma |
| **B624.11** | Leukaemic reticuloendotheliosis |
| **B627500** | Diffuse non-Hodgkin mixed sml & lge cell (diffuse) lymphoma |
| **B621000** | Mycosis fungoides of unspecified site |
| **B62z500** | Unspec malig neop lymphoid/histiocytic nodes inguinal/leg |
| **B619.00** | Mixed cellularity classical Hodgkin lymphoma |
| **BBgJ.00** | [M]Malignant lymphoma, centroblastic type NOS |
| **BBgG.00** | [M]Malignant lymphoma, lymphocytic, poorly different NOS |
| **BBm3.00** | [M]Letterer - Siwe disease |
| **B64y500** | Adult T-cell lymphoma/leukaemia (HTLV-1-associated) |
| **BBk0.00** | [M]Malignant lymphoma, nodular NOS |
| **B61z500** | Hodgkin's disease NOS of lymph nodes inguinal region and leg |
| **B628500** | Diffuse follicle centre lymphoma |
| **BBg1000** | [M]Malignant lymphoma, diffuse NOS |
| **B623.00** | Malignant histiocytosis |
| **B62y800** | Malignant lymphoma NOS of lymph nodes of multiple sites |
| **ByuDE00** | [X]Unspecified B-cell non-Hodgkin's lymphoma |
| **B61z000** | Hodgkin's disease NOS, unspecified site |
| **BBgM.00** | [M]Malignant lymphoma, small cleaved cell, diffuse |
| **BBk0.12** | [M]Follicular lymphosarcoma NOS |
| **B621800** | Mycosis fungoides of lymph nodes of multiple sites |
| **B60..00** | Lymphosarcoma and reticulosarcoma |
| **B614300** | Hodgkin's nodular sclerosis of intra-abdominal lymph nodes |
| **B627000** | Follicular non-Hodgkin's small cleaved cell lymphoma |
| **B628.00** | Follicular lymphoma |
| **B600700** | Reticulosarcoma of spleen |
| **BBm4.00** | [M]True histiocytic lymphoma |
| **BBlz.00** | [M]Mycosis fungoides NOS |
| **BBgN.00** | [M]Malign lymphoma,lymphocytic,intermediate differn, diffuse |
| **B630.00** | Multiple myeloma |
| **B602100** | Burkitt's lymphoma of lymph nodes of head, face and neck |
| **BBg0.00** | [M]Lymphomatous tumour, benign |
| **BBmH.00** | [M] Large cell lymphoma |
| **B62z600** | Unspec malig neop lymphoid/histiocytic of intrapelvic nodes |
| **B61z400** | Hodgkin's disease NOS of lymph nodes of axilla and arm |
| **B62y000** | Malignant lymphoma NOS of unspecified site |
| **BBgC.00** | [M]Malignant lymphoma, lymphocytic, well differentiated NOS |
| **B62D.00** | Histiocytic sarcoma |
| **ByuDF11** | [X]Non-Hodgkin's lymphoma NOS |
| **BBm..00** | [M]Miscellaneous reticuloendothelial neoplasms |
| **C333000** | Waldenstrom's macroglobulinaemia |
| **B611100** | Hodgkin's granuloma of lymph nodes of head, face and neck |
| **B613200** | Hodgkin's, lymphocytic-histiocytic pred intrathoracic nodes |
| **B614z00** | Hodgkin's disease, nodular sclerosis NOS |
| **B613z00** | Hodgkin's, lymphocytic-histiocytic predominance NOS |
| **B615z00** | Hodgkin's disease, mixed cellularity NOS |
| **BBn0.13** | [M]Myelomatosis |
| **B613700** | Hodgkin's, lymphocytic-histiocytic predominance of spleen |
| **B62E500** | Hepatosplenic T-cell lymphoma |
| **BBj..00** | [M]Hodgkin's disease |
| **B601800** | Lymphosarcoma of lymph nodes of multiple sites |
| **BBp0.00** | [M]Mastocytoma NOS |
| **ByuDC00** | [X]Diffuse non-Hodgkin's lymphoma, unspecified |
| **BBm1.00** | [M]Malignant histiocytosis |
| **B600000** | Reticulosarcoma of unspecified site |
| **B62F100** | Mantle cell lymphoma |
| **BBj7.00** | [M]Hodgkin's disease, nodular sclerosis, cellular phase |
| **BBj6100** | [M]Hodgkin,s disease, nodular sclerosis, mixed cellularity |
| **B611.00** | Hodgkin's granuloma |
| **B627B00** | Other types of follicular non-Hodgkin's lymphoma |
| **B628700** | Other types of follicular lymphoma |
| **BBm2.00** | [M]Histiocytic medullary reticulosis |
| **B61z700** | Hodgkin's disease NOS of spleen |
| **B62z200** | Unspec malig neop lymphoid/histiocytic of intrathoracic node |
| **B616400** | Hodgkin's lymphocytic depletion lymph nodes axilla and arm |
| **B601000** | Lymphosarcoma of unspecified site |
| **BBk3.00** | [M]Malig lymphoma, lymphocytic, well differentiated,nodular |
| **BBh..00** | [M]Reticulosarcomas |
| **B62z.00** | Malignant neoplasms of lymphoid and histiocytic tissue NOS |
| **4M22.00** | Lymphoma stage III |
| **BBgT.00** | [M]Malignant lymphoma, large cell, noncleaved, diffuse |
| **B601100** | Lymphosarcoma of lymph nodes of head, face and neck |
| **B62x100** | Lymphoepithelioid lymphoma |
| **B627200** | Follicular non-Hodgkin's large cell lymphoma |
| **B61zz00** | Hodgkin's disease NOS |
| **B62A.00** | Sarcoma of dendritic cells |
| **B935.11** | Histiocytic tumour NOS |
| **BBl0.00** | [M]Mycosis fungoides |
| **B600z00** | Reticulosarcoma NOS |
| **BBn2.00** | [M]Plasmacytoma NOS |
| **BBk7.00** | [M]Malignant lymphoma, centroblastic type, follicular |
| **BBgD.00** | [M]Malig lymphoma, lymphocytic, intermediate different NOS |
| **B62F.00** | Nonfollicular lymphoma |
| **B610100** | Hodgkin's paragranuloma of lymph nodes of head, face, neck |
| **B60y.00** | Other specified reticulosarcoma or lymphosarcoma |
| **BBg2.11** | [M]Non Hodgkins lymphoma |
| **C333.00** | Macroglobulinaemia |
| **B601.00** | Lymphosarcoma |
| **BBj6200** | [M]Hodgkin,s disease, nodular sclerosis, lymphocytic deplet |
| **B623z00** | Malignant histiocytosis NOS |
| **B62y.00** | Malignant lymphoma NOS |
| **B613100** | Hodgkin's, lymphocytic-histiocytic pred of head, face, neck |
| **B61z.11** | Hodgkin lymphoma NOS |
| **B62E200** | Anaplastic large cell lymphoma, ALK-negative |
| **B614.00** | Hodgkin's disease, nodular sclerosis |
| **B61z.00** | Hodgkin's disease NOS |
| **BBv0.00** | [M]Monocytoid B-cell lymphoma |
| **B624z00** | Leukaemic reticuloendotheliosis NOS |
| **B600.00** | Reticulosarcoma |
| **B62Ew00** | Other mature T/NK-cell lymphoma |
| **B614100** | Hodgkin's nodular sclerosis of head, face and neck |
| **B62E100** | Anaplastic large cell lymphoma, ALK-positive |
| **BBh0.00** | [M]Reticulosarcoma NOS |
| **BBg7.00** | [M]Malignant lymphoma, lymphoplasmacytoid type |
| **B62E300** | Cutaneous T-cell lymphoma |
| **B613600** | Hodgkin's, lymphocytic-histiocytic pred intrapelvic nodes |
| **BBgz.00** | [M]Lymphoma, diffuse or NOS |
| **BBg8.00** | [M]Malignant lymphoma, immunoblastic type |
| **B66..11** | Histiocytic leukaemia |
| **B601300** | Lymphosarcoma of intra-abdominal lymph nodes |
| **B627A00** | Diffuse non-Hodgkin's large cell lymphoma |
| **B62x.00** | Malignant lymphoma otherwise specified |
| **B616000** | Hodgkin's lymphocytic depletion of unspecified site |
| **B62x600** | True histiocytic lymphoma |
| **B62E800** | Blastic NK-cell lymphoma |
| **B630400** | Solitary plasmacytoma |
| **B62z100** | Unspec malig neop lymphoid/histiocytic lymph node head/neck |
| **B62z800** | Unspec malig neop lymphoid/histiocytic of multiple sites |
| **BBB1.00** | [M]Adenolymphoma |
| **BBn1.11** | [M]Plasmacytoma, benign |
| **B63..00** | Multiple myeloma and immunoproliferative neoplasms |
| **B627700** | Diffuse non-Hodgkin's lymphoblastic (diffuse) lymphoma |
| **B61z800** | Hodgkin's disease NOS of lymph nodes of multiple sites |
| **B602300** | Burkitt's lymphoma of intra-abdominal lymph nodes |
| **B621z00** | Mycosis fungoides NOS |
| **BBk0.11** | [M]Brill - Symmers' disease |
| **BBg6.00** | [M]Lymphosarcoma NOS |
| **BBg3.00** | [M]Malignant lymphoma, undifferentiated cell type NOS |
| **B62F200** | Lymphoblastic (diffuse) lymphoma |
| **BBj4.00** | [M]Hodgkin's disease,lymphocytic depletion,diffuse fibrosis |
| **BBj6.00** | [M]Hodgkin's disease, nodular sclerosis NOS |
| **BBl..00** | [M]Mycosis fungoides |
| **B627900** | Mucosa-associated lymphoma |
| **B62E600** | Enteropathy-associated T-cell lymphoma |
| **B628100** | Follicular lymphoma grade 2 |
| **B62E700** | Subcutaneous panniculitic T-cell lymphoma |
| **B628000** | Follicular lymphoma grade 1 |
| **B62y300** | Malignant lymphoma NOS of intra-abdominal lymph nodes |
| **BBk2.00** | [M]Malignant lymphoma, centroblastic-centrocytic, follicular |
| **B622z00** | Sezary's disease NOS |
| **B612.00** | Hodgkin's sarcoma |
| **B627600** | Diffuse non-Hodgkin's immunoblastic (diffuse) lymphoma |
| **B623100** | Malignant histiocytosis of lymph nodes head, face and neck |
| **B62y200** | Malignant lymphoma NOS of intrathoracic lymph nodes |
| **B627800** | Diffuse non-Hodgkin's lymphoma undifferentiated (diffuse) |
| **B621.00** | Mycosis fungoides |
| **B614200** | Hodgkin's nodular sclerosis of intrathoracic lymph nodes |
| **B601700** | Lymphosarcoma of spleen |
| **B61B.00** | Lymphocyte-rich classical Hodgkin lymphoma |
| **BBgS.00** | [M]Malignant lymphoma, large cell, cleaved, diffuse |
| **D401100** | Familial erythrophagocytic lymph histiocytosis |
| **B62F.11** | Non-follicular lymphoma |
| **B616.00** | Hodgkin's disease, lymphocytic depletion |
| **B613.00** | Hodgkin's disease, lymphocytic-histiocytic predominance |
| **BBg..00** | [M]Lymphomas, NOS or diffuse |
| **BBh2.00** | [M]Reticulosarcoma, nodular |
| **BBj9.00** | [M]Hodgkin's granuloma |
| **BBp1.00** | [M]Mast cell sarcoma |
| **B625000** | Letterer-Siwe disease of unspecified sites |
| **B623000** | Malignant histiocytosis of unspecified site |
| **B613800** | Hodgkin's, lymphocytic-histiocytic pred of multiple sites |
| **BBj0.00** | [M]Hodgkin's disease NOS |
| **B615500** | Hodgkin's mixed cellularity of lymph nodes inguinal and leg |
| **B62z400** | Unspec malig neop lymphoid/histiocytic lymph node axilla/arm |
| **B627G00** | Mediastinal (thymic) large B-cell lymphoma |
| **ByuD000** | [X]Other Hodgkin's disease |
| **B615000** | Hodgkin's disease, mixed cellularity of unspecified site |
| **B625z00** | Letterer-Siwe disease NOS |
| **BBjz.00** | [M]Hodgkin's disease NOS |
| **C37y700** | Histiocytosis, unspecified |
| **B628600** | Cutaneous follicle centre lymphoma |
| **C333011** | Waldenstrom macroglobulinaemia |
| **B625800** | Letterer-Siwe disease of lymph nodes of multiple sites |
| **B627400** | Diffuse non-Hodgkin's small cleaved cell (diffuse) lymphoma |
| **B627.11** | Non-Hodgkin lymphoma |
| **B627C00** | Follicular non-Hodgkin's lymphoma |
| **B628400** | Follicular lymphoma grade 3b |
| **C333z00** | Macroglobulinaemia NOS |
| **B62xX00** | Oth and unspecif peripheral & cutaneous T-cell lymphomas |
| **BBgG.11** | [M]Lymphoblastic lymphosarcoma NOS |
| **BBg5.00** | [M]Malignant lymphoma, convoluted cell type NOS |
| **B61..11** | Hodgkin lymphoma |
| **BBgL.00** | [M]Malignant lymphoma, small lymphocytic NOS |
| **B614400** | Hodgkin's nodular sclerosis of lymph nodes of axilla and arm |
| **BBgC.12** | [M]Lymphocytic lymphosarcoma NOS |
| **B620300** | Nodular lymphoma of intra-abdominal lymph nodes |
| **B62y700** | Malignant lymphoma NOS of spleen |
| **B614800** | Hodgkin's nodular sclerosis of lymph nodes of multiple sites |
| **B621300** | Mycosis fungoides of intra-abdominal lymph nodes |
| **B630.11** | Kahler's disease |
| **BBj1000** | [M]Hodgkin,s disease, lymphocytic predominance, diffuse |
| **B601200** | Lymphosarcoma of intrathoracic lymph nodes |
| **B625.00** | Letterer-Siwe disease |
| **B62y600** | Malignant lymphoma NOS of intrapelvic lymph nodes |
| **BBgC.11** | [M]Lymphocytic lymphoma NOS |
| **B62E900** | Angioimmunoblastic T-cell lymphoma |
| **B630200** | Plasmacytoma NOS |
| **BBgR.00** | [M]Malignant lymphoma, large cell, diffuse NOS |
| **BBg2.00** | [M]Malignant lymphoma, non Hodgkin's type |
| **B624000** | Leukaemic reticuloendotheliosis of unspecified sites |
| **4M2..00** | Lymphoma staging system |
| **B60z.00** | Reticulosarcoma or lymphosarcoma NOS |
| **BBk0.13** | [M]Giant follicular lymphoma |
| **B616700** | Hodgkin's disease, lymphocytic depletion of spleen |
| **BBgE.00** | [M]Malignant lymphoma, centrocytic |
| **B62yz00** | Malignant lymphoma NOS |
| **B615200** | Hodgkin's mixed cellularity of intrathoracic lymph nodes |
| **B62E.00** | T/NK-cell lymphoma |
| **B61C.00** | Other classical Hodgkin lymphoma |
| **BBv2.00** | [M]AngiocentricT-cell lymphoma |
| **ZV10711** | [V]Personal history of Hodgkin's disease |
| **B62x200** | Peripheral T-cell lymphoma |
| **B61..00** | Hodgkin's disease |
| **ByuD100** | [X]Other types of follicular non-Hodgkin's lymphoma |
| **N330900** | Osteoporosis in multiple myelomatosis |
| **C37y800** | Haemophagocytic lymphohistiocytosis |
| **B62zz00** | Lymphoid and histiocytic malignancy NOS |
| **B600100** | Reticulosarcoma of lymph nodes of head, face and neck |
| **B62y500** | Malignant lymphoma NOS of lymph node inguinal region and leg |
| **B62..00** | Other malignant neoplasm of lymphoid and histiocytic tissue |
| **B615.00** | Hodgkin's disease, mixed cellularity |
| **B627C11** | Follicular lymphoma NOS |
| **B628300** | Follicular lymphoma grade 3a |
| **BBgG.13** | [M]Lymphoblastoma NOS |
| **BBk..00** | [M]Lymphomas, nodular or follicular |
| **B624.00** | Leukaemic reticuloendotheliosis |
| **B62y400** | Malignant lymphoma NOS of lymph nodes of axilla and arm |
| **BBgK.00** | [M]Malig lymphoma, follicular centre cell, non-cleaved NOS |
| **B61z200** | Hodgkin's disease NOS of intrathoracic lymph nodes |
| **B62z000** | Unspec malig neop lymphoid/histiocytic of unspecified site |
| **B620000** | Nodular lymphoma of unspecified site |
| **BBm9.00** | [M] Monocytoid B-cell lymphoma |
| **B601z00** | Lymphosarcoma NOS |
| **B62F000** | Small cell B-cell lymphoma |
| **B627300** | Diffuse non-Hodgkin's small cell (diffuse) lymphoma |
| **C37yB00** | Langerhans' cell histiocytosis |
| **4M21.00** | Lymphoma stage II |
| **B613500** | Hodgkin's, lymphocytic-histiocytic pred inguinal and leg |
| **B627W00** | Unspecified B-cell non-Hodgkin's lymphoma |
| **BBkz.00** | [M]Lymphoma, nodular or follicular NOS |
| **B627.00** | Non - Hodgkin's lymphoma |
| **BBl1.00** | [M]Sezary's disease |
| **BBgB.00** | [M]Malignant lymphoma, follicular centre cell NOS |
| **B623300** | Malignant histiocytosis of intra-abdominal lymph nodes |
| **4M23.00** | Lymphoma stage IV |
| **B618.00** | Nodular sclerosis classical Hodgkin lymphoma |
| **B615100** | Hodgkin's mixed cellularity of lymph nodes head, face, neck |
| **B614700** | Hodgkin's disease, nodular sclerosis of spleen |
| **B61z300** | Hodgkin's disease NOS of intra-abdominal lymph nodes |
| **BBmD.00** | [M] Cutaneous lymphoma |
| **B602z00** | Burkitt's lymphoma NOS |
| **B630000** | Malignant plasma cell neoplasm, extramedullary plasmacytoma |
| **B610.00** | Hodgkin's paragranuloma |
| **B602200** | Burkitt's lymphoma of intrathoracic lymph nodes |
| **BBgQ.00** | [M]Malignant lymphomatous polyposis |
| **B622.00** | Sezary's disease |
| **BBmK.00** | [M]Waldenstrom's macroglobulinaemia |
| **B62z300** | Unspec malig neop lymphoid/histiocytic intra-abdominal nodes |
| **B936.12** | Plasmacytoma NOS |
| **BBg1.00** | [M]Malignant lymphoma NOS |
| **B935.12** | Mastocytoma NOS |
| **B610300** | Hodgkin's paragranuloma of intra-abdominal lymph nodes |
| **A789600** | HIV disease resulting in Burkitt's lymphoma |
| **B602500** | Burkitt's lymphoma of lymph nodes of inguinal region and leg |
| **BBj2.00** | [M]Hodgkin's disease, mixed cellularity |
| **BBm5.00** | [M] Peripheral T-cell lymphoma NOS |
| **BBgG.12** | [M]Lymphoblastic lymphoma NOS |
| **BBg1.11** | [M]Lymphoma NOS |
| **ByuD200** | [X]Other types of diffuse non-Hodgkin's lymphoma |
| **B625200** | Letterer-Siwe disease of intrathoracic lymph nodes |
| **B627D00** | Diffuse non-Hodgkin's centroblastic lymphoma |

# Metastatic cancer (Elixhauser)

| **Read code** | **Description** |
| --- | --- |
| **B58y500** | Secondary malignant neoplasm of prostate |
| **B56y.00** | Secondary and unspec malig neop lymph nodes multiple sites |
| **B582.00** | Secondary malignant neoplasm of skin |
| **BB04.00** | [M]Neoplasm, malig, uncertain whether primary or metastatic |
| **7H62z00** | Excision or biopsy of lymph node NOS |
| **B153.00** | Secondary malignant neoplasm of liver |
| **B583000** | Secondary malignant neoplasm of brain |
| **B565400** | Secondary and unspec malig neop obturator lymph nodes |
| **B576000** | Secondary malignant neoplasm of retroperitoneum |
| **1972B** | SECONDARY NEOPLASTIC DEPOSITS PLEURA |
| **B574100** | Secondary malignant neoplasm of jejunum |
| **B561z00** | Secondary and unspec malig neop intrathoracic LN NOS |
| **ByuC300** | [X]Secondary malignant neoplasm/oth+unspc respiratory organs |
| **B58y211** | Secondary cancer of the cervix |
| **BB03.11** | [M]Secondary neoplasm |
| **B574z00** | Secondary malig neop of small intestine or duodenum NOS |
| **1976A** | MALIGNANT ASCITES |
| **B581200** | Secondary malignant neoplasm of urethra |
| **BB2B.00** | [M]Squamous cell carcinoma, metastatic NOS |
| **B561600** | Secondary and unspec malig neop superfic tracheobronchial LN |
| **B565200** | Secondary and unspec malig neop circumflex iliac LN |
| **B562z00** | Secondary and unspec malig neop intra-abdominal LN NOS |
| **1983AM** | ADENOCARCINOMA CEREBELLAR METASTATIC |
| **B583.00** | Secondary malignant neoplasm of brain and spinal cord |
| **B586.00** | Secondary malignant neoplasm of ovary |
| **1990NB** | NEUROBLASTOMA DISSEMINATED |
| **BBy2.00** | [M]No microscopic confirmation tumour, clinically metastatic |
| **B560700** | Secondary and unspec malig neop submental lymph nodes |
| **B563300** | Secondary and unspec malig neop pectoral lymph nodes |
| **B561000** | Secondary and unspec malig neop internal mammary lymph nodes |
| **B58z.00** | Secondary malignant neoplasm of other specified site NOS |
| **B574200** | Secondary malignant neoplasm of ileum |
| **B563200** | Secondary and unspec malig neop infraclavicular lymph nodes |
| **1982.00** | SKIN SECONDARY DEPOSITS |
| **B582500** | Secondary malignant neoplasm of skin of hip and leg |
| **B562300** | Secondary and unspec malig neop common iliac lymph nodes |
| **B564200** | Secondary and unspec malig neop popliteal lymph nodes |
| **1990M** | METASTASIS GENERAL |
| **B57..00** | Secondary malig neop of respiratory and digestive systems |
| **B58y000** | Secondary malignant neoplasm of breast |
| **1989.00** | SECONDARY NEOPLASTIC DEPOSITS |
| **B56..11** | Lymph node metastases |
| **B582600** | Secondary malignant neoplasm of skin of breast |
| **2022MT** | NEOPLASM MALIGNANT LYMPHOMA METASTATIC |
| **B58y411** | Secondary cancer of the vulva |
| **B581.00** | Secondary malignant neoplasm of other urinary organs |
| **B562100** | Secondary and unspec malig neop superficial mesenteric LN |
| **B583z00** | Secondary malignant neoplasm of brain or spinal cord NOS |
| **B58y600** | Secondary malignant neoplasm of testis |
| **BB14.00** | [M]Carcinomatosis |
| **B58y100** | Secondary malignant neoplasm of uterus |
| **BB03.00** | [M]Neoplasm, metastatic |
| **B560400** | Secondary and unspec malig neop deep parotid lymph nodes |
| **B575z00** | Secondary malig neop of large intestine or rectum NOS |
| **B582400** | Secondary malignant neoplasm of skin of shoulder and arm |
| **B581z00** | Secondary malignant neoplasm of other urinary organ NOS |
| **B576100** | Secondary malignant neoplasm of peritoneum |
| **B582100** | Secondary malignant neoplasm of skin of face |
| **1969M** | SECONDARY NEOPLASTIC DEPOSITS LYMPH GLAN |
| **ByuC.00** | [X]Malignant neoplasm of ill-defined, secondary and unspeci |
| **B587.00** | Secondary malignant neoplasm of adrenal gland |
| **1976M** | PERITONITIS MALIGNANT |
| **B58..11** | Secondary carcinoma of other specified sites |
| **ByuC500** | [X]2ndry malignant neoplasm/bladder+oth+unsp urinary organs |
| **B565300** | Secondary and unspec malig neop sacral lymph nodes |
| **B570.00** | Secondary malignant neoplasm of lung |
| **1970.00** | SECONDARY NEOPLASTIC DEPOSITS LUNG |
| **ByuC400** | [X]Secondary malignant neoplasm/oth+unspcfd digestive organs |
| **B561500** | Secondary and unspec malig neop paratracheal lymph nodes |
| **B583100** | Secondary malignant neoplasm of spinal cord |
| **1969A** | MALIGNANT NEOPLASM LYMPH GLAND/NODE |
| **B581000** | Secondary malignant neoplasm of ureter |
| **B561300** | Secondary and unspec malig neop ant mediastinal lymph nodes |
| **B575000** | Secondary malignant neoplasm of colon |
| **B565000** | Secondary and unspec malig neop internal iliac lymph nodes |
| **B575.00** | Secondary malignant neoplasm of large intestine and rectum |
| **B585000** | Pathological fracture due to metastatic bone disease |
| **1D18.00** | Pain from metastases |
| **209 BL** | MYELOSCLEROSIS SECONDARY |
| **B576.00** | Secondary malig neop of retroperitoneum and peritoneum |
| **B563100** | Secondary and unspec malig neop supratrochlear lymph nodes |
| **B561900** | Secondary and unspec malig neop pulmonary lymph nodes |
| **1977A** | MALIGNANT NEOPLASM LIVER SECONDARY |
| **209 BF** | MYELOFIBROSIS SECONDARY |
| **B560000** | Secondary and unspec malig neop of superficial parotid LN |
| **B561700** | Secondary and unspec malig neop inferior tracheobronchial LN |
| **BB13.00** | [M]Carcinoma, metastatic, NOS |
| **B564z00** | Secondary and unspec malig neop of inguinal and leg LN NOS |
| **B58..00** | Secondary malignant neoplasm of other specified sites |
| **1983.00** | SECONDARY NEOPLASTIC DEPOSITS BRAIN |
| **B56..00** | Secondary and unspecified malignant neoplasm of lymph nodes |
| **B574.00** | Secondary malignant neoplasm of small intestine and duodenum |
| **B560500** | Secondary and unspec malig neop submandibular lymph nodes |
| **B560100** | Secondary and unspec malignant neoplasm mastoid lymph nodes |
| **B561800** | Secondary and unspec malig neop bronchopulmonary lymph nodes |
| **B577.00** | Secondary malignant neoplasm of liver |
| **B565100** | Secondary and unspec malig neop inferior epigastric LN |
| **B582200** | Secondary malignant neoplasm of skin of neck |
| **B564000** | Secondary and unspec malig neop superficial inguinal LN |
| **B562.00** | Secondary and unspec malig neop intra-abdominal lymph nodes |
| **B575100** | Secondary malignant neoplasm of rectum |
| **B582300** | Secondary malignant neoplasm of skin of trunk |
| **B58y800** | Secondary malignant neoplasm of epididymis and vas deferens |
| **B561200** | Secondary and unspec malig neop diaphragmatic lymph nodes |
| **C354A00** | Metastatic calcification |
| **B562000** | Secondary and unspec malig neop coeliac lymph nodes |
| **ByuC200** | [X]2ndry+unspcf malignant neoplasm lymph nodes/multi regions |
| **B564100** | Secondary and unspec malig neop deep inguinal lymph nodes |
| **B57..11** | Metastases of respiratory and/or digestive systems |
| **B57z.00** | Secondary malig neop of respiratory or digestive system NOS |
| **B58y.00** | Secondary malignant neoplasm of other specified sites |
| **1990C** | NEOPLASTIC DISEASE DISSEMINATED |
| **B565z00** | Secondary and unspec malig neop intrapelvic LN NOS |
| **B565.00** | Secondary and unspec malig neop intrapelvic lymph nodes |
| **1973M** | METASTASIS BRONCHUS |
| **ByuC700** | [X]Secondary malignant neoplasm of other specified sites |
| **B580.00** | Secondary malignant neoplasm of kidney |
| **B573.00** | Secondary malignant neoplasm of other respiratory organs |
| **B58y400** | Secondary malignant neoplasm of vulva |
| **B560.00** | Secondary and unspec malig neop lymph nodes head/face/neck |
| **B563000** | Secondary and unspec malig neop axillary lymph nodes |
| **B572.00** | Secondary malignant neoplasm of pleura |
| **B561100** | Secondary and unspec malig neop intercostal lymph nodes |
| **B590.00** | Disseminated malignancy NOS |
| **1983M** | METASTASIS CEREBRAL |
| **B58y200** | Secondary malignant neoplasm of cervix uteri |
| **B560800** | Secondary and unspec malig neop anterior cervical LN |
| **B58yz00** | Secondary malignant neoplasm of other specified site NOS |
| **B594.00** | Secondary malignant neoplasm of unknown site |
| **1985.00** | SECONDARY NEOPLASTIC DEPOSITS BONE |
| **BB85100** | [M]Metastatic signet ring cell carcinoma |
| **B560300** | Secondary and unspec malignant neoplasm occipital lymph node |
| **B57y.00** | Secondary malignant neoplasm of other digestive organ |
| **B582000** | Secondary malignant neoplasm of skin of head |
| **B562400** | Secondary and unspec malig neop external iliac lymph nodes |
| **B560200** | Secondary and unspec malig neop superficial cervical LN |
| **B562200** | Secondary and unspec malig neop inferior mesenteric LN |
| **B590.11** | Carcinomatosis |
| **B583200** | Cerebral metastasis |
| **B564.00** | Secondary and unspec malig neop inguinal and lower limb LN |
| **B584.00** | Secondary malignant neoplasm of other part of nervous system |
| **B563.00** | Secondary and unspec malig neop axilla and upper limb LN |
| **1977.00** | SECONDARY NEOPLASTIC DEPOSITS LIVER |
| **B58y300** | Secondary malignant neoplasm of vagina |
| **ByuC600** | [X]2ndry malignant neoplasm/oth+unspec parts/nervous system |
| **B560600** | Secondary and unspec malig neop of facial lymph nodes |
| **B5y..00** | Malignant neoplasm of other and unspecified site OS |
| **B57..12** | Secondary carcinoma of respiratory and/or digestive systems |
| **B576200** | Malignant ascites |
| **B585.00** | Secondary malignant neoplasm of bone and bone marrow |
| **B582z00** | Secondary malignant neoplasm of skin NOS |
| **1968M** | SECONDARY NEOPLASTIC DEPOSITS MULTI SITE |
| **B591.00** | Other malignant neoplasm NOS |
| **B581100** | Secondary malignant neoplasm of bladder |
| **B56z.00** | Secondary and unspec malig neop lymph nodes NOS |
| **B571.00** | Secondary malignant neoplasm of mediastinum |
| **1989M** | METASTASIS |
| **B561400** | Secondary and unspec malig neop post mediastinal lymph nodes |
| **1970M** | METASTASIS LUNG |
| **B576z00** | Secondary malig neop of retroperitoneum or peritoneum NOS |
| **1972A** | PLEURAL EFFUSION MALIGNANT SECONDARY |
| **B563z00** | Secondary and unspec malig neop axilla and upper limb LN NOS |
| **B58y700** | Secondary malignant neoplasm of penis |
| **BB53.00** | [M]Adenocarcinoma, metastatic, NOS |
| **B577.11** | Liver metastases |
| **1977M** | METASTASIS LIVER |
| **B574000** | Secondary malignant neoplasm of duodenum |
| **B58y900** | Secondary malignant neoplasm of tongue |
| **B560900** | Secondary and unspec malig neop deep cervical LN |
| **B560z00** | Secondary unspec malig neop lymph nodes head/face/neck NOS |
| **B561.00** | Secondary and unspec malig neop intrathoracic lymph nodes |

# Obesity (Elixhauser)

| **Read code** | **Description** |
| --- | --- |
| **9OK7.00** | Obesity monitoring verbal inv. |
| **C380800** | Childhood obesity |
| **66C6.00** | Treatment of obesity started |
| **C380300** | Morbid obesity |
| **C380.00** | Obesity |
| **22K2.00** | Body Mass Index high K/M2 |
| **66CZ.00** | Obesity monitoring NOS |
| **9hN1.00** | Excepted from obesity quality indicators: informed dissent |
| **C380100** | Drug-induced obesity |
| **ZV65319** | [V]Dietary counselling in obesity |
| **66CP.00** | Risk health associ overweight and obesity, at very high risk |
| **66C5.00** | Treatment of obesity changed |
| **22K7.00** | Body mass index 40+ - severely obese |
| **9OK2.00** | Refuses obesity monitoring |
| **9OK1.00** | Attends obesity monitoring |
| **9OKA.00** | Obesity monitoring check done |
| **22A4.11** | O/E - overweight |
| **C380700** | Lifelong obesity |
| **22K4.00** | Body mass index index 25-29 - overweight |
| **Cyu7000** | [X]Other obesity |
| **9OK4.00** | Obesity monitoring 1st letter |
| **C38..00** | Obesity and other hyperalimentation |
| **C380400** | Central obesity |
| **9OKZ.00** | Obesity monitoring admin.NOS |
| **66C4.00** | Has seen dietician - obesity |
| **C380500** | Generalised obesity |
| **9OK..11** | Obesity clinic administration |
| **66C7.00** | Treatment of obesity stopped |
| **L161.12** | Maternal obesity syndrome |
| **22K5.00** | Body mass index 30+ - obesity |
| **66CN.00** | Risk health associated overweight and obesity, at high risk |
| **22AA.00** | Overweight |
| **C38z.00** | Obesity and other hyperalimentation NOS |
| **C380200** | Extreme obesity with alveolar hypoventilation |
| **C380000** | Obesity due to excess calories |
| **66CE.00** | Reason for obesity therapy - occupational |
| **9OK5.00** | Obesity monitoring 2nd letter |
| **C38z000** | Simple obesity NOS |
| **9OK8.00** | Obesity monitor phone invite |
| **1444.00** | H/O: obesity |
| **ZC2CM00** | Dietary advice for obesity |
| **66C1.00** | Initial obesity assessment |
| **9OK3.00** | Obesity monitoring default |
| **66C2.00** | Follow-up obesity assessment |
| **66C..00** | Obesity monitoring |
| **C380600** | Adult-onset obesity |
| **9hN0.00** | Excepted from obesity quality indicators: patient unsuitable |
| **9OK6.00** | Obesity monitoring 3rd letter |
| **Cyu7.00** | [X]Obesity and other hyperalimentation |
| **C38y011** | Obesity hypoventilation syndrome |
| **9OK..00** | Obesity monitoring admin. |
| **66CS.00** | Inter risk hlth overwght obesity adv diet phys act cons drug |

# Other neurological disorders (Elixhauser)

| **Read code** | **Description** |
| --- | --- |
| **E001000** | Uncomplicated presenile dementia |
| **P102.13** | Myelocele with hydrocephalus |
| **F255012** | Motor epilepsy |
| **F255z00** | Partial epilepsy without impairment of consciousness NOS |
| **F25y200** | Locl-rlt(foc)(part)idiop epilep&epilptic syn seiz locl onset |
| **S63z.00** | Other cerebral haemorrhage following injury NOS |
| **F11x400** | Cerebral degeneration due to neoplastic disease |
| **G618.00** | Intracerebral haemorrhage, multiple localized |
| **P100100** | Cervical spina bifida with hydrocephalus |
| **G677400** | Occlusion+stenosis of multiple and bilat cerebral arteries |
| **S628.00** | Traumatic subdural haemorrhage |
| **F259.00** | Early infant epileptic encephalopathy wth suppression bursts |
| **F152000** | Amyotrophic lateral sclerosis |
| **G656.00** | Vertebrobasilar insufficiency |
| **F130300** | Parkinsonism with orthostatic hypotension |
| **SL6y.00** | Antiparkinsonism drug poisoning |
| **14A7.12** | H/O: stroke |
| **G60X.00** | Subarachnoid haemorrh from intracranial artery, unspecif |
| **P11..00** | Spina bifida without mention of hydrocephalus |
| **F250z00** | Generalised nonconvulsive epilepsy NOS |
| **8Hkv.00** | Referral to community multiple sclerosis team |
| **P102.11** | Fissured spine with hydrocephalus |
| **666B.00** | Multiple sclerosis multidisciplinary review |
| **E041.00** | Dementia in conditions EC |
| **F040111** | Cerebellar abscess |
| **P103z11** | Thoracolumbar spina bifida with hydrocephalus - closed |
| **G677100** | Occlusion and stenosis of anterior cerebral artery |
| **P117z00** | Spina bifida without hydrocephalus - open NOS |
| **F25y000** | Cursive (running) epilepsy |
| **G64..00** | Cerebral arterial occlusion |
| **F135000** | Hemiballismus |
| **P102.00** | Spina bifida with hydrocephalus - open |
| **F151z00** | Spinal muscular atrophy NOS |
| **F037.00** | Transverse myelitis |
| **G677300** | Occlusion and stenosis of cerebellar arteries |
| **Eu04100** | [X]Delirium superimposed on dementia |
| **P110300** | Lumbar spina bifida without mention of hydrocephalus |
| **G630.00** | Basilar artery occlusion |
| **G66..13** | CVA - Cerebrovascular accident unspecified |
| **F113z00** | Communicating hydrocephalus - acquired NOS |
| **F250.00** | Generalised nonconvulsive epilepsy |
| **F361.00** | Peroneal muscular atrophy |
| **Eu01y00** | [X]Other vascular dementia |
| **F29y300** | Toxic encephalopathy |
| **Q417100** | Cerebellar (nontraum) and post fossa haemorhage fet newborn |
| **F283.00** | Unspecified encephalopathy |
| **P102.14** | Rachischisis with hydrocephalus |
| **9h2..00** | Exception reporting: stroke quality indicators |
| **2987.11** | O/E - Parkinson posture |
| **F11y100** | Cerebral ataxia |
| **C251.11** | Wernicke's encephalopathy |
| **F255400** | Visual reflex epilepsy |
| **Fyu5700** | [X]Other vascular syndroms/brain in cerebrovasculr diseases |
| **P11z.00** | Spina bifida without mention of hydrocephalus NOS |
| **F14yz00** | Other spinocerebellar disease NOS |
| **Eu02000** | [X]Dementia in Pick's disease |
| **G66..11** | CVA unspecified |
| **G668.00** | Right sided CVA |
| **1M4..00** | Central post-stroke pain |
| **Pyu0400** | [X]Unspecified spina bifida with hydrocephalus |
| **1JA1.00** | Suspected cerebrovascular disease |
| **Gyu6300** | [X]Cerebrl infarctn due/unspcf occlusn or sten/cerebrl artrs |
| **667R.00** | 2 to 4 seizures a month |
| **F25z.11** | Fit (in known epileptic) NOS |
| **F113011** | Low pressure hydrocephalus |
| **G64z111** | Lateral medullary syndrome |
| **E001.00** | Presenile dementia |
| **G614.00** | Pontine haemorrhage |
| **Z7E4.00** | Ataxia |
| **F210.00** | Neuromyelitis optica |
| **G641000** | Cerebral infarction due to embolism of cerebral arteries |
| **G679.00** | Small vessel cerebrovascular disease |
| **Pyu0100** | [X]Other congenital hydrocephalus |
| **Eu02z11** | [X] Presenile dementia NOS |
| **P118.00** | Spina bifida without hydrocephalus - closed |
| **F142200** | Dyssynergia cerebellaris myoclonica |
| **F142z00** | Primary cerebellar degeneration NOS |
| **9kG..00** | Spec serv for pat with multiple sclerosis - enh serv admin |
| **8HHM.00** | Ref to multidisciplinary stroke function improvement service |
| **2987.00** | O/E -Parkinson flexion posture |
| **Eu01300** | [X]Mixed cortical and subcortical vascular dementia |
| **F12X.00** | Secondary parkinsonism, unspecified |
| **F124.00** | Vascular parkinsonism |
| **F142000** | Marie's cerebellar ataxia |
| **Eu00.00** | [X]Dementia in Alzheimer's disease |
| **F134.00** | Huntington's chorea |
| **F254000** | Temporal lobe epilepsy |
| **G621.00** | Subdural haemorrhage - nontraumatic |
| **667Z.00** | Epilepsy monitoring NOS |
| **F103000** | Cerebral degeneration in Hunter's disease |
| **F103100** | Cerebral degeneration in mucopolysaccharidoses |
| **F135z00** | Other choreas NOS |
| **8BIF.00** | Epilepsy medication review |
| **F040100** | Cerebellar intracranial abscess |
| **G631.00** | Carotid artery occlusion |
| **F250400** | Juvenile absence epilepsy |
| **G677000** | Occlusion and stenosis of middle cerebral artery |
| **Fyu2100** | [X]Other secondary parkinsonism |
| **667K.00** | Epilepsy limits activities |
| **F200.00** | Multiple sclerosis of the brain stem |
| **F253.11** | Status epilepticus |
| **G657.00** | Carotid territory transient ischaemic attack |
| **U606711** | [X] Adverse reaction to antiparkinsonism drug |
| **F1y0.00** | Fragile X associated tremor ataxia syndrome |
| **9Om1.00** | Stroke/transient ischaemic attack monitoring second letter |
| **F255y00** | Partial epilepsy without impairment of consciousness OS |
| **P118300** | Lumbar spina bifida without hydrocephalus - closed |
| **Z7E4300** | Truncal ataxia |
| **F135200** | Drug-induced chorea |
| **G63..00** | Precerebral arterial occlusion |
| **E002000** | Senile dementia with paranoia |
| **F208.00** | Secondary progressive multiple sclerosis |
| **2828.00** | Absence seizure |
| **Eu02100** | [X]Dementia in Creutzfeldt-Jakob disease |
| **R003300** | [D]Reflex anoxic seizure |
| **147F.00** | History of Parkinson's disease |
| **8Cc1.00** | Management of multiple sclerosis in early disease phase |
| **667..00** | Epilepsy monitoring |
| **1B1W.00** | Transient epileptic amnesia |
| **G64z200** | Left sided cerebral infarction |
| **G66..12** | Stroke unspecified |
| **G63..12** | Stenosis of precerebral arteries |
| **Gyu6100** | [X]Other subarachnoid haemorrhage |
| **F151.00** | Spinal muscular atrophy |
| **14A7.00** | H/O: CVA/stroke |
| **L440000** | Puerperal cerebrovascular disorder unspecified |
| **P10..00** | Spina bifida with hydrocephalus |
| **8BPa.00** | Antipsychotic drug therapy for dementia |
| **P117.00** | Spina bifida without hydrocephalus - open |
| **F251200** | Epileptic seizures - clonic |
| **G65zz00** | Transient cerebral ischaemia NOS |
| **F211.00** | Schilder's disease |
| **F14y100** | Corticostriatal-spinal degeneration |
| **E004100** | Arteriosclerotic dementia with delirium |
| **G613.00** | Cerebellar haemorrhage |
| **Z7E4400** | Cerebellar ataxia |
| **F251111** | Otohara syndrome |
| **G02..11** | Sydenham's chorea |
| **Fyu4000** | [X]Other specified acute disseminated demyelination |
| **F250300** | Epileptic seizures - akinetic |
| **F03..13** | Transverse myelitis |
| **Eu02z13** | [X] Primary degenerative dementia NOS |
| **G61X000** | Left sided intracerebral haemorrhage, unspecified |
| **F114.00** | Acquired obstructive hydrocephalus |
| **G64z100** | Wallenberg syndrome |
| **F103z00** | Cerebral degeneration in disease NOS |
| **E001300** | Presenile dementia with depression |
| **Eu00011** | [X]Presenile dementia,Alzheimer's type |
| **G633.00** | Multiple and bilateral precerebral arterial occlusion |
| **P22z.11** | Cerebellar hypoplasia |
| **G681.00** | Sequelae of intracerebral haemorrhage |
| **8IAi.00** | Pregnancy advice for patients with epilepsy declined |
| **F102100** | Cerebral degeneration in Niemann-Pick disease |
| **F152100** | Progressive muscular atrophy |
| **667D.00** | Epilepsy control poor |
| **G64z.12** | Cerebellar infarction |
| **Eu01111** | [X]Predominantly cortical dementia |
| **66h..00** | Dementia monitoring |
| **G63y100** | Cerebral infarction due to embolism of precerebral arteries |
| **F258.00** | Post-ictal state |
| **G650.00** | Basilar artery syndrome |
| **F123.00** | Postencephalitic parkinsonism |
| **P11y.00** | Other specified spina bifida without hydrocephalus |
| **C250.00** | Beriberi |
| **F251500** | Tonic-clonic epilepsy |
| **G66..00** | Stroke and cerebrovascular accident unspecified |
| **F255000** | Jacksonian, focal or motor epilepsy |
| **F14z.00** | Spinocerebellar disease NOS |
| **G62..00** | Other and unspecified intracranial haemorrhage |
| **G654.00** | Multiple and bilateral precerebral artery syndromes |
| **F250200** | Epileptic seizures - atonic |
| **G70y011** | Carotid artery disease |
| **G601.00** | Subarachnoid haemorrhage from carotid siphon and bifurcation |
| **F251011** | Tonic-clonic epilepsy |
| **F365.00** | Neuropathy in association with hereditary ataxia |
| **R013000** | [D]Ataxia NOS |
| **A411.00** | Jakob-Creutzfeldt disease |
| **9Of5.00** | Epilepsy monitoring call first letter |
| **F206.00** | Primary progressive multiple sclerosis |
| **G683.00** | Sequelae of cerebral infarction |
| **F120.00** | Paralysis agitans |
| **F14..11** | Cerebellar disease |
| **Eu01.00** | [X]Vascular dementia |
| **E001100** | Presenile dementia with delirium |
| **F21z.00** | Central nervous system demyelination NOS |
| **6678.00** | Epilepsy treatment changed |
| **C253.00** | Wernicke's encephalopathy |
| **9Om..00** | Stroke/transient ischaemic attack monitoring administration |
| **F25G.00** | Severe myoclonic epilepsy in infancy |
| **Eu01200** | [X]Subcortical vascular dementia |
| **9Om2.00** | Stroke/transient ischaemic attack monitoring third letter |
| **Eu00113** | [X]Primary degen dementia of Alzheimer's type, senile onset |
| **P100200** | Thoracic spina bifida with hydrocephalus |
| **G662.00** | Posterior cerebral artery syndrome |
| **F12z.00** | Parkinson's disease NOS |
| **Gyu6F00** | [X]Intracerebral haemorrhage in hemisphere, unspecified |
| **G63y000** | Cerebral infarct due to thrombosis of precerebral arteries |
| **8Cc0.00** | Management of multiple sclerosis in onset phase |
| **G682.00** | Sequelae of other nontraumatic intracranial haemorrhage |
| **G64z000** | Brainstem infarction |
| **G61..11** | CVA - cerebrovascular accid due to intracerebral haemorrhage |
| **F20..00** | Multiple sclerosis |
| **G671000** | Acute cerebrovascular insufficiency NOS |
| **L440.00** | Cerebrovascular disorders in the puerperium |
| **Fyu4.00** | [X]Demyelinating diseases of the central nervous system |
| **F11x.00** | Cerebral degeneration in other disease EC |
| **G60..00** | Subarachnoid haemorrhage |
| **U606719** | [X] Adverse react to anticonvuls or antiparkinson drug NOS |
| **F257.00** | Kojevnikov's epilepsy |
| **F144000** | Cerebellar ataxia due to alcoholism |
| **F10z.00** | Childhood cerebral degenerations NOS |
| **Eu01z00** | [X]Vascular dementia, unspecified |
| **Eu00012** | [X]Primary degen dementia, Alzheimer's type, presenile onset |
| **F11x200** | Cerebral degeneration due to cerebrovascular disease |
| **E004000** | Uncomplicated arteriosclerotic dementia |
| **Gyu6500** | [X]Occlusion and stenosis of other precerebral arteries |
| **6674.00** | Epilepsy associated problems |
| **ZS42400** | Cerebellar dysarthria |
| **Eu00013** | [X]Alzheimer's disease type 2 |
| **67AF.00** | Pregnancy advice for patients with epilepsy |
| **667V.00** | Many seizures a day |
| **F251300** | Epileptic seizures - myoclonic |
| **P100300** | Lumbar spina bifida with hydrocephalus |
| **F25z.00** | Epilepsy NOS |
| **9Ou2.00** | Dementia monitoring second letter |
| **G612.00** | Basal nucleus haemorrhage |
| **Eu02200** | [X]Dementia in Huntington's disease |
| **G6W..00** | Cereb infarct due unsp occlus/stenos precerebr arteries |
| **Eu05y11** | [X]Epileptic psychosis NOS |
| **J622.11** | Encephalopathy - hepatic |
| **G667.00** | Left sided CVA |
| **Eu01000** | [X]Vascular dementia of acute onset |
| **Gyu6000** | [X]Subarachnoid haemorrhage from other intracranial arteries |
| **667F.00** | Seizure free >12 months |
| **G663.00** | Brain stem stroke syndrome |
| **F21yz00** | Other specified central nervous system demyelination NOS |
| **667A.00** | Epilepsy treatment stopped |
| **Eu04000** | [X]Delirium not superimposed on dementia, so described |
| **P110100** | Cervical spina bifida without mention of hydrocephalus |
| **G664.00** | Cerebellar stroke syndrome |
| **F25D.00** | Menstrual epilepsy |
| **F151000** | Unspecified spinal muscular atrophy |
| **ZV12512** | [V]Personal history of cerebrovascular accident (CVA) |
| **G68X.00** | Sequelae of stroke,not specfd as h'morrhage or infarction |
| **G62z.00** | Intracranial haemorrhage NOS |
| **Fyu2600** | [X]Other chorea |
| **F25A.00** | Juvenile myoclonic epilepsy |
| **G63..11** | Infarction - precerebral |
| **Eu00z00** | [X]Dementia in Alzheimer's disease, unspecified |
| **G619.00** | Lobar cerebral haemorrhage |
| **G021.00** | Rheumatic chorea without mention of heart involvement |
| **F25y100** | Gelastic epilepsy |
| **9h6..00** | Exception reporting: epilepsy quality indicators |
| **8CRB.00** | Transient ischaemic attack clinical management plan |
| **F11X.00** | Post-traumatic hydrocephalus, unspecified |
| **F110.00** | Alzheimer's disease |
| **F25X.00** | Status epilepticus, unspecified |
| **6679.00** | Epilepsy treatment started |
| **P118100** | Cervical spina bifida without hydrocephalus - closed |
| **G676000** | Cereb infarct due cerebral venous thrombosis, nonpyogenic |
| **F25y.00** | Other forms of epilepsy |
| **F203.00** | Exacerbation of multiple sclerosis |
| **F207.00** | Relapsing and remitting multiple sclerosis |
| **C315100** | Mitochond encephalopathy, lact acidosis & strokelike episode |
| **Q402311** | Congenital hydrocephalus due to toxoplasmosis |
| **F250500** | Lennox-Gastaut syndrome |
| **P118400** | Sacral spina bifida without hydrocephalus - closed |
| **P7y0.00** | Cerebrovascular system anomalies |
| **8HBJ.00** | Stroke / transient ischaemic attack referral |
| **F256.11** | Lightning spasms |
| **E000.00** | Uncomplicated senile dementia |
| **F254400** | Epileptic automatism |
| **F037000** | Varicella transverse myelitis |
| **E02y100** | Drug-induced dementia |
| **6677.00** | Epilepsy drug side effects |
| **G653.00** | Carotid artery syndrome hemispheric |
| **7A20400** | Endarterectomy of carotid artery NEC |
| **Eu02z16** | [X] Senile dementia, depressed or paranoid type |
| **P10y.00** | Other specified spina bifida with hydrocephalus |
| **F11y.00** | Other cerebral degeneration |
| **667S.00** | 1 to 7 seizures a week |
| **F150.11** | Infantile spinal muscular atrophy |
| **Eu00z11** | [X]Alzheimer's dementia unspec |
| **Eu02300** | [X]Dementia in Parkinson's disease |
| **G616.00** | External capsule haemorrhage |
| **9h22.00** | Excepted from stroke quality indicators: Informed dissent |
| **E00..11** | Senile dementia |
| **FyuA100** | [X]Other hydrocephalus |
| **662o.00** | Haemorrhagic stroke monitoring |
| **E003.00** | Senile dementia with delirium |
| **F201.00** | Multiple sclerosis of the spinal cord |
| **A413.00** | Progressive multifocal leucoencephalopathy |
| **G64z400** | Infarction of basal ganglia |
| **FyuAG00** | [X]Post-traumatic hydrocephalus, unspecified |
| **P235.00** | X-linked hydrocephalus |
| **F281.11** | Anoxic - ischaemic encephalopathy |
| **P23z.00** | Congenital hydrocephalus NOS |
| **F132100** | Progressive myoclonic epilepsy |
| **P110z00** | Unspecified spina bifida without hydrocephalus NOS |
| **G660.00** | Middle cerebral artery syndrome |
| **P118000** | Unspecified spina bifida without hydrocephalus - closed |
| **F251z00** | Generalised convulsive epilepsy NOS |
| **G671.00** | Generalised ischaemic cerebrovascular disease NOS |
| **G65..00** | Transient cerebral ischaemia |
| **F21y211** | Binswanger's encephalopathy |
| **Q200011** | Intracerebral haemorrhage in fetus or newborn |
| **G65..13** | Vertebro-basilar insufficiency |
| **Eu02500** | [X]Lewy body dementia |
| **662e.11** | Stroke annual review |
| **F144200** | Cerebellar ataxia due to neoplasia |
| **G65y.00** | Other transient cerebral ischaemia |
| **G650.11** | Insufficiency - basilar artery |
| **P100.00** | Unspecified spina bifida with hydrocephalus |
| **Eu00100** | [X]Dementia in Alzheimer's disease with late onset |
| **G61X100** | Right sided intracerebral haemorrhage, unspecified |
| **G64z.11** | Brainstem infarction NOS |
| **F113000** | Normal pressure hydrocephalus |
| **Fyu2000** | [X]Other drug-induced secondary parkinsonism |
| **G63z.00** | Precerebral artery occlusion NOS |
| **G67..00** | Other cerebrovascular disease |
| **G67z.00** | Other cerebrovascular disease NOS |
| **9Of4.00** | Epilepsy monitoring telephone invite |
| **F250000** | Petit mal (minor) epilepsy |
| **F251100** | Neonatal myoclonic epilepsy |
| **Fyu5100** | [X]Other epilepsy |
| **Fyu1000** | [X]Other hereditary ataxias |
| **Gyu6600** | [X]Occlusion and stenosis of other cerebral arteries |
| **F15z.00** | Anterior horn cell disease NOS |
| **9Of3.00** | Epilepsy monitoring verbal invite |
| **P103400** | Sacral spina bifida with hydrocephalus - closed |
| **F11z.00** | Cerebral degeneration NOS |
| **F11xz00** | Cerebral degeneration other disease NOS |
| **7P24200** | Delivery of rehabilitation for stroke |
| **F140.00** | Friedreich's ataxia |
| **8IAb.00** | Multiple sclerosis review declined |
| **F204.00** | Benign multiple sclerosis |
| **9Om0.00** | Stroke/transient ischaemic attack monitoring first letter |
| **Q417000** | Intracerebral (nontraumatic) haemorrhage of fet and newborn |
| **G02z.00** | Rheumatic chorea NOS |
| **F144z00** | Cerebellar ataxia in disease NOS |
| **662e.00** | Stroke/CVA annual review |
| **F374100** | Polyneuropathy in beriberi |
| **F152111** | Duchenne Aran muscular atrophy |
| **F256100** | Salaam attacks |
| **E002z00** | Senile dementia with depressive or paranoid features NOS |
| **G672.00** | Hypertensive encephalopathy |
| **9Ou4.00** | Dementia monitoring verbal invite |
| **8CMZ.00** | Dementia care plan |
| **R003z11** | [D]Seizure NOS |
| **F251y00** | Other specified generalised convulsive epilepsy |
| **F255100** | Sensory induced epilepsy |
| **G651.00** | Vertebral artery syndrome |
| **F256000** | Hypsarrhythmia |
| **L440.11** | CVA - cerebrovascular accident in the puerperium |
| **G64z300** | Right sided cerebral infarction |
| **F117.00** | Infantile posthaemorrhagic hydrocephalus |
| **Eu00111** | [X]Alzheimer's disease type 1 |
| **U606600** | [X]Oth unspec antiepileptics caus adverse eff in therap use |
| **F113.00** | Acquired communicating hydrocephalus |
| **F25C.00** | Drug-induced epilepsy |
| **F255500** | Unilateral epilepsy |
| **F250011** | Epileptic absences |
| **F254z00** | Partial epilepsy with impairment of consciousness NOS |
| **F12..00** | Parkinson's disease |
| **Gyu6400** | [X]Other cerebral infarction |
| **F251400** | Epileptic seizures - tonic |
| **F144.00** | Cerebellar ataxia in diseases EC |
| **Gyu6200** | [X]Other intracerebral haemorrhage |
| **G615.00** | Bulbar haemorrhage |
| **L440100** | Puerperal cerebrovascular disorder - delivered |
| **SL6z.00** | Anticonvulsant or antiparkinsonian drug poisoning NOS |
| **P10z.00** | Spina bifida with hydrocephalus NOS |
| **P7y0y00** | Other specified cerebrovascular anomaly |
| **Fyu5900** | [X]Status epilepticus, unspecified |
| **F151200** | Adult spinal muscular atrophy |
| **F253.00** | Grand mal status |
| **G6...00** | Cerebrovascular disease |
| **7A20300** | Endarterectomy and patch repair of carotid artery |
| **Gyu6G00** | [X]Cereb infarct due unsp occlus/stenos precerebr arteries |
| **661M700** | Stroke self-management plan agreed |
| **8Cc4.00** | Management of multiple sclerosis in palliative phase |
| **E004200** | Arteriosclerotic dementia with paranoia |
| **667W.00** | Emergency epilepsy treatment since last appointment |
| **F254200** | Psychosensory epilepsy |
| **F14y.00** | Other spinocerebellar diseases |
| **BBbW.00** | [M]Cerebellar sarcoma NOS |
| **9mD1.00** | Multiple sclerosis monitoring second letter |
| **TJ64.00** | Adverse reaction to antiparkinsonism drugs |
| **Gyu6A00** | [X]Other cerebrovascular disorders in diseases CE |
| **G641.11** | Cerebral embolus |
| **E002.00** | Senile dementia with depressive or paranoid features |
| **G602.00** | Subarachnoid haemorrhage from middle cerebral artery |
| **SC20000** | Traumatic epilepsy |
| **G61..00** | Intracerebral haemorrhage |
| **F255200** | Somatosensory epilepsy |
| **8IEC.00** | Ref multidisciplinary stroke function improvement declined |
| **9Ou3.00** | Dementia monitoring third letter |
| **F255011** | Focal epilepsy |
| **G600.00** | Ruptured berry aneurysm |
| **Fyu2900** | [X]Secondary parkinsonism, unspecified |
| **A94y100** | Syphilitic parkinsonism |
| **667H.00** | Epilepsy prevents employment |
| **P118z00** | Spina bifida without hydrocephalus - closed NOS |
| **G6z..00** | Cerebrovascular disease NOS |
| **G61..12** | Stroke due to intracerebral haemorrhage |
| **Eu00200** | [X]Dementia in Alzheimer's dis, atypical or mixed type |
| **9Of6.00** | Epilepsy monitoring call second letter |
| **Q437000** | Bilirubin encephalopathy |
| **666A.00** | Multiple sclerosis review |
| **P102400** | Sacral spina bifida with hydrocephalus - open |
| **G605.00** | Subarachnoid haemorrhage from basilar artery |
| **E001200** | Presenile dementia with paranoia |
| **ZS7C500** | Language disorder of dementia |
| **F135.00** | Other choreas |
| **P104.00** | Spina bifida with hydrocephalus of late onset |
| **G631.11** | Stenosis, carotid artery |
| **E004.00** | Arteriosclerotic dementia |
| **R003400** | [D]Nocturnal seizure |
| **F25y500** | Panayiotopoulos syndrome |
| **P110000** | Spina bifida without hydrocephalus, site unspecified |
| **F11x900** | Cerebral degeneration in Parkinson's disease |
| **667J.00** | Epilepsy impairs education |
| **F25H.00** | Generalised seizure |
| **P117300** | Lumbar spina bifida without hydrocephalus - open |
| **8Hd6.00** | Admission to stroke unit |
| **G651000** | Vertebro-basilar artery syndrome |
| **G65z.00** | Transient cerebral ischaemia NOS |
| **6672.00** | Follow-up epilepsy assessment |
| **Eu01100** | [X]Multi-infarct dementia |
| **Fyu5200** | [X]Other status epilepticus |
| **G02..00** | Rheumatic chorea |
| **F259.11** | Ohtahara syndrome |
| **7Q04100** | Amyotrophic lateral sclerosis drugs Band 1 |
| **667N.00** | Epilepsy severity |
| **F13..11** | Extrapyramidal disease excluding Parkinson's disease |
| **662M100** | Stroke 6 month review |
| **ZS42113** | Suprabulbar palsy type of dysarthria |
| **F11x500** | Cerebral degeneration due to myxoedema |
| **E030400** | Acute confusional state, of cerebrovascular origin |
| **G606.00** | Subarachnoid haemorrhage from vertebral artery |
| **F11x800** | Cerebral degeneration due to multifocal leucoencephalopathy |
| **F25F.00** | Photosensitive epilepsy |
| **G64..11** | CVA - cerebral artery occlusion |
| **Eu02z00** | [X] Unspecified dementia |
| **38G3.00** | Hyperten, abnorm renal/liver funct, stroke, BLED score |
| **F250y00** | Other specified generalised nonconvulsive epilepsy |
| **Eu06013** | [X]Limbic epilepsy personality |
| **G64..12** | Infarction - cerebral |
| **E001z00** | Presenile dementia NOS |
| **G61X.00** | Intracerebral haemorrhage in hemisphere, unspecified |
| **F25yz00** | Other forms of epilepsy NOS |
| **Eu01.11** | [X]Arteriosclerotic dementia |
| **1473.00** | H/O: epilepsy |
| **E00..12** | Senile/presenile dementia |
| **667T.00** | Daily seizures |
| **E002100** | Senile dementia with depression |
| **F255311** | Partial epilepsy with autonomic symptoms |
| **TJ64z00** | Adverse reaction to antiparkinsonism drugs NOS |
| **667G.00** | Epilepsy restricts employment |
| **Eu02z14** | [X] Senile dementia NOS |
| **G641.00** | Cerebral embolism |
| **G623.00** | Subdural haemorrhage NOS |
| **F250100** | Pykno-epilepsy |
| **9Om3.00** | Stroke/transient ischaemic attack monitoring verbal invitati |
| **P102200** | Thoracic spina bifida with hydrocephalus - open |
| **P7y0z00** | Cerebrovascular system anomaly NOS |
| **G611.00** | Internal capsule haemorrhage |
| **7004300** | Evacuation of intracerebral haematoma NEC |
| **F103.00** | Cerebral degeneration in diseases EC |
| **Gyu6.00** | [X]Cerebrovascular diseases |
| **G666.00** | Pure sensory lacunar syndrome |
| **F252.00** | Petit mal status |
| **U606.11** | [X] Adverse react to anticonvulsants & anti-parkinsons drugs |
| **F251000** | Grand mal (major) epilepsy |
| **G677.00** | Occlusion/stenosis cerebral arts not result cerebral infarct |
| **F110000** | Alzheimer's disease with early onset |
| **F254500** | Complex partial epileptic seizure |
| **F255600** | Simple partial epileptic seizure |
| **G634.00** | Carotid artery stenosis |
| **P100000** | Spina bifida with hydrocephalus, unspecified |
| **F152300** | Pseudobulbar palsy |
| **P117400** | Sacral spina bifida without hydrocephalus - open |
| **1477.00** | H/O: cerebrovascular disease |
| **F202.00** | Generalised multiple sclerosis |
| **7A20311** | Carotid endarterectomy and patch |
| **P103300** | Lumbar spina bifida with hydrocephalus - closed |
| **8IAg.00** | Contraceptive advice for patients with epilepsy declined |
| **13YA.00** | Stroke group member |
| **F256.00** | Infantile spasms |
| **667E.00** | Epilepsy care arrangement |
| **F151111** | Juvenile spinal muscular atrophy |
| **F15y.00** | Other anterior horn cell disease |
| **9mD0.00** | Multiple sclerosis monitoring first letter |
| **F15..00** | Anterior horn cell disease |
| **ZS42111** | Pseudobulbar palsy type of dysarthria |
| **667M.00** | Epilepsy management plan given |
| **F21..00** | Other central nervous system demyelinating diseases |
| **L440300** | Puerperal cerebrovascular disorder with antenatal comp |
| **G60z.00** | Subarachnoid haemorrhage NOS |
| **P233.12** | Hydrocephalus with atresia of foramina of Magendie+Luschka |
| **9h21.00** | Excepted from stroke quality indicators: Patient unsuitable |
| **F256.12** | West syndrome |
| **8IAh.00** | Pre-conception advice for patients with epilepsy declined |
| **Eu00112** | [X]Senile dementia,Alzheimer's type |
| **F14..00** | Spinocerebellar disease |
| **F25..00** | Epilepsy |
| **F25E.00** | Stress-induced epilepsy |
| **G6y..00** | Other specified cerebrovascular disease |
| **P100z00** | Spina bifida with hydrocephalus NOS |
| **Eu80300** | [X]Acquired aphasia with epilepsy [Landau - Kleffner] |
| **A413.11** | Progressive multifocal leukoencephalopathy |
| **Eu02y00** | [X]Dementia in other specified diseases classif elsewhere |
| **8Hla.00** | Referral to dementia care advisor |
| **F24y000** | Progressive supranuclear palsy |
| **G640000** | Cerebral infarction due to thrombosis of cerebral arteries |
| **F11yz00** | Other cerebral degeneration NOS |
| **F110100** | Alzheimer's disease with late onset |
| **P102300** | Lumbar spina bifida with hydrocephalus - open |
| **9Ou..00** | Dementia monitoring administration |
| **F256z00** | Infantile spasms NOS |
| **A940.11** | Locomotor ataxia |
| **1O30.00** | Epilepsy confirmed |
| **G61z.00** | Intracerebral haemorrhage NOS |
| **F25y400** | Benign Rolandic epilepsy |
| **G665.00** | Pure motor lacunar syndrome |
| **G640.00** | Cerebral thrombosis |
| **G68W.00** | Sequelae/other + unspecified cerebrovascular diseases |
| **1461.00** | H/O: dementia |
| **SL6yz00** | Antiparkinsonian drug poisoning NOS |
| **F11..00** | Other cerebral degenerations |
| **E012.00** | Other alcoholic dementia |
| **F121.00** | Parkinsonism secondary to drugs |
| **F11x700** | Cerebral degeneration due to Jakob - Creutzfeldt disease |
| **F14y000** | Ataxia-telangiectasia |
| **G020.00** | Rheumatic chorea with heart involvement |
| **ZV12511** | [V]Personal history of stroke |
| **F135100** | Paroxysmal choreo-athetosis |
| **L440.12** | Stroke in the puerperium |
| **Fyu4200** | [X]Acute disseminated demyelination, unspecified |
| **FyuA400** | [X]Hydrocephalus in neoplastic disease classified elsewhere |
| **F255.00** | Partial epilepsy without impairment of consciousness |
| **G680.00** | Sequelae of subarachnoid haemorrhage |
| **14AK.00** | H/O: Stroke in last year |
| **G603.00** | Subarachnoid haemorrhage from anterior communicating artery |
| **667B.00** | Nocturnal epilepsy |
| **E004z00** | Arteriosclerotic dementia NOS |
| **F21y.00** | Other specified central nervous system demyelinating disease |
| **U606700** | [X]Antiparkinsonism drugs caus advers effects in therap use |
| **P102z00** | Spina bifida with hydrocephalus - open NOS |
| **E031400** | Subacute confusional state, of cerebrovascular origin |
| **P110200** | Thoracic spina bifida without mention of hydrocephalus |
| **F251600** | Grand mal seizure |
| **F254.00** | Partial epilepsy with impairment of consciousness |
| **Eu00000** | [X]Dementia in Alzheimer's disease with early onset |
| **F142.00** | Primary cerebellar degeneration |
| **Gyu6E00** | [X]Subarachnoid haemorrh from intracranial artery, unspecif |
| **G671z00** | Generalised ischaemic cerebrovascular disease NOS |
| **Eu02.00** | [X]Dementia in other diseases classified elsewhere |
| **67IJ000** | Pre-conception advice for patients with epilepsy |
| **F121.11** | Drug induced parkinsonism |
| **Eu84311** | [X]Dementia infantalis |
| **F25y300** | Complex partial status epilepticus |
| **G68..00** | Late effects of cerebrovascular disease |
| **F255300** | Visceral reflex epilepsy |
| **F251.00** | Generalised convulsive epilepsy |
| **P117200** | Thoracic spina bifida without hydrocephalus - open |
| **667C.00** | Epilepsy control good |
| **G617.00** | Intracerebral haemorrhage, intraventricular |
| **2994.00** | O/E-festination-Parkinson gait |
| **Eu02400** | [X]Dementia in human immunodef virus [HIV] disease |
| **G677200** | Occlusion and stenosis of posterior cerebral artery |
| **G632.00** | Vertebral artery occlusion |
| **Fyu5000** | [X]Other generalized epilepsy and epileptic syndromes |
| **F254300** | Limbic system epilepsy |
| **G64z.00** | Cerebral infarction NOS |
| **G65z100** | Intermittent cerebral ischaemia |
| **S621.00** | Open traumatic subarachnoid haemorrhage |
| **E004.11** | Multi infarct dementia |
| **F21X.00** | Acute disseminated demyelination, unspecified |
| **662M.00** | Stroke monitoring |
| **8CS1.00** | Multiple sclerosis care plan agreed |
| **F21y300** | Central demyelination of corpus callosum |
| **F143.00** | Cerebellar ataxia NOS |
| **9Ou1.00** | Dementia monitoring first letter |
| **F115.00** | Hydrocephalus |
| **5C10.00** | Carotid artery doppler abnormal |
| **U606718** | [X] Adverse reaction to antiparkinsonism drugs NOS |
| **G610.00** | Cortical haemorrhage |
| **F10..00** | Cerebral degenerations usually manifest in childhood |
| **U606.00** | [X]Antiepilept + antiparksn drug caus advers eff therap use |
| **F132z12** | Myoclonic seizure |
| **P103.00** | Spina bifida with hydrocephalus - closed |
| **G604.00** | Subarachnoid haemorrhage from posterior communicating artery |
| **8Cc2.00** | Management of multiple sclerosis in stable disability phase |
| **8IB2.00** | Contraceptiv advice for patients with epilepsy not indicated |
| **F254100** | Psychomotor epilepsy |
| **G64..13** | Stroke due to cerebral arterial occlusion |
| **667Q.00** | 1 to 12 seizures a year |
| **F11x600** | Cerebral degeneration due to vitamin B12 deficiency |
| **G67y.00** | Other cerebrovascular disease OS |
| **Fyu4100** | [X]Other specified demyelinating diseases/the CNS |
| **Fyu2200** | [X]Parkinsonism in diseases classified elsewhere |
| **F132200** | Myoclonic encephalopathy |
| **F20z.00** | Multiple sclerosis NOS |
| **G6X..00** | Cerebrl infarctn due/unspcf occlusn or sten/cerebrl artrs |
| **G63y.00** | Other precerebral artery occlusion |
| **G65..12** | Transient ischaemic attack |
| **F146.00** | Early onset cerebellar ataxia with hypogonadism |
| **297A.00** | O/E - Parkinsonian tremor |
| **Fyu3000** | [X]Other Alzheimer's disease |
| **ZS82.00** | Acquired epileptic aphasia |
| **P23..00** | Congenital hydrocephalus |

# Paralysis (Elixhauser)

| **Read code** | **Description** |
| --- | --- |
| **F232.00** | Congenital quadriplegia |
| **1B33.00** | Paralysis present |
| **F141.00** | Hereditary spastic paraplegia |
| **2837.00** | O/E - diplegia |
| **F246100** | Cauda equina syndrome with cord bladder |
| **F240.00** | Quadriplegia |
| **F393.11** | Familial hypokalaemic periodic paralysis |
| **F137000** | Athetoid cerebral palsy |
| **SN48111** | Paralysis following electric shock |
| **F240000** | Flaccid tetraplegia |
| **F246z00** | Cauda equina syndrome NOS |
| **F221.00** | Spastic hemiplegia |
| **F23y100** | Flaccid infantile cerebral palsy |
| **7L0D100** | Correction of obstetric palsy |
| **8E53.00** | Exercises for paralysis |
| **F152200** | Progressive bulbar palsy |
| **8E54.00** | Exercises for spasticity |
| **SJ24.00** | Cauda equina injury without bony injury |
| **A941.11** | General paralysis of insane |
| **F240.11** | Tetraplegia |
| **F137.11** | Athetoid cerebral palsy |
| **F241000** | Flaccid paraplegia |
| **F240100** | Spastic tetraplegia |
| **F23y400** | Ataxic diplegic cerebral palsy |
| **F23..00** | Congenital cerebral palsy |
| **F244.00** | Monoplegia of upper limb |
| **F230.11** | Paraplegia - congenital |
| **2949.00** | Lower limb spasticity |
| **F241100** | Spastic paraplegia |
| **F2B0.00** | Spastic quadriplegic cerebral palsy |
| **7L0D111** | Correction of Erb's palsy |
| **F230.00** | Congenital diplegia |
| **R014.00** | [D]Transient paralysis of a limb |
| **294B.00** | Worsening limb spasticity |
| **F23y200** | Spastic cerebral palsy |
| **F23..11** | Congenital spastic cerebral palsy |
| **F24yz11** | Specified palsy NEC |
| **294E.00** | Upper limb flaccidity |
| **2835.00** | O/E - paraplegia |
| **Q206111** | Erb-Duchenne paralysis |
| **F393.00** | Familial periodic paralysis |
| **Q206.00** | Brachial plexus palsy due to birth trauma |
| **2992.11** | O/E - spastic gait |
| **F241.00** | Paraplegia |
| **38Gw.00** | Gross Motor Function Classification System Cerebral Palsy |
| **F230000** | Congenital paraplegia |
| **F343000** | Acute radial nerve palsy |
| **Q206z00** | Brachial plexus palsy due to birth trauma NOS |
| **F23y600** | Choreoathetoid cerebral palsy |
| **Q207.00** | Other cranial or peripheral nerve palsy due to birth trauma |
| **F231.00** | Congenital hemiplegia |
| **2992.00** | O/E - gait spastic |
| **F234.00** | Infantile hemiplegia NOS |
| **F246000** | Cauda equina syndrome not affecting bladder |
| **294A.00** | Upper limb spasticity |
| **R012200** | [D]Spastic gait |
| **F233.11** | Congenital spastic foot |
| **Q206000** | Brachial palsy unspecified, due to birth trauma |
| **N12C300** | Lumbar disc prolapse with cauda equina compression |
| **F23y300** | Dyskinetic cerebral palsy |
| **2834.00** | O/E - monoplegia |
| **Q206y00** | Other specified brachial plexus palsy due to birth trauma |
| **F038.00** | Tropical spastic paraplegia |
| **F230z00** | Congenital diplegia NOS |
| **F220.00** | Flaccid hemiplegia |
| **F22..00** | Hemiplegia |
| **F230111** | Spastic diplegic cerebral palsy |
| **F233.00** | Congenital monoplegia |
| **F232.11** | Tetraplegia - congenital |
| **Q206112** | Erb's palsy |
| **283..00** | O/E - paralysis |
| **F23..12** | Infantile cerebral palsy |
| **F36y000** | Supranuclear paralysis |
| **F2B..00** | Cerebral palsy |
| **SJ24000** | Closed injury cauda equina |
| **F245.00** | Monoplegia unspecified |
| **F22z.00** | Hemiplegia NOS |
| **SN48112** | Paralysis following electric shock |
| **2833.00** | O/E - hemiplegia |
| **7L0D114** | L'Episcopo operation for obstetric palsy |
| **F342100** | Tardy ulnar nerve palsy |
| **F24z.00** | Paralysis NOS |
| **F23z.00** | Congenital cerebral palsy NOS |
| **Fyu9.00** | [X]Cerebral palsy and other paralytic syndromes |
| **E260000** | Psychogenic paralysis |
| **F242.00** | Diplegia of upper limbs |
| **F2Bz.00** | Cerebral palsy NOS |
| **N231011** | Massive muscular calcification associated with paraplegia |
| **F2B1.00** | Spastic hemiplegic cerebral palsy |
| **E201400** | Hysterical paralysis |
| **F2By.00** | Other cerebral palsy |
| **F246.00** | Cauda equina syndrome |
| **F23yz00** | Other infantile cerebral palsy NOS |
| **F222.00** | Left hemiplegia |
| **2836.00** | O/E - quadriplegia |
| **F223.00** | Right hemiplegia |
| **R014000** | [D]Transient monoplegia NOS |
| **S114500** | Closed spinal fracture with cauda equina lesion |
| **Fyu9000** | [X]Other infantile cerebral palsy |
| **F243.00** | Monoplegia of lower limb |
| **7L0D115** | Sever operation for Erb's palsy |
| **F221.11** | Spastic foot |
| **F230100** | Cerebral palsy with spastic diplegia |
| **Q207z00** | Cranial or peripheral nerve palsy due to birth trauma NOS |
| **283Z.00** | O/E - paralysis NOS |
| **F23y000** | Ataxic infantile cerebral palsy |
| **G669.00** | Cerebral palsy, not congenital or infantile, acute |

# Peptic ulcer disease (Elixhauser)

| **Read code** | **Description** |
| --- | --- |
| **ZV12C00** | [V] Personal history of gastric ulcer |
| **J121.00** | Chronic duodenal ulcer |
| **14C1.00** | H/O: peptic ulcer |
| **J120200** | Acute duodenal ulcer with perforation |
| **J121200** | Chronic duodenal ulcer with perforation |
| **J11y.00** | Unspecified gastric ulcer |
| **J120y00** | Acute duodenal ulcer unspecified |
| **J11z.00** | Gastric ulcer NOS |
| **J131000** | Chronic peptic ulcer without mention of complication |
| **J120400** | Acute duodenal ulcer with obstruction |
| **J14yz00** | Unspecified gastrojejunal ulcer NOS |
| **J120000** | Acute duodenal ulcer without mention of complication |
| **J12z.00** | Duodenal ulcer NOS |
| **J125.00** | Anti-platelet induced duodenal ulcer |
| **J14..13** | Jejunal ulcer |
| **7627000** | Closure of perforated duodenal ulcer |
| **J110z00** | Acute gastric ulcer NOS |
| **J121z00** | Chronic duodenal ulcer NOS |
| **J13..11** | Stress ulcer NOS |
| **J11..00** | Gastric ulcer - (GU) |
| **14C1.12** | H/O: gastric ulcer |
| **761J.11** | Stomach ulcer operations |
| **J12y.00** | Unspecified duodenal ulcer |
| **J122.00** | Duodenal ulcer disease |
| **J112.00** | Anti-platelet induced gastric ulcer |
| **J13y.00** | Unspecified peptic ulcer |
| **J12yz00** | Unspecified duodenal ulcer NOS |
| **J13y200** | Unspecified peptic ulcer with perforation |
| **14C1.11** | H/O: duodenal ulcer |
| **J111400** | Chronic gastric ulcer with obstruction |
| **761Jy00** | Other specified operation on gastric ulcer |
| **J130.00** | Acute peptic ulcer |
| **J111000** | Chronic gastric ulcer without mention of complication |
| **7627100** | Suture of duodenal ulcer not elsewhere classified |
| **J112z00** | Anti-platelet induced gastric ulcer NOS |
| **761Jz00** | Operation on gastric ulcer NOS |
| **J13y000** | Unspecified peptic ulcer without mention of complication |
| **J130200** | Acute peptic ulcer with perforation |
| **J13..00** | Peptic ulcer - (PU) site unspecified |
| **761J.00** | Operations on gastric ulcer |
| **J13yz00** | Unspecified peptic ulcer NOS |
| **J14y200** | Unspecified gastrojejunal ulcer with perforation |
| **7627z00** | Operation on duodenal ulcer NOS |
| **J140z00** | Acute gastrojejunal ulcer NOS |
| **J110y00** | Acute gastric ulcer unspecified |
| **7627.00** | Operations on duodenal ulcer |
| **J140200** | Acute gastrojejunal ulcer with perforation |
| **J110000** | Acute gastric ulcer without mention of complication |
| **J17y800** | Healed gastric ulcer leaving a scar |
| **J11..12** | Pyloric ulcer |
| **7612500** | Resection of gastric ulcer by cautery |
| **J111211** | Perforated chronic gastric ulcer |
| **J130000** | Acute peptic ulcer without mention of complication |
| **J131z00** | Chronic peptic ulcer NOS |
| **J13y400** | Unspecified peptic ulcer with obstruction |
| **J121000** | Chronic duodenal ulcer without mention of complication |
| **J131y00** | Chronic peptic ulcer unspecified |
| **J12y200** | Unspecified duodenal ulcer with perforation |
| **J123.00** | Duodenal erosion |
| **ZV12711** | [V]Personal history of peptic ulcer |
| **J121400** | Chronic duodenal ulcer with obstruction |
| **ZV12712** | [V]Personal history of duodenal ulcer |
| **J121211** | Perforated chronic duodenal ulcer |
| **J140.00** | Acute gastrojejunal ulcer |
| **J131200** | Chronic peptic ulcer with perforation |
| **J111.00** | Chronic gastric ulcer |
| **J110.00** | Acute gastric ulcer |
| **J124.00** | Recurrent duodenal ulcer |
| **J110400** | Acute gastric ulcer with obstruction |
| **J11z.12** | Multiple gastric ulcers |
| **J11y000** | Unspecified gastric ulcer without mention of complication |
| **J11y200** | Unspecified gastric ulcer with perforation |
| **J111200** | Chronic gastric ulcer with perforation |
| **J141.00** | Chronic gastrojejunal ulcer |
| **J12y400** | Unspecified duodenal ulcer with obstruction |
| **7627y00** | Other specified operation on duodenal ulcer |
| **J14..00** | Gastrojejunal ulcer (GJU) |
| **J120.00** | Acute duodenal ulcer |
| **761J100** | Closure of gastric ulcer NEC |
| **J11yz00** | Unspecified gastric ulcer NOS |
| **J12y000** | Unspecified duodenal ulcer without mention of complication |
| **761J111** | Suture of ulcer of stomach NEC |
| **J12..00** | Duodenal ulcer - (DU) |
| **J131.00** | Chronic peptic ulcer |
| **J130z00** | Acute peptic ulcer NOS |
| **J11..11** | Prepyloric ulcer |
| **J120z00** | Acute duodenal ulcer NOS |
| **J131400** | Chronic peptic ulcer with obstruction |
| **J102000** | Peptic ulcer of oesophagus |
| **J13z.00** | Peptic ulcer NOS |
| **J113.00** | Non steroidal anti inflammatory drug induced gastric ulcer |
| **J111y00** | Chronic gastric ulcer unspecified |
| **J14z.00** | Gastrojejunal ulcer NOS |
| **761J000** | Closure of perforated gastric ulcer |
| **J110200** | Acute gastric ulcer with perforation |
| **J14y.00** | Unspecified gastrojejunal ulcer |
| **7612111** | Balfour excision of gastric ulcer |
| **J11y400** | Unspecified gastric ulcer with obstruction |
| **J121y00** | Chronic duodenal ulcer unspecified |
| **J126.00** | Non steroidal anti inflammatory drug induced duodenal ulcer |
| **J14..12** | Gastrocolic ulcer |
| **J130y00** | Acute peptic ulcer unspecified |
| **J111z00** | Chronic gastric ulcer NOS |

# Peripheral vascular disease (Elixhauser)

| **Read code** | **Description** |
| --- | --- |
| **G713.11** | Ruptured abdominal aortic aneurysm |
| **7A59z00** | Transluminal balloon assisted coil embolisation aneurysm NOS |
| **7A27D00** | Operation on aneurysm of axillary artery |
| **7A47.13** | Other emergency bypass of deep femoral artery |
| **7A48000** | Bypass femoral artery by fem/pop art anast c prosthesis NEC |
| **7A5A000** | Perc translumin stent assis coil embolis three more aneurysm |
| **7A41100** | Bypass iliac artery by iliac/femoral artery anastomosis NEC |
| **7A34F00** | Operation on aneurysm of suprarenal artery NEC |
| **G723300** | Aneurysm of dorsalis pedis artery |
| **G341z00** | Aneurysm of heart NOS |
| **7A41.00** | Other bypass of iliac artery |
| **G720000** | Aneurysm of brachial artery |
| **7A46z00** | Other replacement of aneurysmal femoral/popliteal artery NOS |
| **7A46.11** | Other replacement aneurysmal femoral artery by anastomosis |
| **7A5Bz00** | Other transluminal embolisation of aneurysm NOS |
| **7A16z00** | Other bypass of segment of aorta NOS |
| **4439A** | INTERMITTENT CLAUDICATION |
| **7A1B700** | Endovascular stenting for aorto-uniiliac aneurysm |
| **7A48700** | Bypass popliteal artery by pop/tib a anast c vein graft NEC |
| **4419.00** | AORTIC ANEURYSM |
| **585I000** | Abdominal aortic aneurysm screen ultrasound scan abnormal |
| **7A32000** | Percutaneous transluminal angioplasty of renal artery |
| **7A43.11** | Other open operations on common iliac artery |
| **7A12100** | Bypass bifurc aorta by anastom aorta to femoral artery NEC |
| **7A14y00** | Other replacement of aneurysmal segment of aorta OS |
| **7A45y00** | Emergency replacement aneurysmal femoral/popliteal artery OS |
| **7A10300** | Axillo-unifemoral PTFE bypass graft |
| **G700.00** | Aortic atherosclerosis |
| **G742700** | Embolism and thrombosis of the dorsalis pedis artery |
| **P76z.00** | Peripheral vascular system anomaly NOS |
| **R054z00** | [D]Gangrene NOS |
| **7A59.00** | Transluminal balloon assisted coil embolisation of aneurysm |
| **7A10400** | Bypass aorta anastomosis axillary artery bi femoral arteries |
| **7A35300** | Percutaneous transluminal angioplasty suprarenal artery NEC |
| **7A11311** | Y graft abdominal Aortic aneurysm |
| **7A45.15** | Emergency replacement aneurysmal superficial femoral artery |
| **7A48A00** | Bypass femoral artery by fem/peron a anast c vein graft NEC |
| **G718.00** | Leaking abdominal aortic aneurysm |
| **G73z012** | Vascular claudication |
| **7A48300** | Bypass popliteal artery by pop/pop a anast c vein graft NEC |
| **G72yB00** | Aneurysm of other visceral artery |
| **7A13400** | Emerg replace aneurysm abdom aorta by anast aorta/aorta NEC |
| **7A47200** | Emerg bypass femoral art by fem/pop a anast c vein graft NEC |
| **G742.00** | Embolism and thrombosis of an arm or leg artery |
| **G72y500** | Aneurysm of splenic artery |
| **G72y200** | Aneurysm of internal carotid artery |
| **M271300** | Arterial leg ulcer |
| **G72y300** | Aneurysm of neck artery NOS |
| **G716000** | Thoracoabdominal aortic aneurysm, without mention of rupture |
| **G740.00** | Embolism and thrombosis of the abdominal aorta |
| **G742600** | Embolism and thrombosis of the anterior tibial artery |
| **7A31300** | Operation on aneurysm of renal artery |
| **G73..13** | Peripheral ischaemia |
| **G726.00** | Dissection of renal artery |
| **G742800** | Embolism and thrombosis of the posterior tibial artery |
| **7A21400** | Operation on aneurysm of carotid artery |
| **G73zz00** | Peripheral vascular disease NOS |
| **7A43200** | Operation on aneurysm of iliac artery NEC |
| **G731.00** | Thromboangiitis obliterans |
| **7A47.16** | Other emergency bypass of femoral artery |
| **G72..00** | Other aneurysm |
| **7A16y00** | Other specified other bypass of segment of aorta |
| **G711.00** | Thoracic aortic aneurysm which has ruptured |
| **7A42111** | Endarterectomy of common iliac artery NEC |
| **G73y.00** | Other specified peripheral vascular disease |
| **7A23800** | Percutaneous coil embolisation of cerebral artery aneurysm |
| **G73z000** | Intermittent claudication |
| **7A28100** | Percutaneous transluminal angioplasty of brachial artery |
| **G73..00** | Other peripheral vascular disease |
| **7A1Cz00** | Translum ins stent graft for aneurysmal segment of aorta NOS |
| **G720.00** | Aneurysm of artery of arm |
| **G716.00** | Aortic aneurysm without mention of rupture NOS |
| **7A11.00** | Replacement of aneurysmal bifurcation of aorta |
| **7A48C00** | Bypass femoral artery by femoral/femoral art anastomosis NEC |
| **7A12000** | Emerg bypass bifurc aorta by anast aorta to femoral artery |
| **7A22000** | Percutaneous transluminal angioplasty of carotid artery |
| **7A58y00** | Other specified transluminal coil embolisation of aneurysm |
| **7A46.15** | Other replacement of aneurysmal superficial femoral artery |
| **G701011** | ARAS - Atherosclerotic renal artery stenosis |
| **7A11000** | Emerg repl aneurysm bifurc aorta by anast aorta to fem art |
| **7A27C00** | Operation on aneurysm of subclavian artery |
| **G732.00** | Peripheral gangrene |
| **16I..00** | Claudication distance |
| **7A5Az00** | Transluminal stent assisted coil embolisation aneurysm NOS |
| **7A43011** | Repair of common iliac artery NEC |
| **G722100** | Aneurysm of external iliac artery |
| **G72z.00** | Aneurysm NOS |
| **7A17100** | Revision of prosthesis of bifurcation of aorta |
| **G742z00** | Peripheral arterial embolism and thrombosis NOS |
| **G742400** | Embolism and thrombosis of the femoral artery |
| **7A42011** | Endarterectomy and patch repair of common iliac artery |
| **7A14100** | Replace aneurysm thoracic aorta by anast of aorta/aorta NEC |
| **7A44000** | Percutaneous transluminal angioplasty of iliac artery |
| **7A35100** | Percutaneous transluminal angioplasty sup mesenteric art NEC |
| **7A58000** | Percutaneous transluminal coil embolisation aneurysm small |
| **9m10.00** | Peripheral vascular disease monitoring first letter |
| **7A13100** | Emerg replace aneurysm thor aorta by anastom aorta to aorta |
| **G74yz00** | Embolism and thrombosis of other arteries NOS |
| **7A1B500** | Endovascular stenting of aorto-uniiliac aneurysm |
| **G723z00** | Aneurysm of leg artery NOS |
| **G72y800** | Aneurysm of superior mesenteric artery |
| **7A40000** | Emerg replace aneurysm iliac art by iliac/femoral art anast |
| **7A45200** | Emerg replace aneurysm fem art by fem/pop anast c vein graft |
| **7A45.12** | Emergency replacement of aneurysmal common femoral artery |
| **7A27E00** | Operation on aneurysm of brachial artery |
| **7A4B100** | Percutaneous transluminal angioplasty of popliteal artery |
| **7A1BB00** | Endovascular ins stent for aortic dissection in any position |
| **7A14.00** | Other replacement of aneurysmal segment of aorta |
| **G74y000** | Embolism and/or thrombosis of the common iliac artery |
| **7A19400** | Operation on aneurysm of aorta NEC |
| **7A13411** | Tube graft abdominal Aortic aneurysm (emergency) |
| **7A45.00** | Emergency replacement of aneurysmal femoral/popliteal artery |
| **7A1B000** | Endovascular stenting infrarenal abdominal aortic aneurysm |
| **7A34D00** | Operation on aneurysm of superior mesenteric artery NEC |
| **7A11200** | Emerg repl aneurysm bifurc aorta by anast aorta to iliac a |
| **G720200** | Aneurysm of ulnar artery |
| **Gyu7200** | [X]Aortic aneurysm of unspecified site, nonruptured |
| **7A45700** | Emerg replace aneurysm pop art by pop/tib anast c vein graft |
| **14NB.00** | H/O: Peripheral vascular disease procedure |
| **G73..11** | Peripheral ischaemic vascular disease |
| **7A46D00** | Replace aneurysm popliteal artery by pop/fem anastomosis NEC |
| **7A1Bz00** | Transluminal operations on aneurysmal segment of aorta NOS |
| **G741.00** | Embolism and thrombosis of the thoracic aorta |
| **7A11211** | Y graft of abdominal Aortic aneurysm (emergency) |
| **7A40.11** | Replacement of aneurysmal iliac artery by anastomosis |
| **7A53400** | Operation on aneurysm of artery NEC |
| **7A12.00** | Other bypass of bifurcation of aorta |
| **7A14.11** | Aortic aneurysm repair |
| **7A12z00** | Other bypass of bifurcation of aorta NOS |
| **G74y700** | Embolism and thrombosis of the axillary artery |
| **662U.00** | Peripheral vascular disease monitoring |
| **7A1C200** | Endov insertion of stent graft for thoracic aortic aneurysm |
| **7A54000** | Percutaneous transluminal angioplasty of artery NEC |
| **7A28000** | Percutaneous transluminal angioplasty of subclavian artery |
| **7A10100** | Bypass aorta by anastomosis axillary to femoral artery NEC |
| **7A19200** | Open embolectomy of bifurcation of aorta |
| **G742100** | Embolism and thrombosis of the radial artery |
| **7A47.12** | Other emergency bypass of common femoral artery |
| **14AE.00** | H/O: aortic aneurysm |
| **G722.00** | Aneurysm of iliac artery |
| **P766.00** | Peripheral arterio-venous aneurysm |
| **7A15.00** | Other emergency bypass of segment of aorta |
| **G722z00** | Aneurysm of iliac artery NOS |
| **7A1BC00** | Endovas insert stent for aortic aneurysm of bifurcation NEC |
| **7A47y00** | Other emergency bypass of femoral or popliteal artery OS |
| **G742500** | Embolism and thrombosis of the popliteal artery |
| **7A48.15** | Other bypass of popliteal artery |
| **7A4A500** | Operation on aneurysm of femoral artery NEC |
| **7A28200** | Percutaneous transluminal angioplasty of vertebral artery |
| **7A14411** | Tube graft of Abdominal aortic aneurysm |
| **P7y0100** | Congenital cerebral arteriovenous aneurysm |
| **7A11z00** | Replacement of aneurysmal bifurcation of aorta NOS |
| **G742000** | Embolism and thrombosis of the brachial artery |
| **7A13300** | Emerg replace aneurysm infrarenal aorta by anast aorta/aorta |
| **7A1C500** | Endovas insertion of stent graft for aorto-uniiliac aneurysm |
| **7A45000** | Emerg replace aneurysm fem art by fem/pop art anast c prosth |
| **7A16.00** | Other bypass of segment of aorta |
| **G73yz00** | Other specified peripheral vascular disease NOS |
| **7A1C.00** | Translum insert stent graft for aneurysmal segment of aorta |
| **G71z.00** | Aortic aneurysm NOS |
| **g71..00** | Aortic aneurysm |
| **7A5B.00** | Other transluminal embolisation of aneurysm |
| **G72y100** | Aneurysm of external carotid artery |
| **G673.00** | Cerebral aneurysm, nonruptured |
| **G74y300** | Embolism and thrombosis of the iliac artery unspecified |
| **7A5By00** | Other specified other transluminal embolisation of aneurysm |
| **7A48.11** | Other bypass of femoral or popliteal artery by anastomosis |
| **7A1B.00** | Transluminal operations on aneurysmal segment of aorta |
| **G74y600** | Embolism and thrombosis of the splenic artery |
| **7A6H400** | Percutaneous transluminal angioplasty of vascular graft |
| **G70..00** | Atherosclerosis |
| **7A14z00** | Other replacement of aneurysmal segment of aorta NOS |
| **7A49900** | Reconstruction of popliteal artery with vein graft |
| **7A48.12** | Other bypass of common femoral artery |
| **7A46y00** | Other replacement of aneurysmal femoral/popliteal artery OS |
| **Gyu7100** | [X]Aortic aneurysm of unspecified site, ruptured |
| **G72y400** | Aneurysm of subclavian artery |
| **7A26D00** | Reconstruction of subclavian artery with vein graft |
| **7A1C000** | Endovas ins stent graft for infrarenal abdom aortic aneurysm |
| **G711.11** | Ruptured thoracic aortic aneurysm |
| **2I16.00** | O/E - gangrene |
| **G702.00** | Extremity artery atheroma |
| **G723000** | Aneurysm of femoral artery |
| **7A5A.00** | Transluminal stent assisted coil embolisation of aneurysm |
| **7A44211** | Arteriography of common iliac artery |
| **7A1BD00** | Endovascular insertion of stent for aorto-uniiliac aneurysm |
| **7A14000** | Replace aneurysm ascend aorta by anast of aorta/aorta NEC |
| **G731000** | Buerger's disease |
| **7A14400** | Replace aneurysm abdominal aorta by anast aorta to aorta NEC |
| **G743.00** | Embolism and thrombosis of other and unspec parts aorta |
| **G73..12** | Ischaemia of legs |
| **G712.00** | Thoracic aortic aneurysm without mention of rupture |
| **P767.00** | Congenital peripheral aneurysm |
| **G722200** | Aneurysm of internal iliac artery |
| **SP12z00** | Peripheral vascular complications of care NOS |
| **G72yz00** | Other aneurysm NOS |
| **G732200** | Gangrene of finger |
| **G720100** | Aneurysm of radial artery |
| **G727.00** | Dissection of iliac artery |
| **7A48y00** | Other bypass of femoral artery or popliteal artery OS |
| **G732300** | Gangrene of thumb |
| **Gyu7300** | [X]Aneurysm of other specified arteries |
| **7A47.14** | Other emergency bypass of popliteal artery |
| **7A48.14** | Other bypass of femoral artery |
| **7A41300** | Bypass iliac artery by femoral/femoral art anastomosis NEC |
| **7A40.00** | Replacement of aneurysmal iliac artery |
| **7A47.00** | Other emergency bypass of femoral artery or popliteal artery |
| **G723400** | Aneurysm of posterior tibial artery |
| **G74z.00** | Arterial embolism and thrombosis NOS |
| **7A13y00** | Emergency replacement of aneurysmal segment of aorta OS |
| **Gyu7000** | [X]Atherosclerosis of other arteries |
| **7A58300** | Percutaneous transluminal coil embolisation aneurysm giant |
| **7A46100** | Replace aneurysm pop art by pop/pop art anastom c prosth NEC |
| **7A41000** | Emerg bypass iliac art by iliac/femoral art anastomosis NEC |
| **7A11y00** | Replacement of aneurysmal bifurcation of aorta OS |
| **G710.00** | Dissecting aortic aneurysm |
| **7A13000** | Emerg replace aneurysm asc aorta by anastom aorta to aorta |
| **7A35200** | Percutaneous transluminal angioplasty inf mesenteric art NEC |
| **7A41D00** | Bypass iliac artery by iliac/iliac artery anastomosis NEC |
| **7A4B000** | Percutaneous transluminal angioplasty of femoral artery |
| **7A40200** | Emerg replace aneurysmal iliac artery by fem/fem art anast |
| **G728.00** | Dissection of artery of lower extremity |
| **G72A.00** | Dissection of other specified arteries |
| **7A41z00** | Other bypass of iliac artery NOS |
| **7A5B000** | Percutaneous translumin liquid polymer embolisation aneurysm |
| **7A1Cy00** | OS translum ins stent graft for aneurysmal segment of aorta |
| **7A48800** | Bypass femoral artery by fem/peron a anast c prosthesis NEC |
| **7A52300** | Repair of artery using vein graft NEC |
| **4459N** | GANGRENE |
| **G734.00** | Peripheral arterial disease |
| **66f3.00** | Aortic aneurysm monitoring |
| **G713.00** | Abdominal aortic aneurysm which has ruptured |
| **7A46.00** | Other replacement of aneurysmal femoral artery |
| **G714100** | Inflammatory abdominal aortic aneurysm |
| **7A44.11** | Transluminal operations on common iliac artery |
| **R054000** | [D]Gangrene, spreading cutaneous |
| **7A58z00** | Transluminal coil embolisation of aneurysm NOS |
| **G74y500** | Embolism and thrombosis of the subclavian artery |
| **7A42.11** | Reconstruction of common iliac artery |
| **G72B.00** | Dissection of artery |
| **G701.00** | Renal artery atherosclerosis |
| **7A49800** | Reconstruction of femoral artery with vein graft |
| **9m11.00** | Peripheral vascular disease monitoring second letter |
| **G742300** | Embolism and thrombosis of an arm artery NOS |
| **7A40A00** | Replace aneurysm iliac art by aorta/ext iliac art anast NEC |
| **7A40z00** | Replacement of aneurysmal iliac artery NOS |
| **7A41200** | Emerg bypass iliac artery by femoral/femoral art anast NEC |
| **G723.00** | Aneurysm of leg artery |
| **7A1B900** | Endovascular insertion stent for suprarenal aortic aneurysm |
| **7A46C00** | Replace aneurysm fem artery by fem/fem art anastomosis NEC |
| **G731100** | Presenile gangrene |
| **7A10200** | Axillo-bifemoral bypass graft |
| **G714000** | Juxtarenal aortic aneurysm |
| **G721.00** | Aneurysm of renal artery |
| **G714.00** | Abdominal aortic aneurysm without mention of rupture |
| **7A48400** | Bypass femoral artery by fem/tib art anast c prosthesis NEC |
| **G73z.00** | Peripheral vascular disease NOS |
| **G742900** | Embolism and thrombosis of a leg artery NOS |
| **G70z.00** | Arteriosclerotic vascular disease NOS |
| **7A58100** | Percutaneous transluminal coil embolisation aneurysm medium |
| **7A11300** | Replace aneurysm bifurc aorta by anast aorta to iliac artery |
| **7A48.00** | Other bypass of femoral artery or popliteal artery |
| **7A4A400** | Ligation of aneurysm of popliteal artery |
| **7A46000** | Replace aneurysm fem art by fem/pop art anastom c prosth NEC |
| **4459TE** | GANGRENE TOE |
| **7A13.00** | Emergency replacement of aneurysmal segment of aorta |
| **G71..00** | Aortic aneurysm |
| **G714300** | Aneurysm of suprarenal aorta |
| **4439GD** | PVD (PERIPHERAL VASCULAR DISEASE) |
| **6075GM** | SCROTAL GANGRENE |
| **Gyu7400** | [X]Other specified peripheral vascular diseases |
| **7A41y00** | Other specified other bypass of iliac artery |
| **7A13.11** | Emergency repair of aortic aneurysm |
| **7A41.11** | Other bypass of iliac artery by anastomosis |
| **7A41900** | Bypass common iliac artery by aorta/com iliac art anast NEC |
| **G713000** | Ruptured suprarenal aortic aneurysm |
| **7A47600** | Emerg bypass femoral art by fem/tib a anast c vein graft NEC |
| **G732000** | Gangrene of toe |
| **7A1B300** | Endovascular stenting of aortic dissection in any position |
| **7A46.14** | Other replacement of aneurysmal popliteal artery |
| **G72yA00** | Aneurysm of hepatic artery |
| **G421.00** | Aneurysm of pulmonary artery |
| **G341100** | Other cardiac wall aneurysm |
| **G74y.00** | Embolism and thrombosis of other specified artery |
| **7A47700** | Emerg bypass pop art by pop/tib art anast c vein graft NEC |
| **7A1B600** | Endovascular stenting for aortic aneurysm of bifurcation NEC |
| **7A1A100** | Percutaneous transluminal angioplasty of aorta NEC |
| **G74..00** | Arterial embolism and thrombosis |
| **4459CR** | ULCER WITH GANGRENE |
| **7A47C00** | Emerg bypass femoral artery by fem/fem art anastomosis NEC |
| **G722000** | Aneurysm of common iliac artery |
| **P76..00** | Other peripheral vascular system anomalies |
| **G723200** | Aneurysm of anterior tibial artery |
| **9m12.00** | Peripheral vascular disease monitoring third letter |
| **G720z00** | Aneurysm of arm artery NOS |
| **7A46300** | Replace aneurysm pop art by pop/pop a anast c vein graft NEC |
| **7A48z00** | Other bypass of femoral artery or popliteal artery NOS |
| **9N4h.00** | DNA - Did not attend peripheral vascular disease clinic |
| **G714.11** | AAA - Abdominal aortic aneurysm without mention of rupture |
| **4430G** | SYMMETRICAL GANGRENE EXTREMITIES |
| **G732400** | Gangrene of hand |
| **G72y000** | Aneurysm of common carotid art |
| **2456.00** | O/E - arterial wall - aneurysm |
| **G72y600** | Aneurysm of axillary artery |
| **G702z00** | Extremity artery atheroma NOS |
| **7A48.16** | Other bypass of superficial femoral artery |
| **7A1BA00** | Endovascular insertion of stent for thoracic aortic aneurysm |
| **G72y.00** | Aneurysm of other artery |
| **7A40y00** | Other specified replacement of aneurysmal iliac artery |
| **R054.00** | [D]Gangrene |
| **7A15z00** | Other emergency bypass of segment of aorta NOS |
| **G72y900** | Aneurysm of inferior mesenteric artery |
| **7A11100** | Replace aneurysm bifurc aorta by anast aorta to femoral art |
| **G715.00** | Ruptured aortic aneurysm NOS |
| **7A1B100** | Endovascular stenting of suprarenal aortic aneurysm |
| **7A47z00** | Other emergency bypass of femoral or popliteal artery NOS |
| **G723100** | Aneurysm of popliteal artery |
| **G72y700** | Aneurysm of coeliac artery |
| **G732100** | Gangrene of foot |
| **7A1B800** | Endovascul insert stent infrarenal abdominal aortic aneurysm |
| **G73z011** | Claudication |
| **G74y900** | Embolism and thrombosis of the hepatic artery |
| **7A45D00** | Emerg replace aneurysm pop artery by pop/fem art anastomosis |
| **7A47.15** | Other emergency bypass of superficial femoral artery |
| **7A1B200** | Endovascular stenting of thoracic aortic aneurysm |
| **9m1..00** | Peripheral vascular disease monitoring invitation |
| **7A15y00** | Other specified other emergency bypass of segment of aorta |
| **7A45.14** | Emergency replacement of aneurysmal popliteal artery |
| **7A34E00** | Operation on aneurysm of inferior mesenteric artery NEC |
| **G715000** | Thoracoabdominal aortic aneurysm, ruptured |
| **G70y000** | Carotid artery atherosclerosis |
| **4410N** | DISSECTION AORTA |
| **7A23z00** | Operation on cerebral artery/ circle of Willis aneurysm NOS |
| **7A1C300** | Endov ins stent graft for aortic dissection in any position |
| **4459FT** | GANGRENE FOOT |
| **7A26F00** | Reconstruction of brachial artery with vein graft |
| **Gyu7800** | [X]Aneurysm of aorta in diseases classified elsewhere |
| **7A1C100** | Endovas insert of stent graft for suprarenal aortic aneurysm |
| **7A47300** | Emerg bypass pop art by pop/pop art anast c vein graft NEC |
| **7A12y00** | Other specified other bypass of bifurcation of aorta |
| **7A5A200** | Perc trans stent assisted coil embolisation single aneurysm |
| **7A28C00** | Percutaneous transluminal angioplasty of axillary artery |
| **G731z00** | Thromboangiitis obliterans NOS |
| **7A59200** | Perc translum balloon assist coil embolisat single aneurysm |
| **G714200** | Infrarenal abdominal aortic aneurysm |
| **7A35000** | Percutaneous transluminal angioplasty of coeliac artery NEC |
| **G74y800** | Embolism and thrombosis of the coeliac artery |
| **G723500** | Ruptured popliteal artery aneurysm |
| **7A34K00** | Operation on aneurysm visceral branch of abdominal aorta NEC |
| **7A58.00** | Transluminal coil embolisation of aneurysm |
| **7A13z00** | Emergency replacement of aneurysmal segment of aorta NOS |
| **7A43111** | Open embolectomy of common iliac artery |
| **M271400** | Mixed venous and arterial leg ulcer |
| **G723600** | Post radiological femoral false aneurysm |
| **P72z100** | Congenital aneurysm of aorta |
| **G742200** | Embolism and thrombosis of the ulnar artery |
| **G76A.00** | Arterial insufficiency |

# Psychoses (Elixhauser)

| **Read code** | **Description** |
| --- | --- |
| **E11y000** | Unspecified manic-depressive psychoses |
| **E116400** | Mixed bipolar affective disorder, severe, with psychosis |
| **E1...00** | Non-organic psychoses |
| **E131.00** | Acute hysterical psychosis |
| **E107.11** | Cyclic schizophrenia |
| **Eu2z.11** | [X]Psychosis NOS |
| **Eu20511** | [X]Chronic undifferentiated schizophrenia |
| **E103300** | Acute exacerbation of subchronic paranoid schizophrenia |
| **E110600** | Single manic episode in full remission |
| **E03y300** | Unspecified puerperal psychosis |
| **E11z000** | Unspecified affective psychoses NOS |
| **E103000** | Unspecified paranoid schizophrenia |
| **Eu24.13** | [X]Induced psychotic disorder |
| **E102100** | Subchronic catatonic schizophrenia |
| **E114000** | Bipolar affective disorder, currently manic, unspecified |
| **E102z00** | Catatonic schizophrenia NOS |
| **6657.00** | On lithium |
| **Eu31500** | [X]Bipolar affect dis cur epi severe depres with psyc symp |
| **Eu33311** | [X]Endogenous depression with psychotic symptoms |
| **Eu31.11** | [X]Manic-depressive illness |
| **E13y100** | Brief reactive psychosis |
| **E116300** | Mixed bipolar affective disorder, severe, without psychosis |
| **1BH2.00** | Ideas of reference |
| **Eu31y00** | [X]Other bipolar affective disorders |
| **E110100** | Single manic episode, mild |
| **E105.00** | Latent schizophrenia |
| **Eu30z00** | [X]Manic episode, unspecified |
| **E13yz00** | Other reactive psychoses NOS |
| **Eu2..00** | [X]Schizophrenia, schizotypal and delusional disorders |
| **E114300** | Bipolar affect disord, currently manic, severe, no psychosis |
| **E117300** | Unspecified bipolar affective disorder, severe, no psychosis |
| **E117400** | Unspecified bipolar affective disorder,severe with psychosis |
| **Eu20200** | [X]Catatonic schizophrenia |
| **E117500** | Unspecified bipolar affect disord, partial/unspec remission |
| **E100.00** | Simple schizophrenia |
| **Eu21.13** | [X]Latent schizophrenia |
| **E101500** | Hebephrenic schizophrenia in remission |
| **Eu30.11** | [X]Bipolar disorder, single manic episode |
| **E100000** | Unspecified schizophrenia |
| **E102.00** | Catatonic schizophrenia |
| **Eu23100** | [X]Acute polymorphic psychot disord with symp of schizophren |
| **ZRhi.00** | Rust inventory of schizotypal cognitions |
| **Eu25z00** | [X]Schizoaffective disorder, unspecified |
| **1BH..11** | Delusion |
| **E111400** | Recurrent manic episodes, severe, with psychosis |
| **E12z.00** | Paranoid psychosis NOS |
| **Eu25200** | [X]Schizoaffective disorder, mixed type |
| **Eu13500** | [X]Mental & behav dis due to seds/hypntcs: psychotic disordr |
| **E00y.00** | Other senile and presenile organic psychoses |
| **E00..00** | Senile and presenile organic psychotic conditions |
| **E103200** | Chronic paranoid schizophrenia |
| **Eu23z00** | [X]Acute and transient psychotic disorder, unspecified |
| **E111000** | Recurrent manic episodes, unspecified |
| **E110.00** | Manic disorder, single episode |
| **Eu25211** | [X]Cyclic schizophrenia |
| **Eu20000** | [X]Paranoid schizophrenia |
| **E11..00** | Affective psychoses |
| **Eu23000** | [X]Acute polymorphic psychot disord without symp of schizoph |
| **Eu25y00** | [X]Other schizoaffective disorders |
| **Eu23211** | [X]Brief schizophreniform disorder |
| **E141.00** | Disintegrative psychosis |
| **E02..00** | Drug psychoses |
| **E10y.11** | Cenesthopathic schizophrenia |
| **Eu23200** | [X]Acute schizophrenia-like psychotic disorder |
| **1BH..00** | Delusions |
| **E116200** | Mixed bipolar affective disorder, moderate |
| **E117000** | Unspecified bipolar affective disorder, unspecified |
| **E100400** | Acute exacerbation of chronic schizophrenia |
| **Eu20212** | [X]Schizophrenic catalepsy |
| **E100z00** | Simple schizophrenia NOS |
| **Eu30212** | [X]Mania with mood-incongruent psychotic symptoms |
| **E1z..00** | Non-organic psychosis NOS |
| **E03z.00** | Transient organic psychoses NOS |
| **Eu30100** | [X]Mania without psychotic symptoms |
| **E212000** | Unspecified schizoid personality disorder |
| **E03yz00** | Other transient organic psychoses NOS |
| **Eu10514** | [X]Alcoholic psychosis NOS |
| **E10yz00** | Other schizophrenia NOS |
| **E141100** | Residual disintegrative psychoses |
| **E111100** | Recurrent manic episodes, mild |
| **E101.00** | Hebephrenic schizophrenia |
| **E0y..00** | Other specified organic psychoses |
| **E101z00** | Hebephrenic schizophrenia NOS |
| **Eu02z12** | [X] Presenile psychosis NOS |
| **E02y.00** | Other drug psychoses |
| **E110000** | Single manic episode, unspecified |
| **E114z00** | Bipolar affective disorder, currently manic, NOS |
| **E11y300** | Other mixed manic-depressive psychoses |
| **E114.00** | Bipolar affective disorder, currently manic |
| **E13z.11** | Psychotic episode NOS |
| **E107100** | Subchronic schizo-affective schizophrenia |
| **E03y.00** | Other transient organic psychoses |
| **E212200** | Schizotypal personality |
| **Eu04.13** | [X]Acute / subacute infective psychosis |
| **E134.00** | Psychogenic paranoid psychosis |
| **E111200** | Recurrent manic episodes, moderate |
| **E116600** | Mixed bipolar affective disorder, in full remission |
| **E116z00** | Mixed bipolar affective disorder, NOS |
| **E0z..00** | Organic psychoses NOS |
| **Eu84013** | [X]Infantile psychosis |
| **Eu23112** | [X]Cycloid psychosis with symptoms of schizophrenia |
| **Eu31y11** | [X]Bipolar II disorder |
| **E11yz00** | Other and unspecified manic-depressive psychoses NOS |
| **146D.00** | H/O: manic depressive disorder |
| **E12..00** | Paranoid states |
| **1BH1.00** | Grandiose delusions |
| **Eu0z.12** | [X]Symptomatic psychosis NOS |
| **E02z.00** | Drug psychosis NOS |
| **Eu31900** | [X]Bipolar affective disorder type II |
| **E110z00** | Manic disorder, single episode NOS |
| **E14y000** | Atypical childhood psychoses |
| **E101400** | Acute exacerbation of chronic hebephrenic schizophrenia |
| **E14yz00** | Other childhood psychoses NOS |
| **Eu14500** | [X]Mental & behav dis due to use cocaine: psychotic disorder |
| **E105500** | Latent schizophrenia in remission |
| **Eu31z00** | [X]Bipolar affective disorder, unspecified |
| **Eu25000** | [X]Schizoaffective disorder, manic type |
| **Eu20z00** | [X]Schizophrenia, unspecified |
| **Eu20011** | [X]Paraphrenic schizophrenia |
| **38C1400** | Assessment of cause of psychotic and behavioural symptoms |
| **E100300** | Acute exacerbation of subchronic schizophrenia |
| **Eu03.11** | [X]Korsakov's psychosis, nonalcoholic |
| **E116.00** | Mixed bipolar affective disorder |
| **E107.00** | Schizo-affective schizophrenia |
| **Eu20y13** | [X]Schizophrenifrm psychos NOS |
| **E10y100** | Coenesthopathic schizophrenia |
| **E111.00** | Recurrent manic episodes |
| **Eu21.16** | [X]Pseudoneurotic schizophrenia |
| **E102000** | Unspecified catatonic schizophrenia |
| **Eu31400** | [X]Bipol aff disord, curr epis sev depress, no psychot symp |
| **Eu20211** | [X]Catatonic stupor |
| **Eu3z.11** | [X]Affective psychosis NOS |
| **E14y.00** | Other childhood psychoses |
| **Eu25z11** | [X]Schizoaffective psychosis NOS |
| **E03..00** | Transient organic psychoses |
| **E10z.00** | Schizophrenia NOS |
| **E111300** | Recurrent manic episodes, severe without mention psychosis |
| **Eu30z11** | [X]Mania NOS |
| **Eu21.14** | [X]Prepsychotic schizophrenia |
| **Eu23300** | [X]Other acute predominantly delusional psychotic disorders |
| **6657.11** | Lithium monitoring |
| **69DD.00** | Antipsychotic medication physical health check |
| **Eu20300** | [X]Undifferentiated schizophrenia |
| **E04y.00** | Other specified chronic organic psychoses |
| **E02yz00** | Other drug psychoses NOS |
| **E106.00** | Residual schizophrenia |
| **E100100** | Subchronic schizophrenia |
| **Eu30200** | [X]Mania with psychotic symptoms |
| **Eu23012** | [X]Cycloid psychosis |
| **E105000** | Unspecified latent schizophrenia |
| **E11zz00** | Other affective psychosis NOS |
| **6657.12** | Started lithium |
| **Eu23z11** | [X]Brief reactive psychosis NOS |
| **E110.11** | Hypomanic psychoses |
| **212T.00** | Psychosis, schizophrenia + bipolar affective disord resolved |
| **E103z00** | Paranoid schizophrenia NOS |
| **E14..00** | Psychoses with origin in childhood |
| **13L3.12** | Schizophrenic child |
| **E130.00** | Reactive depressive psychosis |
| **Eu23212** | [X]Brief schizophrenifrm psych |
| **E107500** | Schizo-affective schizophrenia in remission |
| **Eu84111** | [X]Atypical childhood psychosis |
| **Eu84312** | [X]Disintegrative psychosis |
| **1BH0.00** | Delusion of persecution |
| **Eu25212** | [X]Mixed schizophrenic and affective psychosis |
| **Eu23z12** | [X]Reactive psychosis |
| **E01y.00** | Other alcoholic psychosis |
| **E101000** | Unspecified hebephrenic schizophrenia |
| **212X.00** | Psychosis resolved |
| **E111z00** | Recurrent manic episode NOS |
| **E10y000** | Atypical schizophrenia |
| **E114100** | Bipolar affective disorder, currently manic, mild |
| **Eu20111** | [X]Disorganised schizophrenia |
| **ZV11112** | [V]Personal history of manic-depressive psychosis |
| **E100.11** | Schizophrenia simplex |
| **Eu84512** | [X]Schizoid disorder of childhood |
| **E117100** | Unspecified bipolar affective disorder, mild |
| **Eu12500** | [X]Mental & behav dis due to cannabinoids: psychotic disordr |
| **E10y.00** | Other schizophrenia |
| **E113400** | Recurrent major depressive episodes, severe, with psychosis |
| **E114400** | Bipolar affect disord, currently manic,severe with psychosis |
| **Eu20214** | [X]Schizophrenic flexibilatis cerea |
| **Eu31200** | [X]Bipolar affect disorder cur epi manic with psychotic symp |
| **Eu21.18** | [X]Schizotypal personality disorder |
| **225F.00** | O/E - delusion of persecution |
| **Eu25012** | [X]Schizophreniform psychosis, manic type |
| **Eu23214** | [X]Schizophrenic reaction |
| **E117z00** | Unspecified bipolar affective disorder, NOS |
| **Eu2z.00** | [X]Unspecified nonorganic psychosis |
| **E11..11** | Bipolar psychoses |
| **E107000** | Unspecified schizo-affective schizophrenia |
| **Eu2y.00** | [X]Other nonorganic psychotic disorders |
| **E117200** | Unspecified bipolar affective disorder, moderate |
| **E11y.00** | Other and unspecified manic-depressive psychoses |
| **E107z00** | Schizo-affective schizophrenia NOS |
| **Eu23312** | [X]Psychogenic paranoid psychosis |
| **1S42.00** | Manic mood |
| **E112400** | Single major depressive episode, severe, with psychosis |
| **Eu25011** | [X]Schizoaffective psychosis, manic type |
| **Eu15500** | [X]Mental/behav dis oth stims inc caffeine: psychotic dis |
| **Eu44.14** | [X]Hysterical psychosis |
| **E100500** | Schizophrenia in remission |
| **E120.00** | Simple paranoid state |
| **1BH3.00** | Paranoid ideation |
| **Eu30.00** | [X]Manic episode |
| **E115400** | Bipolar affect disord, now depressed, severe with psychosis |
| **Eu84314** | [X]Symbiotic psychosis |
| **Eu32311** | [X]Single episode of major depression and psychotic symptoms |
| **E104.00** | Acute schizophrenic episode |
| **Eu30211** | [X]Mania with mood-congruent psychotic symptoms |
| **E212z00** | Schizoid personality disorder NOS |
| **ZRby100** | Profile of mood states, bipolar |
| **E117600** | Unspecified bipolar affective disorder, in full remission |
| **Eu16500** | [X]Mental & behav dis due to hallucinogens: psychotic disord |
| **Eu2y.11** | [X]Chronic hallucinatory psychosis |
| **8HHs.00** | Referral to psychosis early intervention service |
| **Eu21.11** | [X]Latent schizophrenic reaction |
| **286..11** | Poor insight into psychotic condition |
| **212V.00** | Bipolar affective disorder resolved |
| **E110400** | Single manic episode, severe, with psychosis |
| **R20z.00** | [D]Senility, without psychosis NOS |
| **E102500** | Catatonic schizophrenia in remission |
| **Eu20311** | [X]Atypical schizophrenia |
| **146H.00** | H/O: psychosis |
| **E116500** | Mixed bipolar affective disorder, partial/unspec remission |
| **E121.00** | Chronic paranoid psychosis |
| **Eu20y00** | [X]Other schizophrenia |
| **E110200** | Single manic episode, moderate |
| **E103400** | Acute exacerbation of chronic paranoid schizophrenia |
| **1464.00** | H/O: schizophrenia |
| **Eu20y12** | [X]Schizophreniform disord NOS |
| **Eu31700** | [X]Bipolar affective disorder, currently in remission |
| **E1y..00** | Other specified non-organic psychoses |
| **E00z.00** | Senile or presenile psychoses NOS |
| **E114600** | Bipolar affective disorder, currently manic, full remission |
| **Eu22000** | [X]Delusional disorder |
| **E13y.00** | Other reactive psychoses |
| **Eu25.00** | [X]Schizoaffective disorders |
| **E10..00** | Schizophrenic disorders |
| **Eu20600** | [X]Simple schizophrenia |
| **E117.00** | Unspecified bipolar affective disorder |
| **Eu31911** | [X]Bipolar II disorder |
| **Eu21.15** | [X]Prodromal schizophrenia |
| **Eu0z.11** | [X]Organic psychosis NOS |
| **E114.11** | Manic-depressive - now manic |
| **E212.00** | Schizoid personality disorder |
| **E13..00** | Other nonorganic psychoses |
| **Eu23y00** | [X]Other acute and transient psychotic disorders |
| **Eu31.00** | [X]Bipolar affective disorder |
| **E103500** | Paranoid schizophrenia in remission |
| **Eu05200** | [X]Organic delusional [schizophrenia-like] disorder |
| **Eu20100** | [X]Hebephrenic schizophrenia |
| **Eu18500** | [X]Mental & behav dis due to vol solvents: psychotic disordr |
| **ZS7C611** | Schizophrenic language |
| **Eu33316** | [X]Recurrent severe episodes/reactive depressive psychosis |
| **E04z.00** | Chronic organic psychosis NOS |
| **E011100** | Korsakov's alcoholic psychosis with peripheral neuritis |
| **E116100** | Mixed bipolar affective disorder, mild |
| **E00y.11** | Presbyophrenic psychosis |
| **ZV11000** | [V]Personal history of schizophrenia |
| **E11z.00** | Other and unspecified affective psychoses |
| **E115000** | Bipolar affective disorder, currently depressed, unspecified |
| **225E.00** | O/E - paranoid delusions |
| **Eu21.17** | [X]Pseudopsychopathic schizophrenia |
| **E105z00** | Latent schizophrenia NOS |
| **Eu20.00** | [X]Schizophrenia |
| **E116000** | Mixed bipolar affective disorder, unspecified |
| **Eu30y00** | [X]Other manic episodes |
| **Eu26.00** | [X]Nonorganic psychosis in remission |
| **E13z.00** | Nonorganic psychosis NOS |
| **E114200** | Bipolar affective disorder, currently manic, moderate |
| **212W.00** | Schizophrenia resolved |
| **Eu32900** | [X]Single major depr ep, severe with psych, psych in remiss |
| **Eu22011** | [X]Paranoid psychosis |
| **E102400** | Acute exacerbation of chronic catatonic schizophrenia |
| **Eu33300** | [X]Recurrent depress disorder cur epi severe with psyc symp |
| **E107400** | Acute exacerbation of chronic schizo-affective schizophrenia |
| **E100200** | Chronic schizophrenic |
| **Eu22.00** | [X]Persistent delusional disorders |
| **Eu20213** | [X]Schizophrenic catatonia |
| **Eu21.12** | [X]Borderline schizophrenia |
| **Eu02z15** | [X] Senile psychosis NOS |
| **Eu30000** | [X]Hypomania |
| **E105200** | Chronic latent schizophrenia |
| **E14z.11** | Childhood schizophrenia NOS |
| **285..11** | Psychotic condition, insight present |
| **E13..11** | Reactive psychoses |
| **Eu21.00** | [X]Schizotypal disorder |
| **Eu20500** | [X]Residual schizophrenia |
| **E11..13** | Manic psychoses |
| **E04..00** | Other chronic organic psychoses |
| **Eu32A00** | [X]Recurr major depr ep, severe with psych, psych in remiss |
| **Eu31000** | [X]Bipolar affective disorder, current episode hypomanic |
| **E107300** | Acute exacerbation subchronic schizo-affective schizophrenia |
| **E111500** | Recurrent manic episodes, partial or unspecified remission |
| **E114500** | Bipolar affect disord,currently manic, part/unspec remission |
| **13Y2.00** | Schizophrenia association member |
| **E111600** | Recurrent manic episodes, in full remission |
| **Eu31600** | [X]Bipolar affective disorder, current episode mixed |
| **E11y100** | Atypical manic disorder |
| **E103100** | Subchronic paranoid schizophrenia |
| **Eu31100** | [X]Bipolar affect disorder cur epi manic wout psychotic symp |
| **Eu31800** | [X]Bipolar affective disorder type I |
| **8BM0100** | Antipsychotic medication review |
| **Eu60100** | [X]Schizoid personality disorder |
| **Eu05212** | [X]Schizophrenia-like psychosis in epilepsy |
| **E14z.00** | Child psychosis NOS |
| **SL93.00** | Other antipsychotics/neuroleptics/tranquilliser poisoning |
| **E115.00** | Bipolar affective disorder, currently depressed |
| **E107200** | Chronic schizo-affective schizophrenia |
| **E110300** | Single manic episode, severe without mention of psychosis |
| **Eu31y12** | [X]Recurrent manic episodes |
| **E103.00** | Paranoid schizophrenia |

# Pulmonary circulation disorders (Elixhauser)

| **Read code** | **Description** |
| --- | --- |
| **L096400** | Pulmonary embolism following abortive pregnancy |
| **G4...11** | Heart disease - pulmonary |
| **7Q01200** | Primary pulmonary hypertension drugs Band 3 |
| **L431.00** | Amniotic fluid pulmonary embolism |
| **L43yz00** | Other obstetric pulmonary embolism NOS |
| **L431100** | Amniotic fluid pulmonary embolism - delivered |
| **G40z.00** | Acute pulmonary heart disease NOS |
| **G41z.11** | Chronic cor pulmonale |
| **7Q01000** | Primary pulmonary hypertension drugs Band 1 |
| **L43zz00** | Obstetric pulmonary embolism NOS |
| **SB16.00** | Pulmonary blood vessel injury |
| **L432.00** | Obstetric blood-clot pulmonary embolism |
| **L43z.00** | Obstetric pulmonary embolism NOS |
| **G401100** | Recurrent pulmonary embolism |
| **G41y000** | Secondary pulmonary hypertension |
| **G401.00** | Pulmonary embolism |
| **P737.11** | Dilatation of pulmonary artery |
| **G42..00** | Other pulmonary circulation disease |
| **G401000** | Post operative pulmonary embolus |
| **7A0B000** | Pulmonary thromboendarterectomy |
| **Gyu4.00** | [X]Pulmon heart disease & diseases of pulmonary circulation |
| **G41yz00** | Other chronic pulmonary heart disease NOS |
| **7A6Ky00** | Other specified operations on individual pulmonary veins |
| **L43z400** | Obstetric pulmonary embolism NOS with postnatal complication |
| **G410.00** | Primary pulmonary hypertension |
| **G41y100** | Thromboembolic pulmonary hypertension |
| **L43z000** | Obstetric pulmonary embolism NOS, unspecified |
| **G400.00** | Acute cor pulmonale |
| **G411.00** | Kyphoscoliotic heart disease |
| **L43..00** | Obstetric pulmonary embolism |
| **G42..11** | Pulmonary vessel disease |
| **G41z.00** | Chronic pulmonary heart disease NOS |
| **L430.00** | Obstetric air pulmonary embolism |
| **Gyu4100** | [X]Other diseases of pulmonary vessels |
| **G42yz00** | Other specified pulmonary circulation disease NOS |
| **G4y..00** | Other specified pulmonary circulation disease |
| **G41y.00** | Other chronic pulmonary heart disease |
| **7Q01100** | Primary pulmonary hypertension drugs Band 2 |
| **H54..00** | Pulmonary congestion and hypostasis |
| **G41..00** | Chronic pulmonary heart disease |
| **L43z100** | Obstetric pulmonary embolism NOS - delivered |
| **G42y.00** | Other specified pulmonary circulation disease |
| **G40..00** | Acute pulmonary heart disease |
| **7Q01300** | Primary pulmonary hypertension drugs Band 4 |
| **ZV12900** | [V] Personal history of pulmonary embolism |
| **H541.00** | Pulmonary congestion |
| **G4z..00** | Pulmonary circulation disease NOS |
| **G42z.00** | Other pulmonary circulation disease NOS |
| **G4...00** | Pulmonary circulation diseases |
| **P736.12** | Pulmonary arterio-venous malformation |

# Renal disease (Elixhauser)

| **Read code** | **Description** |
| --- | --- |
| **SP08F00** | Acute rejection of renal transplant - grade II |
| **A160000** | Tuberculous nephropathy |
| **K08y511** | AIN - Acute interstitial nephritis |
| **K100000** | Chronic pyelonephritis without medullary necrosis |
| **K08z.00** | Impaired renal function disorder NOS |
| **TB00111** | Renal transplant with complication, without blame |
| **8882.00** | Intestinal dialysis |
| **K0A5200** | Hereditry nephropathy NEC,difus membran glomerulnephritis |
| **583 GC** | GLOMERULONEPHRITIS CHRONIC |
| **K08y.00** | Other impaired renal function disorder |
| **9Ot4.00** | Chronic kidney disease monitoring telephone invite |
| **7L1B000** | Insertion of ambulatory peritoneal dialysis catheter |
| **1Z19.00** | Chronic kidney disease stage 2 with proteinuria |
| **K100300** | Chronic pyonephrosis |
| **K08..00** | Impaired renal function disorder |
| **SP15411** | Kidney failure as a complication of care |
| **1Z1F.00** | Chronic kidney disease stage 3B with proteinuria |
| **K034.00** | Renal cortical necrosis unspecified |
| **K0C1.00** | Nephropathy induced by other drugs meds and biologl substncs |
| **7L1A100** | Peritoneal dialysis |
| **K052.00** | Chronic kidney disease stage 2 |
| **K0A5600** | Hereditary nephropathy, NEC, dense deposit disease |
| **Kyu1E00** | [X]Tubulo-interstit nephritis, not specif as acute or chron |
| **ZV45100** | [V]Renal dialysis status |
| **K01y.00** | Nephrotic syndrome with other pathological kidney lesions |
| **K050.00** | End stage renal failure |
| **Z919300** | Reversing haemodialysis lines |
| **K100200** | Chronic pyelitis |
| **K0A3200** | Chron nephritic syndrom difuse membranous glomerulonephritis |
| **K04y.00** | Other acute renal failure |
| **TB11.11** | Renal dialysis with complication, without blame |
| **SP08G00** | Acute rejection of renal transplant - grade III |
| **SP08D00** | Acute-on-chronic rejection of renal transplant |
| **7L1A200** | Haemodialysis NEC |
| **Gy10.00** | Stenosis of dialysis arteriovenous graft |
| **K0A1600** | Rapid progressive nephritic syndrome, dense deposit disease |
| **K017.00** | Nephrotic syn difus mesangial prolifertiv glomerulonephritis |
| **ZV56.00** | [V]Aftercare involving intermittent dialysis |
| **SP07G00** | Stenosis of arteriovenous dialysis fistula |
| **K032600** | Berger's IgA or IgG nephropathy |
| **SP08R00** | Renal transplant rejection |
| **K041.00** | Acute renal cortical necrosis |
| **K08y513** | Acute tubulo-interstitial nephritis |
| **7257300** | Correction iridodialysis NEC |
| **K05..12** | End stage renal failure |
| **K13yz11** | Salt-losing nephritis |
| **G72C.00** | Ruptured aneurysm of dialysis vascular access |
| **TA02000** | Accid cut,puncture,perf,h'ge - kidney dialysis |
| **K032y00** | Nephritis unsp+OS membranoprolif glomerulonephritis lesion |
| **1Z16.00** | Chronic kidney disease stage 3B |
| **K0A0200** | Acute nephritic syn, diffuse membranous glomerulonephritis |
| **7B06300** | Exploration of renal transplant |
| **K0A3000** | Chronic nephritic syndrome, minor glomerular abnormality |
| **F447C00** | Iridodialysis |
| **5930AR** | OSTEODYSTROPHY URAEMIC |
| **1Z1L.11** | CKD stage 5 without proteinuria |
| **K13yB00** | Ischaemic nephropathy |
| **SP08E00** | Acute rejection of renal transplant - grade I |
| **1Z1K.11** | CKD stage 5 with proteinuria |
| **ZV56y11** | [V]Aftercare involving peritoneal dialysis |
| **K06..11** | Uraemia NOS |
| **K080200** | Renal infantilism |
| **K0A4X00** | Isolated proteinuria, with oth specif morpholog changes |
| **K03U.00** | Unspecif nephr synd, diff concentric glomerulonephritis |
| **K03z.00** | Unspecified glomerulonephritis NOS |
| **K018.00** | Nephrotic syn,difus endocapilary proliftv glomerulonephritis |
| **7L1A011** | Thomas intravascular shunt for dialysis |
| **SP08W00** | Vascular complication of renal transplant |
| **K019.00** | Nephrotic syn,diffuse mesangiocapillary glomerulonephritis |
| **1Z13.00** | Chronic kidney disease stage 4 |
| **Gy60.00** | Rupture of dialysis arteriovenous graft |
| **7A61A00** | Ligation of arteriovenous dialysis graft |
| **Gy31.00** | Occlusion of dialysis arteriovenous fistula |
| **7L1B.00** | Placement ambulatory apparatus compensation renal failure |
| **Gy5..00** | Haemorrhage of dialysis vascular access |
| **SP01500** | Mechanical complication of dialysis catheter |
| **K03T.00** | Tubulo-interstit nephritis, not specif as acute or chron |
| **K016.00** | Nephrotic syndrome, diffuse membranous glomerulonephritis |
| **K06..12** | Kidney failure unspecified |
| **K00..00** | Acute glomerulonephritis |
| **C353600** | Renal failure-associated hyperphosphataemia |
| **K031.00** | Membranous nephritis unspecified |
| **7L1B.11** | Placement ambulatory dialysis apparatus - compens renal fail |
| **K060.11** | Impaired renal function |
| **K100.00** | Chronic pyelonephritis |
| **K100100** | Chronic pyelonephritis with medullary necrosis |
| **K032y13** | Mesangioproliferative glomerulonephritis NEC |
| **7L1Cz00** | Placement other apparatus- compensate for renal failure NOS |
| **ZV56100** | [V]Preparatory care for dialysis |
| **7L1By00** | Placement ambulatory apparatus- compensate renal failure OS |
| **1Z17.00** | Chronic kidney disease stage 1 with proteinuria |
| **K032y11** | Hypocomplementaemic persistent glomerulonephritis NEC |
| **9Ot0.00** | Chronic kidney disease monitoring first letter |
| **7L1A500** | Continuous ambulatory peritoneal dialysis |
| **SP0F.00** | Haemodialysis first use syndrome |
| **1Z1D.00** | Chronic kidney disease stage 3A with proteinuria |
| **4N2..00** | Dialysis fluid glucose level |
| **K0A3500** | Chronic neph syn difus mesangiocapillary glomerulonephritis |
| **ZV42000** | [V]Kidney transplanted |
| **9Ot2.00** | Chronic kidney disease monitoring third letter |
| **SP08H00** | Acute rejection of renal transplant |
| **7L1Ay00** | Other specified compensation for renal failure |
| **Gy21.00** | Thrombosis of dialysis arteriovenous fistula |
| **14S2.00** | H/O: kidney recipient |
| **K0A0400** | Ac neph syn difus endocaplry prolifrative glomerulonephritis |
| **SP0E.00** | Disorders associated with peritoneal dialysis |
| **14V2.00** | H/O: renal dialysis |
| **TB00100** | Kidney transplant with complication, without blame |
| **K02y200** | Chronic focal glomerulonephritis |
| **K06..00** | Renal failure unspecified |
| **K02..12** | Nephropathy - chronic |
| **Z1A1.00** | Peritoneal dialysis training |
| **1Z19.11** | CKD stage 2 with proteinuria |
| **K00yz00** | Other acute glomerulonephritis NOS |
| **SP08T00** | Urological complication of renal transplant |
| **K0A8.00** | Rapidly progressive glomerulonephritis |
| **PD23.00** | Congenital hydronephrosis |
| **9Ot1.00** | Chronic kidney disease monitoring second letter |
| **7L1C.00** | Placement other apparatus for compensation for renal failure |
| **1Z1H.00** | Chronic kidney disease stage 4 with proteinuria |
| **1Z18.00** | Chronic kidney disease stage 1 without proteinuria |
| **K054.00** | Chronic kidney disease stage 4 |
| **K03..12** | Nephropathy, unspecified |
| **5932MN** | RENAL MEDULLARY NECROSIS |
| **K055.00** | Chronic kidney disease stage 5 |
| **K035.00** | Renal medullary necrosis unspecified |
| **K08y500** | Acute interstitial nephritis |
| **K02..00** | Chronic glomerulonephritis |
| **7B00500** | Allotransplantation of kidney from cadaver NEC |
| **Kyu0900** | [X]Unsp nephrit synd, diff mesang prolif glomerulonephritis |
| **K03W.00** | Unsp nephrit synd, diff endocap prolif glomerulonephritis |
| **K0A0100** | Acute nephritic syndrome, focal+segmental glomerular lesions |
| **G72D200** | Aneurysm of anastomotic site of dialysis AV fistula |
| **1Z1H.11** | CKD stage 4 with proteinuria |
| **K000.00** | Acute proliferative glomerulonephritis |
| **SP08V00** | Very mild acute rejection of renal transplant |
| **1Z1C.11** | CKD stage 3 without proteinuria |
| **K0A5.00** | Hereditary nephropathy not elsewhere classified |
| **K014.00** | Nephrotic syndrome, minor glomerular abnormality |
| **K100600** | Calculous pyelonephritis |
| **K0y..00** | Other specified nephritis, nephrosis or nephrotic syndrome |
| **7L1A.11** | Dialysis for renal failure |
| **K03y000** | Other nephritis and nephrosis in diseases EC |
| **K0A4W00** | Isolated proteinuria, with unspecified morpholog changes |
| **K00y200** | Acute focal nephritis |
| **1Z1J.11** | CKD stage 4 without proteinuria |
| **K10y.00** | Pyelonephritis and pyonephrosis unspecified |
| **K0A0700** | Acute nephrotic syndrm diffuse crescentic glomerulonephritis |
| **K011.00** | Nephrotic syndrome with membranous glomerulonephritis |
| **K100500** | Chronic obstructive pyelonephritis |
| **K0A0000** | Acute nephritic syndrome, minor glomerular abnormality |
| **K101000** | Acute pyelonephritis without medullary necrosis |
| **G72D100** | Aneurysm of needle site of dialysis arteriovenous fistula |
| **K0E..00** | Acute-on-chronic renal failure |
| **K0A2200** | Recur+persist haematuria difus membranous glomerulonephritis |
| **4I29.00** | Peritoneal dialysis sample |
| **67P4100** | Discussion about kidney transplantation |
| **K03y.00** | Other nephritis and nephrosis unspecified |
| **1Z11.00** | Chronic kidney disease stage 2 |
| **7L1A400** | Automated peritoneal dialysis |
| **K0A5500** | [X]Heredtry nephrpthy NEC difus mesangiocapilry glomneph |
| **K03..00** | Nephritis and nephropathy unspecified |
| **K032200** | Focal glomerulon + focal recurr macroscop glomerulonephritis |
| **K00y000** | Acute glomerulonephritis in diseases EC |
| **K020.00** | Chronic proliferative glomerulonephritis |
| **K101100** | Acute pyelonephritis with medullary necrosis |
| **K0A2800** | IgA nephropathy |
| **K0A3700** | Chronic nephritic syn diffuse crescentic glomerulonephritis |
| **K05..00** | Chronic renal failure |
| **K012.00** | Nephrotic syndrome+membranoproliferative glomerulonephritis |
| **K033.00** | Rapidly progressive nephritis unspecified |
| **K081.00** | Nephrogenic diabetes insipidus |
| **K0G..00** | Sickle cell nephropathy |
| **Gy40.00** | Infection of dialysis arteriovenous graft |
| **9Ot..00** | Chronic kidney disease monitoring administration |
| **K02..11** | Nephritis - chronic |
| **5930A** | OSTEODYSTROPHY AZOTAEMIC |
| **K080.00** | Renal osteodystrophy |
| **1Z1G.11** | CKD stage 3B without proteinuria |
| **K0...00** | Nephritis, nephrosis and nephrotic syndrome |
| **D215.00** | Anaemia secondary to renal failure |
| **K0C0.00** | Analgesic nephropathy |
| **7A61900** | Ligation of arteriovenous dialysis fistula |
| **D215000** | Anaemia secondary to chronic renal failure |
| **K08y700** | Acute infective interstitial nephritis |
| **SP0G.00** | Anaphylactoid reaction due to haemodialysis |
| **Z919.00** | Care of haemodialysis equipment |
| **K0z..00** | Nephritis, nephrosis and nephrotic syndrome NOS |
| **K100z00** | Chronic pyelonephritis NOS |
| **1Z1B.11** | CKD stage 3 with proteinuria |
| **K0A3300** | Chron neph syn difus mesangial prolifrtiv glomerulonephritis |
| **Kyu0F00** | [X]Hereditary nephropathy, unspecif morphological changes |
| **K032400** | Familial glomerulonephritis in Alport's syndrome |
| **TA22000** | Failure of sterile precautions during kidney dialysis |
| **K0A5000** | Hereditary nephropathy NEC, minor glomerular abnormality |
| **1Z1E.00** | Chronic kidney disease stage 3A without proteinuria |
| **K04z.00** | Acute renal failure NOS |
| **K030.00** | Proliferative nephritis unspecified |
| **K08yz00** | Other impaired renal function disorder NOS |
| **SP05613** | [X] Peritoneal dialysis associated peritonitis |
| **K104.00** | Xanthogranulomatous pyelonephritis |
| **9Ot3.00** | Chronic kidney disease monitoring verbal invite |
| **K0A0600** | Acute nephritic syndrome, dense deposit disease |
| **1Z12.00** | Chronic kidney disease stage 3 |
| **G72D.00** | Aneurysm of dialysis arteriovenous fistula |
| **SP06B00** | Continuous ambulatory peritoneal dialysis associated perit |
| **Gy3..00** | Occlusion of dialysis vascular access |
| **ZV56000** | [V]Aftercare involving extracorporeal dialysis |
| **K0A5X00** | Hereditary nephropathy, unspecif morphological changes |
| **K01..00** | Nephrotic syndrome |
| **1Z1F.11** | CKD stage 3B with proteinuria |
| **K01w112** | Wilms' tumour + nephrotic syndrome + pseudohermaphroditism |
| **K0A2700** | Recur+persist haematuria difus crescentic glomerulonephritis |
| **K0A3100** | Chronic nephritic syndrm focal+segmental glomerular lesions |
| **K000111** | CGN - Crescentic glomerulonephritis |
| **Kyu2100** | [X]Other chronic renal failure |
| **K03V.00** | Unspecified nephritic syndrome, dense deposit disease |
| **K08y000** | Hypokalaemic nephropathy |
| **7L1A000** | Renal dialysis |
| **K013.11** | Lipoid nephrosis |
| **K190X00** | Persistent proteinuria, unspecified |
| **7B00200** | Transplantation of kidney from cadaver |
| **K00..11** | Acute nephritis |
| **G22z.11** | Renal hypertension |
| **A160200** | Tuberculous pyelonephritis |
| **K0A3600** | Chronic nephritic syndrome, dense deposit disease |
| **K0B6.00** | Balkan nephropathy |
| **K02y300** | Chronic diffuse glomerulonephritis |
| **Z1A..00** | Dialysis training |
| **1Z15.00** | Chronic kidney disease stage 3A |
| **K0A1300** | Rpd prog neph syn df mesangial prolifratv glomerulonephritis |
| **582 N** | CHRONIC NEPHRITIS |
| **7A60600** | Creation of graft fistula for dialysis |
| **K021.00** | Chronic membranous glomerulonephritis |
| **1Z1..00** | Chronic renal impairment |
| **K00y100** | Acute exudative nephritis |
| **K02yz00** | Other chronic glomerulonephritis NOS |
| **ZV56011** | [V]Aftercare involving renal dialysis NOS |
| **6AA..00** | Chronic kidney disease annual review |
| **Kyu1400** | [X]Nephropathy induced by other drugs+biological substances |
| **C372411** | Uric acid nephropathy |
| **7L1A600** | Peritoneal dialysis NEC |
| **7L1Cy00** | Placement other apparatus- compensate for renal failure OS |
| **14V2.11** | H/O: kidney dialysis |
| **K032300** | Anaphylactoid glomerulonephritis |
| **K032y14** | Mesangiocapillary glomerulonephritis NEC |
| **F410400** | New partial retinal detachment with retinal dialysis |
| **ZV56z00** | [V]Unspecified aftercare involving intermittent dialysis |
| **1Z1D.11** | CKD stage 3A with proteinuria |
| **Gy51.00** | Haemorrhage of dialysis arteriovenous fistula |
| **Kyu1000** | [X]Other chronic tubulo-interstitial nephritis |
| **K0A1.00** | Rapidly progressive nephritic syndrome |
| **K032.00** | Membranoproliferative nephritis unspecified |
| **A844100** | Plasmodium malariae malaria with nephropathy |
| **7B00212** | Cadaveric renal transplant |
| **K10y300** | Pyelonephritis in diseases EC |
| **4N0..00** | Dialysis fluid urea level |
| **K01z.00** | Nephrotic syndrome NOS |
| **K03y200** | Other interstitial nephritis |
| **1Z1G.00** | Chronic kidney disease stage 3B without proteinuria |
| **K032500** | Other familial glomerulonephritis |
| **K010.00** | Nephrotic syndrome with proliferative glomerulonephritis |
| **Kyu2.00** | [X]Renal failure |
| **7L1B200** | Flushing of peritoneal dialysis catheter |
| **1Z1A.00** | Chronic kidney disease stage 2 without proteinuria |
| **7B00400** | Allotransplantation kidney from cadaver, heart non-beating |
| **K042.00** | Acute renal medullary necrosis |
| **K03..11** | Nephritis and nephropathy unspecified |
| **K053.00** | Chronic kidney disease stage 3 |
| **K01x111** | Kimmelstiel - Wilson disease |
| **K02y000** | Chronic glomerulonephritis + diseases EC |
| **K0A0500** | Acute neph syn, diffuse mesangiocapillary glomerulonephritis |
| **K100400** | Nonobstructive reflux-associated chronic pyelonephritis |
| **K0A3.00** | Chronic nephritic syndrome |
| **K0A5100** | Hereditary nephropathy NEC,focal+segmnt glomerular lesion |
| **K0D..00** | End-stage renal disease |
| **7598A** | NEPHRITIS HEREDITARY |
| **K03X.00** | Unsp nephrit synd, diff mesang prolif glomerulonephritis |
| **K032000** | Focal membranoproliferative glomerulonephritis |
| **K00y.00** | Other acute glomerulonephritis |
| **Gy1..00** | Stenosis of dialysis vascular access |
| **Kyu5G00** | [X]Persistent proteinuria, unspecified |
| **K0C2.00** | Nephropathy induced by unspec drug medicament or biol subs |
| **ZVu3G00** | [X]Other dialysis |
| **K01x200** | Nephrotic syndrome in malaria |
| **C341z00** | Gouty nephropathy NOS |
| **K0A1200** | Rapid progres neph syn diffuse membranous glomerulonephritis |
| **G22..00** | Hypertensive renal disease |
| **ZV56y00** | [V]Other specified aftercare involving intermittent dialysis |
| **1Z14.00** | Chronic kidney disease stage 5 |
| **K04E.00** | Acute kidney injury stage 3 |
| **7L1Az00** | Compensation for renal failure NOS |
| **K023.00** | Chronic rapidly progressive glomerulonephritis |
| **661M200** | Chronic kidney disease self-management plan agreed |
| **K00z.00** | Acute glomerulonephritis NOS |
| **1Z1J.00** | Chronic kidney disease stage 4 without proteinuria |
| **Cyu2300** | [X]Unspecified diabetes mellitus with renal complications |
| **K01x300** | Nephrotic syndrome in polyarteritis nodosa |
| **K02z.00** | Chronic glomerulonephritis NOS |
| **SP08N00** | Unexplained episode of renal transplant dysfunction |
| **7B00211** | Allotransplantation of kidney from cadaver |
| **K022.00** | Chronic membranoproliferative glomerulonephritis |
| **5932KH** | NEPHROPATHY HYPOKALAEMIC |
| **K0A3400** | Chron neph syn difuse endocap prolifrativ glomerulonephritis |
| **K032y15** | Mixed membranous and proliferative glomerulonephritis NEC |
| **F374A00** | Polyneuropathy in uraemia |
| **1Z1E.11** | CKD stage 3A without proteinuria |
| **K001.00** | Acute nephritis with lesions of necrotising glomerulitis |
| **K01B.00** | Nephrotic syndrome, diffuse crescentic glomerulonephritis |
| **K01x.00** | Nephrotic syndrome in diseases EC |
| **K0A1100** | Rapid progres nephritic syn focal+segmental glomerulr lesion |
| **Z1A2.00** | Haemodialysis training |
| **Gy41.00** | Infection of dialysis arteriovenous fistula |
| **5932EC** | CHRONIC RENAL FAILURE |
| **9Ni9.00** | Did not attend chronic kidney disease monitoring clinic |
| **K01w000** | Finnish nephrosis syndrome |
| **7L1A.00** | Compensation for renal failure |
| **66i..00** | Chronic kidney disease monitoring |
| **K080300** | Renal rickets |
| **583 A** | NEPHRITIS |
| **C341.00** | Gouty nephropathy |
| **K01x000** | Nephrotic syndrome in amyloidosis |
| **K01A.00** | Nephrotic syndrome, dense deposit disease |
| **5932E** | RENAL FAILURE |
| **583 MA** | MESANGIOCAPILLARY GLOMERULONEPHRITIS |
| **K0A5300** | Heredtry nephrpthy NEC difus mesangial prolif glomnephrit |
| **D310100** | Henoch-Schonlein nephritis |
| **K013.12** | Steroid sensitive nephrotic syndrome |
| **K00y300** | Acute diffuse nephritis |
| **Q48y000** | Congenital renal failure |
| **SP08300** | Kidney transplant failure and rejection |
| **K05..13** | Chronic kidney disease |
| **K02y.00** | Other chronic glomerulonephritis |
| **583 MN** | NEPHROPATHY MEMBRANOUS |
| **1Z1L.00** | Chronic kidney disease stage 5 without proteinuria |
| **583 MP** | MEMBRANOPROLIFERATIVE GLOMERULONEPHRITIS |
| **Z919100** | Priming haemodialysis lines |
| **K0A0.00** | Acute nephritic syndrome |
| **5930R** | OSTEODYSTROPHY RENAL |
| **7B00600** | Xenograft renal transplant |
| **1Z10.00** | Chronic kidney disease stage 1 |
| **1Z1C.00** | Chronic kidney disease stage 3 without proteinuria |
| **14D1.00** | H/O: nephritis |
| **K05..11** | Chronic uraemia |
| **K105.00** | Chronic infective interstitial nephritis |
| **K080100** | Renal dwarfism |
| **K0A2300** | Recur+persist haemuria df mesangial prolif glomerulnephritis |
| **C372400** | Urate nephropathy |
| **Kyu2000** | [X]Other acute renal failure |
| **A786.00** | Haemorrhagic nephrosonephritis |
| **K000100** | Crescentic glomerulonephritis |
| **K032z00** | Nephritis unsp+membranoprolif glomerulonephritis lesion NOS |
| **K0A4.00** | Isolated proteinuria with specified morphological lesion |
| **TB11.00** | Kidney dialysis with complication, without blame |
| **Kyu1100** | [X]Other and unspecified hydronephrosis |
| **7261011** | Cyclodialysis |
| **SP08J00** | Chronic rejection of renal transplant |
| **8L50.00** | Renal transplant planned |
| **K0A1700** | Rapid progres nephritic syn df crescentic glomerulonephritis |
| **K0A2500** | Recur+persist hmuria df mesangiocapilary glomerulonephritis |
| **K0A0300** | Acut neph syn, diffuse mesangial prolifrative glomnephritis |
| **7L1B100** | Removal of ambulatory peritoneal dialysis catheter |
| **1Z1K.00** | Chronic kidney disease stage 5 with proteinuria |
| **5932A** | RENAL DISEASE |
| **Z91A.00** | Peritoneal dialysis bag procedure |
| **7B0Fz00** | Interventions associated with transplantation of kidney NOS |
| **1Z1B.00** | Chronic kidney disease stage 3 with proteinuria |
| **K013.00** | Nephrotic syndrome with minimal change glomerulonephritis |
| **K03yz00** | Other nephritis and nephrosis NOS |
| **K051.00** | Chronic kidney disease stage 1 |
| **1Z1A.11** | CKD stage 2 without proteinuria |
| **K01w.00** | Congenital nephrotic syndrome |
| **K015.00** | Nephrotic syndrome, focal and segmental glomerular lesions |
| **K01x411** | Lupus nephritis |
| **7L1f000** | Extracorporeal albumin haemodialysis |
| **G233.00** | Hypertensive heart and renal disease with renal failure |
| **K080000** | Phosphate-losing tubular disorders |
| **Gy30.00** | Occlusion of dialysis arteriovenous graft |
| **K0C4.00** | Toxic nephropathy, not elsewhere classified |
| **7L1C000** | Insertion of temporary peritoneal dialysis catheter |
| **1Z17.11** | CKD stage 1 with proteinuria |
| **K080z00** | Renal osteodystrophy NOS |

# Rhematoid arthritis and collagen diseases (Elixhauser)

| **Read code** | **Description** |
| --- | --- |
| **N001.12** | Systemic sclerosis |
| **9hR..00** | Exception reporting: rheumatoid arthritis quality indicators |
| **N04..00** | Rheumatoid arthritis and other inflammatory polyarthropathy |
| **N040.00** | Rheumatoid arthritis |
| **9mM0.00** | Rheumatoid arthritis monitoring invitation first letter |
| **N10y000** | Inflammatory spondylopathies in diseases EC |
| **N040M00** | Rheumatoid arthritis of IP joint of toe |
| **N10y.00** | Other inflammatory spondylopathies |
| **7340BC** | SCLERODERMA ACROSCLEROTIC |
| **N04..11** | Inflammatory polyarthropathy |
| **N045000** | Juvenile ankylosing spondylitis |
| **N00y.11** | Fibrosclerosis - systemic |
| **9mM3.00** | Rheumatoid arthritis monitoring verbal invitation |
| **N040L00** | Rheumatoid arthritis of lesser MTP joint |
| **N045600** | Pauciarticular onset juvenile chronic arthritis |
| **F396100** | Myopathy due to disseminated lupus erythematosus |
| **66H..13** | Rheumatoid arthrit. monitoring |
| **N040F00** | Rheumatoid arthritis of ankle |
| **N00y.00** | Other specified diffuse collagen diseases |
| **F37X.00** | Inflammatory polyneuropathy, unspecified |
| **N042000** | Rheumatic carditis |
| **N002.00** | Sicca (Sjogren's) syndrome |
| **715 MR** | MONOARTICULAR RHEUMATISM |
| **F396400** | Myopathy due to rheumatoid arthritis |
| **N003X00** | Dermatopolymyositis, unspecified |
| **F396600** | Myopathy due to scleroderma |
| **7149A** | RHEUMATIC ARTHRITIS |
| **718 BH** | RHEUMATISM HANDS ACUTE |
| **H121500** | Pharyngitis sicca |
| **7179FN** | RHEUMATISM NONARTICULAR |
| **N045100** | Juvenile seronegative polyarthritis |
| **L 151F** | SERO POSITIVE RHEUMATOID ARTHRITIS |
| **7161.00** | POLYMYOSITIS |
| **N102.00** | Sacroiliitis NEC |
| **N040D00** | Rheumatoid arthritis of knee |
| **N040P00** | Seronegative rheumatoid arthritis |
| **N04y200** | Adult-onset Still's disease |
| **N04y000** | Rheumatoid lung |
| **N04yz00** | Other specified inflammatory polyarthropathy NOS |
| **N04y011** | Caplan's syndrome |
| **N043200** | Pauciarticular juvenile rheumatoid arthritis |
| **N040K00** | Rheumatoid arthritis of 1st MTP joint |
| **Nyu6.00** | [X]Spondylopathies |
| **H570.00** | Rheumatoid lung |
| **N003.00** | Dermatomyositis |
| **N002.11** | Keratoconjunctivitis sicca |
| **9hR0.00** | Except rheumatoid arthritis quality indicator: pt unsuitable |
| **N040000** | Rheumatoid arthritis of cervical spine |
| **Nyu6100** | [X]Other specified inflammatory spondylopathies |
| **N042.00** | Other rheumatoid arthropathy + visceral/systemic involvement |
| **7P20300** | Delivery of rehabilitation for rheumatoid arthritis |
| **N045500** | Juvenile rheumatoid arthritis |
| **N004.00** | Polymyositis |
| **N040500** | Rheumatoid arthritis of elbow |
| **N040100** | Other rheumatoid arthritis of spine |
| **N040900** | Rheumatoid arthritis of PIP joint of finger |
| **C37z.11** | Marinesco-Sjogren syndrome |
| **N001000** | Progressive systemic sclerosis |
| **N240700** | Hand rheumatism |
| **N040E00** | Rheumatoid arthritis of tibio-fibular joint |
| **N040B00** | Rheumatoid arthritis of hip |
| **N005.00** | Adult Still's Disease |
| **14G1.00** | H/O: rheumatoid arthritis |
| **N04y300** | Remitting seronegative symmetrical synovitis pitting oedema |
| **F371000** | Polyneuropathy in disseminated lupus erythematosus |
| **L 151E** | SERO NEGATIVE RHEUMATOID ARTHRITIS |
| **N047.00** | Seropositive errosive rheumatoid arthritis |
| **N043000** | Juvenile rheumatoid arthropathy unspecified |
| **H160400** | Laryngitis sicca |
| **F4A3200** | Keratoconjunctivitis sicca (excluding Sjogren's syndrome) |
| **N043100** | Acute polyarticular juvenile rheumatoid arthritis |
| **7340C** | SCLERODERMA DIFFUSE |
| **N060.11** | Endemic polyarthritis |
| **N000400** | Systemic lupus erythematosus with pericarditis |
| **K01x400** | Nephrotic syndrome in systemic lupus erythematosus |
| **N040J00** | Rheumatoid arthritis of other tarsal joint |
| **ZRq9.00** | Systemic lupus erythematosus disease activity index |
| **ZRq8.00** | Systemic lupus activity measure |
| **N043z00** | Juvenile rheumatoid arthritis NOS |
| **7179GA** | RHEUMATISM MUSCULAR ARM |
| **N10..00** | Inflammatory spondylopathies |
| **N101.00** | Spinal enthesopathy |
| **N240.00** | Rheumatism and fibrositis unspecified |
| **N2z..00** | Nonarticular rheumatism NOS |
| **N001.11** | Acrosclerosis |
| **7179GB** | RHEUMATISM NONARTICULAR SHOULDER |
| **H57y400** | Lung disease with systemic lupus erythematosus |
| **38DZ000** | Disease activity score 28 joint in rheumatoid arthritis |
| **718 AH** | RHEUMATISM HANDS |
| **7340BA** | ACROSCLEROSIS |
| **N04y111** | Sero negative polyarthritis |
| **G5y8.00** | Rheumatoid myocarditis |
| **N240000** | Rheumatism unspecified |
| **F371200** | Polyneuropathy in rheumatoid arthritis |
| **N041.00** | Felty's syndrome |
| **PH12.11** | Sjogren - Larsson syndrome |
| **N000300** | Systemic lupus erythematosus with organ or sys involv |
| **N043300** | Monarticular juvenile rheumatoid arthritis |
| **7123.00** | RHEUMATOID ARTHRITIS |
| **N045.00** | Other juvenile arthritis |
| **7123CR** | RHEUMATOID ARTHRITIS INCREASED ACTIVITY |
| **M210.11** | Addison's keloid |
| **N040300** | Rheumatoid arthritis of sternoclavicular joint |
| **M210z00** | Circumscribed scleroderma NOS |
| **K0H..00** | Acute scleroderma renal crisis |
| **N000z00** | Systemic lupus erythematosus NOS |
| **7340A** | SCLERODERMA |
| **N231400** | Polymyositis ossificans |
| **Nyu1200** | [X]Other specified rheumatoid arthritis |
| **7341AA** | LUPUS ERYTHEMATOSUS SYSTEMIC |
| **7179G** | RHEUMATISM MUSCULAR |
| **9mM1.00** | Rheumatoid arthritis monitoring invitation second letter |
| **N040800** | Rheumatoid arthritis of MCP joint |
| **N001200** | Systemic sclerosis induced by drugs and chemicals |
| **H57y300** | Lung disease with Sjogren's disease |
| **N040R00** | Rheumatoid nodule |
| **7121.00** | SYNDROME FELTY'S |
| **N04y.00** | Other specified inflammatory polyarthropathy |
| **N040600** | Rheumatoid arthritis of distal radio-ulnar joint |
| **M210.00** | Circumscribed scleroderma |
| **Nyu6500** | [X]Other specified spondylopathies |
| **N040H00** | Rheumatoid arthritis of talonavicular joint |
| **9mM..00** | Rheumatoid arthritis monitoring invitation |
| **Nyu1000** | [X]Rheumatoid arthritis+involvement/other organs or systems |
| **9mM4.00** | Rheumatoid arthritis monitoring telephone invitation |
| **N040Q00** | Rheumatoid bursitis |
| **ZRq8.11** | SLAM - Systemic lupus activity measure |
| **N044.11** | Jaccoud's syndrome |
| **N044.00** | Chronic post-rheumatic arthropathy |
| **6954.00** | SYSTEMIC LUPUS ERYTHEMATOSUS |
| **N200.00** | Giant cell arteritis with polymyalgia rheumatica |
| **7341CL** | LIBMAN- SACKS DISEASE |
| **Nyu4500** | [X]Other forms of systemic sclerosis |
| **N042100** | Rheumatoid lung disease |
| **K0J0.00** | Renal involvement in scleroderma |
| **N040700** | Rheumatoid arthritis of wrist |
| **7340D** | SCLERODERMA GENERALIZED |
| **66HB000** | Rheumatoid arthritis annual review |
| **N044.12** | Nodular fibrositis of chronic rheumatic disease |
| **M210400** | Linear scleroderma |
| **F396700** | Myopathy due to Sjogren's disease |
| **7179PR** | POLYMYALGIA RHEUMATICA |
| **718** | RHEUMATISM |
| **N040T00** | Flare of rheumatoid arthritis |
| **N000.00** | Systemic lupus erythematosus |
| **2A42.00** | SYSTEMIC LUPUS ERYTHEMATOSUS WITH RENAL |
| **N000100** | Libman-Sacks disease |
| **N001.00** | Scleroderma |
| **388p.00** | BASDAI - Bath ankylosing spondylitis disease activity index |
| **Nyu4400** | [X]Other dermatomyositis |
| **N040N00** | Rheumatoid vasculitis |
| **M210000** | Unspecified circumscribed scleroderma |
| **N040A00** | Rheumatoid arthritis of DIP joint of finger |
| **N003.11** | Poikilodermatomyositis |
| **N240200** | Muscular rheumatism |
| **N040C00** | Rheumatoid arthritis of sacro-iliac joint |
| **Nyu1100** | [X]Other seropositive rheumatoid arthritis |
| **9mM2.00** | Rheumatoid arthritis monitoring invitation third letter |
| **N00..11** | Collagen diseases |
| **G5yA.00** | Rheumatoid carditis |
| **N000200** | Drug-induced systemic lupus erythematosus |
| **N042200** | Rheumatoid nodule |
| **Nyu1.00** | [X]Inflammatory polyarthropathies |
| **N003100** | Dermatopolymyositis in neoplastic disease |
| **Nyu1G00** | [X]Seropositive rheumatoid arthritis, unspecified |
| **Nyu4300** | [X]Other forms of systemic lupus erythematosus |
| **7341.00** | LUPUS ERYTHEMATOSUS DISSEMINATED |
| **G759.00** | Juvenile polyarteritis |
| **N240z00** | Rheumatism or fibrositis NOS |
| **N10yz00** | Other inflammatory spondylopathies NOS |
| **38DZ.00** | Disease activity score in rheumatoid arthritis |
| **N003000** | Juvenile dermatomyositis |
| **N00z.00** | Collagen disease NOS |
| **N042z00** | Rheumatoid arthropathy + visceral/systemic involvement NOS |
| **N040400** | Rheumatoid arthritis of acromioclavicular joint |
| **N100.00** | Ankylosing spondylitis |
| **7341AD** | LUPUS ERYTHEMATOSUS ACUTE |
| **N040200** | Rheumatoid arthritis of shoulder |
| **N04X.00** | Seropositive rheumatoid arthritis, unspecified |
| **N001100** | CREST syndrome |
| **7341AC** | ACUTE SYSTEMIC LUPUS ERYTHEMATOSUS |
| **N043.00** | Juvenile rheumatoid arthritis - Still's disease |
| **9hR1.00** | Except rheumatoid arthritis qual indicator: informed dissent |
| **Nyu4E00** | [X]Dermatopolymyositis, unspecified |
| **N04z.00** | Inflammatory polyarthropathy NOS |
| **N040S00** | Rheumatoid arthritis - multiple joint |
| **7124A** | RHEUMATOID ARTHRITIS SPINE |
| **N2y..00** | Other specified nonarticular rheumatism |
| **N000000** | Disseminated lupus erythematosus |
| **N20..00** | Polymyalgia rheumatica |
| **N040G00** | Rheumatoid arthritis of subtalar joint |

# Solid tumour or leukaemia (Elixhauser)

| **Read code** | **Description** |
| --- | --- |
| **B17y.00** | Malignant neoplasm of other specified sites of pancreas |
| **B50..00** | Malignant neoplasm of eye |
| **BBEC.00** | [M]Malignant melanoma in junctional naevus |
| **BB5D.11** | [M]Biliary tract adenomas and adenocarcinomas |
| **BBLG.00** | [M]Carcinoma in pleomorphic adenoma |
| **B48yz00** | Malignant neoplasm of other male genital organ NOS |
| **B34yz00** | Malignant neoplasm of other site of female breast NOS |
| **B302z00** | Malignant neoplasm of vertebral column NOS |
| **A220.11** | Malignant pustule |
| **B81y000** | Carcinoma in situ of pleura |
| **B223100** | Malignant neoplasm of middle lobe of lung |
| **B150z00** | Primary malignant neoplasm of liver NOS |
| **B200100** | Malignant neoplasm of nasal conchae |
| **B114.00** | Malignant neoplasm of body of stomach |
| **B326500** | Malignant melanoma of thumb |
| **B4A1.00** | Malignant neoplasm of renal pelvis |
| **B800400** | Carcinoma in situ of floor of mouth |
| **B333500** | Malignant neoplasm of skin of temple |
| **A789800** | HIV disease resulting in multiple malignant neoplasms |
| **B825200** | Carcinoma in situ of skin of axilla |
| **B4...11** | Carcinoma of genitourinary organ |
| **B205.00** | Malignant neoplasm of sphenoidal sinus |
| **B810000** | Carcinoma in situ of thyroid cartilage |
| **B055100** | Malignant neoplasm of roof of mouth |
| **B801z00** | Carcinoma in situ of oesophagus NOS |
| **B130.00** | Malignant neoplasm of hepatic flexure of colon |
| **B542100** | Malignant neoplasm of craniopharyngeal duct |
| **B337200** | Malignant neoplasm of skin of knee |
| **BBLH.00** | [M]Rhabdoid sarcoma |
| **B115.00** | Malignant neoplasm of lesser curve of stomach unspecified |
| **B822z00** | Carcinoma in situ skin of ear/external auricular canal NOS |
| **B011.00** | Malignant neoplasm of dorsal surface of tongue |
| **B213200** | Malignant neoplasm of cuneiform cartilage |
| **BB0A.00** | [M]Malignant tumour, fusiform cell type |
| **B801200** | Carcinoma in situ of lower 1/3 oesophagus |
| **B517200** | Malignant neoplasm of midbrain |
| **B471.00** | Malignant neoplasm of descended testis |
| **B830100** | Intraductal carcinoma in situ of breast |
| **B36..00** | Local recurrence of malignant tumour of breast |
| **B4A1z00** | Malignant neoplasm of renal pelvis NOS |
| **B337400** | Malignant neoplasm of skin of lower leg |
| **B105.00** | Malignant neoplasm of lower third of oesophagus |
| **ZV10011** | [V]Personal history of malignant neoplasm of anus |
| **B18y600** | Malignant neoplasm of the pouch of Douglas |
| **B337300** | Malignant neoplasm of skin of popliteal fossa area |
| **BB58.00** | [M]Carcinoma, diffuse type |
| **BB5RA00** | [M]Merkel cell carcinoma |
| **N237300** | Pseudosarcomatous fibromatosis |
| **B450z00** | Malignant neoplasm of vagina NOS |
| **BB2E.00** | [M]Squamous cell carcinoma, small cell, non-keratinising |
| **B484.00** | Malignant neoplasm of epididymis |
| **BB3E.00** | [M]Basal cell carcinoma, micronodular |
| **B832.00** | Carcinoma in situ of other and unspecified parts of uterus |
| **B015.00** | Malignant neoplasm of tongue, junctional zone |
| **BBZG.11** | [M]Adamantinoma, malignant |
| **B150200** | Primary angiosarcoma of liver |
| **BBe9.00** | [M]Triton tumour, malignant |
| **B173.00** | Malignant neoplasm of pancreatic duct |
| **BB1C.00** | [M]Giant cell carcinoma |
| **142..00** | H/O: malignant neoplasm (*) |
| **BBK3300** | [M]Mixed cell rhabdomyosarcoma |
| **B481.00** | Malignant neoplasm of glans penis |
| **B4A0.00** | Malignant neoplasm of kidney parenchyma |
| **B6z0.00** | Kaposi's sarcoma of lymph nodes |
| **B142000** | Malignant neoplasm of cloacogenic zone |
| **B81y100** | Carcinoma in situ of nasal cavity |
| **B49..00** | Malignant neoplasm of urinary bladder |
| **B828X00** | Melanoma in situ of other and unspecified parts of face |
| **BBVA.00** | [M] Small cell osteosarcoma |
| **B34..00** | Malignant neoplasm of female breast |
| **B064z00** | Malignant neoplasm of anterior epiglottis NOS |
| **B203.00** | Malignant neoplasm of ethmoid sinus |
| **B3y..00** | Malig neop of bone, connective tissue, skin and breast OS |
| **B440.11** | Cancer of ovary |
| **BB84.00** | [M]Mucin-producing adenocarcinoma |
| **B524100** | Malignant neoplasm of peripheral nerve,upp limb,incl should |
| **ByuFG00** | [X]Other carcinoma in situ of breast |
| **BBj0.11** | [M]Lymphogranuloma, malignant |
| **BBEM.00** | [M]Malignant melanoma in giant pigmented naevus |
| **B0zz.00** | Malignant neoplasm of lip, oral cavity and pharynx NOS |
| **BBV3.00** | [M]Fibroblastic osteosarcoma |
| **B543.00** | Malignant neoplasm of pineal gland |
| **BB5f.00** | [M]Thyroid adenoma and adenocarcinoma |
| **B545.00** | Malignant neoplasm of aortic body and other paraganglia |
| **B837.00** | Carcinoma in situ of bladder |
| **B241200** | Malignant neoplasm of myocardium |
| **BB5R100** | [M]Carcinoid tumour, malignant |
| **B111.00** | Malignant neoplasm of pylorus of stomach |
| **BB2A.12** | [M]Spinous cell carcinoma |
| **B46..00** | Malignant neoplasm of prostate |
| **B483.00** | Malignant neoplasm of penis, part unspecified |
| **ZV10512** | [V]Personal history of malignant neoplasm of kidney |
| **B002300** | Malignant neoplasm of upper lip, oral aspect |
| **ZV10416** | [V]Personal history of malignant neoplasm of testis |
| **BBEG.00** | [M]Malignant melanoma in Hutchinson's melanotic freckle |
| **B082.00** | Malignant neoplasm aryepiglottic fold, hypopharyngeal aspect |
| **B513.00** | Malignant neoplasm of parietal lobe |
| **BB1G.00** | [M]Spheroidal cell carcinoma |
| **B34y.00** | Malignant neoplasm of other site of female breast |
| **B137.00** | Malignant neoplasm of splenic flexure of colon |
| **BBX3.00** | [M]Malignant giant cell tumour of soft parts |
| **B314.00** | Malignant neoplasm of connective and soft tissue of abdomen |
| **B151z00** | Malignant neoplasm of intrahepatic bile ducts NOS |
| **BB4z.00** | [M]Transitional cell papilloma or carcinoma NOS |
| **BBV..11** | [M]Juxtacortical osteogenic sarcoma |
| **B306100** | Malignant neoplasm of ischium |
| **B35zz00** | Malignant neoplasm of male breast NOS |
| **Byu5.00** | [X]Malignant neoplasm of mesothelial and soft tissue |
| **BBEz.00** | [M]Naevi or melanoma NOS |
| **B302100** | Malignant neoplasm of thoracic vertebra |
| **B112.00** | Malignant neoplasm of pyloric antrum of stomach |
| **BBV1.11** | [M]Osteoblastic sarcoma |
| **B545100** | Malignant neoplasm of aortic body |
| **B512z00** | Malignant neoplasm of temporal lobe NOS |
| **B307000** | Malignant neoplasm of femur |
| **BBX1.00** | [M]Giant cell tumour of bone, malignant |
| **B512000** | Malignant neoplasm of hippocampus |
| **BB85z00** | [M]Signet ring carcinoma NOS |
| **BBP5.00** | [M]Epithelioid mesothelioma, malignant |
| **B33X.00** | Malignant neoplasm overlapping lesion of skin |
| **B002100** | Malignant neoplasm of upper lip, frenulum |
| **BBZ2.11** | [M]Intraosseous carcinoma |
| **B312400** | Malignant neoplasm of connective and soft tissue of foot |
| **B8z..00** | Carcinoma in situ NOS |
| **Byu9.00** | [X]Malignant neoplasm of urinary tract |
| **B340100** | Malignant neoplasm of areola of female breast |
| **B327100** | Malignant melanoma of thigh |
| **B49y000** | Malignant neoplasm, overlapping lesion of bladder |
| **BB5D.00** | [M]Hepatobiliary tract adenomas and carcinomas |
| **B0z1.00** | Malignant neoplasm of Waldeyer's ring |
| **B003300** | Malignant neoplasm of lower lip, oral aspect |
| **B021.00** | Malignant neoplasm of submandibular gland |
| **B827200** | Carcinoma in situ of skin of knee |
| **ZV10412** | [V]Personal history of malignant neoplasm of genital organ |
| **B825.00** | Carcinoma in situ of skin of trunk, excluding scrotum |
| **B340.00** | Malignant neoplasm of nipple and areola of female breast |
| **B300.00** | Malignant neoplasm of bones of skull and face |
| **BB82114** | [M]Mucous adenocarcinoma |
| **BB5jz00** | [M]Endometrioid adenoma or carcinoma NOS |
| **B1z..00** | Malig neop oth/ill-defined sites digestive tract/peritoneum |
| **B213z00** | Malignant neoplasm of laryngeal cartilage NOS |
| **B342.00** | Malignant neoplasm of upper-inner quadrant of female breast |
| **BB9M.00** | [M]Intracystic carcinoma NOS |
| **B060z00** | Malignant neoplasm tonsil NOS |
| **B540.00** | Malignant neoplasm of adrenal gland |
| **BBF6.00** | [M]Epithelioid cell sarcoma |
| **BB9J.11** | [M]Paget's disease, breast |
| **B335A00** | Malignant neoplasm of skin of scapular region |
| **B003200** | Malignant neoplasm of lower lip, mucosa |
| **B503.00** | Malignant neoplasm of conjunctiva |
| **BB24.11** | [M]Verrucous epidermoid carcinoma |
| **B33..00** | Other malignant neoplasm of skin |
| **B521z00** | Malignant neoplasm of cerebral meninges NOS |
| **BB80100** | [M]Cystadenocarcinoma NOS |
| **BBZ2.00** | [M]Odontogenic tumour, malignant |
| **BBT1.11** | [M]Angiosarcoma |
| **B326100** | Malignant melanoma of upper arm |
| **BB06.00** | [M]Tumour cells, uncertain whether benign or malignant |
| **B42..00** | Malignant neoplasm of placenta |
| **B151000** | Malignant neoplasm of interlobular bile ducts |
| **BBV..00** | [M]Osteomas and osteosarcomas |
| **B212.00** | Malignant neoplasm of subglottis |
| **BBW4.00** | [M]Chondrosarcoma NOS |
| **BBN5.00** | [M]Clear cell sarcoma of tendons and aponeuroses |
| **BB9D.00** | [M]Medullary carcinoma with lymphoid stroma |
| **B014.00** | Malignant neoplasm of anterior 2/3 of tongue unspecified |
| **BB07.00** | [M]Tumour cells, malignant |
| **B440.00** | Malignant neoplasm of ovary |
| **BB5b.00** | [M]Granular cell carcinoma |
| **B831100** | Carcinoma in situ of exocervix |
| **B8y0.00** | Carcinoma in situ of eye |
| **B826.00** | Carcinoma in situ of skin of upper limb and shoulder |
| **B081.00** | Malignant neoplasm of pyriform sinus |
| **B45X.00** | Malignant neoplasm/overlapping lesion/feml genital organs |
| **BB1N.00** | [M]Small cell-large cell carcinoma |
| **B062000** | Malignant neoplasm of faucial pillar |
| **B22..00** | Malignant neoplasm of trachea, bronchus and lung |
| **BB5U.00** | [M]Villous adenomas and adenocarcinomas |
| **ZV10y14** | [V]Personal history of malignant neoplasm of skin |
| **B453.00** | Malignant neoplasm of clitoris |
| **B33..15** | Malignant neoplasm of sweat gland |
| **B300200** | Malignant neoplasm of malar bone |
| **BBJ1.00** | [M]Liposarcoma NOS |
| **B346.00** | Malignant neoplasm of axillary tail of female breast |
| **B050.11** | Malignant neoplasm of buccal mucosa |
| **ZV10513** | [V]Personal history of malignant neoplasm of kidney |
| **B517z00** | Malignant neoplasm of brain stem NOS |
| **B001z00** | Malignant neoplasm of lower lip, vermilion border NOS |
| **B4Ay000** | Malignant neoplasm of overlapping lesion of urinary organs |
| **B808100** | Carcinoma in situ of intrahepatic bile ducts |
| **B806.00** | Carcinoma in situ of anus NOS |
| **B80z000** | Carcinoma in situ of pancreas |
| **B1z0.11** | Cancer of bowel |
| **B051000** | Malignant neoplasm of upper buccal sulcus |
| **B49y.00** | Malignant neoplasm of other site of urinary bladder |
| **BBK2.00** | [M]Myoma and myosarcoma |
| **BBQA100** | [M]Struma ovarii, malignant |
| **BB3D.00** | [M]Basal cell carcinoma, nodular |
| **B332.00** | Malignant neoplasm skin of ear and external auricular canal |
| **BB5V311** | [M]Eosinophil carcinoma |
| **BB90.00** | [M]Intraductal carcinoma, noninfiltrating NOS |
| **B35z000** | Malignant neoplasm of ectopic site of male breast |
| **B305C00** | Malignant neoplasm of fifth metacarpal bone |
| **B224000** | Malignant neoplasm of lower lobe bronchus |
| **B243.00** | Malignant neoplasm of posterior mediastinum |
| **B204.00** | Malignant neoplasm of frontal sinus |
| **B430211** | Malignant neoplasm of endometrium |
| **BBF1.00** | [M]Sarcoma NOS |
| **ZV10y13** | [V]Personal history of malignant neoplasm of eye |
| **BBF3.00** | [M]Spindle cell sarcoma |
| **B18y700** | Malignant neoplasm of mesentery |
| **B343.00** | Malignant neoplasm of lower-inner quadrant of female breast |
| **B490.00** | Malignant neoplasm of trigone of urinary bladder |
| **B0...00** | Malignant neoplasm of lip, oral cavity and pharynx |
| **B110z00** | Malignant neoplasm of cardia of stomach NOS |
| **B508.00** | Malignant neoplasm, overlapping lesion of eye and adnexa |
| **B59zX00** | Kaposi's sarcoma, unspecified |
| **BB5Dz00** | [M]Hepatobiliary adenoma or carcinoma NOS |
| **B327000** | Malignant melanoma of hip |
| **BBE1100** | [M]Desmoplastic melanoma, malignant |
| **BB5D500** | [M]Hepatocellular carcinoma NOS |
| **BB5D512** | [M]Hepatoma, malignant |
| **B304400** | Malignant neoplasm of ulna |
| **B8...00** | Carcinoma in situ |
| **B22z.00** | Malignant neoplasm of bronchus or lung NOS |
| **B10y.00** | Malignant neoplasm of other specified part of oesophagus |
| **B480.00** | Malignant neoplasm of prepuce (foreskin) |
| **B325400** | Malignant melanoma of perianal skin |
| **B335600** | Malignant neoplasm of skin of perineum |
| **B51y000** | Malignant neoplasm of corpus callosum |
| **BB69z00** | [M]Sebaceous adenoma or adenocarcinoma NOS |
| **B823000** | Carcinoma in situ of skin of forehead skin |
| **SN5y100** | Malignant hypothermia due to anaesthetic |
| **B47z.00** | Malignant neoplasm of testis NOS |
| **BB51100** | [M]Adenocarcinoma in situ in tubulovillous adenoma |
| **B325.00** | Malignant melanoma of trunk (excluding scrotum) |
| **B340z00** | Malignant neoplasm of nipple or areola of female breast NOS |
| **B833000** | Carcinoma in situ of ovary |
| **B13..00** | Malignant neoplasm of colon |
| **B331.00** | Malignant neoplasm of eyelid including canthus |
| **B523z00** | Malignant neoplasm of spinal meninges NOS |
| **B8yy300** | Carcinoma in situ of pituitary gland |
| **BB18.00** | [M]Carcinoma, undifferentiated type, NOS |
| **B10z.00** | Malignant neoplasm of oesophagus NOS |
| **BB5L.00** | [M]Adenomatous and adenocarcinomatous polyps |
| **B81y600** | Carcinoma in situ of maxillary sinus |
| **B550200** | Malignant neoplasm of nose NOS |
| **BBT1.00** | [M]Haemangiosarcoma |
| **ByuA000** | [X]Malignant neoplasm/other and unspecified cranial nerves |
| **B826100** | Carcinoma in situ of skin of upper arm |
| **B18y.00** | Malignant neoplasm of specified parts of peritoneum |
| **ZV67700** | [V]Follow-up exam after radiotherapy for malignant neoplasm |
| **B304.00** | Malignant neoplasm of scapula and long bones of upper arm |
| **B828700** | Melanoma in situ of scalp |
| **B801000** | Carcinoma in situ of upper 1/3 oesophagus |
| **BB5N.11** | [M]Adenoma or or adenocarcinoma in polyposis coli |
| **B00..00** | Malignant neoplasm of lip |
| **B6z..00** | Malignant neoplasm lymphatic or haematopoietic tissue NOS |
| **B325100** | Malignant melanoma of breast |
| **B07y.00** | Malignant neoplasm of other specified site of nasopharynx |
| **BB36.00** | [M]Metatypical carcinoma |
| **B012.00** | Malignant neoplasm of tongue, tip and lateral border |
| **B35z.00** | Malignant neoplasm of other site of male breast |
| **B300100** | Malignant neoplasm of frontal bone |
| **142..12** | H/O: carcinoma |
| **B332z00** | Malig neop skin of ear and external auricular canal NOS |
| **B118.00** | Siewert type II adenocarcinoma |
| **B306z00** | Malignant neoplasm of pelvis, sacrum or coccyx NOS |
| **B1z2.00** | Malignant neoplasm, overlapping lesion of digestive system |
| **B802000** | Carcinoma in situ of cardia of stomach |
| **B062200** | Malignant neoplasm of palatoglossal arch |
| **B441.00** | Malignant neoplasm of fallopian tube |
| **B062100** | Malignant neoplasm of glossopalatine fold |
| **B804z00** | Carcinoma in situ of rectum or rectosigmoid junction NOS |
| **B013000** | Malignant neoplasm of anterior 2/3 of tongue ventral surface |
| **B23y.00** | Malignant neoplasm of other specified pleura |
| **D212000** | Anaemia in ovarian carcinoma |
| **B052.00** | Malignant neoplasm of hard palate |
| **BB5W100** | [M]Oxyphilic adenocarcinoma |
| **B500z00** | Malignant neoplasm of eyeball NOS |
| **BBK3700** | [M]Alveolar rhabdomyosarcoma |
| **B337500** | Malignant neoplasm of skin of ankle |
| **BB82.00** | [M]Mucinous adenoma and adenocarcinoma |
| **B34y000** | Malignant neoplasm of ectopic site of female breast |
| **B063.00** | Malignant neoplasm of vallecula |
| **BBJ6.00** | [M]Round cell liposarcoma |
| **B807100** | Carcinoma in situ of jejunum |
| **B327300** | Malignant melanoma of popliteal fossa area |
| **BBGP.00** | [M]Pigmented dermatofibrosarcoma protuberans |
| **BB2G.00** | [M]Adenoid squamous cell carcinoma |
| **B327z00** | Malignant melanoma of lower limb or hip NOS |
| **B305.11** | Malignant neoplasm of carpal bones |
| **BBG8.00** | [M]Infantile fibrosarcoma |
| **B305100** | Malignant neoplasm of carpal bone - lunate |
| **B350000** | Malignant neoplasm of nipple of male breast |
| **B21..00** | Malignant neoplasm of larynx |
| **4M74.00** | Clark melanoma level 5 |
| **B826z00** | Carcinoma in situ of skin of upper limb or shoulder NOS |
| **BB51.00** | [M]Adenocarcinoma in situ |
| **B33z.00** | Malignant neoplasm of skin NOS |
| **B308.00** | Malignant neoplasm of short bones of leg |
| **BB5cz00** | [M]Parathyroid adenoma or adenocarcinoma NOS |
| **B520200** | Malignant neoplasm of acoustic nerve |
| **BBK3611** | [M]Sarcoma botryoides |
| **B311100** | Malignant neoplasm of connective and soft tissue, upper arm |
| **BBF..00** | [M]Soft tissue tumours and sarcomas NOS |
| **B224z00** | Malignant neoplasm of lower lobe, bronchus or lung NOS |
| **BB4A.00** | [M]Papillary transitional cell carcinoma |
| **B062.00** | Malignant neoplasm of tonsillar pillar |
| **BB1J.12** | [M]Round cell carcinoma |
| **ZV10z00** | [V]Personal history of unspecified malignant neoplasm |
| **Byu1.00** | [X]Malignant neoplasm of digestive organs |
| **B808000** | Carcinoma in situ of liver |
| **B524W00** | Mal neoplasm/periph nerves+autonomic nervous system,unspc |
| **B303200** | Malignant neoplasm of clavicle |
| **ZV67A00** | [V]Folow-up exam aft other treatment for malignant neoplasm |
| **BB5B500** | [M]Glucagonoma, malignant |
| **B833100** | Carcinoma in situ of fallopian tube |
| **Byu1100** | [X]Other specified carcinomas of liver |
| **ZV10300** | [V]Personal history of malignant neoplasm of breast |
| **BB5j500** | [M]Endometrioid adenofibroma, malignant |
| **B828300** | Melanoma in situ of scalp and neck |
| **BBE..00** | [M]Naevi and melanomas |
| **BB24.12** | [M]Verrucous squamous cell carcinoma |
| **B31z000** | Kaposi's sarcoma of soft tissue |
| **BB26.11** | [M]Papillary epidermoid carcinoma |
| **B808600** | Carcinoma in situ of ampulla of Vater |
| **BB96.00** | [M]Noninfiltrating intraductal papillary adenocarcinoma |
| **B241300** | Malignant neoplasm of pericardium |
| **B524200** | Malignant neoplasm of peripheral nerve of low limb, incl hip |
| **B306200** | Malignant neoplasm of pubis |
| **BB5f200** | [M]Follicular adenocarcinoma, well differentiated type |
| **BBF5.11** | [M]Round cell sarcoma |
| **B550300** | Malignant neoplasm of jaw NOS |
| **BB34.00** | [M]Basal cell carcinoma, fibroepithelial type |
| **B823300** | Carcinoma in situ of skin of cheek |
| **BB5S.00** | [M]Respiratory tract adenomas and adenocarcinomas |
| **Byu9000** | [X]Malignant neoplasm of urinary organ, unspecified |
| **B073z00** | Malignant neoplasm of anterior wall of nasopharynx NOS |
| **BBE1.13** | [M]Melanosarcoma NOS |
| **B01z.00** | Malignant neoplasm of tongue NOS |
| **B800700** | Carcinoma in situ of nasopharynx |
| **BB29.12** | [M]Intraepidermal carcinoma NOS |
| **BBCC100** | [M]Leydig cell tumour, malignant |
| **B170.00** | Malignant neoplasm of head of pancreas |
| **B225.00** | Malignant neoplasm of overlapping lesion of bronchus & lung |
| **BB61.00** | [M]Sweat gland adenoma and adenocarcinomas |
| **BB5B.00** | [M]Pancreatic adenomas and carcinomas |
| **B494.00** | Malignant neoplasm of posterior wall of urinary bladder |
| **BBb0.12** | [M]Gliosarcoma |
| **Byu4.00** | [X]Melanoma and other malignant neoplasms of skin |
| **B300800** | Malignant neoplasm of temporal bone |
| **B43z.00** | Malignant neoplasm of body of uterus NOS |
| **B59..00** | Malignant neoplasm of unspecified site |
| **BB5D800** | [M]Hepatocellular carcinoma, fibrolamellar |
| **BB94.00** | [M]Juvenile breast carcinoma |
| **B122.00** | Malignant neoplasm of ileum |
| **B111z00** | Malignant neoplasm of pylorus of stomach NOS |
| **BBF2.00** | [M]Sarcomatosis NOS |
| **B827000** | Carcinoma in situ of skin of hip |
| **B116.00** | Malignant neoplasm of greater curve of stomach unspecified |
| **BBE2.00** | [M]Nodular melanoma |
| **B524.00** | Malig neopl peripheral nerves and autonomic nervous system |
| **B007.00** | Malignant neoplasm of lip, unspecified |
| **BB17.00** | [M]Large cell carcinoma NOS |
| **B072.00** | Malignant neoplasm of lateral wall of nasopharynx |
| **B001000** | Malignant neoplasm of lower lip, external |
| **B335800** | Malignant neoplasm of skin of buttock |
| **B0z2.00** | Malignant neoplasm of laryngopharynx |
| **BBW4.11** | [M]Fibrochondrosarcoma |
| **BB5j200** | [M]Endometrioid carcinoma |
| **B430300** | Malignant neoplasm of myometrium of corpus uteri |
| **B4A..11** | Renal malignant neoplasm |
| **B540000** | Malignant neoplasm of adrenal cortex |
| **B151.00** | Malignant neoplasm of intrahepatic bile ducts |
| **B11y.00** | Malignant neoplasm of other specified site of stomach |
| **B802400** | Carcinoma in situ of pyloric canal |
| **B222000** | Malignant neoplasm of upper lobe bronchus |
| **B470.00** | Malignant neoplasm of undescended testis |
| **B10..00** | Malignant neoplasm of oesophagus |
| **BBW6.00** | [M]Juxtacortical chondrosarcoma |
| **BBQ7200** | [M]Teratoma, malignant, NOS |
| **B064000** | Malignant neoplasm of epiglottis, free border |
| **B210.00** | Malignant neoplasm of glottis |
| **1425000** | H/O Malignant melanoma |
| **B031.00** | Malignant neoplasm of lower gum |
| **B827.11** | Carcinoma in situ of skin of leg |
| **B104.00** | Malignant neoplasm of middle third of oesophagus |
| **B812100** | Carcinoma in situ of main bronchus |
| **B803300** | Carcinoma in situ of sigmoid colon |
| **BB81B00** | [M]Serous surface papillary carcinoma |
| **B551100** | Malignant neoplasm of chest wall NOS |
| **B812200** | Carcinoma in situ of upper lobe bronchus and lung |
| **B300A00** | Malignant neoplasm of maxilla |
| **B430z00** | Malignant neoplasm of corpus uteri NOS |
| **B808.00** | Carcinoma in situ of liver and biliary system |
| **BBTK.00** | [M]Epithelioid haemangioendothelioma, malignant |
| **Byu8000** | [X]Malignant neoplasm/other specified male genital organs |
| **B802.00** | Carcinoma in situ of stomach |
| **B554.00** | Malignant neoplasm of upper limb NOS |
| **B180100** | Malignant neoplasm of perinephric tissue |
| **B807200** | Carcinoma in situ of ileum |
| **B004300** | Malignant neoplasm of lip, oral aspect |
| **B11yz00** | Malignant neoplasm of other specified site of stomach NOS |
| **B150300** | Hepatocellular carcinoma |
| **B310500** | Malignant neoplasm soft tissues of cervical spine |
| **BB5T100** | [M]Papillary adenocarcinoma NOS |
| **BB5h100** | [M]Adrenal cortical carcinoma |
| **B4A1100** | Malignant neoplasm of ureteropelvic junction |
| **B002200** | Malignant neoplasm of upper lip, mucosa |
| **B800300** | Carcinoma in situ of gums |
| **BB5U200** | [M]Villous adenocarcinoma |
| **BB94.11** | [M]Secretory breast carcinoma |
| **B320.00** | Malignant melanoma of lip |
| **B331000** | Malignant neoplasm of canthus |
| **BB9C.00** | [M]Medullary carcinoma with amyloid stroma |
| **Byu8200** | [X]Malignant neoplasm of male genital organ, unspecified |
| **BB1F.00** | [M]Polygonal cell carcinoma |
| **B119.00** | Siewert type III adenocarcinoma |
| **B323200** | Malignant melanoma of eyebrow |
| **B2zy.00** | Malignant neoplasm of other site of respiratory tract |
| **B12..00** | Malignant neoplasm of small intestine and duodenum |
| **B541.00** | Malignant neoplasm of parathyroid gland |
| **B8yy100** | Carcinoma in situ of adrenal gland |
| **B48y200** | Malignant neoplasm, overlapping lesion male genital orgs |
| **BBV5.00** | [M]Osteosarcoma in Paget's disease of bone |
| **B106.00** | Malignant neoplasm, overlapping lesion of oesophagus |
| **B550.00** | Malignant neoplasm of head, neck and face |
| **ByuF600** | [X]Melanoma in situ of other sites |
| **B013.00** | Malignant neoplasm of ventral surface of tongue |
| **B313000** | Malignant neoplasm of connective and soft tissue of axilla |
| **BBV1.12** | [M]Osteochondrosarcoma |
| **BB29.13** | [M]Intraepithelial squamous cell carcinoma |
| **B010z00** | Malignant neoplasm of fixed part of tongue NOS |
| **B24z.00** | Malignant neoplasm of heart, thymus and mediastinum NOS |
| **B303000** | Malignant neoplasm of rib |
| **B131.00** | Malignant neoplasm of transverse colon |
| **B162.00** | Malignant neoplasm of ampulla of Vater |
| **B30X.00** | Malignant neoplasm/bones+articular cartilage/limb,unspfd |
| **ZV10111** | [V]Personal history of malignant neoplasm of bronchus |
| **BBn3.00** | [M]Plasma cell tumour, malignant |
| **B810z00** | Carcinoma in situ of larynx NOS |
| **B304300** | Malignant neoplasm of radius |
| **B327800** | Malignant melanoma of toe |
| **B150100** | Hepatoblastoma of liver |
| **B221z00** | Malignant neoplasm of main bronchus NOS |
| **B18y100** | Malignant neoplasm of mesocaecum |
| **B223000** | Malignant neoplasm of middle lobe bronchus |
| **B310100** | Malignant neoplasm of soft tissue of face |
| **B8y..00** | Carcinoma in situ of other and unspecified sites |
| **BB1K.00** | [M]Oat cell carcinoma |
| **B312500** | Malignant neoplasm of connective and soft tissue of toe |
| **B324z00** | Malignant melanoma of scalp and neck NOS |
| **BB56.00** | [M]Superficial spreading adenocarcinoma |
| **BB5Wz00** | [M]Oxyphilic adenoma or adenocarcinoma NOS |
| **BBrA312** | [M]Granulocytic sarcoma |
| **B802z00** | Carcinoma in situ of stomach NOS |
| **B4A..00** | Malig neop of kidney and other unspecified urinary organs |
| **B305A00** | Malignant neoplasm of third metacarpal bone |
| **BB2A.00** | [M]Squamous cell carcinoma NOS |
| **B501000** | Malignant neoplasm of connective tissue of orbit |
| **B812400** | Carcinoma in situ of lower lobe bronchus and lung |
| **B21y.00** | Malignant neoplasm of larynx, other specified site |
| **BB5C100** | [M]Gastrinoma, malignant |
| **B065.00** | Malignant neoplasm of junctional region of epiglottis |
| **B803000** | Carcinoma in situ of hepatic flexure of colon |
| **B52..00** | Malig neop of other and unspecified parts of nervous system |
| **B123.00** | Malignant neoplasm of Meckel's diverticulum |
| **B831000** | Carcinoma in situ of endocervix |
| **B555.00** | Malignant neoplasm of lower limb NOS |
| **BB5a.00** | [M]Renal adenoma and carcinoma |
| **B828400** | Melanoma in situ of trunk |
| **BBd2.00** | [M]Meningioma, malignant |
| **B221100** | Malignant neoplasm of hilus of lung |
| **B4...00** | Malignant neoplasm of genitourinary organ |
| **BB91000** | [M]Intraductal papillary adenocarcinoma with invasion |
| **B325600** | Malignant melanoma of umbilicus |
| **BB5T.00** | [M]Papillary adenomas and adenocarcinomas |
| **B550500** | Malignant neoplasm of supraclavicular fossa NOS |
| **B180z00** | Malignant neoplasm of retroperitoneum NOS |
| **B5z..00** | Malignant neoplasm of other and unspecified site NOS |
| **B332200** | Malignant neoplasm of pinna NEC |
| **B807z00** | Carcinoma in situ other and unspecified small intestine NOS |
| **B310400** | Malignant neoplasm of tarsus of eyelid |
| **BB5c.00** | [M]Parathyroid adenomas and adenocarcinomas |
| **B151400** | Malignant neoplasm of intrahepatic gall duct |
| **B81y900** | Carcinoma in situ of sphenoidal sinus |
| **BB1D.00** | [M]Spindle cell carcinoma |
| **B110111** | Malignant neoplasm of gastro-oesophageal junction |
| **B803100** | Carcinoma in situ of transverse colon |
| **BBG8.11** | [M]Congenital fibrosarcoma |
| **ByuF300** | [X]Carcinoma in situ of other parts of respiratory system |
| **BB49.00** | [M]Cloacogenic carcinoma |
| **BB5S212** | [M]Bronchiolar carcinoma |
| **B335500** | Malignant neoplasm of skin of groin |
| **BB9G.00** | [M]Infiltrating ductular carcinoma |
| **B327900** | Malignant melanoma of great toe |
| **B011z00** | Malignant neoplasm of dorsum of tongue NOS |
| **B120.00** | Malignant neoplasm of duodenum |
| **B491.00** | Malignant neoplasm of dome of urinary bladder |
| **B040.00** | Malignant neoplasm of anterior portion of floor of mouth |
| **B825000** | Carcinoma in situ of skin of breast |
| **Byu5400** | [X]Malignant neoplasm/peripheral nerves of trunk,unspecified |
| **B182.00** | Overlapping malign lesion of retroperitoneum and peritoneum |
| **B0zy.00** | Malignant neoplasm of other sites lip, oral cavity, pharynx |
| **B06z.00** | Malignant neoplasm of oropharynx NOS |
| **B812z00** | Carcinoma in situ of bronchus or lung NOS |
| **B15..00** | Malignant neoplasm of liver and intrahepatic bile ducts |
| **B18y400** | Malignant neoplasm of parietal peritoneum |
| **B142.00** | Malignant neoplasm of anal canal |
| **B072z00** | Malignant neoplasm of lateral wall of nasopharynx NOS |
| **B51yz00** | Malignant neoplasm of other part of brain NOS |
| **B801.00** | Carcinoma in situ of oesophagus |
| **B516.00** | Malignant neoplasm of cerebellum |
| **B311500** | Malignant neoplasm of connective and soft tissue of thumb |
| **Byu7100** | [X]Malignant neoplasm/other specified female genital organs |
| **BB5M.00** | [M]Tubular adenomas and adenocarcinomas |
| **B334z00** | Malignant neoplasm of scalp or skin of neck NOS |
| **B313200** | Malignant neoplasm of great vessels |
| **B010.11** | Malignant neoplasm of posterior third of tongue |
| **BBE1.00** | [M]Malignant melanoma NOS |
| **B827z00** | Carcinoma in situ of skin of lower limb or hip NOS |
| **BB9K.00** | [M]Paget's disease and infiltrating breast duct carcinoma |
| **BB5N100** | [M]Adenocarcinoma in adenomatous polposis coli |
| **BBJ5.12** | [M]Myxoliposarcoma |
| **B326200** | Malignant melanoma of fore-arm |
| **B800900** | Carcinoma in situ of hypopharynx |
| **B410000** | Malignant neoplasm of endocervical canal |
| **B223z00** | Malignant neoplasm of middle lobe, bronchus or lung NOS |
| **Byu3.00** | [X]Malignant neoplasm of bone and articular cartilage |
| **BBG1.00** | [M]Fibrosarcoma NOS |
| **BB09.00** | [M]Malignant tumour, giant cell type |
| **ZV10214** | [V]Personal history of malignant neoplasm of nose |
| **BB02.00** | [M]Neoplasm, malignant |
| **B05..00** | Malignant neoplasm of other and unspecified parts of mouth |
| **BB5V100** | [M]Chromophobe carcinoma |
| **BBL9.00** | [M]Carcinosarcoma NOS |
| **ZV10.00** | [V]Personal history of malignant neoplasm |
| **B517100** | Malignant neoplasm of medulla oblongata |
| **B180.00** | Malignant neoplasm of retroperitoneum |
| **B44z.00** | Malignant neoplasm of uterine adnexa NOS |
| **BBM7.11** | [M]Cystosarcoma phyllodes, benign |
| **B810800** | Carcinoma in situ of vocal fold - glottis |
| **B000z00** | Malignant neoplasm of upper lip, vermilion border NOS |
| **BB5B300** | [M]Insulinoma, malignant |
| **B823100** | Carcinoma in situ of skin of eyebrow |
| **BB5D111** | [M]Bile duct carcinoma |
| **BBV1.00** | [M]Osteosarcoma NOS |
| **BBE1.12** | [M]Melanoma NOS |
| **BB5f700** | [M]Nonencapsulated sclerosing carcinoma |
| **BB5j.00** | [M]Endometrioid adenomas and carcinomas |
| **B411.00** | Malignant neoplasm of exocervix |
| **B060000** | Malignant neoplasm of faucial tonsil |
| **B50y.00** | Malignant neoplasm of other specified site of eye |
| **BBQ7300** | [M]Teratocarcinoma |
| **BBZC.00** | [M]Ameloblastic odontosarcoma |
| **B334000** | Malignant neoplasm of scalp |
| **B334.00** | Malignant neoplasm of scalp and skin of neck |
| **BBC4.00** | [M]Granulosa cell tumour, malignant |
| **7G05D00** | Excision biopsy of basal cell carcinoma |
| **BB11.11** | [M]Intraepithelial carcinoma NOS |
| **BB2F.00** | [M]Squamous cell carcinoma, spindle cell type |
| **BB5R600** | [M]Mucocarcinoid tumour, malignant |
| **ZV10415** | [V]Personal history of malignant neoplasm of prostate |
| **B142.11** | Anal carcinoma |
| **F122.00** | Malignant neuroleptic syndrome |
| **B325200** | Malignant melanoma of buttock |
| **ByuB.00** | [X]Malignant neoplasm of thyroid and other endocrine glands |
| **B222z00** | Malignant neoplasm of upper lobe, bronchus or lung NOS |
| **B333.00** | Malignant neoplasm skin of other and unspecified parts face |
| **B005.00** | Malignant neoplasm of commissure of lip |
| **B545200** | Malignant neoplasm of coccygeal body |
| **B073.00** | Malignant neoplasm of anterior wall of nasopharynx |
| **B300z00** | Malignant neoplasm of bones of skull and face NOS |
| **B1zy.00** | Malignant neoplasm other spec digestive tract and peritoneum |
| **BBJ8.00** | [M]Mixed type liposarcoma |
| **4F32.00** | Ascitic fluid: malignant cells |
| **B222.00** | Malignant neoplasm of upper lobe, bronchus or lung |
| **B1...11** | Carcinoma of digestive organs and peritoneum |
| **Byu2.00** | [X]Malignant neoplasm of respiratory and intrathoracic orga |
| **B803.00** | Carcinoma in situ of colon |
| **BBK0700** | [M]Myxoid leiomyosarcoma |
| **B174.00** | Malignant neoplasm of Islets of Langerhans |
| **ZV10014** | [V]Personal history of malignant neoplasm of large intestine |
| **B326z00** | Malignant melanoma of upper limb or shoulder NOS |
| **BB5B100** | [M]Islet cell carcinoma |
| **BBN2.00** | [M]Synovial sarcoma, spindle cell type |
| **B810700** | Carcinoma in situ of vestibular fold |
| **B053.00** | Malignant neoplasm of soft palate |
| **B0z..00** | Malig neop other/ill-defined sites lip, oral cavity, pharynx |
| **B003z00** | Malignant neoplasm of lower lip, inner aspect NOS |
| **B826300** | Carcinoma in situ of skin of hand |
| **B517000** | Malignant neoplasm of cerebral peduncle |
| **B230.00** | Malignant neoplasm of parietal pleura |
| **BB5Tz00** | [M]Papillary adenoma or adenocarcinoma NOS |
| **B113.00** | Malignant neoplasm of fundus of stomach |
| **B242.00** | Malignant neoplasm of anterior mediastinum |
| **B1z1100** | Fibrosarcoma of spleen |
| **Byu0.00** | [X]Malignant neoplasm of lip, oral cavity and pharynx |
| **ZV10211** | [V]Personal history of malignant neoplasm - accessory sinus |
| **Byu5700** | [X]Malignant neoplasm of peritoneum, unspecified |
| **B16..00** | Malignant neoplasm gallbladder and extrahepatic bile ducts |
| **B54X.00** | Malignant neoplasm-pluriglandular involvement,unspecified |
| **BB46.00** | [M]Schneiderian carcinoma |
| **B331200** | Malignant neoplasm of lower eyelid |
| **B201100** | Malignant neoplasm of tympanic cavity |
| **B82z.00** | Carcinoma in situ of skin NOS |
| **B803600** | Carcinoma in situ of ascending colon |
| **BBX1.12** | [M]Osteoclastoma, malignant |
| **B83z.00** | Carcinoma in situ of urinary organs NOS |
| **BB5Bz00** | [M]Pancreatic adenoma or carcinoma NOS |
| **B54y.00** | Malignant neoplasm of other specified endocrine gland |
| **B072000** | Malignant neoplasm of pharyngeal recess |
| **BBF5.00** | [M]Small cell sarcoma |
| **B313100** | Malignant neoplasm of diaphragm |
| **B323.00** | Malignant melanoma of other and unspecified parts of face |
| **B83..00** | Carcinoma in situ of breast and genitourinary system |
| **BBM8.00** | [M]Cystosarcoma phyllodes NOS |
| **Byu5900** | [X]Malignant neoplasm/connective + soft tissue,unspecified |
| **B800.12** | Carcinoma in situ of pharynx |
| **ZV10411** | [V]Personal history of malignant neoplasm of cervix uteri |
| **B03y.00** | Malignant neoplasm of other sites of gum |
| **BBF4.00** | [M]Giant cell sarcoma (except of bone) |
| **B315.00** | Malignant neoplasm of connective and soft tissue of pelvis |
| **B32y.00** | Malignant melanoma of other specified skin site |
| **BBN4.00** | [M]Synovial sarcoma, biphasic type |
| **B823600** | Carcinoma in situ of skin of jaw |
| **B51..11** | Cerebral tumour - malignant |
| **4M71.00** | Clark melanoma level 2 |
| **B524000** | Malignant neoplasm of peripheral nerves of head, face & neck |
| **B301.00** | Malignant neoplasm of mandible |
| **B311200** | Malignant neoplasm of connective and soft tissue of fore-arm |
| **B52X.00** | Malignant neoplasm of meninges, unspecified |
| **B073200** | Malignant neoplasm posterior margin nasal septum and choanae |
| **B051.00** | Malignant neoplasm of vestibule of mouth |
| **B442.00** | Malignant neoplasm of broad ligament |
| **B054.00** | Malignant neoplasm of uvula |
| **B10z.11** | Oesophageal cancer |
| **BBN1.00** | [M]Synovial sarcoma NOS |
| **B16z.00** | Malignant neoplasm gallbladder/extrahepatic bile ducts NOS |
| **ByuF100** | [X]Carcinoma in situ of other specified digestive organs |
| **B48..00** | Malignant neoplasm of penis and other male genital organs |
| **BB51000** | [M]Adenocarcinoma in situ in villous adenoma |
| **B542.00** | Malignant neoplasm pituitary gland and craniopharyngeal duct |
| **B1z1.00** | Malignant neoplasm of spleen NEC |
| **BB5S400** | [M]Alveolar adenocarcinoma |
| **B337100** | Malignant neoplasm of skin of thigh |
| **B803700** | Carcinoma in situ of splenic flexure of colon |
| **B514.00** | Malignant neoplasm of occipital lobe |
| **B303.00** | Malignant neoplasm of ribs, sternum and clavicle |
| **B8yy.00** | Carcinoma in situ of other specified site |
| **B41yz00** | Malignant neoplasm of other site of cervix NOS |
| **BBB4.00** | [M]Adenocarcinoma with spindle cell metaplasia |
| **B545z00** | Malignant neoplasm of aortic body or paraganglia NOS |
| **B55yz00** | Malignant neoplasm of specified site NOS |
| **B13z.00** | Malignant neoplasm of colon NOS |
| **BBEH.00** | [M]Superficial spreading melanoma |
| **BB61200** | [M]Sweat gland adenocarcinoma |
| **B53..00** | Malignant neoplasm of thyroid gland |
| **B201000** | Malignant neoplasm of auditory (Eustachian) tube |
| **B22z.11** | Lung cancer |
| **B310300** | Malignant neoplasm of cartilage of ear |
| **B551z00** | Malignant neoplasm of thorax NOS |
| **B823500** | Carcinoma in situ of skin of temple |
| **ZV10511** | [V]Personal history of malignant neoplasm of bladder |
| **B800500** | Carcinoma in situ of cheek |
| **B335200** | Malignant neoplasm of skin of breast |
| **B240.00** | Malignant neoplasm of thymus |
| **B323400** | Malignant melanoma of external surface of nose |
| **B220100** | Malignant neoplasm of mucosa of trachea |
| **B810100** | Carcinoma in situ of cricoid cartilage |
| **B325000** | Malignant melanoma of axilla |
| **ZV10400** | [V]Personal history of malignant neoplasm of genital organ |
| **B117.00** | Malignant neoplasm, overlapping lesion of stomach |
| **B04z.00** | Malignant neoplasm of floor of mouth NOS |
| **B337800** | Malignant neoplasm of skin of toe |
| **B511.00** | Malignant neoplasm of frontal lobe |
| **B2z..00** | Malig neop other/ill-defined sites resp/intrathoracic organs |
| **B800.00** | Carcinoma in situ of lip, oral cavity and pharynx |
| **BB5Lz00** | [M]Adenomatous or adenocarcinomatous polyp NOS |
| **BBd2.11** | [M]Leptomeningeal sarcoma |
| **B33z000** | Kaposi's sarcoma of skin |
| **B335000** | Malignant neoplasm of skin of axillary fold |
| **ZV10y15** | [V]Personal history of malignant neoplasm of thyroid |
| **B420.00** | Choriocarcinoma |
| **B241z00** | Malignant neoplasm of heart NOS |
| **4M70.00** | Clark melanoma level 1 |
| **BB82112** | [M]Gelatinous adenocarcinoma |
| **B32y000** | Overlapping malignant melanoma of skin |
| **B825100** | Carcinoma in situ of skin of chest wall NOS |
| **BB1L.00** | [M]Small cell carcinoma, fusiform cell type |
| **BB81E11** | [M]Pseudomucinous adenocarcinoma |
| **B070.00** | Malignant neoplasm of roof of nasopharynx |
| **B336300** | Malignant neoplasm of skin of hand |
| **BBQ3.00** | [M]Embryonal carcinoma NOS |
| **B24X.00** | Malignant neoplasm of mediastinum, part unspecified |
| **B825700** | Carcinoma in situ of skin of buttock |
| **B325300** | Malignant melanoma of groin |
| **BB5D100** | [M]Cholangiocarcinoma |
| **B161z00** | Malignant neoplasm of extrahepatic bile ducts NOS |
| **B310000** | Malignant neoplasm of soft tissue of head |
| **B43..00** | Malignant neoplasm of body of uterus |
| **BBVz.00** | [M]Osteoma or osteosarcoma NOS |
| **B41..00** | Malignant neoplasm of cervix uteri |
| **BB81200** | [M]Serous cystadenocarcinoma, NOS |
| **B827.00** | Carcinoma in situ of skin of lower limb and hip |
| **B062300** | Malignant neoplasm of palatopharyngeal arch |
| **B810811** | Carcinoma in situ of glottis |
| **B454.11** | Primary vulval cancer |
| **B911013** | Choriocarcinoma |
| **BBK0400** | [M]Epithelioid leiomyosarcoma |
| **BBV2.00** | [M]Chondroblastic osteosarcoma |
| **B220z00** | Malignant neoplasm of trachea NOS |
| **BB2C.00** | [M]Squamous cell carcinoma, keratinising type NOS |
| **BB60.00** | [M]Skin appendage adenoma and carcinoma |
| **4M3..00** | Breslow depth staging for melanoma |
| **B4A2.00** | Malignant neoplasm of ureter |
| **BB01.00** | [M]Neoplasm, uncertain whether benign or malignant |
| **BBB6100** | [M]Thymoma, malignant |
| **BBR4.00** | [M]Malignant teratoma, trophoblastic |
| **B335300** | Malignant neoplasm of skin of abdominal wall |
| **B550400** | Malignant neoplasm of neck NOS |
| **BB5B600** | [M]Mixed islet cell and exocrine adenocarcinoma |
| **ZV10017** | [V]Personal history of malignant neoplasm of rectum |
| **B81z.00** | Carcinoma in situ of respiratory organ NOS |
| **B213100** | Malignant neoplasm of cricoid cartilage |
| **B551.00** | Malignant neoplasm of thorax |
| **B334100** | Malignant neoplasm of skin of neck |
| **B810.00** | Carcinoma in situ of larynx |
| **BB2C.11** | [M]Epidermoid carcinoma, keratinising type |
| **B141.00** | Malignant neoplasm of rectum |
| **B202.00** | Malignant neoplasm of maxillary sinus |
| **B333000** | Malignant neoplasm of skin of cheek, external |
| **Byu5300** | [X]Kaposi's sarcoma, unspecified |
| **BB5D300** | [M]Bile duct cystadenocarcinoma |
| **B803200** | Carcinoma in situ of descending colon |
| **B808400** | Carcinoma in situ of cystic duct |
| **B302000** | Malignant neoplasm of cervical vertebra |
| **BB9B.00** | [M]Medullary carcinoma NOS |
| **B517.00** | Malignant neoplasm of brain stem |
| **B553000** | Malignant neoplasm of inguinal region NOS |
| **B18y500** | Malignant neoplasm of pelvic peritoneum |
| **F396200** | Myopathy due to malignant disease |
| **B308100** | Malignant neoplasm of talus |
| **B304100** | Malignant neoplasm of acromion |
| **ZV10414** | [V]Personal history of malignant neoplasm of ovary |
| **BBEV.00** | [M]Blue naevus, malignant |
| **ZV10500** | [V]Personal history of malignant neoplasm of urinary organ |
| **B821.00** | Carcinoma in situ of skin of eyelid including canthus |
| **B05y.00** | Malignant neoplasm of other specified mouth parts |
| **B828100** | Melanoma in situ of eyelid, including canthus |
| **B332000** | Malignant neoplasm of skin of auricle (ear) |
| **BBLC100** | [M]Mesenchymoma, malignant |
| **BB80z00** | [M]Cystadenoma or carcinoma NOS |
| **B067.00** | Malignant neoplasm of posterior wall of oropharynx |
| **B55..00** | Malignant neoplasm of other and ill-defined sites |
| **B831.00** | Carcinoma in situ of cervix uteri |
| **B305D00** | Malignant neoplasm of phalanges of hand |
| **B81y.11** | Carcinoma in situ of nasal sinuses |
| **B450100** | Malignant neoplasm of vaginal vault |
| **4K2M.00** | Crv smr - hi grade dyskaryosis? invasive squamous carcinoma |
| **B221.00** | Malignant neoplasm of main bronchus |
| **ByuD400** | [X]Other malignant immunoproliferative diseases |
| **B321.00** | Malignant melanoma of eyelid including canthus |
| **B80z100** | Carcinoma in situ of spleen |
| **B593.00** | Primary malignant neoplasm of unknown site |
| **B825z00** | Carcinoma in situ of skin of trunk NOS |
| **B050.00** | Malignant neoplasm of cheek mucosa |
| **B003000** | Malignant neoplasm of lower lip, buccal aspect |
| **B11z.00** | Malignant neoplasm of stomach NOS |
| **BBEG.11** | [M]Lentigo maligna melanoma |
| **B542000** | Malignant neoplasm of pituitary gland |
| **B300B00** | Malignant neoplasm of turbinate |
| **B001.00** | Malignant neoplasm of lower lip, vermilion border |
| **BB52.00** | [M]Adenocarcinoma NOS |
| **BBLJ.00** | [M]Clear cell sarcoma of kidney |
| **B51..00** | Malignant neoplasm of brain |
| **B812000** | Carcinoma in situ of carina of bronchus |
| **B18yz00** | Malignant neoplasm of specified parts of peritoneum NOS |
| **B5...11** | Carcinoma of other and unspecified sites |
| **B510400** | Malignant neoplasm of hypothalamus |
| **B337.00** | Malignant neoplasm of skin of lower limb and hip |
| **B828600** | Melanoma in situ of lower limb, including hip |
| **ZV10015** | [V]Personal history of malignant neoplasm of liver |
| **BB5V711** | [M]Mucoid cell carcinoma |
| **B331100** | Malignant neoplasm of upper eyelid |
| **BBM0100** | [M]Brenner tumour, malignant |
| **BB5Cz00** | [M]Gastrinoma or carcinoma NOS |
| **ZV10212** | [V]Personal history of malignant neoplasm of larynx |
| **BB71.00** | [M]Mucoepidermoid carcinoma |
| **B911000** | Malignant hydatidiform mole |
| **44a4.00** | Squamous cell carcinoma antigen level |
| **Byu7000** | [X]Malignant neoplasm of uterine adnexa, unspecified |
| **B214.00** | Malignant neoplasm, overlapping lesion of larynx |
| **Byu2000** | [X]Malignant neoplasm of bronchus or lung, unspecified |
| **B143.00** | Malignant neoplasm of anus unspecified |
| **BB3G.00** | [M]Pigmented basal cell carcinoma |
| **B325500** | Malignant melanoma of perineum |
| **BB82z00** | [M]Mucinous adenoma or adenocarcinoma NOS |
| **B110000** | Malignant neoplasm of cardiac orifice of stomach |
| **B804.00** | Carcinoma in situ of rectum and rectosigmoid junction |
| **B515.00** | Malignant neoplasm of cerebral ventricles |
| **B807300** | Carcinoma in situ of Meckel's diverticulum |
| **B836000** | Carcinoma in situ of testis |
| **BB91.11** | [M]Duct carcinoma NOS |
| **B515000** | Malignant neoplasm of choroid plexus |
| **B060.00** | Malignant neoplasm of tonsil |
| **B35..00** | Malignant neoplasm of male breast |
| **B800200** | Carcinoma in situ of salivary glands |
| **B013z00** | Malignant neoplasm of ventral tongue surface NOS |
| **B327.00** | Malignant melanoma of lower limb and hip |
| **B02..00** | Malignant neoplasm of major salivary glands |
| **B335700** | Malignant neoplasm of skin of back |
| **B430.00** | Malignant neoplasm of corpus uteri, excluding isthmus |
| **B060100** | Malignant neoplasm of palatine tonsil |
| **B215.00** | Malignant neoplasm of epiglottis NOS |
| **BBW8.00** | [M]Chondroblastoma, malignant |
| **B324.00** | Malignant melanoma of scalp and neck |
| **BB3C.00** | [M]Superficial basal cell carcinoma |
| **BBV..12** | [M]Parosteal osteosarcoma |
| **A788W00** | HIV disease resulting in unspecified malignant neoplasm |
| **B124.00** | Malignant neoplasm, overlapping lesion of small intestine |
| **BB5S211** | [M]Alveolar cell carcinoma |
| **B496.00** | Malignant neoplasm of ureteric orifice |
| **B6...00** | Malignant neoplasm of lymphatic and haemopoietic tissue |
| **ByuE.00** | [X]Malignant neoplasms/independent (primary) multiple sites |
| **B827100** | Carcinoma in situ of skin of thigh |
| **B326.00** | Malignant melanoma of upper limb and shoulder |
| **B303300** | Malignant neoplasm of costal cartilage |
| **B8yy200** | Carcinoma in situ of parathyroid gland |
| **BBQ7400** | [M]Malignant teratoma, undifferentiated type |
| **BB1M.00** | [M]Small cell carcinoma, intermediate cell |
| **B017.00** | Malignant overlapping lesion of tongue |
| **BB57.00** | [M]Adenocarcinoma, intestinal type |
| **ByuA300** | [X]Malig neopl, overlap lesion brain & other part of CNS |
| **ZV67B00** | [V]Folow-up exam aft unspec treatment for malignant neoplasm |
| **B34z.00** | Malignant neoplasm of female breast NOS |
| **B00z000** | Malignant neoplasm of lip, unspecified, external |
| **BB5W112** | [M]Oncytic adenocarcinoma |
| **BB42.00** | [M]Transitional cell carcinoma in situ |
| **B150.00** | Primary malignant neoplasm of liver |
| **B322000** | Malignant melanoma of auricle (ear) |
| **B827400** | Carcinoma in situ of skin of foot |
| **BBK3200** | [M]Pleomorphic rhabdomyosarcoma |
| **B22y.00** | Malignant neoplasm of other sites of bronchus or lung |
| **BBFz.00** | [M]Soft tissue tumour or sarcoma NOS |
| **ByuB100** | [X]Malignant neoplasm of endocrine gland, unspecified |
| **B000000** | Malignant neoplasm of upper lip, external |
| **BB32.00** | [M]Multicentric basal cell carcinoma |
| **B150000** | Primary carcinoma of liver |
| **ByuF900** | [X]Carcinoma in situ of skin, unspecified |
| **B102.00** | Malignant neoplasm of abdominal oesophagus |
| **B300400** | Malignant neoplasm of occipital bone |
| **B501z00** | Malignant neoplasm of orbit NOS |
| **B141.12** | Rectal carcinoma |
| **BBW9.00** | [M]Mesenchymal chondrosarcoma |
| **BBV1.13** | [M]Osteogenic sarcoma NOS |
| **ZV10y12** | [V]Personal history of malignant neoplasm of brain |
| **B550000** | Malignant neoplasm of head NOS |
| **B510z00** | Malignant neoplasm of cerebrum NOS |
| **B180200** | Malignant neoplasm of retrocaecal tissue |
| **BBQ4.11** | [M]Infantile embryonal carcinoma |
| **BB5V700** | [M]Basophil carcinoma |
| **B802300** | Carcinoma in situ of pyloric antrum |
| **B485.00** | Malignant neoplasm of spermatic cord |
| **B322100** | Malignant melanoma of external auditory meatus |
| **B1...00** | Malignant neoplasm of digestive organs and peritoneum |
| **BB08.00** | [M]Malignant tumour, small cell type |
| **BBTD200** | [M]Haemangiopericytoma, malignant |
| **B524600** | Malignant neoplasm,overlap lesion periph nerve & auton ns |
| **B41y.00** | Malignant neoplasm of other site of cervix |
| **B81..00** | Carcinoma in situ of respiratory system |
| **B18..00** | Malignant neoplasm of retroperitoneum and peritoneum |
| **B327500** | Malignant melanoma of ankle |
| **B21z.00** | Malignant neoplasm of larynx NOS |
| **BBQ9.00** | [M]Dermoid cyst with malignant transformation |
| **ZV10417** | [V]Personal history of malignant neoplasm of uterine body |
| **B524300** | Malignant neoplasm of peripheral nerve of thorax |
| **B551000** | Malignant neoplasm of axilla NOS |
| **B307200** | Malignant neoplasm of tibia |
| **B82..00** | Carcinoma in situ of skin |
| **B004200** | Malignant neoplasm of lip unspecified, mucosa |
| **ByuFC00** | [X]Carcinoma in situ of oth+unspecified male genital organs |
| **B307100** | Malignant neoplasm of fibula |
| **B200.00** | Malignant neoplasm of nasal cavities |
| **BB5J.11** | [M]Cylindroid adenocarcinoma |
| **B825400** | Carcinoma in situ of skin of abdominal wall |
| **BBCA.00** | [M]Sertoli cell carcinoma |
| **B330.00** | Malignant neoplasm of skin of lip |
| **B802200** | Carcinoma in situ of body of stomach |
| **B45..00** | Malig neop of other and unspecified female genital organs |
| **B315200** | Malignant neoplasm of connective and soft tissue of perineum |
| **BBQ7500** | [M]Malignant teratoma, intermediate type |
| **B136.00** | Malignant neoplasm of ascending colon |
| **BBB7.00** | [M]Epithelial-myoepithelial carcinoma |
| **BBY0.00** | [M]Ewing's sarcoma |
| **B30W.00** | Malignant neoplasm/overlap lesion/bone+articulr cartilage |
| **B333400** | Malignant neoplasm of skin of nose (external) |
| **B213300** | Malignant neoplasm of thyroid cartilage |
| **BB62.00** | [M]Apocrine adenoma and adenocarcinomas |
| **BB5L300** | [M]Adenocarcinoma in multiple adenomatous polyps |
| **B224.00** | Malignant neoplasm of lower lobe, bronchus or lung |
| **B30..00** | Malignant neoplasm of bone and articular cartilage |
| **B54z.00** | Malig neop of endocrine gland or related structure NOS |
| **B101.00** | Malignant neoplasm of thoracic oesophagus |
| **B497.00** | Malignant neoplasm of urachus |
| **B333z00** | Malignant neoplasm skin other and unspec part of face NOS |
| **BBe7.11** | [M]Schwannoma, malignant |
| **B30z000** | Osteosarcoma |
| **BBDB.00** | [M]Glomangiosarcoma |
| **BBV9.00** | [M]Myxoid chondrosarcoma |
| **B828900** | Melanoma in situ of back |
| **B12z.00** | Malignant neoplasm of small intestine NOS |
| **Byu2400** | [X]Malignant neoplasm/ill-defined sites within resp system |
| **B336z00** | Malignant neoplasm of skin of upper limb or shoulder NOS |
| **B071.00** | Malignant neoplasm of posterior wall of nasopharynx |
| **B241000** | Malignant neoplasm of endocardium |
| **B030.00** | Malignant neoplasm of upper gum |
| **B300500** | Malignant neoplasm of orbital bone |
| **B506.00** | Malignant neoplasm of choroid |
| **B828W00** | Melanoma in situ, unspecified |
| **BB69.00** | [M]Sebaceous adenoma and adenocarcinoma |
| **BB1J.00** | [M]Small cell carcinoma NOS |
| **B832.11** | Carcinoma in situ of body of uterus |
| **B808200** | Carcinoma in situ of hepatic duct |
| **B810300** | Carcinoma in situ of arytenoid cartilage |
| **B323500** | Malignant melanoma of temple |
| **B308200** | Malignant neoplasm of calcaneum |
| **B835.00** | Carcinoma in situ of penis |
| **B335400** | Malignant neoplasm of skin of umbilicus |
| **B808300** | Carcinoma in situ of gall bladder |
| **B055z00** | Malignant neoplasm of palate NOS |
| **B300600** | Malignant neoplasm of parietal bone |
| **B486.00** | Malignant neoplasm of scrotum |
| **B553100** | Malignant neoplasm of presacral region |
| **B51y200** | Malignant neoplasm, overlapping lesion of brain |
| **BBb0.00** | [M]Glioma, malignant |
| **B206.00** | Malignant neoplasm, overlapping lesion of accessory sinuses |
| **B810200** | Carcinoma in situ of epiglottis |
| **BBgJ.11** | [M]Germinoblastic sarcoma NOS |
| **B520000** | Malignant neoplasm of olfactory bulb |
| **BB5z.00** | [M]Adenoma or adenocarcinoma NOS |
| **B517300** | Malignant neoplasm of pons |
| **B17..00** | Malignant neoplasm of pancreas |
| **B451000** | Malignant neoplasm of greater vestibular (Bartholin's) gland |
| **BB5Nz00** | [M]Adenomatous or adenocarcinomatous polyps of the colon NOS |
| **4M73.00** | Clark melanoma level 4 |
| **B004000** | Malignant neoplasm of lip unspecified, buccal aspect |
| **B006.00** | Malignant neoplasm of overlapping lesion of lip |
| **B224100** | Malignant neoplasm of lower lobe of lung |
| **B337600** | Malignant neoplasm of skin of heel |
| **B304200** | Malignant neoplasm of humerus |
| **B452.00** | Malignant neoplasm of labia minora |
| **B326400** | Malignant melanoma of finger |
| **B830.00** | Carcinoma in situ of breast |
| **B308z00** | Malignant neoplasm of short bones of leg NOS |
| **BB81800** | [M]Papillary serous cystadenocarcinoma |
| **BBLD.00** | [M]Embryonal sarcoma |
| **ByuA.00** | [X]Malignant neoplasm of eye, brain and other parts of cent |
| **B17z.00** | Malignant neoplasm of pancreas NOS |
| **B241.00** | Malignant neoplasm of heart |
| **B551200** | Malignant neoplasm of intrathoracic site NOS |
| **B306.00** | Malignant neoplasm of pelvic bones, sacrum and coccyx |
| **B332100** | Malignant neoplasm of skin of external auditory meatus |
| **B410.00** | Malignant neoplasm of endocervix |
| **B451.00** | Malignant neoplasm of labia majora |
| **B833200** | Carcinoma in situ of vagina |
| **B08..00** | Malignant neoplasm of hypopharynx |
| **B800800** | Carcinoma in situ of oropharynx |
| **B482.00** | Malignant neoplasm of body of penis |
| **B141.11** | Carcinoma of rectum |
| **B51y.00** | Malignant neoplasm of other parts of brain |
| **B801100** | Carcinoma in situ of middle 1/3 oesophagus |
| **BBK0200** | [M]Leiomyosarcoma NOS |
| **B410100** | Malignant neoplasm of endocervical gland |
| **B520100** | Malignant neoplasm of optic nerve |
| **B01y.00** | Malignant neoplasm of other sites of tongue |
| **BBK3600** | [M]Embryonal rhabdomyosarcoma |
| **B800.11** | Carcinoma in situ of oral cavity |
| **B825800** | Carcinoma in situ of perianal skin |
| **B487.00** | Malignant neoplasm, overlapping lesion of penis |
| **B07..00** | Malignant neoplasm of nasopharynx |
| **BBD 1.00** | [M]Paraganglioma, malignant |
| **B55y200** | Malignant neoplasm of flank NOS |
| **B50z.00** | Malignant neoplasm of eye NOS |
| **B200z00** | Malignant neoplasm of nasal cavities NOS |
| **BBV4.00** | [M]Telangiectatic osteosarcoma |
| **BBET.00** | [M]Mixed epithelioid and spindle melanoma |
| **BB9F.00** | [M]Lobular carcinoma NOS |
| **B523.00** | Malignant neoplasm of spinal meninges |
| **BB62100** | [M]Apocrine adenocarcinoma |
| **B061.00** | Malignant neoplasm of tonsillar fossa |
| **B55y.00** | Malignant neoplasm of other specified sites |
| **B020.00** | Malignant neoplasm of parotid gland |
| **BBG3.00** | [M]Fibromyxosarcoma |
| **B45y.00** | Malignant neoplasm of other specified female genital organ |
| **B324100** | Malignant melanoma of neck |
| **B41y100** | Malignant neoplasm of squamocolumnar junction of cervix |
| **BBK2100** | [M]Myosarcoma |
| **B49z.00** | Malignant neoplasm of urinary bladder NOS |
| **B336400** | Malignant neoplasm of skin of finger |
| **BBJ3.00** | [M]Liposarcoma, well differentiated type |
| **BBZN.11** | [M]Odontogenic fibrosarcoma |
| **B110.00** | Malignant neoplasm of cardia of stomach |
| **B52W.00** | Malig neopl, overlap lesion brain & other part of CNS |
| **B828.00** | Melanoma in situ of skin |
| **B056.00** | Malignant neoplasm of retromolar area |
| **BB91.00** | [M]Infiltrating duct carcinoma |
| **B138.00** | Malignant neoplasm, overlapping lesion of colon |
| **B11y100** | Malignant neoplasm of posterior wall of stomach NEC |
| **BB5..00** | [M]Adenomas and adenocarcinomas |
| **BBF4.11** | [M]Pleomorphic cell sarcoma |
| **B013100** | Malignant neoplasm of frenulum linguae |
| **BB5f600** | [M]Papillary and follicular adenocarcinoma |
| **B4A4.00** | Malignant neoplasm of paraurethral glands |
| **4M72.00** | Clark melanoma level 3 |
| **ZV10112** | [V]Personal history of malignant neoplasm of lung |
| **BB62z00** | [M]Apocrine adenoma or adenocarcinoma NOS |
| **BB43.00** | [M]Transitional cell carcinoma NOS |
| **B300900** | Malignant neoplasm of zygomatic bone |
| **B12y.00** | Malignant neoplasm of other specified site small intestine |
| **B100.00** | Malignant neoplasm of cervical oesophagus |
| **B010.00** | Malignant neoplasm of base of tongue |
| **BB81500** | [M]Papillary cystadenocarcinoma, NOS |
| **B18y200** | Malignant neoplasm of mesorectum |
| **Byu2500** | [X]Malignant neoplasm of mediastinum, part unspecified |
| **ByuC800** | [X]Malignant neoplasm without specification of site |
| **B14..00** | Malignant neoplasm of rectum, rectosigmoid junction and anus |
| **B432.00** | Malignant neoplasm of overlapping lesion of corpus uteri |
| **B55y100** | Malignant neoplasm of trunk NOS |
| **B110100** | Malignant neoplasm of cardio-oesophageal junction of stomach |
| **BB81H00** | [M]Papillary mucinous cystadenocarcinoma |
| **BB91100** | [M]Infiltrating duct and lobular carcinoma |
| **BBGM.00** | [M]Dermatofibrosarcoma NOS |
| **BBJ5.00** | [M]Myxoid liposarcoma |
| **B6y..00** | Malignant neoplasm lymphatic or haematopoietic tissue OS |
| **BBH1.00** | [M]Myxosarcoma |
| **BBR2.00** | [M]Choriocarcinoma |
| **B81yz00** | Carcinoma in situ of specified parts respiratory system NOS |
| **B550z00** | Malignant neoplasm of head, neck and face NOS |
| **B812.00** | Carcinoma in situ of bronchus and lung |
| **B40..00** | Malignant neoplasm of uterus, part unspecified |
| **B00z100** | Malignant neoplasm of lip, unspecified, lipstick area |
| **BB5fz00** | [M]Thyroid adenoma or adenocarcinoma NOS |
| **B824100** | Carcinoma in situ of skin of neck |
| **B430000** | Malignant neoplasm of cornu of corpus uteri |
| **BBE4.00** | [M]Balloon cell melanoma |
| **BB6A.00** | [M]Ceruminous adenoma and adenocarcinoma |
| **BB5Xz00** | [M]Clear cell adenoma or adenocarcinoma NOS |
| **BB22.00** | [M]Papillary carcinoma NOS |
| **B47..00** | Malignant neoplasm of testis |
| **BB5K.00** | [M]Cribriform carcinoma |
| **B524500** | Malignant neoplasm of peripheral nerve of pelvis |
| **B305.12** | Malignant neoplasm of metacarpal bones |
| **Byu2100** | [X]Malignant neoplasm/overlap lesion/heart,mediastinm+pleura |
| **BBZG.00** | [M]Ameloblastoma, malignant |
| **BBDA.00** | [M]Phaeochromocytoma, malignant |
| **Byu7.00** | [X]Malignant neoplasm of female genital organs |
| **B44..00** | Malignant neoplasm of ovary and other uterine adnexa |
| **B542z00** | Malig neop pituitary gland or craniopharyngeal duct NOS |
| **B171.00** | Malignant neoplasm of body of pancreas |
| **BB5C.00** | [M]Gastrinoma and carcinomas |
| **B200200** | Malignant neoplasm of septum of nose |
| **B132.00** | Malignant neoplasm of descending colon |
| **B310200** | Malignant neoplasm of soft tissue of neck |
| **BB2M.00** | [M]Lymphoepithelial carcinoma |
| **B064100** | Malignant neoplasm of glossoepiglottic fold |
| **B44y.00** | Malignant neoplasm of other site of uterine adnexa |
| **B80z.00** | Carcinoma in situ of other and unspecified digestive organs |
| **BB2A.13** | [M]Squamous cell carcinoma of skin NOS |
| **BB54.00** | [M]Scirrhous adenocarcinoma |
| **B4A3.00** | Malignant neoplasm of urethra |
| **BB47.00** | [M]Transitional cell carcinoma, spindle cell type |
| **B828200** | Melanoma in situ of ear and external auricular canal |
| **B807.00** | Carcinoma in situ of other and unspecified small intestine |
| **7G03K00** | Excision malignant skin tumour |
| **B804100** | Carcinoma in situ of rectum |
| **7G03J00** | Excision of melanoma |
| **B80..00** | Carcinoma in situ of digestive organs |
| **ByuA100** | [X]Malignant neoplasm/central nervous system, unspecified |
| **ByuA200** | [X]Malignant neoplasm of meninges, unspecified |
| **B00zz00** | Malignant neoplasm of lip, vermilion border NOS |
| **B03..00** | Malignant neoplasm of gum |
| **Byu2300** | [X]Malignant neopl/overlapping les/resp+intrathoracic organs |
| **B161000** | Malignant neoplasm of cystic duct |
| **BB5L100** | [M]Adenocarcinoma in adenomatous polyp |
| **BB5f111** | [M]Follicular carcinoma |
| **BB11.00** | [M]Carcinoma in situ NOS |
| **BB5Uz00** | [M]Villous adenoma or adenocarcinoma NOS |
| **B062z00** | Malignant neoplasm of tonsillar fossa NOS |
| **B822.00** | Carcinoma in situ skin of ear and external auricular canal |
| **BB5R500** | [M]Carcinoid tumour, nonargentaffin, malignant |
| **BBT7100** | [M]Haemangioendothelioma, malignant |
| **B822.11** | Carcinoma in situ of ear |
| **B2...11** | Carcinoma of respiratory tract and intrathoracic organs |
| **B553200** | Malignant neoplasm of sacrococcygeal region |
| **B04..00** | Malignant neoplasm of floor of mouth |
| **B810600** | Carcinoma in situ of aryepiglottic fold |
| **BBB2.00** | [M]Adenocarcinoma with squamous metaplasia |
| **B553z00** | Malignant neoplasm of pelvis NOS |
| **B443.00** | Malignant neoplasm of parametrium |
| **B653100** | Granulocytic sarcoma |
| **B20z.00** | Malignant neoplasm of accessory sinus NOS |
| **B222100** | Malignant neoplasm of upper lobe of lung |
| **B540100** | Malignant neoplasm of adrenal medulla |
| **B55y000** | Malignant neoplasm of back NOS |
| **B003100** | Malignant neoplasm of lower lip, frenulum |
| **B830000** | Lobular carcinoma in situ of breast |
| **B501.00** | Malignant neoplasm of orbit |
| **B335100** | Malignant neoplasm of skin of chest, excluding breast |
| **BB24.00** | [M]Verrucous carcinoma NOS |
| **B051100** | Malignant neoplasm of lower buccal sulcus |
| **F373.00** | Polyneuropathy in malignant disease |
| **B805.00** | Carcinoma in situ of anal canal |
| **BB5V.00** | [M]Pituitary adenomas and carcinomas |
| **BB5..11** | [M]Adenocarcinomas |
| **B431.00** | Malignant neoplasm of isthmus of uterine body |
| **B504.00** | Malignant neoplasm of cornea |
| **B524400** | Malignant neoplasm of peripheral nerve of abdomen |
| **B306300** | Malignant neoplasm of sacral vertebra |
| **BB5D700** | [M]Combined hepatocellular carcinoma and cholangiocarcinoma |
| **B307.00** | Malignant neoplasm of long bones of leg |
| **B23z.00** | Malignant neoplasm of pleura NOS |
| **B08z.00** | Malignant neoplasm of hypopharynx NOS |
| **B042.00** | Malignant neoplasm, overlapping lesion of floor of mouth |
| **B832000** | Carcinoma in situ of endometrium |
| **B811.00** | Carcinoma in situ of trachea |
| **BB85.00** | [M]Signet ring carcinoma |
| **ZV10y00** | [V]Personal history of other specified malignant neoplasm |
| **B1zz.00** | Malignant neoplasm of digestive tract and peritoneum NOS |
| **B336100** | Malignant neoplasm of skin of upper arm |
| **B01..00** | Malignant neoplasm of tongue |
| **B4Ay.00** | Malignant neoplasm of other urinary organs |
| **Byu4000** | [X]Malignant melanoma of other+unspecified parts of face |
| **B306000** | Malignant neoplasm of ilium |
| **B220.00** | Malignant neoplasm of trachea |
| **B333200** | Malignant neoplasm of skin of eyebrow |
| **BB82111** | [M]Colloid adenocarcinoma |
| **BB5f100** | [M]Follicular adenocarcinoma NOS |
| **B831.11** | CIN III - carcinoma in situ of cervix |
| **B322.00** | Malignant melanoma of ear and external auricular canal |
| **B2zz.00** | Malignant neoplasm of respiratory tract NOS |
| **BBP7.00** | [M]Mesothelioma, biphasic type, malignant |
| **B23..00** | Malignant neoplasm of pleura |
| **B507100** | Malignant neoplasm of nasolacrimal duct |
| **B495.00** | Malignant neoplasm of bladder neck |
| **B134.00** | Malignant neoplasm of caecum |
| **B836.00** | Carcinoma in situ other and unspecified male genital organs |
| **B201300** | Malignant neoplasm of mastoid air cells |
| **BBEX.00** | [M]Melanoma in situ |
| **B04y.00** | Malignant neoplasm of other sites of floor of mouth |
| **B54..00** | Malig neop of other endocrine glands and related structures |
| **B3z..00** | Malig neop of bone, connective tissue, skin and breast NOS |
| **B45y000** | Malignant neoplasm of overlapping lesion of vulva |
| **B06yz00** | Malignant neoplasm of other specified site of oropharynx NOS |
| **B000.00** | Malignant neoplasm of upper lip, vermilion border |
| **B4y..00** | Malignant neoplasm of genitourinary organ OS |
| **B31..00** | Malignant neoplasm of connective and other soft tissue |
| **B1z1000** | Angiosarcoma of spleen |
| **B323z00** | Malignant melanoma of face NOS |
| **B336500** | Malignant neoplasm of skin of thumb |
| **B340000** | Malignant neoplasm of nipple of female breast |
| **B016.00** | Malignant neoplasm of lingual tonsil |
| **B308D00** | Malignant neoplasm of phalanges of foot |
| **B822000** | Carcinoma in situ of skin of auricle |
| **B344.00** | Malignant neoplasm of upper-outer quadrant of female breast |
| **B327700** | Malignant melanoma of foot |
| **B26..00** | Malignant neoplasm, overlap lesion of resp & intrathor orgs |
| **B824000** | Carcinoma in situ of scalp |
| **B833300** | Carcinoma in situ of vulva |
| **B41..11** | Cervical carcinoma (uterus) |
| **B305z00** | Malignant neoplasm of hand bones NOS |
| **B335900** | Malignant neoplasm of perianal skin |
| **B337700** | Malignant neoplasm of skin of foot |
| **B512.00** | Malignant neoplasm of temporal lobe |
| **BB5F.00** | [M]Trabecular adenocarcinoma |
| **BBLA.00** | [M]Carcinosarcoma, embryonal type |
| **B521.00** | Malignant neoplasm of cerebral meninges |
| **BB31.00** | [M]Basal cell carcinoma NOS |
| **B500.00** | Malig neop eyeball excl conjunctiva, cornea, retina, choroid |
| **B510500** | Malignant neoplasm of thalamus |
| **B335z00** | Malignant neoplasm of skin of trunk, excluding scrotum, NOS |
| **B8yy000** | Carcinoma in situ of thyroid gland |
| **BBEQ.00** | [M]Spindle cell melanoma NOS |
| **B820.00** | Carcinoma in situ of skin of lip |
| **B803z00** | Carcinoma in situ of colon NOS |
| **B339.00** | Dermatofibrosarcoma protuberans |
| **B347.00** | Malignant neoplasm, overlapping lesion of breast |
| **B073100** | Malignant neoplasm of nasopharyngeal soft palate surface |
| **BB9B.11** | [M]C cell carcinoma |
| **B825600** | Carcinoma in situ of skin of perineum |
| **B492.00** | Malignant neoplasm of lateral wall of urinary bladder |
| **B111000** | Malignant neoplasm of prepylorus of stomach |
| **B02y.00** | Malignant neoplasm of other major salivary glands |
| **B066.00** | Malignant neoplasm of lateral wall of oropharynx |
| **B592.00** | Malignant neoplasms of independent (primary) multiple sites |
| **BB1B.00** | [M]Giant cell and spindle cell carcinoma |
| **Byu7300** | [X]Malignant neoplasm of female genital organ, unspecified |
| **B06y.00** | Malignant neoplasm of oropharynx, other specified sites |
| **B507.00** | Malignant neoplasm of lacrimal duct |
| **B010000** | Malignant neoplasm of base of tongue dorsal surface |
| **B48y000** | Malignant neoplasm of seminal vesicle |
| **B5...00** | Malignant neoplasm of other and unspecified sites |
| **B48z.00** | Malignant neoplasm of penis and other male genital organ NOS |
| **BBK3100** | [M]Rhabdomyosarcoma NOS |
| **B520z00** | Malignant neoplasm of cranial nerves NOS |
| **B595.00** | Malignant tumour of unknown origin |
| **BB48.00** | [M]Basaloid carcinoma |
| **B41z.00** | Malignant neoplasm of cervix uteri NOS |
| **B313.00** | Malignant neoplasm of connective and soft tissue of thorax |
| **B107.00** | Siewert type I adenocarcinoma |
| **BB12.00** | [M]Carcinoma NOS |
| **BBP1.00** | [M]Mesothelioma, malignant |
| **B41y000** | Malignant neoplasm of cervical stump |
| **BBA2.00** | [M]Acinar cell carcinoma |
| **B160.00** | Malignant neoplasm of gallbladder |
| **B160.11** | Carcinoma gallbladder |
| **Byu3100** | [X]Malignant neoplasm/bones+articular cartilage/limb,unspfd |
| **BB9K000** | [M]Paget's disease and intraductal carcinoma of breast |
| **BB1E.00** | [M]Pseudosarcomatous carcinoma |
| **ZV10018** | [V]Personal history of malignant neoplasm of stomach |
| **B1z0.00** | Malignant neoplasm of intestinal tract, part unspecified |
| **BBEG000** | [M]Acral lentiginous melanoma, malignant |
| **BB4..00** | [M]Transitional cell papillomas and carcinomas |
| **B525.00** | Malignant neoplasm of cauda equina |
| **B510000** | Malignant neoplasm of basal ganglia |
| **BB93.00** | [M]Comedocarcinoma NOS |
| **Byu1300** | [X]Malignant neoplsm/ill-defin sites within digestive system |
| **B083.00** | Malignant neoplasm of posterior pharynx |
| **Byu4200** | [X]Oth malignant neoplasm/skin of oth+unspecfd parts of face |
| **B52z.00** | Malignant neoplasm of nervous system NOS |
| **Byu3300** | [X]Malignant neoplasm/bone+articular cartilage, unspecified |
| **B07z.00** | Malignant neoplasm of nasopharynx NOS |
| **B803400** | Carcinoma in situ of caecum |
| **BB5U100** | [M]Adenocarcinoma in villous adenoma |
| **B52y.00** | Malignant neoplasm of other specified part of nervous system |
| **BB5L200** | [M]Adenocarcinoma in situ in adenomatous polyp |
| **B11..00** | Malignant neoplasm of stomach |
| **B51z.00** | Malignant neoplasm of brain NOS |
| **B322z00** | Malignant melanoma of ear and external auricular canal NOS |
| **ZV10y11** | [V]Personal history of malignant neoplasm of bone |
| **B493.00** | Malignant neoplasm of anterior wall of urinary bladder |
| **4D56.00** | Pleural fluid: malignant cells |
| **B111100** | Malignant neoplasm of pyloric canal of stomach |
| **B470z00** | Malignant neoplasm of undescended testis NOS |
| **B003.00** | Malignant neoplasm of lower lip, inner aspect |
| **B315000** | Malignant neoplasm of connective and soft tissue of buttock |
| **B041.00** | Malignant neoplasm of lateral portion of floor of mouth |
| **BBEA.00** | [M]Amelanotic melanoma |
| **B507000** | Malignant neoplasm of lacrimal sac |
| **B022.00** | Malignant neoplasm of sublingual gland |
| **BB5X.00** | [M]Clear cell adenomas and adenocarcinomas |
| **B13y.00** | Malignant neoplasm of other specified sites of colon |
| **B306400** | Malignant neoplasm of coccygeal vertebra |
| **B311400** | Malignant neoplasm of connective and soft tissue of finger |
| **B431000** | Malignant neoplasm of lower uterine segment |
| **H51y700** | Malignant pleural effusion |
| **B337000** | Malignant neoplasm of skin of hip |
| **BB33.00** | [M]Basal cell carcinoma, morphoea type |
| **B303z00** | Malignant neoplasm of rib, sternum and clavicle NOS |
| **B001100** | Malignant neoplasm of lower lip, lipstick area |
| **B350100** | Malignant neoplasm of areola of male breast |
| **B18y300** | Malignant neoplasm of omentum |
| **B161300** | Malignant neoplasm of sphincter of Oddi |
| **ByuD.00** | [X]Malignant neoplasms of lymphoid, haematopoietic and rela |
| **B200000** | Malignant neoplasm of cartilage of nose |
| **ZV10y16** | [V]Personal history of malignant neoplasm of tongue |
| **BBDB.11** | [M]Glomoid sarcoma |
| **B071100** | Malignant neoplasm of pharyngeal tonsil |
| **BB5Mz00** | [M]Tubular adenoma or adenocarcinoma NOS |
| **BBLE.00** | [M]Adenosarcoma |
| **B211.00** | Malignant neoplasm of supraglottis |
| **B18z.00** | Malignant neoplasm of retroperitoneum and peritoneum NOS |
| **BB52000** | [M]Adenocarcinoma in tubulovillous adenoma |
| **B140.00** | Malignant neoplasm of rectosigmoid junction |
| **BBL8.00** | [M]Hepatoblastoma |
| **B326000** | Malignant melanoma of shoulder |
| **B326300** | Malignant melanoma of hand |
| **BB29.11** | [M]Epidermoid carcinoma in situ |
| **ByuFA00** | [X]Carcinoma in situ of other parts of cervix |
| **BBX1.11** | [M]Giant cell bone sarcoma |
| **B121.00** | Malignant neoplasm of jejunum |
| **BB5y000** | [M]Basal cell adenocarcinoma |
| **B00..11** | Carcinoma of lip |
| **Byu4300** | [X]Malignant neoplasm of skin, unspecified |
| **B410z00** | Malignant neoplasm of endocervix NOS |
| **B011100** | Malignant neoplasm of midline of tongue |
| **B305.00** | Malignant neoplasm of hand bones |
| **B323100** | Malignant melanoma of chin |
| **BBM9.00** | [M]Cystosarcoma phyllodes, malignant |
| **B055000** | Malignant neoplasm of junction of hard and soft palate |
| **BB2J.00** | [M]Squamous cell carcinoma, microinvasive |
| **B545000** | Malignant neoplasm of glomus jugulare |
| **BB5N.00** | [M]Adenomatous and adenocarcinomatous polyps of colon |
| **B060200** | Malignant neoplasm of overlapping lesion of tonsil |
| **B803500** | Carcinoma in situ of appendix |
| **BB2A.11** | [M]Epidermoid carcinoma NOS |
| **B327400** | Malignant melanoma of lower leg |
| **B836300** | Carcinoma in situ of scrotum |
| **8CP0.00** | Cancer care plan discussed with patient |
| **B827300** | Carcinoma in situ of skin of lower leg |
| **B324000** | Malignant melanoma of scalp |
| **B064.00** | Malignant neoplasm of anterior epiglottis |
| **B804000** | Carcinoma in situ of rectosigmoid junction |
| **B33y.00** | Malignant neoplasm of other specified skin sites |
| **ZV67600** | [V]Follow-up examination aft surgery for malignant neoplasm |
| **BBL7111** | [M]Adenosarcoma |
| **B336.00** | Malignant neoplasm of skin of upper limb and shoulder |
| **BBP3.11** | [M]Sarcomatoid mesothelioma |
| **BBB5.00** | [M]Adenocarcinoma with apocrine metaplasia |
| **BB5X100** | [M]Clear cell adenocarcinoma NOS |
| **BB5Vz00** | [M]Pituitary adenoma or carcinoma NOS |
| **B824.00** | Carcinoma in situ of scalp and skin of neck |
| **B8yyz00** | Carcinoma in situ of other specified site NOS |
| **B135.00** | Malignant neoplasm of appendix |
| **B808z00** | Carcinoma in situ of liver or biliary system NOS |
| **B430100** | Malignant neoplasm of fundus of corpus uteri |
| **BBJH.00** | [M]Dedifferentiated liposarcoma |
| **B080.00** | Malignant neoplasm of postcricoid region |
| **B14z.00** | Malignant neoplasm rectum,rectosigmoid junction and anus NOS |
| **B500200** | Malignant neoplasm of crystalline lens |
| **B06..00** | Malignant neoplasm of oropharynx |
| **B213000** | Malignant neoplasm of arytenoid cartilage |
| **B31z.00** | Malignant neoplasm of connective and soft tissue, site NOS |
| **BB1P.00** | [M]Non-small cell carcinoma |
| **B823400** | Carcinoma in situ of skin of nose |
| **B81y500** | Carcinoma in situ of mastoid air cells |
| **B800000** | Carcinoma in situ of lip |
| **BB5D513** | [M]Liver cell carcinoma |
| **BB3F.00** | [M]Basal cell carcinoma, infiltrative |
| **B25..00** | Malig neo, overlapping lesion of heart, mediastinum & pleura |
| **B32..00** | Malignant melanoma of skin |
| **B17yz00** | Malignant neoplasm of specified site of pancreas NOS |
| **B522.00** | Malignant neoplasm of spinal cord |
| **B304000** | Malignant neoplasm of scapula |
| **B05z000** | Kaposi's sarcoma of palate |
| **B175.00** | Malignant neoplasm, overlapping lesion of pancreas |
| **BB69100** | [M]Sebaceous adenocarcinoma |
| **BBJ7.00** | [M]Pleomorphic liposarcoma |
| **B828000** | Melanoma in situ of lip |
| **B05z.00** | Malignant neoplasm of mouth NOS |
| **B325700** | Malignant melanoma of back |
| **B325800** | Malignant melanoma of chest wall |
| **BB5W111** | [M]Hurthle cell adenocarcinoma |
| **B335.00** | Malignant neoplasm of skin of trunk, excluding scrotum |
| **B812300** | Carcinoma in situ of middle lobe bronchus and lung |
| **B13z.11** | Colonic cancer |
| **B337900** | Malignant neoplasm of skin of great toe |
| **B510300** | Malignant neoplasm of globus pallidus |
| **B430200** | Malignant neoplasm of endometrium of corpus uteri |
| **B350.00** | Malignant neoplasm of nipple and areola of male breast |
| **BBK2z00** | [M]Myoma or myosarcoma NOS |
| **B071000** | Malignant neoplasm of adenoid |
| **Byu8.00** | [X]Malignant neoplasm of male genital organs |
| **B300C00** | Malignant neoplasm of vomer |
| **B834.00** | Carcinoma in situ of prostate |
| **BB16.00** | [M]Epithelioma, malignant |
| **B62x500** | Malignant immunoproliferative small intestinal disease |
| **B333100** | Malignant neoplasm of skin of chin |
| **B055.00** | Malignant neoplasm of palate unspecified |
| **B833.00** | Carcinoma in situ other and unspecified female genital organ |
| **B323000** | Malignant melanoma of external surface of cheek |
| **B0z0.00** | Malignant neoplasm of pharynx unspecified |
| **B103.00** | Malignant neoplasm of upper third of oesophagus |
| **B300700** | Malignant neoplasm of sphenoid bone |
| **B521200** | Malignant neoplasm of cerebral pia mater |
| **B592X00** | Kaposi's sarcoma of multiple organs |
| **BB9E.00** | [M]Lobular carcinoma in situ |
| **BBL4.00** | [M]Mixed tumour, malignant, NOS |
| **B81y.00** | Carcinoma in situ of other specified part respiratory system |
| **BBK1100** | [M]Angiomyosarcoma |
| **B181.00** | Mesothelioma of peritoneum |
| **B48y.00** | Malignant neoplasm of other male genital organ |
| **BBe2.00** | [M]Neurofibrosarcoma |
| **B327600** | Malignant melanoma of heel |
| **B498.00** | Local recurrence of malignant tumour of urinary bladder |
| **B33z.11** | Squamous cell carcinoma of skin NOS |
| **BB5f300** | [M]Follicular adenocarcinoma, trabecular type |
| **B1z1z00** | Malignant neoplasm of spleen NOS |
| **B4A1000** | Malignant neoplasm of renal calyces |
| **BB1A.00** | [M]Pleomorphic carcinoma |
| **B16y.00** | Malignant neoplasm other gallbladder/extrahepatic bile duct |
| **B221000** | Malignant neoplasm of carina of bronchus |
| **ByuC000** | [X]Malignant neoplasm of other specified sites |
| **B08y.00** | Malignant neoplasm of other specified hypopharyngeal site |
| **BB9E000** | [M]Intraductal carcinoma and lobular carcinoma in situ |
| **BB26.00** | [M]Papillary squamous cell carcinoma |
| **B200300** | Malignant neoplasm of vestibule of nose |
| **B161100** | Malignant neoplasm of hepatic duct |
| **B333300** | Malignant neoplasm of skin of forehead |
| **B337z00** | Malignant neoplasm of skin of lower limb or hip NOS |
| **BB80.00** | [M]Cystadenoma and carcinoma |
| **B800600** | Carcinoma in situ of palate |
| **B500000** | Malignant neoplasm of ciliary body |
| **BBe7.00** | [M]Neurilemmoma, malignant |
| **BBY0.11** | [M]Endothelial bone sarcoma |
| **B15z.00** | Malignant neoplasm of liver and intrahepatic bile ducts NOS |
| **Byu1200** | [X]Malignant neoplasm of intestinal tract, part unspecified |
| **Byu3200** | [X]Malignant neoplasm/overlap lesion/bone+articulr cartilage |
| **Byu6.00** | [X]Malignant neoplasm of breast |
| **B161.00** | Malignant neoplasm of extrahepatic bile ducts |
| **BB81.11** | [M]Ovarian cystadenoma or carcinoma |
| **ZV10019** | [V]Personal history of malignant neoplasm of tongue |
| **B828800** | Melanoma in situ of back of hand |
| **BB5az00** | [M]Renal adenoma or carcinoma NOS |
| **B163.00** | Malignant neoplasm, overlapping lesion of biliary tract |
| **BBd2.12** | [M]Meningothelial sarcoma |
| **BB9H.00** | [M]Inflammatory carcinoma |
| **BBV..13** | [M]Periosteal osteogenic sarcoma |
| **ByuC100** | [X]Malignant neoplasm/overlap lesion/other+ill-defined sites |
| **BB19.00** | [M]Carcinoma, anaplastic type, NOS |
| **B828500** | Melanoma in situ of upper limb, including shoulder |
| **B933.11** | Cystosarcoma phyllodes |
| **BBES.00** | [M]Spindle cell melanoma, type B |
| **B002z00** | Malignant neoplasm of upper lip, inner aspect NOS |
| **B33z100** | Naevoid basal cell carcinoma syndrome |
| **B807000** | Carcinoma in situ of duodenum |
| **BB2D.00** | [M]Squamous cell carcinoma, large cell, non-keratinising |
| **BBEN.11** | [M]Juvenila melanoma |
| **BB5J.00** | [M]Adenoid cystic carcinoma |
| **B412.00** | Malignant neoplasm, overlapping lesion of cervix uteri |
| **BB5S200** | [M]Bronchiolo-alveolar adenocarcinoma |
| **B544.00** | Malignant neoplasm of carotid body |
| **B14y.00** | Malig neop other site rectum, rectosigmoid junction and anus |
| **B02z.00** | Malignant neoplasm of major salivary gland NOS |
| **BBR3.00** | [M]Choriocarcinoma combined with teratoma |
| **BBEP.00** | [M]Epithelioid cell melanoma |
| **B134.11** | Carcinoma of caecum |
| **B30z.00** | Malignant neoplasm of bone and articular cartilage NOS |
| **B303100** | Malignant neoplasm of sternum |
| **B312000** | Malignant neoplasm of connective and soft tissue of hip |
| **B17y000** | Malignant neoplasm of ectopic pancreatic tissue |
| **B03z.00** | Malignant neoplasm of gum NOS |
| **B550100** | Malignant neoplasm of cheek NOS |
| **B3...00** | Malig neop of bone, connective tissue, skin and breast |
| **BBGJ.00** | [M]Fibroxanthoma, malignant |
| **B431z00** | Malignant neoplasm of isthmus of uterine body NOS |
| **B33..14** | Malignant neoplasm of sebaceous gland |
| **B000100** | Malignant neoplasm of upper lip, lipstick area |
| **B338.00** | Squamous cell carcinoma of skin |
| **B305000** | Malignant neoplasm of carpal bone - scaphoid |
| **B325z00** | Malignant melanoma of trunk, excluding scrotum, NOS |
| **B4Az.00** | Malignant neoplasm of kidney or urinary organs NOS |
| **ByuFF00** | [X]Melanoma in situ, unspecified |
| **BB43.11** | [M]Urothelial carcinoma |
| **BBGJ.11** | [M]Fibroxanthosarcoma |
| **BBE1000** | [M]Malignant melanoma, regressing |
| **B308800** | Malignant neoplasm of first metatarsal bone |
| **B004.00** | Malignant neoplasm of lip unspecified, inner aspect |
| **BB5P.00** | [M]Solid carcinoma NOS |
| **B55z.00** | Malignant neoplasm of other and ill defined site NOS |
| **B071z00** | Malignant neoplasm of posterior wall of nasopharynx NOS |
| **B303400** | Malignant neoplasm of costo-vertebral joint |
| **B161211** | Carcinoma common bile duct |
| **B308300** | Malignant neoplasm of medial cuneiform |
| **B33..11** | Basal cell carcinoma |
| **BB81E00** | [M]Mucinous cystadenocarcinoma NOS |
| **BBB0.00** | [M]Adenosquamous carcinoma |
| **1O0..00** | Cancer confirmed |
| **B454.00** | Malignant neoplasm of vulva unspecified |
| **B3...12** | Sarcoma of bone and connective tissue |
| **B552.00** | Malignant neoplasm of abdomen |
| **B161200** | Malignant neoplasm of common bile duct |
| **B074.00** | Malignant neoplasm, overlapping lesion of nasopharynx |
| **B800z00** | Carcinoma in situ of lip, oral cavity and pharynx NOS |
| **B540z00** | Malignant neoplasm of adrenal gland NOS |
| **B336000** | Malignant neoplasm of skin of shoulder |
| **Byu4100** | [X]Malignant melanoma of skin, unspecified |
| **B4z..00** | Malignant neoplasm of genitourinary organ NOS |
| **BBE1.11** | [M]Melanocarcinoma |
| **B223.00** | Malignant neoplasm of middle lobe, bronchus or lung |
| **B307z00** | Malignant neoplasm of long bones of leg NOS |
| **B81y700** | Carcinoma in situ of ethmoidal sinus |
| **B300000** | Malignant neoplasm of ethmoid bone |
| **B311000** | Malignant neoplasm of connective and soft tissue of shoulder |
| **BB35.00** | [M]Basosquamous carcinoma |
| **B32z.00** | Malignant melanoma of skin NOS |
| **B825500** | Carcinoma in situ of skin of groin |
| **B520.00** | Malignant neoplasm of cranial nerves |
| **B451z00** | Malignant neoplasm of labia majora NOS |
| **BBB3.00** | [M]Adenocarcinoma with cartilaginous and osseous metaplasia |
| **B311300** | Malignant neoplasm of connective and soft tissue of hand |
| **B81y400** | Carcinoma in situ of Eustachian tube |
| **B306500** | Malignant sacral teratoma |
| **B48y100** | Malignant neoplasm of tunica vaginalis |
| **B345.00** | Malignant neoplasm of lower-outer quadrant of female breast |
| **B201200** | Malignant neoplasm of tympanic antrum |
| **B172.00** | Malignant neoplasm of tail of pancreas |
| **B82y.00** | Carcinoma in situ of other specified sites of skin |
| **B510.00** | Malignant neoplasm cerebrum (excluding lobes and ventricles) |
| **B59z.00** | Malignant neoplasm of unspecified site NOS |
| **BBf..00** | [M]Granular cell tumours and alveolar soft part sarcoma |
| **B826000** | Carcinoma in situ of skin of shoulder |
| **B231.00** | Malignant neoplasm of visceral pleura |
| **B327200** | Malignant melanoma of knee |
| **ZV10016** | [V]Personal history of malignant neoplasm of oesophagus |
| **BB5Sz00** | [M]Respiratory tract adenoma or adenocarcinoma NOS |
| **B323300** | Malignant melanoma of forehead |
| **BB5R900** | [M]Neuroendocrine carcinoma |
| **BBdB.00** | [M]Meningeal sarcomatosis |
| **B302.00** | Malignant neoplasm of vertebral column |
| **Byu5A00** | [X]Malignant neoplasm overlapping lesion of skin |
| **BBJ1.11** | [M]Fibroliposarcoma |
| **B823.00** | Carcinoma in situ of skin of other parts of face |
| **ByuE000** | [X]Malignant neoplasms/independent(primary)multiple sites |
| **B3...11** | Carcinoma of bone, connective tissue, skin and breast |
| **B213.00** | Malignant neoplasm of laryngeal cartilage |
| **B505.00** | Malignant neoplasm of retina |
| **BB29.00** | [M]Squamous cell carcinoma in situ NOS |
| **B808500** | Carcinoma in situ of common bile duct |
| **B502.00** | Malignant neoplasm of lacrimal gland |
| **Byu5B00** | [X]Kaposi's sarcoma of other sites |
| **BB85000** | [M]Signet ring cell carcinoma |
| **BB21.00** | [M]Papillary carcinoma in situ |
| **B24..00** | Malignant neoplasm of thymus, heart and mediastinum |
| **BBL0.00** | [M]Endometrial stromal sarcoma |
| **B308B00** | Malignant neoplasm of fourth metatarsal bone |
| **B826200** | Carcinoma in situ of skin of lower arm |
| **B341.00** | Malignant neoplasm of central part of female breast |
| **B002.00** | Malignant neoplasm of upper lip, inner aspect |
| **B500100** | Malignant neoplasm of iris |
| **B450.00** | Malignant neoplasm of vagina |
| **B802100** | Carcinoma in situ of fundus of stomach |
| **B43y.00** | Malignant neoplasm of other site of uterine body |
| **BBQ7213** | [M]Teratoblastoma, malignant |
| **B151200** | Malignant neoplasm of intrahepatic biliary passages |
| **B833z00** | Carcinoma in situ of female genital organs NOS |
| **BBE1.14** | [M]Naevocarcinoma |
| **B0...11** | Carcinoma of lip, oral cavity and pharynx |
| **BBf2.00** | [M]Alveolar soft part sarcoma |
| **B300300** | Malignant neoplasm of nasal bone |
| **B808.11** | Carcinoma in situ of biliary system |
| **BB82100** | [M]Mucinous adenocarcinoma |
| **B303500** | Malignant neoplasm of xiphoid process |
| **B152.00** | Malignant neoplasm of liver unspecified |
| **B553.00** | Malignant neoplasm of pelvis |
| **B825300** | Carcinoma in situ of skin of back |
| **BB92.00** | [M]Comedocarcinoma, noninfiltrating |
| **B302200** | Malignant neoplasm of lumbar vertebra |
| **B133.00** | Malignant neoplasm of sigmoid colon |
| **BB5M100** | [M]Tubular adenocarcinoma |
| **BBZN.00** | [M]Ameloblastic fibrosarcoma |
| **B11y000** | Malignant neoplasm of anterior wall of stomach NEC |
| **BB5a000** | [M]Renal cell carcinoma |
| **B336200** | Malignant neoplasm of skin of fore-arm |
| **BB60100** | [M]Skin appendage carcinoma |
| **B800100** | Carcinoma in situ of tongue |
| **B471z00** | Malignant neoplasm of descended testis NOS |
| **B510100** | Malignant neoplasm of cerebral cortex |
| **BB5W.00** | [M]Oxyphilic adenomas and adenocarcinomas |
| **BBTA.00** | [M]Kaposi's sarcoma |
| **B45z.00** | Malignant neoplasm of female genital organ NOS |

# Valvular disease (Elixhauser)

| **Read code** | **Description** |
| --- | --- |
| **G140500** | Tricuspid stenosis and insufficiency, cause unspecified |
| **791Dy00** | Other specified excision of valve of heart |
| **G540.14** | Mitral valve regurgitation |
| **7910.11** | Mitral valvuloplasty |
| **P6X..00** | Congenital malformation of tricuspid valve, unspecified |
| **7915300** | Revision of plastic repair of pulmonary valve |
| **G121.12** | Aortic regurgitation - rheumatic |
| **7912300** | Replacement of tricuspid valve NEC |
| **G11..11** | Rheumatic mitral valve disease |
| **G112.00** | Mitral stenosis with insufficiency |
| **ZV45H00** | [V]Presence of prosthetic heart valve |
| **G140412** | Tricuspid incompetence, cause unspecified |
| **791y.00** | Heart valve or adjacent structures operations OS |
| **G541212** | Aortic regurgitation alone, cause unspecified |
| **7918300** | Excision of vegetations of valve of heart |
| **G540.15** | Mitral valve prolapse |
| **G141.00** | Rheumatic pulmonary valve disease |
| **7916z00** | Open incision of valve of heart NOS |
| **G12z.00** | Rheumatic aortic valve disease NOS |
| **G114.00** | Ruptured mitral valve cusp |
| **7931000** | Inspection of valve of heart |
| **P651.00** | Fused commissure of the mitral valve |
| **7918111** | De Vega tricuspid annuloplasty |
| **G140514** | Tricuspid stenosis and regurgitation, cause unspecified |
| **G541z00** | Aortic valve disorders NOS |
| **7917y00** | Other specified closed incision of valve of heart |
| **7911500** | Transapical aortic valve implantation |
| **7914411** | Repair of valve of heart NEC |
| **7918200** | Annuloplasty of valve of heart NEC |
| **Gyu1100** | [X]Other rheumatic aortic valve diseases |
| **G540100** | Mitral incompetence, cause unspecified |
| **791D.00** | Excision of valve of heart |
| **791A.00** | Remove obstruction from structure adjacent to valve of heart |
| **7911100** | Xenograft replacement of aortic valve |
| **24D5.00** | O/E - aortic systolic murmur |
| **7913300** | Replacement of pulmonary valve NEC |
| **7918500** | Closure of pulmonary valve |
| **791..00** | Valves of heart and adjacent structures operations |
| **G112.13** | Mitral stenosis with regurgitation |
| **7912511** | Tricuspid valve repair NEC |
| **A932200** | Syphilitic endocarditis of aortic valve |
| **G11..00** | Mitral valve diseases |
| **7911600** | Transluminal aortic valve implantation |
| **G140112** | Tricuspid incompetence - rheumatic |
| **G541.00** | Aortic valve disorders |
| **791C500** | Aortoventriculoplasty with pulmonary valve autograft |
| **7913z00** | Plastic repair of pulmonary valve NOS |
| **7919500** | Percutaneous transluminal pulmonary valve perfor dilation |
| **7911y00** | Other specified plastic repair of aortic valve |
| **G541300** | Aortic stenosis alone, cause unspecified |
| **Pyu2200** | [X]Other congenital malformations of pulmonary valve |
| **G140z00** | Rheumatic tricuspid valve disease NOS |
| **A932300** | Syphilitic endocarditis of tricuspid valve |
| **G543.00** | Pulmonary valve disorders |
| **G544X00** | Multiple valve disease, unspecified |
| **7915000** | Revision of plastic repair of mitral valve |
| **7914300** | Replacement of valve of heart NEC |
| **P65z.00** | Congenital mitral stenosis NOS |
| **7915y00** | Other specified revision of plastic repair of valve of heart |
| **G542X00** | Nonrheumatic tricuspid valve disorder, unspecified |
| **791A300** | Repair of supraaortic stenosis |
| **Gyu1200** | [X]Other tricuspid valve diseases |
| **7910.12** | Replacement of mitral valve |
| **G110.11** | Rheumatic mitral stenosis |
| **24G..00** | Aortic valve gradient |
| **P641.00** | Bicuspid aortic valve |
| **G542.00** | Tricuspid valve disorders, non-rheumatic |
| **G14021X** | Rheumatic tricuspid stenosis and regurgitation |
| **G542z00** | Tricuspid valve disorders NOS |
| **7912.12** | Tricuspid valvuloplasty |
| **7910100** | Xenograft replacement of mitral valve |
| **7915.00** | Revision of plastic repair of valve of heart |
| **G54z.00** | Endocarditis, valve unspecified |
| **G122.00** | Rheumatic aortic stenosis with insufficiency |
| **7911000** | Allograft replacement of aortic valve |
| **P602z00** | Congenital pulmonary stenosis NOS |
| **G132.13** | Mitral regurgitation and aortic stenosis |
| **P601000** | Hypoplasia of pulmonary valve |
| **7N40000** | [SO]Mitral valve |
| **7910000** | Allograft replacement of mitral valve |
| **G140200** | Rheumatic tricuspid stenosis and insufficiency |
| **7918y00** | Other specified other open operation on valve of heart |
| **7910z00** | Plastic repair of mitral valve NOS |
| **P66..00** | Congenital mitral insufficiency |
| **P60zz00** | Other pulmonary valve anomaly NOS |
| **P65..00** | Congenital mitral stenosis |
| **7911411** | Aortic valve repair NEC |
| **7913411** | Pulmonary valve repair NEC |
| **7914211** | Edwards prosthetic replacement of valve of heart |
| **7918100** | Annuloplasty of tricuspid valve |
| **7917.00** | Closed incision of heart valve |
| **ZV42200** | [V]Heart valve transplanted |
| **7N40300** | [SO]Pulmonary valve |
| **7914.11** | Replacement of unspecified valve of heart |
| **G130.00** | Mitral and aortic stenosis |
| **G111.00** | Rheumatic mitral insufficiency |
| **G13z.00** | Mitral and aortic valve disease NOS |
| **791D000** | Tricuspid valvectomy |
| **24D7.00** | O/E -pulmonary systolic murmur |
| **24D8.00** | O/E-pulmonary diastolic murmur |
| **G542200** | Nonrheumatic tricuspid valve stenosis with insufficiency |
| **G544000** | Disorders of both aortic and tricuspid valves |
| **7910411** | Mitral valve repair NEC |
| **7914100** | Xenograft replacement of valve of heart NEC |
| **7917000** | Closed mitral valvotomy |
| **7911.12** | Replacement of aortic valve |
| **7911.00** | Plastic repair of aortic valve |
| **P63..00** | Congenital aortic valve stenosis |
| **7912000** | Allograft replacement of tricuspid valve |
| **P6yyC00** | Fusion of mitral valve cusps |
| **G14z.11** | Rheumatic valvulitis, chronic NOS |
| **SP00200** | Mechanical complication of heart valve prosthesis |
| **Gy0..00** | Cardiovascular syphilis |
| **P61..00** | Congenital tricuspid atresia and stenosis |
| **P652.00** | Parachute deformity of the mitral valve |
| **7917200** | Closed tricuspid valvotomy |
| **7918.00** | Other open operations on valve of heart |
| **G12..00** | Rheumatic aortic valve disease |
| **G110.00** | Mitral stenosis |
| **7914212** | Starr prosthetic replacement of valve of heart |
| **G541500** | Aortic stenosis |
| **P712.13** | Postductal aortic stenosis |
| **7912100** | Xenograft replacement of tricuspid valve |
| **G140300** | Tricuspid stenosis, cause unspecified |
| **P61z.00** | Congenital tricuspid atresia or stenosis NOS |
| **7919z00** | Therapeutic transluminal operation on heart valve NOS |
| **A93..00** | Cardiovascular syphilis |
| **791z.00** | Heart valve and adjacent structures operations NOS |
| **791A200** | Repair of subaortic stenosis |
| **G140100** | Rheumatic tricuspid insufficiency |
| **7914.00** | Plastic repair of unspecified valve of heart |
| **P60z.00** | Other pulmonary valve anomalies |
| **G54z000** | Incompetence of unspecified heart valve |
| **G141z00** | Rheumatic pulmonary valve disease NOS |
| **7915200** | Revision of plastic repair of tricuspid valve |
| **7915100** | Revision of plastic repair of aortic valve |
| **P601z00** | Congenital atresia of pulmonary valve NOS |
| **7918z00** | Other open operation on valve of heart NOS |
| **G54z100** | Stenosis of unspecified heart valve |
| **24D..11** | O/E - aortic murmur |
| **7919.00** | Therapeutic transluminal operations on valve of heart |
| **P6yy700** | Atresia of heart valve NEC |
| **7914200** | Prosthetic replacement of valve of heart NEC |
| **G54z500** | Valvular heart disease |
| **G111.11** | Mitral incompetence - rheumatic |
| **791Ay00** | Removal of obstruction from struct adjacent heart valve OS |
| **P64..00** | Congenital aortic valve insufficiency |
| **7912z00** | Plastic repair of tricuspid valve NOS |
| **7N40.00** | [SO]Valve of heart |
| **TB01200** | Implant of heart valve prosthesis + complication, no blame |
| **G540z00** | Mitral valve disorders NOS |
| **ZVu6e00** | [X]Presence of other heart valve replacement |
| **G54zz00** | Endocarditis, valve unspecified, NOS |
| **G540.12** | Mitral valve insufficiency |
| **Gyu5800** | [X]Other pulmonary valve disorders |
| **7914600** | Replacement of truncal valve |
| **P60..00** | Pulmonary valve anomalies |
| **P610.00** | Congenital tricuspid atresia |
| **P600.00** | Pulmonary valve anomaly, unspecified |
| **790Dy00** | Other specified creation of valved cardiac conduit |
| **7912500** | Tricuspid valvuloplasty NEC |
| **7916.00** | Open incision of heart valve |
| **G543215** | Pulmonary regurgitation, cause unspecified |
| **7914000** | Allograft replacement of valve of heart NEC |
| **G543400** | Pulmonary valve stenosis with insufficiency |
| **G11z.00** | Mitral valve disease NOS |
| **P6y0.00** | Subaortic stenosis |
| **G543012** | Pulmonary regurgitation, non-rheumatic |
| **7918000** | Annuloplasty of mitral valve |
| **7916000** | Open mitral valvotomy |
| **G542011** | Tricuspid insufficiency, non-rheumatic |
| **7910212** | Bjork-Shiley prosthetic replacement of mitral valve |
| **G54z300** | Endocarditis, valve unspecified, OS |
| **G544200** | Combined disorders of mitral, aortic and tricuspid valves |
| **P60z000** | Congenital insufficiency of the pulmonary valve |
| **G112.12** | Mitral stenosis with incompetence |
| **G541400** | Aortic valve stenosis with insufficiency |
| **14S4.00** | H/O: heart valve recipient |
| **7902000** | Correct Fallot tetralogy- valved right ventr outflow conduit |
| **7914y00** | Other specified plastic repair of unspecified valve of heart |
| **G111.12** | Mitral regurgitation - rheumatic |
| **G120.00** | Rheumatic aortic stenosis |
| **7913.12** | Replacement of pulmonary valve |
| **7N40400** | [SO]Truncal valve |
| **7910300** | Replacement of mitral valve NEC |
| **P611.00** | Congenital tricuspid stenosis |
| **P64z.00** | Congenital aortic valve insufficiency NOS |
| **7917z00** | Closed incision of valve of heart NOS |
| **7N40100** | [SO]Aortic valve |
| **G541100** | Aortic stenosis, non-rheumatic |
| **G54z014** | Insufficiency of unspecified heart valve |
| **A932.11** | Syphilitic valve disease |
| **G542012** | Tricuspid regurgitation, non-rheumatic |
| **G13..00** | Diseases of mitral and aortic valves |
| **P640.00** | Congenital aortic valve insufficiency, unspecified |
| **SP00400** | Infect and inflammatory reaction due to cardiac valve pros |
| **P722400** | Supra-valvular aortic stenosis |
| **P602.00** | Congenital pulmonary stenosis |
| **7A65y00** | Other specified repair of valve of vein |
| **7916200** | Open tricuspid valvotomy |
| **7914500** | Truncal valve repair |
| **791D100** | Pulmonary valvectomy |
| **G54z013** | Regurgitation of unspecified heart valve |
| **7918400** | Closure of tricuspid valve |
| **G141000** | Rheumatic pulmonary stenosis |
| **G541700** | Aortic valve calcification |
| **G14021Y** | Rheumatic tricuspid stenosis and incompetence |
| **G132.12** | Mitral incompetence and aortic stenosis |
| **7919y00** | Therapeutic transluminal operation on heart valve OS |
| **7913100** | Xenograft replacement of pulmonary valve |
| **7910200** | Prosthetic replacement of mitral valve |
| **A932.00** | Syphilitic endocarditis |
| **7A65100** | Interposition of valve of vein |
| **32A3.00** | ECG: P mitrale |
| **7914z00** | Plastic repair of unspecified valve of heart NOS |
| **A932100** | Syphilitic endocarditis of mitral valve |
| **G542000** | Tricuspid incompetence, non-rheumatic |
[truncated: 6,475 more chars]
