# Supplementary material for: Patient Characteristics and Practice Variation Associated With New Community Prescription of Benzodiazepine and z‐Drug Hypnotics After Critical Illness: A Retrospective Cohort Study Using the UK Clinical Practice Research Datalink
Source: Pharmacoepidemiol Drug Saf. 2024 Nov 27;33(12):e70056. doi: 10.1002/pds.70056 (PMC11602247; doi:10.1002/pds.70056)
Supplement: Supplementary file 2 — Tables S1‐S4. [file PDS-33-e70056-s002.docx]

*Patient characteristics and practice variation associated with new community prescription of benzodiazepine and z-drug hypnotics after critical illness: a retrospective cohort study using the UK Clinical Practice Research Datalink*

**Supplementary Materials**

[Supplemental Table S1. Comparison of patients included in complete case analysis versus excluded from the final multivariable model examining the association between patient characteristics and new benzodiazepine prescribing after critical illness. 2](#_Toc178679162)

[Supplemental Table S2. Sensitivity analysis of the association of patient characteristics and new benzodiazepine or z-drug prescribing, excluding patients who died or were readmitted to hospital within 30 days (n=38,742). 4](#_Toc178679163)

[Supplemental Table S3. Sensitivity analysis of the association of patient characteristics and new benzodiazepine or z-drug prescribing, excluding patients who died or were readmitted to hospital within 90 days (n=30,686). 6](#_Toc178679164)

[Supplemental Table S4. Sensitivity analysis of the association between patient characteristics and new benzodiazepine or z-drug prescription after redefining treatment-naïve patients as those without benzodiazepine or z-drug prescription within 365 days of index hospitalisation (n=51,592). 8](#_Toc178679165)

#### **Supplemental Table S1.** Comparison of patients included in complete case analysis versus excluded from the final multivariable model examining the association between patient characteristics and new benzodiazepine prescribing after critical illness.

| **Patient characteristics** | | **Missing values per variable**  **Denominator=52,846 (%)** | **Excluded from complete case analysis (any missing data)**  **Denominator=4,015 (%)** | **Included in complete case analysis**  **Denominator=48,831 (%)** |
| --- | --- | --- | --- | --- |
| Sex | *Missing*  Male  Female | 0 (0) | 2235 (55.7)  1780 (44.3) | 28281 (57.9)  20550 (42.1) |
| Age group | *Missing*  18-49  50-64  65-79  80+ | 0 (0) | 781 (19.5)  930 (23.2)  1564 (39.0)  740 (18.4) | 10274 (21.0)  13062 (26.7)  18752 (38.4)  6743 (13.8) |
| Ethnicity | *Missing*  Asian  Black  Mixed  White  Other | 227 (0.4) | 167 (4.4)  94 (2.5)  16 (0.4)  3475 (91.7)  36 (1.0) | 2738 (5.6)  1959 (4.0)  295 (0.6)  43040 (88.1)  799 (1.6) |
| IMD quintile | *Missing*  1, Least deprived  2  3  4  5, Most deprived | 83 (0.2) | 749 (19.0)  756 (19.2)  747 (19.0)  744 (18.9)  936 (23.8) | 9215 (18.9)  9590 (19.6)  9386 (19.2)  10249 (21.0)  10391 (21.3) |
| Modified Elixhauser comorbidity count^1^ | *Missing*  None  1  2  3  4  5 or more | 0 (0) | 600 (14.9)  750 (18.7)  703 (17.5)  606 (15.1)  502 (12.5)  854 (21.3) | 6434 (13.2)  9132 (18.7)  9047 (18.5)  7821 (16.0)  6048 (12.4)  10349 (21.2) |
| History of insomnia | | 0 (0) | 327 (8.1) | 3755 (7.7) |
| History of anxiety or depression | | 0 (0) | 1214 (30.2) | 15243 (31.2) |
| History of psychoses | | 0 (0) | 92 (2.3) | 996 (2.0) |
| History of alcohol misuse | | 0 (0) | 434 (10.8) | 5865 (12.0) |
| History of drug misuse | | 0 (0) | 80 (2.0) | 1182 (2.4) |
| History of opioid prescription | | 0 (0) | 1182 (29.4) | 13012 (26.6) |
| History of gabapentin/pregabalin prescription | | 0 (0) | 209 (5.2) | 3006 (6.2) |
| Number hospital admissions (1 year) | *Missing*  None  1  2  3+ | 0 (0) | 1611 (40.1)  999 (24.9)  562 (14.0)  843 (21.0) | 16260 (33.3)  13260 (27.2)  7643 (15.7)  11668 (23.9) |
| Number primary care consultations (1 year) | *Missing*  0-2  3-7  8-14  15+ | 0 (0) | 1157 (28.8)  938 (23.4)  984 (24.5)  936 (23.3) | 12067 (24.7)  11672 (23.9)  13185 (27.0)  11907 (24.4) |
| Year of hospital admission | *Missing*  2010  2018 | 0 (0) | 2061 (51.3)  1954 (48.7) | 19049 (39.0)  29782 (61.0) |
| Hospital admission method | *Missing*  Elective  Emergency  Transfer/Other | 3 (<0.1) | 1232 (30.7)  2367 (59.0)  413 (10.3) | 22199 (45.5)  23172 (47.5)  3460 (7.1) |
| Primary condition  at index hospitalisation | *Missing*  Circulatory  Neoplasms  Digestive  Injury  Respiratory  Musculoskeletal  Genitourinary  Infectious  Abnormal finding^2^  Other | 0 (0) | 1095 (27.3)  503 (12.5)  479 (11.9)  550 (13.7)  311 (7.7)  258 (6.4)  180 (4.5)  145 (3.6)  109 (2.7)  385 (9.6) | 14139 (29.0)  9679 (19.8)  5294 (10.8)  4393 (9.0)  4362 (8.9)  1868 (3.8)  1617 (3.3)  1422 (2.9)  1189 (2.4)  4868 (10.0) |
| ICU type | *Missing*  Gen/Med/Surg  Cardio/Thoracic  Neuro  Other | 1891 (3.6) | 1821 (85.7)  126 (5.9)  20 (0.9)  157 (7.4) | 34461 (70.6)  10222 (20.9)  2262 (4.6)  1886 (3.9) |
| Total organ systems supported | *Missing*  None  1  2  3 | 3721 (7.0) | 25 (8.5)  85 (28.9)  166 (56.5)  18 (6.1) | 4275 (8.8)  14948 (30.6)  27496 (56.3)  2112 (4.3) |
| Out of hours ICU discharge | *Missing*  No  Yes | 1891 (3.6) | 1638 (77.1)  486 (22.9) | 41449 (84.9)  7382 (15.1) |
| Hospital LOS | *Missing*  less than 7 days  7-13 days  14 days or more | 0 (0) | 1063 (26.5)  686 (17.1)  2266 (56.4) | 20047 (41.1)  13701 (28.1)  15083 (30.9) |
| New benzodiazepine or  z-drug prescription (outcome) | *Missing*  No  Yes | 0 (0) | 3791 (94.4)  224 (5.6) | 46286 (94.8)  2545 (5.2) |

^1^modified by removing four mental health conditions (depression, psychoses, alcohol misuse, drug misuse) for individual analysis. ^2^symptoms, signs and abnormal clinical and laboratory findings, not elsewhere classified *IMD*: index of multiple deprivation; *ICU*: intensive care unit; *Gen/Med/Surg:* General/Medical/Surgical; *LOS:* length of stay

#### **Supplemental Table S2.** Sensitivity analysis of the association of patient characteristics and new benzodiazepine or z-drug prescribing, excluding patients who died or were readmitted to hospital within 30 days (n=38,742).

| **Patient characteristics** | | **Received Rx (%)** | **OR multivariable (95% CI)**  **Complete cases n=35,917** |
| --- | --- | --- | --- |
| Sex | Male  Female | 1086 (4.9)  739 (4.5) | Reference  0.90 (0.81-1.00) |
| Age group | 18-49  50-64  65-79  80+ | 357 (4.3)  532 (5.2)  699 (4.7)  237 (4.4) | Reference  0.92 (0.79-1.07)  0.83 (0.70-0.98)  0.80 (0.65-0.98) |
| Ethnicity | Asian  Black  Mixed  White  Other | 74 (3.6)  36 (2.5)  6 (2.7)  1691 (4.9)  14 (2.3) | 0.88 (0.68-1.12)  0.55 (0.38-0.81)  0.55 (0.23-1.36)  Reference  0.58 (0.34-0.99) |
| IMD quintile | 1, Least deprived  2  3  4  5, Most deprived | 368 (5.0)  404 (5.3)  336 (4.5)  337 (4.2)  378 (4.6) | 1.16 (0.98-1.36)  1.22 (1.04-1.42)  0.98 (0.83-1.16)  0.98 (0.83-1.15)  Reference |
| Modified Elixhauser comorbidity count^1^ | None  1  2  3  4  5 or more | 217 (4.0)  330 (4.4)  390 (5.4)  307 (5.0)  240 (5.1)  341 (4.5) | Reference  1.03 (0.85-1.25)  1.22 (1.01-1.48)  1.04 (0.85-1.28)  1.09 (0.88-1.36)  0.91 (0.73-1.12) |
| History of insomnia | No  Yes | 1530 (4.3)  295 (10.1) | Reference  2.16 (1.86-2.50) |
| History of anxiety or depression | No  Yes | 1079 (4.0)  746 (6.3) | Reference  1.42 (1.27-1.59) |
| History of psychoses | No  Yes | 1774 (4.7)  51 (7.2) | Reference  1.32 (0.97-1.81) |
| History of alcohol misuse | No  Yes | 1544 (4.5)  281 (6.3) | Reference  1.18 (1.01-1.37) |
| History of drug misuse | No  Yes | 1764 (4.7)  61 (6.9) | Reference  1.13 (0.84-1.53) |
| History of opioid prescription | No  Yes | 1193 (4.2)  632 (6.3) | Reference  1.38 (1.23-1.55) |
| History of gabapentin/pregabalin prescription | No  Yes | 1704 (4.7)  121 (5.6) | Reference  0.98 (0.79-1.21) |
| Number hospital admissions  (1 year) | None  1  2  3+ | 564 (4.1)  499 (4.6)  349 (5.7)  413 (5.2) | Reference  1.08 (0.94-1.24)  1.28 (1.09-1.49)  1.14 (0.98-1.32) |
| Number primary care consultations (1 year) | 0-2  3-7  8-14  15+ | 428 (4.3)  378 (3.9)  492 (4.7)  527 (6.0) | Reference  1.01 (0.87-1.18)  1.14 (0.98-1.32)  1.35 (1.16-1.58) |
| Year of admission | 2010  2018 | 921 (5.9)  904 (3.9) | Reference  0.60 (0.54-0.67) |
| Hospital admission method | Elective  Emergency  Transfer/Other | 834 (4.5)  873 (5.0)  118 (4.1) | Reference  1.42 (1.24-1.63)  1.23 (0.99-1.54) |
| Primary condition  at index hospitalisation | Circulatory  Neoplasms  Digestive  Injury  Respiratory  Musculoskeletal  Genitourinary  Infectious  Abnormal findings^2^  Other | 588 (5.1)  420 (5.6)  166 (4.0)  188 (5.3)  138 (4.1)  82 (4.7)  34 (3.1)  34 (3.5)  60 (6.7)  115 (2.9) | Reference  1.36 (1.16-1.59)  0.73 (0.60-0.90)  0.96 (0.79-1.18)  0.65 (0.52-0.81)  1.03 (0.77-1.36)  0.66 (0.46-0.97)  0.65 (0.44-0.96)  1.21 (0.89-1.64)  0.66 (0.52-0.83) |
| ICU type | Gen/Med/Surg  Cardio/Thoracic  Neuro  Other | 1169 (4.4)  442 (5.4)  87 (5.7)  32 (2.1) | Reference  1.29 (1.11-1.49)  1.35 (1.07-1.71)  0.70 (0.48-1.01) |
| Total organ systems supported | None  1  2  3 | 127 (3.8)  442 (3.9)  1045 (5.1)  70 (5.6) | Reference  0.98 (0.79-1.20)  1.18 (0.97-1.44)  1.24 (0.90-1.69) |
| Out of hours ICU discharge | No  Yes | 1438 (4.5)  292 (5.1) | Reference  1.09 (0.95-1.25) |
| Hospital LOS | less than 7 days  7-13 days  14 days or more | 635 (3.8)  524 (4.9)  666 (5.9) | Reference  1.18 (1.04-1.34)  1.29 (1.13-1.48) |

Multivariable models were adjusted for all covariates shown.

^1^modified by removing four mental health conditions (depression, psychoses, alcohol misuse, drug misuse) for individual analysis. ^2^symptoms, signs and abnormal clinical and laboratory findings, not elsewhere classified

*Rx:* prescription (here meaning benzodiazepine or z-drug); *IMD*: index of multiple deprivation; *ICU*: intensive care unit; *Gen/Med/Surg:* General/Medical/Surgical; *LOS:* length of stay

#### **Supplemental Table S3.** Sensitivity analysis of the association of patient characteristics and new benzodiazepine or z-drug prescribing, excluding patients who died or were readmitted to hospital within 90 days (n=30,686).

| **Patient characteristics** | | **Received Rx (%)** | **OR multivariable (95% CI)**  **Complete cases n=28,429** |
| --- | --- | --- | --- |
| Sex | Male  Female | 778 (4.4)  497 (3.9) | Reference  0.88 (0.77-1.00) |
| Age group | 18-49  50-64  65-79  80+ | 269 (3.9)  370 (4.6)  487 (4.2)  149 (3.6) | Reference  0.88 (0.73-1.05)  0.79 (0.65-0.96)  0.72 (0.56-0.93) |
| Ethnicity | Asian  Black  Mixed  White  Other | 51 (3.1)  26 (2.3)  <5 (<2.7)  1183 (4.4)  8 (1.6) | 0.85 (0.63-1.15)  0.59 (0.37-0.93)  0.46 (0.15-1.46)  Reference  0.47 (0.23-0.95) |
| IMD quintile | 1, Least deprived  2  3  4  5, Most deprived | 266 (4.5)  291 (4.8)  228 (3.9)  238 (3.8)  251 (3.9) | 1.29 (1.06-1.57)  1.35 (1.12-1.63)  1.01 (0.83-1.23)  1.05 (0.87-1.28)  Reference |
| Modified Elixhauser comorbidity count^1^ | None  1  2  3  4  5 or more | 167 (3.5)  243 (3.9)  274 (4.8)  212 (4.4)  170 (4.7)  209 (3.8) | Reference  1.02 (0.82-1.27)  1.22 (0.97-1.52)  1.02 (0.80-1.30)  1.13 (0.88-1.46)  0.85 (0.65-1.10) |
| History of insomnia | No  Yes | 1067 (3.8)  208 (9.1) | Reference  2.26 (1.90-2.70) |
| History of anxiety or depression | No  Yes | 749 (3.5)  526 (5.7) | Reference  1.53 (1.34-1.74) |
| History of psychoses | No  Yes | 1239 (4.1)  36 (6.7) | Reference  1.40 (0.97-2.03) |
| History of alcohol misuse | No  Yes | 1090 (4.0)  185 (5.5) | Reference  1.13 (0.94-1.35) |
| History of drug misuse | No  Yes | 1228 (4.1)  47 (6.7) | Reference  1.25 (0.89-1.77) |
| History of opioid prescription | No  Yes | 873 (3.8)  402 (5.4) | Reference  1.31 (1.14-1.51) |
| History of gabapentin/pregabalin prescription | No  Yes | 1191 (4.1)  84 (5.4) | Reference  1.08 (0.83-1.39) |
| Number hospital admissions  (1 year) | None  1  2  3+ | 443 (3.8)  382 (4.3)  245 (5.2)  205 (3.9) | Reference  1.09 (0.93-1.28)  1.24 (1.03-1.49)  0.92 (0.76-1.12) |
| Number primary care consultations (1 year) | 0-2  3-7  8-14  15+ | 316 (3.9)  263 (3.4)  347 (4.2)  349 (5.3) | Reference  0.97 (0.81-1.17)  1.14 (0.96-1.36)  1.41 (1.17-1.70) |
| Year of admission | 2010  2018 | 642 (5.1)  633 (3.5) | Reference  0.61 (0.53-0.69) |
| Hospital admission method | Elective  Emergency  Transfer/Other | 585 (4.0)  603 (4.5)  87 (3.5) | Reference  1.36 (1.15-1.60)  1.14 (0.88-1.47) |
| Primary condition  at index hospitalisation | Circulatory  Neoplasms  Digestive  Injury  Respiratory  Musculoskeletal  Genitourinary  Infectious  Abnormal findings^2^  Other | 482 (4.9)  207 (4.2)  117 (3.7)  148 (5.0)  92 (3.5)  70 (4.6)  20 (2.5)  22 (2.9)  35 (5.2)  82 (2.4) | Reference  1.11 (0.91-1.36)  0.76 (0.59-0.97)  0.99 (0.78-1.25)  0.61 (0.47-0.80)  1.09 (0.79-1.49)  0.59 (0.37-0.96)  0.56 (0.34-0.90)  0.99 (0.66-1.48)  0.66 (0.50-0.86) |
| ICU type | Gen/Med/Surg  Cardio/Thoracic  Neuro  Other | 766 (3.8)  360 (5.2)  62 (5.0)  17 (1.3) | Reference  1.38 (1.16-1.64)  1.39 (1.05-1.84)  0.58 (0.35-0.97) |
| Total organ systems supported | None  1  2  3 | 79 (2.9)  277 (3.2)  769 (4.7)  49 (5.2) | Reference  1.10 (0.84-1.43)  1.44 (1.12-1.85)  1.50 (1.02-2.20) |
| Out of hours ICU discharge | No  Yes | 1005 (4.0)  200 (4.4) | Reference  1.07 (0.91-1.27) |
| Hospital LOS | less than 7 days  7-13 days  14 days or more | 467 (3.4)  372 (4.4)  436 (5.1) | Reference  1.19 (1.03-1.38)  1.26 (1.07-1.48) |

Multivariable models were adjusted for all covariates shown.

^1^modified by removing four mental health conditions (depression, psychoses, alcohol misuse, drug misuse) for individual analysis. ^2^symptoms, signs and abnormal clinical and laboratory findings, not elsewhere classified

*Rx:* prescription (here meaning benzodiazepine or z-drug); *IMD*: index of multiple deprivation; *ICU*: intensive care unit; *Gen/Med/Surg:* General/Medical/Surgical; *LOS:* length of stay

#### **Supplemental Table S4.** Sensitivity analysis of the association between patient characteristics and new benzodiazepine or z-drug prescription after redefining treatment-naïve patients as those without benzodiazepine or z-drug prescription within 365 days of index hospitalisation (n=51,592).

| **Patient characteristics** | | **Received Rx (%)** | **OR multivariable (95% CI)**  **Complete cases n=47,677** |
| --- | --- | --- | --- |
| Sex | Male  Female | 1525 (5.1)  1044 (4.8) | Reference  0.91 (0.83-0.99) |
| Age group | 18-49  50-64  65-79  80+ | 475 (4.4)  747 (5.5)  1006 (5.1)  341 (4.6) | Reference  0.98 (0.85-1.12)  0.88 (0.77-1.02)  0.82 (0.69-0.97) |
| Ethnicity | Asian  Black  Mixed  White  Other | 115 (4.0)  49 (2.4)  11 (3.6)  2367 (5.2)  20 (2.4) | 0.90 (0.73-1.10)  0.53 (0.39-0.73)  0.73 (0.39-1.39)  Reference  0.57 (0.36-0.90) |
| IMD quintile | 1, Least deprived  2  3  4  5, Most deprived | 496 (5.1)  565 (5.6)  483 (4.9)  493 (4.6)  529 (4.8) | 1.14 (0.99-1.31)  1.23 (1.08-1.41)  1.04 (0.90-1.19)  1.02 (0.89-1.16)  Reference |
| Modified Elixhauser comorbidity count^1^ | None  1  2  3  4  5 or more | 6613 (95.8)  9208 (95.3)  9016 (94.6)  7788 (94.8)  6069 (94.7)  10329 (94.9) | Reference  1.02 (0.87-1.20)  1.13 (0.96-1.34)  0.99 (0.83-1.19)  1.03 (0.85-1.24)  0.94 (0.79-1.13) |
| History of insomnia | No  Yes | 2217 (4.6)  352 (9.4) | Reference  1.86 (1.64-2.12) |
| History of anxiety or depression | No  Yes | 1561 (4.4)  1008 (6.4) | Reference  1.37 (1.25-1.50) |
| History of psychoses | No  Yes | 2499 (4.9)  70 (6.8) | Reference  1.29 (0.99-1.68) |
| History of alcohol misuse | No  Yes | 2193 (4.8)  376 (6.2) | Reference  1.13 (0.99-1.28) |
| History of drug misuse | No  Yes | 2483 (4.9)  86 (7.3) | Reference  1.24 (0.97-1.59) |
| History of opioid prescription | No  Yes | 1635 (4.3)  934 (6.8) | Reference  1.45 (1.32-1.60) |
| History of gabapentin/pregabalin prescription | No  Yes | 2382 (4.9)  187 (6.1) | Reference  1.03 (0.87-1.22) |
| Number hospital admissions  (1 year) | None  1  2  3+ | 754 (4.3)  688 (4.9)  477 (6.0)  650 (5.4) | Reference  1.05 (0.94-1.18)  1.21 (1.06-1.38)  1.05 (0.93-1.19) |
| Number primary care consultations (1 year) | 0-2  3-7  8-14  15+ | 567 (4.4)  525 (4.2)  697 (5.0)  780 (6.3) | Reference  1.05 (0.92-1.20)  1.22 (1.08-1.39)  1.43 (1.25-1.63) |
| Year of admission | 2010  2018 | 1262 (6.1)  1307 (4.2) | Reference  0.62 (0.57-0.68) |
| Hospital admission method | Elective  Emergency  Transfer/Other | 1108 (4.8)  1294 (5.2)  167 (4.4) | Reference  1.37 (1.22-1.54)  1.18 (0.98-1.43) |
| Primary condition  at index hospitalisation | Circulatory  Neoplasms  Digestive  Injury  Respiratory  Musculoskeletal  Genitourinary  Infectious  Abnormal findings^2^  Other | 768 (5.1)  632 (6.3)  261 (4.6)  251 (5.3)  204 (4.5)  94 (4.6)  61 (3.5)  61 (4.0)  82 (6.5)  155 (3.0) | Reference  1.48 (1.29-1.69)  0.83 (0.70-0.99)  0.98 (0.82-1.16)  0.76 (0.63-0.91)  0.96 (0.74-1.24)  0.69 (0.52-0.92)  0.75 (0.56-1.01)  1.14 (0.88-1.48)  0.66 (0.54-0.81) |
| ICU type | Gen/Med/Surg  Cardio/Thoracic  Neuro  Other | 1691 (4.8)  569 (5.6)  135 (6.1)  47 (2.4) | Reference  1.30 (1.15-1.48)  1.35 (1.12-1.64)  0.71 (0.52-0.97) |
| Total organ systems supported | None  1  2  3 | 161 (3.8)  658 (4.5)  1435 (5.3)  123 (5.9) | Reference  1.09 (0.91-1.31)  1.23 (1.03-1.46)  1.32 (1.02-1.70) |
| Out of hours ICU discharge | No  Yes | 2038 (4.8)  404 (5.3) | Reference  1.08 (0.96-1.21) |
| Hospital LOS | less than 7 days  7-13 days  14 days or more | 835 (4.1)  709 (5.0)  1025 (6.1) | Reference  1.13 (1.01-1.26)  1.30 (1.16-1.45) |

Multivariable models were adjusted for all covariates shown.

^1^modified by removing four mental health conditions (depression, psychoses, alcohol misuse, drug misuse) for individual analysis. ^2^symptoms, signs and abnormal clinical and laboratory findings, not elsewhere classified

*Rx:* prescription (here meaning benzodiazepine or z-drug); *IMD*: index of multiple deprivation; *ICU*: intensive care unit; *Gen/Med/Surg:* General/Medical/Surgical; *LOS:* length of stay
